# Tripartite motif-containing 34 (TRIM34) protein interacts with the nucleocytoplasmic transport machinery and negatively modulates antiviral responses

**DOI:** 10.1371/journal.ppat.1014142

**Published:** 2026-05-20

**Authors:** Paula Vázquez-Utrilla, Vanessa Rivero, Marta L. DeDiego

**Affiliations:** 1 Department of Molecular and Cell Biology, Centro Nacional de Biotecnología-Consejo Superior de Investigaciones Científicas (CNB-CSIC), Madrid, Spain; 2 Escuela de Doctorado, Universidad Autónoma de Madrid (UAM)Madrid, Spain; Washington University in Saint Louis, UNITED STATES OF AMERICA

## Abstract

The tripartite motif-containing (TRIM) 34 protein is an interferon (IFN)-induced protein whose expression is upregulated after influenza A virus (IAV) infection. Here, we identify a previously unknown function for TRIM34 as a negative regulator of innate immune responses following IFN treatment and IAV infection, even in mice, suggesting that this effect is broad and conserved. Complementary overexpression and silencing experiments in cultured cells show that TRIM34 expression positively correlates with IAV titers, indicating that TRIM34 promotes viral replication, likely by counteracting antiviral responses. Moreover, we show novel interactions of TRIM34 with cellular proteins involved in the nucleocytoplasmic transport of host mRNAs and proteins, even in mock-infected cells. Remarkably, these TRIM34 interactions seem independent of TRIM34 E3 ubiquitin ligase activity and affect the nuclear import of IFN-regulatory factor 3 (IRF3) and the nuclear export of host mRNAs encoding antiviral functions, without affecting the nuclear export of viral mRNAs. These results provide a likely mechanism by which TRIM34 dampens host innate immune responses. These TRIM34-mediated effects could be further exploited to develop new antiviral drugs against IAV, and potentially other viral infections.

## Author summary

The expression of tripartite motif-containing (TRIM) 34 is induced by interferons (IFNs) and viral infections, including Influenza A virus (IAV). In this work, we show that TRIM34 counteracts innate immune responses triggered by IFN treatment and IAV infections in both cell culture systems and mice. Notably, TRIM34 expression levels positively correlated with IAV titers *in vitro,* indicating that TRIM34 supports viral replication, likely through its ability to suppress antiviral responses. Mechanistically, TRIM34 interacted with proteins involved in the transport of host proteins and mRNAs between the nucleus and the cytoplasm, even in mock-infected cells, being this effect likely independent of TRIM34 E3 ubiquitin ligase activity. Remarkably, these interactions inhibit the import of IFN-regulatory factor 3 (IRF3) from the cytoplasm to the nucleus, and lead to the nuclear retention of host mRNAs encoding antiviral factors. In contrast, the nuclear export of viral mRNAs is not affected. This selective disruption of host nucleocytoplasmic trafficking provides a plausible molecular mechanism for TRIM34-mediated antagonism of innate immune responses. Together, our findings identify TRIM34 as a proviral host factor that facilitates IAV replication by selectively impairing antiviral gene expression. Targeting TRIM34 may therefore represent a novel antiviral strategy against IAV and potentially other viral infections.

## Introduction

Influenza viruses (IVs) belong to the *Orthomyxoviridae* family, being enveloped viruses with a segmented, negative-sense, single-stranded RNA genome. IVs are classified into four types – A, B, C and D – with the type A (IAV) and B (IBV) producing annual epidemics in humans worldwide [1]. Despite extensive vaccination efforts, IVs remain a major global health challenge; the World Health Organization (WHO) estimates that IVs continues to cause approximately 1 billion infections each year, resulting in 3–5 million severe cases and nearly 500,000 deaths [2,3].

Innate immunity provides the first protective barrier against viral infections, with interferons (IFNs), pro-inflammatory cytokines and interferon-stimulated genes (ISGs)orchestrating a coordinated response that restricts replication at the earliest stages [4–6]. Recognition of pathogen-associated molecular patterns (PAMPs) by pattern recognition receptors (PRRs), such as retinoic acid-inducible gene I (RIG-I)-like receptors (RLRs) and Toll-like receptors (TLRs), initiates signaling cascades that culminate in the production of IFNs and pro-inflammatory cytokines [7–10]. Binding of IFNs to their cognate receptors activates the Janus kinase transducer and activator of transcription (JAK–STAT) pathway [4,11], driving the transcription of hundreds of ISGs. Collectively, these genes constitute the backbone of the antiviral state, modulating viral restriction and shaping immune balance, yet many of these ISGs remain poorly characterized in their broader biological functions [4,12–15].

Nucleocytoplasmic trafficking constitutes a central interface between host defense and viral replication [16,17]. Antiviral immunity relies on the nuclear import of transcription factors such as IFN regulatory factor 3 (IRF3), signal transducer and activator of transcription 1 (STAT1), and nuclear factor κB (NF-κB), which drive IFNs, pro-inflammatory cytokines, and ISGs expression [8,18,19], as well as on the nuclear export of antiviral mRNAs to ensure their cytoplasmic translation and effector activity [16,17]. In this context, the nucleoporin 93 (Nup93) has been demonstrated to regulate antiviral innate immunity by promoting the nuclear translocation of Interferon Regulatory Factor 3 (IRF3) [20]. After IRF3 phosphorylation, IRF3 must translocate to the nucleus to induce the transcription of interferons (IFNs) [21,22]. Additionally, the ribonucleic acid export 1 (RAE1) protein plays an important role in mRNA and RNA transport from the nucleus to the cytoplasm where protein translation occurs [23], thereby modulating the expression of host proteins. Disruption of either process not only weakens host immune integrity but also creates opportunities for viral subversion, as many viruses have evolved strategies to manipulate these pathways for their own replication and persistence [24]. Influenza A virus (IAV) exemplifies this dependency: its replication cycle critically relies on the nuclear export of viral ribonucleoproteins for assembly in the cytoplasm [25,26], while simultaneously subverting host nucleocytoplasmic trafficking [16,24]. Specifically, IAV interferes with the nuclear translocation of host transcription factors to dampen interferon responses [27,28], and further suppresses host gene expression by inhibiting nuclear export of host mRNAs, a process largely mediated by its non-structural protein 1 (NS1) [23,29–31]. This shared reliance on nucleocytoplasmic trafficking highlights it as a regulatory hub at the interface between host defense and viral pathogenesis [32].

Among ISGs, the tripartite motif-containing (TRIM) family – comprising at least 80 distinct proteins – has emerged as a multifunctional group with diverse roles in immunity, cell death, and cancer. TRIM proteins share a RING/B-box/coiled-coil (RBCC or tripartite motif) core, with an additional C-terminal domain [33]. The RING domain confers E3 ubiquitin ligase activity, the coiled-coil domain mediates both homo- and hetero-oligomerization, and the C-terminal domain facilitates substrate recognition, RNA binding, and protein–protein interactions [34]. This modular organization enables TRIM proteins to regulate a broad spectrum of cellular processes, including ubiquitination, autophagy, innate immune signaling, and direct viral restriction [33,35–37].

Within this family, we focused on TRIM34, an interferon-inducible protein [38]. Originally identified as RNF21, TRIM34 is characterized by a RING domain, B-box 1 and 2, a coiled-coil domain, and a C-terminal SPRY/B30.2 domain [39]. TRIM34 shares homology with TRIM5, TRIM6 and TRIM22 [40], and cooperates with TRIM5α to form higher-order complexes that recognize retroviral capsids and restrict early stages of HIV-1 and SIV infections [41–43]. Beyond retroviral restriction, TRIM34 regulates programmed cell death and tumor progression. During IAV infection, it promotes K63-linked poly-ubiquitination of Z-DNA binding protein 1 (ZBP1), recruiting the receptor-interacting serine/threonine-protein kinase 3 (RIPK3) and activating programmed cell death pathways, thus limiting lethal infection [44]. TRIM34 also localizes to mitochondria to trigger apoptosis via mitochondrial membrane potential loss and cytochrome c release [45]. In parallel, TRIM34 has emerged as a tumor suppressor. In non-small-cell lung cancer (NSCLC), it is induced by interferon-γ (IFN-γ) and promotes apoptosis through mechanistic target of rapamycin complex 1 (mTORC1)-dependent metabolic reprogramming [46,47]. In hepatocellular carcinoma (HCC), TRIM34 enhances ferroptosis and improves immunotherapy efficacy by regulating the up-frameshift protein 1 (UPF1)/glutathione peroxidase 4 (GPX4) axis [48]. Furthermore, TRIM34 inhibits triple-negative breast cancer (TNBC) progression by attenuating fatty acid synthesis via facilitating fatty acid synthase (FASN) ubiquitination [49]. However, its function as an ISG has not been characterized.

Here, we identified a novel interaction between TRIM34 and the nucleocytoplasmic transport machinery, focusing on its consequences for both innate immune signaling and IAV life cycle. To our knowledge, this represents a novel function for a TRIM protein, and such interactions have not been previously described for any member of the TRIM family. A deeper understanding of host proteins that modulate IAV replication, such as TRIM34, will be crucial for the development of novel antiviral drugs targeting these factors, representing an essential strategy to combat viral infections.

## Results

### TRIM34 expression is upregulated by IFN and IAV infection

**Fig 1:**
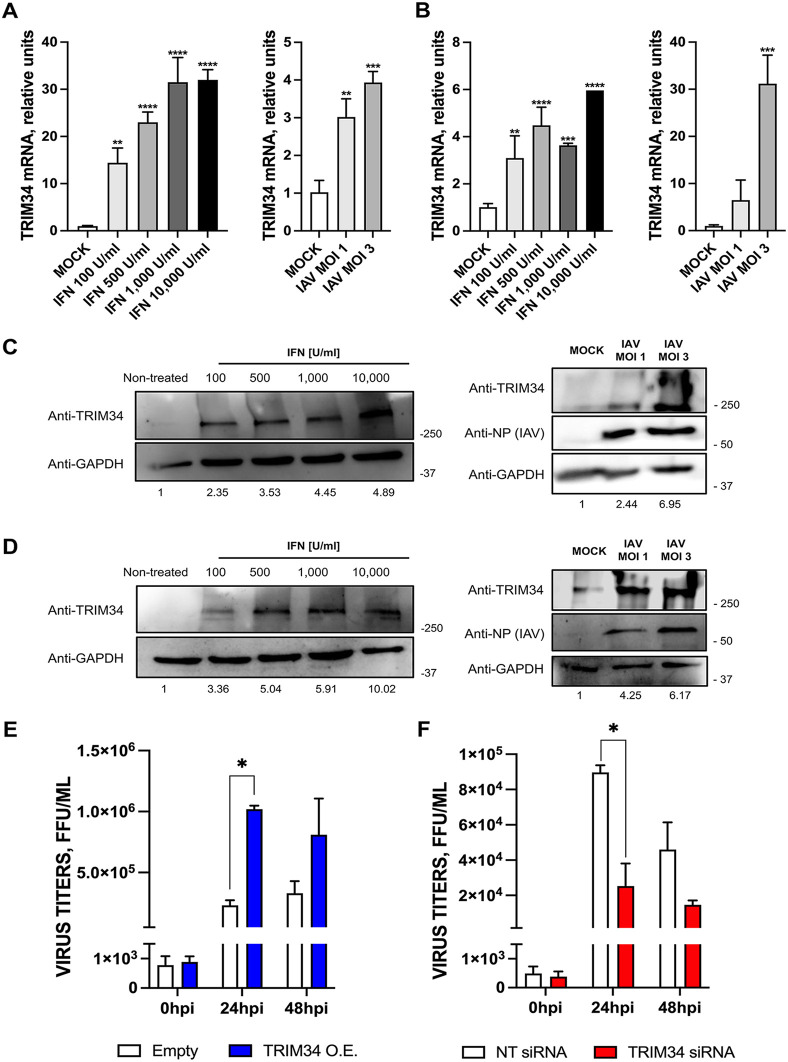
TRIM34 expression is upregulated by IFN treatment and IAV infection in human 293T and A549 cells. Human 293T **(A, C)** and human A549 **(B, D)** cells were treated with recombinant universal type I IFN or infected with IAV during 24h. TRIM34 gene expression was evaluated by RT-qPCR **(A, B)** and by Western blot, using an anti-TRIM34 specific antibody, and an anti-GAPDH specific antibody, used as a loading control **(C, D)**, and compared to the levels in mock-treated or mock-infected cells. Data in A and B represents means and SDs of results from triplicate wells. Three different experiments were performed, with similar results. **p < 0.01, ***p < 0.001, ****p < 0.0001; one-way ANOVA followed by Dunnett’s post-hoc test. Protein bands in C and D were quantified using the ImageJ software and normalized to the levels of GAPDH expression (numbers below the blots). **TRIM34 positively affects IAV replication *in vitro.*** **(E)** Human 293T cells were transfected with the pCAGGS plasmid expressing TRIM34-FLAG protein or a pCAGGS empty plasmid, as control. At 24h post-transfection (hpt), cells were mock-infected or infected with IAV (MOI 1). **(F)** Human 293T cells were transfected twice with a non-targeted (NT) control siRNA or TRIM34 siRNA at two consecutive days, 24 h apart. On day 3, cells were mock-infected or infected with IAV (MOI 1). **(E, F)** At 0-, 24-, and 48-hours post-infection (hpi), cell culture supernatants were collected and titrated by immunofocus assay in MDCK cells. Data represents means and SDs of results from triplicate wells. Three different experiments were performed, with similar results. *p < 0.05; Student’s t-test with Holm-Šídák correction*.*

TRIM34, also known as Ring Finger Protein 21 (RNF21) or Interferon-Responsive Finger Protein (IFP1) [39], has been previously described as an IFN-induced gene [45,46,50,51], whose expression is induced by influenza A virus (IAV) infection through type I interferon signaling [38]. In addition, other viruses that robustly trigger interferon responses may also induce TRIM34 indirectly [46]. To validate that TRIM34 functions as an ISG in our cell culture models, human embryonic kidney 293T and human epithelial lung adenocarcinoma-derived A549 cells were treated with IFN (100; 500; 1,000 and 10,000 U/mL) or infected with IAV (MOIs 1 and 3) during 24h. Then, the levels of TRIM34 were measured by RT-qPCR and Western blot, and compared to the mock-treated or mock-infected cells (Fig 1A-1D). In human embryonic kidney 293T cells, TRIM34 mRNA levels were increased by 14.4; 23; 31.5 and 31.9-fold after treating the cells with 100; 500; 1,000 and 10,000 U/mL of IFN; and, by 3, and 3.9-fold in cells infected with IAV at MOIs 1 and 3 (Fig 1A), which correlated with 2.35; 3.53; 4.45; and 4.89-fold increases in TRIM34 protein levels following treatment of 293T cells with 100; 500; 1,000 and 10,000 U/mL of IFN; and by by 2.44, and 6.95-fold increases upon IAV infections at MOIs 1 and 3, as observed by Western blot using an anti-TRIM34 antibody (Fig 1C). In human lung epithelial adenocarcinoma A549 cells, levels of TRIM34 mRNA were induced by 3.1; 4.5; 3.7 and 5.9-fold in cells treated with 100; 500; 1,000 and 10,000 U/mL of IFN; and by 6.4 and 31.2-fold, after IAV infections at MOIs 1 and 3 (Fig 1B), correlating with TRIM34 increases at the protein level of 3.36; 5.04; 5.91 and 10.02-fold in A549 cells treated with 100; 500; 1,000 and 10,000 U/mL of IFN (Fig 1D). Similarly, IAV infection at MOIs 1 and 3 led to a 4.25 and 6.17-fold increase (Fig 1D). All these data confirm that TRIM34 behaves as an ISG in our *in vitro* model. Furthermore, Western blot analysis was performed on extracts from human A549 and 293T cells using an anti-TRIM34 antibody to confirm endogenous protein expression under basal conditions (mock-treated cells) (Fig AA in S1 Appendix). The Western blot results showed high-molecular-weight bands (>250 kDa), likely corresponding to distinct oligomeric forms of TRIM34. Oligomerization is a common feature of cytoplasmic TRIM family members. Most TRIMs need to dimerize to be enzymatically active, and higher-order assemblies (protein bands with molecular weights >250 kDa) have also frequently been observed with some TRIM proteins, as is the case for TRIM34 [42].

### TRIM34 facilitates IAV replication *in vitro*

To investigate whether TRIM34 influences IAV replication, we used complementary overexpression and gene-silencing approaches. First, 293T cells were transfected with either the TRIM34-FLAG-encoding plasmid or the empty control plasmid, followed by infection with IAV (MOI 1). Transfection only modestly reduced infection from nearly 100% infected cells in non-transfected cells, as measured by immunofluorescence with an anti-IAV NP specific antibody, to ~80% in cells transfected with either the empty plasmid or the plasmid encoding TRIM34-FLAG (Fig AB in S1 Appendix). TRIM34 overexpression at 24 and 48 hpi was confirmed by Western blot analysis using anti-FLAG and anti-GAPDH (as loading control) antibodies (Fig AC in S1 Appendix). In addition, we found TRIM34 expression in mock-infected 293T cells, which was significantly increased, as expected, in cells transfected with the TRIM34-FLAG encoding plasmid (Fig AD in S1 Appendix). Then, extracellular infectious virus production was quantified at 0, 24 and 48 hpi. Viral titers were increased at 24 and 48 hpi, compared to 0 hpi, indicating an active viral replication (Fig 1E and 1F). IAV titers showed a 4.5 and 2.4-fold increase in TRIM34-overexpressing cells compared to the cells transfected with the empty vector control, at 24 and 48 hpi (Fig 1E), strongly suggesting that TRIM34 overexpression increases IAV titers. In addition, 293T cells were transfected with a TRIM34-specific siRNA or a non-targeting (NT) siRNA control prior to IAV infection (MOI 1). Transfection with the TRIM34-specific siRNA resulted in a reduction of TRIM34 mRNA levels by more than 80% (87% at 24 hpi and 81% at 48 hpi), as quantified by RT-qPCR (Fig AE in S1 Appendix); correlating with a reduction in the protein levels, as quantified by Western blot (Fig AF in S1 Appendix). Interestingly, a 3.5 and 3.1-fold reduction in viral titers was observed in TRIM34-knocked-down cells relative to control cells, transfected with the NT siRNA, at 24 and 48 hpi (Fig 1F), indicating that silencing the expression of TRIM34 decreases viral titers.

**Fig 2:**
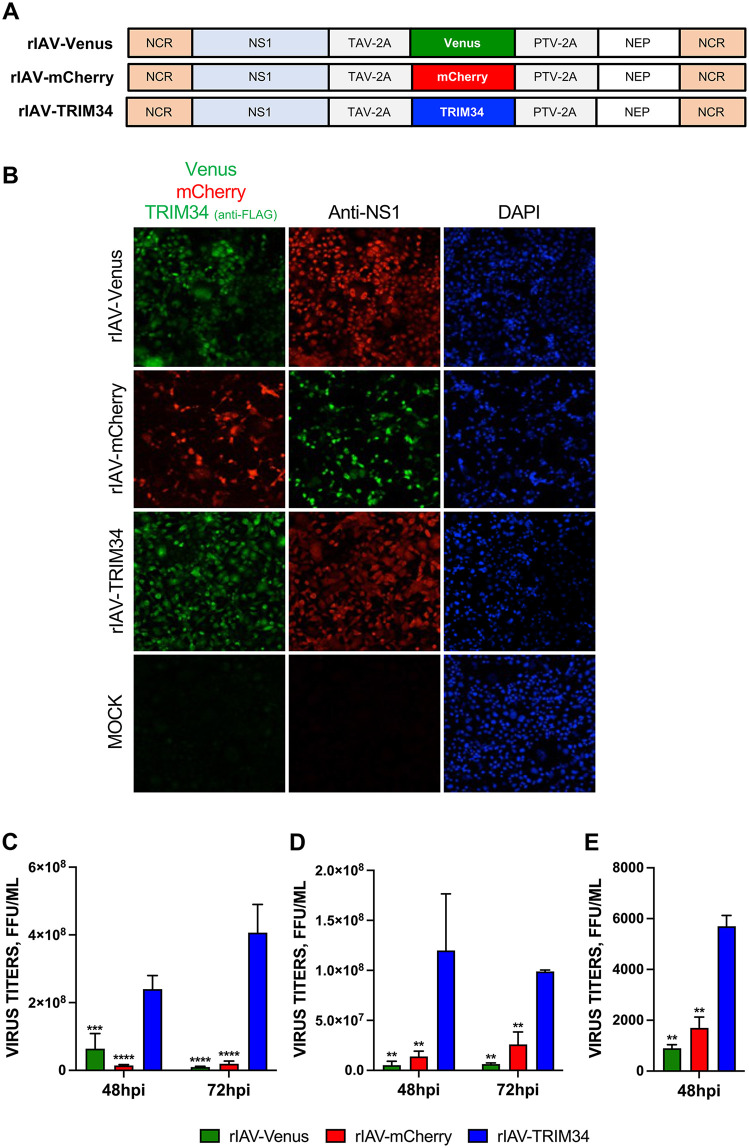
TRIM34 positively affects IAV replication *in vitro.* **(A)** Schematic representation of recombinant PR8 viruses expressing Venus, mCherry or TRIM34. A modified IAV-PR8 NS segment encoding NS1, either Venus (top), mCherry (middle) or TRIM34-FLAG (bottom), and NEP are indicated. Orange boxes at the beginning and end of each viral segment represent the viral 3′ and 5′ noncoding regions (NCR). Blue and white boxes indicate the viral NS1 and NEP genes, respectively. The thosea asigna virus (TAV) 2A autoproteolytic cleavage site used for the expression of NS1 and Venus, mCherry or TRIM34 and the porcine teschovirus (PTV) 2A autoproteolytic cleavage site used for the expression of Venus, mCherry or TRIM34 and NEP are indicated in gray. **(B, C, D, E)** Human A549 or MDCK cells were mock-infected or infected (MOI 0,1 and 1) with the recombinant viruses (rIAV-Venus, rIAV-mCherry or rIAV-TRIM34). **(B)** At 24hpi, MDCK cells were fixed, permeabilized and visualized for Venus and mCherry expression. Cells were stained with anti-FLAG (TRIM34) and anti-NS1 (infected-cells control) antibodies. DAPI was used for nuclear staining. **(C**-**D)** MDCK cells were infected with the viruses rIAV-Venus, rIAV-mCherry and rIAV-TRIM34 at MOIs 0.1 **(C)** or 1 **(D)** and viral titers were determined at 48 and 72hpi in MDCK cells. **(E)** A549 cells were infected with the viruses rIAV-Venus, rIAV-mCherry and rIAV-TRIM34 at MOI 1, and viral titers were determined at 48hpi in MDCK cells. Data represents means and SDs of results from triplicate wells. Three different experiments were performed, with similar results. **p < 0.01, ***p < 0.001, ****p < 0.0001 for comparisons between rIAV-TRIM34 with rIAV-Venus and rIAV-mCherry using one-way ANOVA followed by Dunnett’s post-hoc test.

To validate the finding showing that human TRIM34 positively modulates IAV production using an alternative experimental strategy, we generated recombinant IAVs expressing the human TRIM34-FLAG gene (rIAV-TRIM34) or the fluorescent proteins Venus and mCherry, as controls (rIAV-Venus, and rIAV-mCherry, respectively). To achieve this, the open reading frames (ORFs) of Venus, mCherry or TRIM34 were cloned into plasmids encoding a split segment of the IAV NS gene, as previously described for Venus and mCherry-expressing plasmids [19,52]. This design allowed for autoproteolytic processing of NS1, nuclear export protein (NEP) (also known as non-structural protein 2, or NS2) and Venus, mCherry or TRIM34-FLAG proteins via thosea asigna virus (TAV) 2A autoproteolytic cleavage site placed between NS1 and the respective inserted protein, and porcine teschovirus (PTV) 2A autoproteolytic cleavage site located between the inserted protein and NEP (Fig 2A). To verify expression of the heterologous proteins, MDCK cells were either mock-infected or infected (MOI 0.1) with rIAV-Venus, rIAV-mCherry, or rIAV-TRIM34. Expression of Venus and mCherry was assessed by fluorescence microscopy, while TRIM34 expression was evaluated by immunofluorescence with an anti-FLAG antibody. Additionally, immunofluorescence staining for the viral NS1 protein was performed to confirm similar levels of infection across conditions. The results demonstrated that the majority of NS1-positive cells also expressed Venus, mCherry, or TRIM34 (Fig 2B). Consistent with the overexpression data presented in Fig 1E, viral titers in MDCK and A549 cells infected with rIAV-TRIM34 were higher than those in cells infected with control viruses (rIAV-Venus and rIAV-mCherry). This increase was observed in MDCK cells infected at MOIs of 0.1 and 1 (Fig 2C and 2D) at 48 and 72 hpi, as well as in A549 cells infected at an MOI of 1 at 48 hpi (Fig 2E), further indicating that TRIM34 overexpression enhances IAV replication in cell cultures.

### TRIM34 protein interacts with the nucleocytoplasmic transport machinery

**Fig 3:**
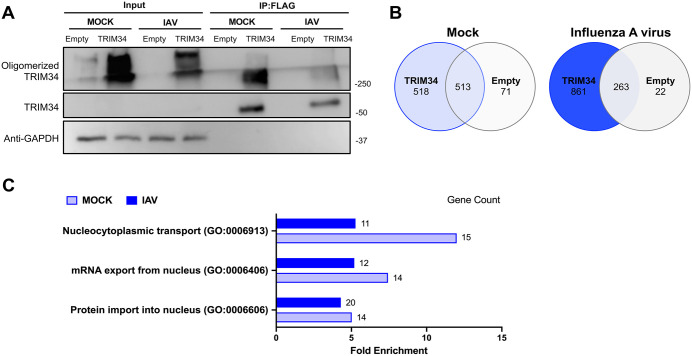
TRIM34 protein interacts with the nucleocytoplasmic transport machinery. **(A)** Human 293T cells were co-transfected with the pCAGGS plasmids encoding TRIM34-FLAG or the empty plasmid, as control. At 24hpt, cells were mock-infected or infected with IAV (MOI 1). Co-immunoprecipitation (co-IP) experiments using agarose beads conjugated to an anti-FLAG antibody, to pull down TRIM34 were performed. TRIM34 was detected by Western blotting using a TRIM34-specific antibody in the cellular lysates (input) and after the co-IP. An anti-GAPDH antibody served as a loading control for the input samples. Molecular weight markers (in kilodaltons) are indicated on the right. **(B)** Venn diagram of proteins identified by mass spectrometry in the co-IP pull-downs. **(C)** Qualitative proteomic analysis of specific proteins following TRIM34 overexpression. Gene Ontology (GO) biological processes were identified using the DAVID functional annotation tool and selected based on statistical significance (FDR < 0.05). The graphical representation includes GO terms containing the keywords ‘nucleus’, ‘nuclear’, or ‘nucleocytoplasmic’, with fold enrichment (>2) and gene counts (>3), shown for both mock and IAV conditions. The X-axis represents fold enrichment, with the number of genes indicated next to each bar.

To identify viral and cellular interaction partners of TRIM34, and to gain broader insight into the physiological functions in which TRIM34 might be involved, we performed co-immunoprecipitation (co-IP) experiments using agarose beads conjugated to an anti-FLAG antibody, across five experimental conditions: (1–3) mock-infected cells transfected with either an empty plasmid, a plasmid expressing TRIM34-FLAG, or a plasmid expressing GBP1-FLAG (GBP1 fused to a FLAG tag); and (4,5) IAV-infected cells transfected with the empty plasmid or the TRIM34-FLAG plasmid, respectively. First, the expression of TRIM34, using GAPDH as a loading control (Fig 3A), as well as the expression of GBP1 (Fig B in S1 Appendix), was confirmed by Western blotting, followed by LC-MS/MS analysis of the co-IP pull-downs. The prevalence of monomeric species in immunoprecipitated samples compared to the input (Fig 3A) is a common observation, as the lysis and solubilization steps required for co-immunoprecipitation can disrupt native oligomeric assemblies [53,54]. Qualitative proteomic analysis revealed 518 and 861 proteins, associated with TRIM34 in mock and IAV conditions, respectively, eliminating the proteins that were also identified in the cells transfected with the empty plasmid, which were considered as non-specific (Tables A and B in S2 Appendix; Fig 3B). Interestingly, among the TRIM34 interactome, we found the IAV NS1 protein, which is a well-characterized viral antagonist of innate immunity [55–59], and the host NS1-binding protein (NS1-BP) [60,61]. Although the interaction between IAV NS1 and TRIM34 was confirmed by co-immunoprecipitation and Western blot (Fig C in S1 Appendix), we focused this study on the host interactome, since TRIM34 expression is not restricted to IAV infection, and these cellular factors are present even in the absence of an IAV infection (Table B in S2 Appendix).

Then, the TRIM34-binding host proteins were grouped according to their Gene Ontology (GO) biological processes using the DAVID functional annotation tool, selecting significantly enriched terms based on an FDR-adjusted p-value<0.05 (Table C in S2 Appendix). Among the most significant biological processes under both mock and IAV conditions, we focused on those containing the keywords ‘nucleus’, ‘nuclear’, or ‘nucleocytoplasmic’, as they were both highly significant and biologically relevant. The processes common to both conditions included ‘nucleocytoplasmic transport’, ‘mRNA export from the nucleus’, and ‘protein import into the nucleus’ (Fig 3C) and involve proteins responsible for the transport of mRNAs and proteins between the nucleus and cytoplasm, such as nucleoporins and importins (Table 1). Importantly, these proteins were not detected after immunoprecipitation of GBP1-FLAG in mock- or IAV-infected cells overexpressing GBP1-FLAG, which was used as a FLAG-tag control (Tables D and E in S2 Appendix), strongly suggesting that TRIM34 binding to these proteins is not mediated by the FLAG tag. In addition, the biological processes ‘nucleocytoplasmic transport’, ‘mRNA export from the nucleus’, and ‘protein import into the nucleus’, were not statistically significant terms in the GBP1-FLAG interactome, as determined by analysis of their GO biological processes using the DAVID functional annotation tool, performed as described for TRIM34. In this study, we prioritized these GO terms because our data indicated that TRIM34 is a host factor that facilitates IAV replication (Figs 1E, 1F, 2C, 2D and 2E) and because the nucleocytoplasmic trafficking of proteins and RNAs through the nuclear pore complex plays a crucial role in regulating the cellular innate immune responses, affecting viral replication, as is the case with IAV infections [23,24,32,62].

**Table 1:** Common host proteins associated with nuclear transport processes found in the TRIM34-interactome, both in mock and IAV conditions. Proteins identified in both mock and IAV-infected samples that belong to the selected GO biological processes. The common processes include nucleocytoplasmic transport, mRNA export from the nucleus, and protein import into the nucleus.

Gene	Protein
**Nucleocytoplasmic transport (GO:0006913)**	
Q8N1F7	**Nuclear pore complex protein Nup93** (93 kDa nucleoporin) (Nucleoporin Nup93)
P35658	Nuclear pore complex protein Nup214 (214 kDa nucleoporin) (Nucleoporin Nup214) (Protein CAN)
P78406	**mRNA export factor RAE1** (Rae1 protein homolog) (mRNA-associated protein mrnp 41)
P37198	Nuclear pore glycoprotein p62 (62 kDa nucleoporin) (Nucleoporin Nup62)
Q99567	Nuclear pore complex protein Nup88 (88 kDa nucleoporin) (Nucleoporin Nup88)
Q5SRE5	Nucleoporin NUP188 (hNup188)
**mRNA export from nucleus (GO:0006406)**	
P52298	Nuclear cap-binding protein subunit 2 (20 kDa nuclear cap-binding protein) (Cell proliferation-inducing gene 55 protein) (NCBP 20 kDa subunit) (CBP20) (NCBP-interacting protein 1) (NIP1)
Q99567	Nuclear pore complex protein Nup88 (88 kDa nucleoporin) (Nucleoporin Nup88)
O14980	Exportin-1 (Exp1) (Chromosome region maintenance 1 protein homolog)
P35658	Nuclear pore complex protein Nup214 (214 kDa nucleoporin) (Nucleoporin Nup214) (Protein CAN)
P78406	**mRNA export factor RAE1** (Rae1 protein homolog) (mRNA-associated protein mrnp 41)
Q9Y5S9	RNA-binding protein 8A (Binder of OVCA1–1) (BOV-1) (RNA-binding motif protein 8A) (RNA-binding protein Y14) (Ribonucleoprotein RBM8A)
O00148	ATP-dependent RNA helicase DDX39A (EC 3.6.4.13) (DEAD box protein 39) (Nuclear RNA helicase URH49)
**Protein import into nucleus (GO:0006606)**	
Q13501	Sequestosome-1 (EBI3-associated protein of 60 kDa) (EBIAP) (p60) (Phosphotyrosine-independent ligand for the Lck SH2 domain of 62 kDa) (Ubiquitin-binding protein p62) (p62)
Q8N1F7	**Nuclear pore complex protein Nup93** (93 kDa nucleoporin) (Nucleoporin Nup93)
P35658	Nuclear pore complex protein Nup214 (214 kDa nucleoporin) (Nucleoporin Nup214) (Protein CAN)
Q8TEX9	Importin-4 (Imp4) (Importin-4b) (Imp4b) (Ran-binding protein 4) (RanBP4)
Q96P70	Importin-9 (Imp9) (Ran-binding protein 9) (RanBP9)
O95373	Importin-7 (Imp7) (Ran-binding protein 7) (RanBP7)
P37198	Nuclear pore glycoprotein p62 (62 kDa nucleoporin) (Nucleoporin Nup62)
Q99567	Nuclear pore complex protein Nup88 (88 kDa nucleoporin) (Nucleoporin Nup88)
O60684	Importin subunit alpha-7 (Karyopherin subunit alpha-6)
Q92973	Transportin-1 (Importin beta-2) (Karyopherin beta-2) (M9 region interaction protein) (MIP)
P52292	Importin subunit alpha-1 (Karyopherin subunit alpha-2) (RAG cohort protein 1) (SRP1-alpha)
Q5SRE5	Nucleoporin NUP188 (hNup188)

### TRIM34 interacts with Nup93

**Fig 4:**
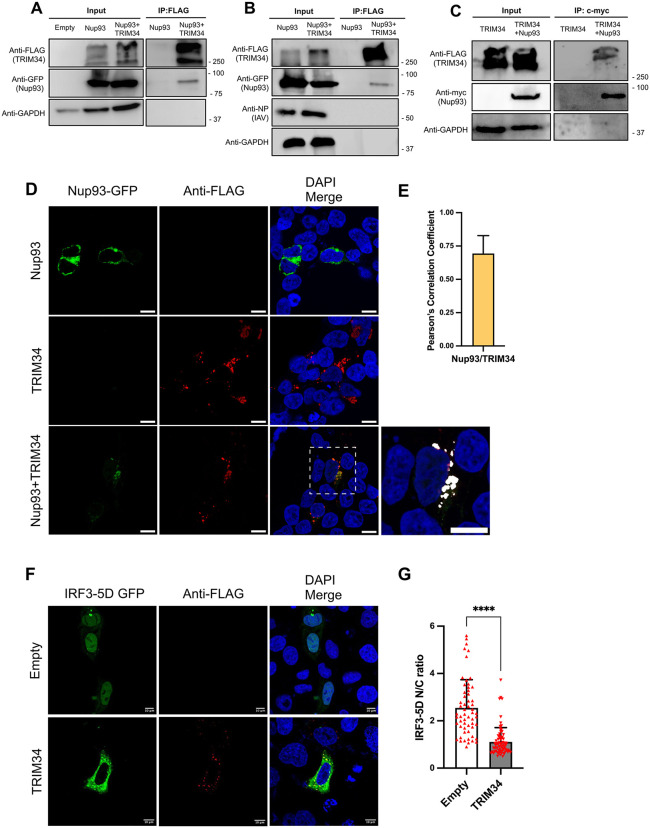
TRIM34 interacts with Nup93 and reduces IRF3-5D nuclear translocation. **(A, B)** Human 293T cells were co-transfected with the plasmid pEGFP-C1X expressing Nup93 fused to the EGFP protein alone or together with the pCAGGS plasmid encoding TRIM34-FLAG. At 24hpt, cells were **(A)** mock-infected o **(B)** infected with IAV (MOI 1). **(A, B)** Co-immunoprecipitation (Co-IP) experiment using an anti-FLAG antibody to pull down TRIM34 were performed. TRIM34 and Nup93 were detected by Western blotting using specific antibodies for the FLAG tag (to detect TRIM34) and the GFP tag (to detect Nup93) in the cellular lysates (input) and after the co-IP. An anti-GAPDH antibody served as a loading control for the input samples, and an anti-IAV NP antibody was used to confirm infection. Molecular weight markers (in kilodaltons) are indicated on the right. **(C)** Human 293T cells were co-transfected with the TRIM34-FLAG-encoding plasmid alone or together with the Nup93-myc-encoding plasmid. A co-immunoprecipitation (Co-IP) experiment using an anti-myc antibody to pull down Nup93 was performed. TRIM34 and Nup93 were detected by Western blotting using specific antibodies for the FLAG tag (to detect TRIM34) and the myc tag (to detect Nup93) in the cellular lysates (input) and after the co-IP. An anti-GAPDH antibody served as a loading control for the input samples. Molecular weight markers (in kilodaltons) are indicated on the right. **(D)** Human 293T cells were co-transfected with the plasmid pEGFP-C1X expressing Nup93 fused to the EGFP protein alone or together with the pCAGGS plasmid encoding TRIM34-FLAG. At 24hpt, cells were fixed with paraformaldehyde, TRIM34 was detected using an anti-FLAG antibody (red), Nup93 was identified through the GFP fluorescence signal encoded by the plasmid (green), and nuclei were stained with DAPI (blue). Confocal images show cells transfected with the plasmid expressing TRIM34-FLAG and Nup93-GFP separately, and cells co-transfected with both plasmids. Areas of co-localization of both proteins appear in yellow in the third picture and in white in the fourth picture (zoom). Scale bar, 10 μm. **(E)** Pearson’s correlation coefficient for Nup93 and TRIM34 fluorescent signals. Data represents means and SDs of 30 cells that were counted in three independent experiments. **(F)** Human HeLa cells were co-transfected with the pCAGGS plasmids encoding TRIM34-FLAG or the empty control, and the pCAGGS-IRF3-5D-GFP, expressing a constitutively active form of IRF3 (IRF3-5D) fused to GFP [63,64]. At 24hpt, cells were fixed with paraformaldehyde, TRIM34-FLAG was labeled with a specific antibody for the tag (in red) and nuclei were stained with DAPI (in blue). Scale bar, 10 μm. **(G)** Fluorescence intensity of IRF3-5D-GFP was quantified in the nucleus and cytoplasm to determine nuclear-to-cytoplasmic (N/C) ratios. **(F, G)** Data represents means and SDs of 58 (empty) and 76 (TRIM34) cells that were counted in three independent experiments. ****p < 0.0001; Student’s t-test with Holm-Šídák correction*.*

We hypothesized that TRIM34 modulates innate immune responses, and therefore, virus replication, through direct or indirect interactions with components of the nucleocytoplasmic transport machinery. Nup93 is essential for the assembly of the nuclear pore complex (NPC) and is localized within the inner ring of nucleoporins [20]. First, to confirm the mass spectrometry analysis showing that TRIM34 interacts with Nup93, 293T cells were co-transfected with plasmids encoding FLAG-tagged TRIM34 and Nup93 fused to the EGFP protein, followed by mock-infection (Fig 4A) or by infection with IAV (MOI 1) (Fig 4B). After confirming the overexpression of TRIM34 and Nup93, and IAV infection with an anti-IAV NP antibody by Western blot (Fig 4B), cell lysates were subjected to immunoprecipitation using anti-FLAG affinity columns. Notably, Nup93 co-immunoprecipitated with TRIM34 under both mock (Fig 4A) and IAV-infected (Fig 4B) conditions. In addition, to further validate the interaction, we generated a c-myc-tagged Nup93 plasmid, and we co-expressed Nup93-myc with TRIM34-FLAG by means of plasmid co-transfection. Then, a reciprocal co-immunoprecipitation using agarose beads conjugated to an anti-c-myc specific antibody in mock-treated cells was performed, showing that Nup93-myc and TRIM34-FLAG co-immunoprecipitated together (Fig 4C). These data indicate an interaction between the two proteins, which may be direct or mediated by other proteins, potentially components of the nuclear pore complex.

To examine potential intracellular colocalization of TRIM34 and Nup93, 293T cells were transfected with the corresponding plasmids, and their subcellular distribution was analyzed by immunofluorescence and confocal microscopy (Fig 4D and 4E). Partial colocalization of TRIM34 and Nup93 was observed, consistent with an interaction between the two proteins.

Given the role of Nup93 in promoting IRF3 nuclear translocation [20], we examined whether TRIM34 affects IRF3 nuclear localization. To this end, we analyzed the distribution of a constitutively active mutant of IRF3, known as IRF3-5D, by immunofluorescence and confocal microscopy (Fig 4F and 4G). IRF3-5D contains aspartic amino acid substitutions at five key phosphorylation sites, mimicking its activated state and allowing IRF3 nuclear entry in the absence of stimulation [63,64]. Quantification of fluorescence intensity in the nucleus and cytoplasm was used to determine the nuclear-to-cytoplasmic (N/C) ratio (Fig 4G), showing that overexpression of TRIM34 significantly impairs the nuclear translocation of IRF3-5D.

### TRIM34 interacts with RAE1

**Fig 5:**
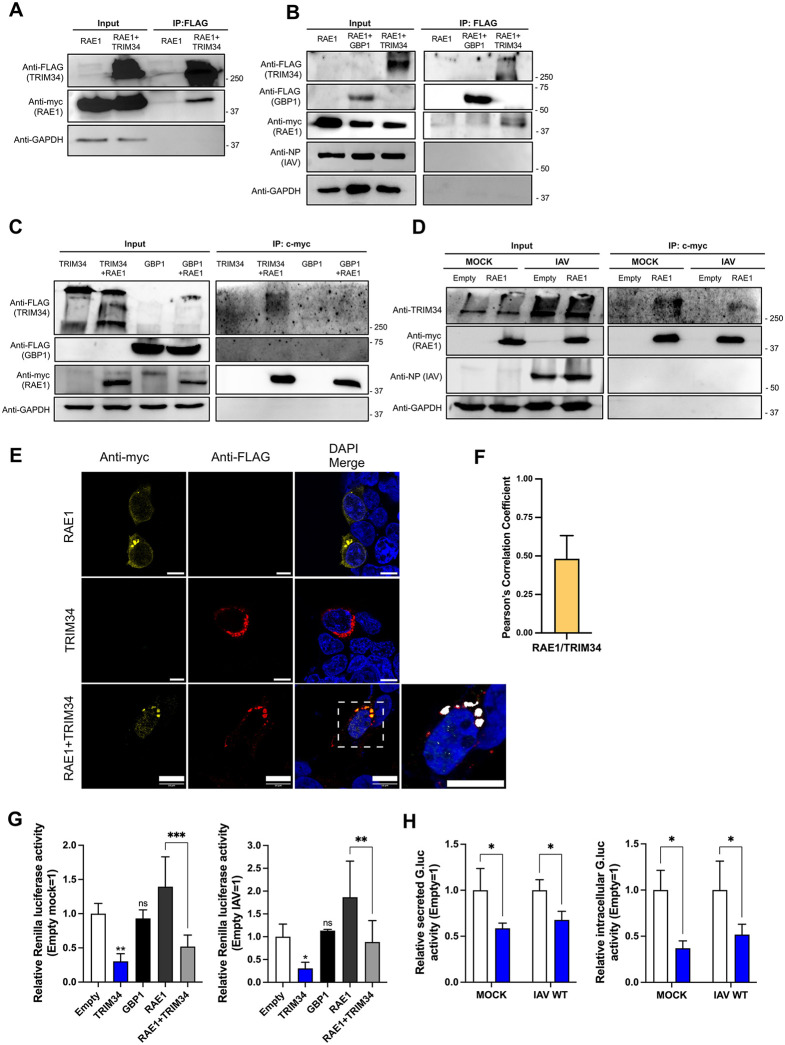
TRIM34 interacts with RAE1. Human 293T cells were co-transfected with the pcDNA3.1-RAE1-myc plasmid, expressing RAE1 fused to a c-myc tag, alone or in combination with the pCAGGS plasmid encoding TRIM34-FLAG or with the pCAGGS plasmid encoding GBP1-FLAG. At 24hpt, **(A)** cells were mock-infected or **(B)** infected with IAV (MOI 1). In addition, cells were co-transfected with the plasmids pcDNA3.1-RAE1-myc and pcDNA3.1-GBP1-FLAG to confirm that the interaction between TRIM34 and RAE1 is not due to the FLAG tag **(B)**. **(A, B)** Co-immunoprecipitation (Co-IP) experiments using an anti-FLAG antibody, to pull-down TRIM34 were performed. TRIM34, GBP1, and RAE1 were detected by Western blotting using specific antibodies for the FLAG tag (to detect TRIM34 and GBP1) and the c-myc tag (to detect RAE1) in the cellular lysates (input) and after the IP. An anti-GAPDH antibody served as a loading control for the input samples, and an anti-IAV NP antibody was used to confirm infection. Molecular weight markers (in kilodaltons) are indicated on the right. **(C, D)** Co-immunoprecipitation (Co-IP) experiments using an anti-c-myc antibody, to pull-down RAE1 were performed. 293T cells were transfected with TRIM34-FLAG-overexpressing plasmid together with the RAE-1-myc-overexpressing plasmid **(C)**, or with the RAE1-myc overexpressing plasmid alone **(D)**. TRIM34, GBP1, and RAE1 were detected by Western blotting using FLAG antibody (to detect TRIM34 and GBP1) and c-myc antibody (to detect RAE1) in input lysates and after IP **(C)**, or with anti-TRIM34 specific antibody replacing anti-FLAG antibody **(D)**. GAPDH was used as a loading control for the input samples, and an anti-IAV NP antibody was used to confirm infection. Molecular weight markers (in kilodaltons) are indicated on the right. **Partial co-localization of TRIM34 and RAE1 in the cytoplasm.** **(E)** At 24hpt, cells were fixed with paraformaldehyde, TRIM34-FLAG and RAE1-myc were labeled with specific antibodies for the tags (in red and yellow, respectively), and nuclei were stained with DAPI (in blue). Confocal images show cells transfected with the plasmid expressing TRIM34-FLAG and RAE1-myc separately, and cells co-transfected with both plasmids. Areas of co-localization of both proteins appear in orange in the third picture and in white in the fourth picture (zoom). Scale bar, 10 μm. **(F)** Pearson’s correlation coefficient for RAE1 and TRIM34 fluorescent signals. Data represents means and SDs of 30 cells that were counted in three independent experiments. **TRIM34 decreases host gene expression**. **(G)** Human 293T cells were co-transfected with a plasmid constitutively expressing Renilla luciferase (Rluc) and plasmids encoding TRIM34-FLAG, GBP1-FLAG or RAE1-myc alone, or the plasmids expressing TRIM34-FLAG and RAE1-myc together. At 24hpt, cells were mock-infected and infected with IAV (MOI 1). **(G)** At 24hpi, levels of Rluc were determined and normalized to the levels of mock-infected cells transfected with the empty plasmid or to the levels of IAV-infected cells transfected with the empty plasmid. **(H)** Alternatively, cells were co-transfected with a plasmid expressing Gaussia luciferase (Gluc) and the plasmid encoding TRIM34-FLAG or an empty plasmid, as control, and the cells were left mock-infected or infected with IAV. At 24 hpi, secreted and intracellular levels of Gluc were quantified and normalized to the levels in cells transfected with the empty plasmid. **(G, H)** Data represents means and SDs of results from triplicate wells. Three different experiments were performed, with similar results. **(G)** **p < 0.01, ***p < 0.001; one-way ANOVA followed by Dunnett’s post-hoc test. **(H)** *p < 0.05; Student’s t-test with Holm-Šídák correction.

Additionally, the interaction between TRIM34 and the mRNA export factor RAE1 was confirmed by co-immunoprecipitation using anti-FLAG agarose beads followed by Western blot analysis in 293T cells overexpressing both proteins (TRIM34-FLAG and RAE1-myc), under mock and IAV-infected conditions (Fig 5A and 5B), although the interaction may be direct or mediated by other proteins within the macromolecular complex. As an additional control to ensure that the interaction of TRIM34 with RAE1 is not mediated by the FLAG tag, GBP1-FLAG was included in the co-IP, showing that it does not bind to RAE1 (Fig 5B). In addition, to further validate the interaction, TRIM34-FLAG and RAE1-myc were co-expressed by plasmid co-transfection, and a reciprocal co-immunoprecipitation was performed using agarose beads conjugated to an anti-myc specific antibody, in mock-treated cells (Fig 5C). Alternatively, to analyze whether RAE1 interacts with endogenous TRIM34, RAE1-myc was overexpressed alone, and a co-immunoprecipitation was performed using agarose beads conjugated to an anti-myc specific antibody (Fig 5D). RAE1-myc interacted with both overexpressed TRIM34-FLAG (Fig 5C) and endogenous TRIM34 (Fig 5D). Remarkably, as a control, RAE1-myc did not interacted with GBP1-FLAG (Fig 5D). These results further support the interaction of TRIM34-FLAG with RAE1-myc, showing, in addition, that RAE1-myc interacts with endogenous TRIM34.

To investigate a potential intracellular colocalization of TRIM34 and RAE1, 293T cells were transfected with the plasmids, and the subcellular localization of both proteins was determined by immunofluorescence and confocal microscopy (Fig 5E and 5F). A partial co-localization of TRIM34 and RAE1 was observed (Fig 5E and 5F), supporting the interaction of TRIM34 with RAE1.

Since we discovered that TRIM34 interacts with proteins involved in the nuclear export of host mRNAs, such as nuclear cap-binding protein subunit 2 (CBP20), nucleoporin 88 (Nup88), exportin-1 (XPO1), nucleoporin 214 (Nup214), mRNA export factor RAE1, RNA-binding protein 8A and ATP-dependent RNA helicase DDX39A (Table 1), we conducted a bioluminescence assay to evaluate whether TRIM34 affects cellular gene expression. In this experiment, 293T cells were co-transfected with the plasmid expressing Renilla luciferase (Rluc), and either the empty vector or the vector expressing TRIM34, together or not with the plasmid expressing RAE1, and subsequently infected with IAV (MOI 1). In addition, as a control, the cells were transfected with the plasmid expressing GBP1-FLAG. Then, the expression of Rluc was quantified in a luminometer. The Rluc luminescent signal was lower in cells overexpressing TRIM34, both in mock and IAV-infected conditions, compared to the control cells transfected with the empty plasmid (Fig 5G), by contrast, this effect was not observed in control cells overexpressing GBP1-FLAG (Fig 5G), strongly suggesting that TRIM34 overexpression leads to an inhibition of gene expression, most likely by affecting the transport of mRNAs to the cytoplasm. Furthermore, overexpression of RAE1 led to an increase in the luminescent signal (Fig 5G), most likely due to enhanced nucleocytoplasmic transport, as previously described [65–67]. However, when TRIM34 and RAE1 were co-transfected, we obtained an intermediate luminescent value (Fig 5G), suggesting that TRIM34 counteracts the increase in gene expression induced by RAE1. To confirm these findings and to demonstrate that the effect of TRIM34 is not restricted to Rluc, cells were co-transfected with a plasmid expressing Gaussia luciferase (Gluc), together with an empty vector or a plasmid encoding TRIM34-FLAG (Fig 5H). Secreted and intracellular Gluc were reduced by TRIM34 overexpression in both mock- and IAV-infected cells (Fig 5H), indicating that TRIM34 mediates suppression of gene expression. Furthermore, cells were co-transfected with a GFP reporter plasmid together with either an empty vector, a plasmid encoding TRIM34-FLAG or a plasmid expressing GBP1-FLAG. Western blot analysis revealed a reduction in GFP protein levels in cells overexpressing TRIM34, compared with cells transfected with the empty vector or those overexpressing GBP1 (Fig D in S1 Appendix). Taken together, these data (Figs 5G, 5H and Fig D in S1 Appendix) indicate that TRIM34 overexpression suppresses gene expression using multiple reporter systems.

### TRIM34 impairs innate immune responses induced by IFN and IAV infection

**Fig 6:**
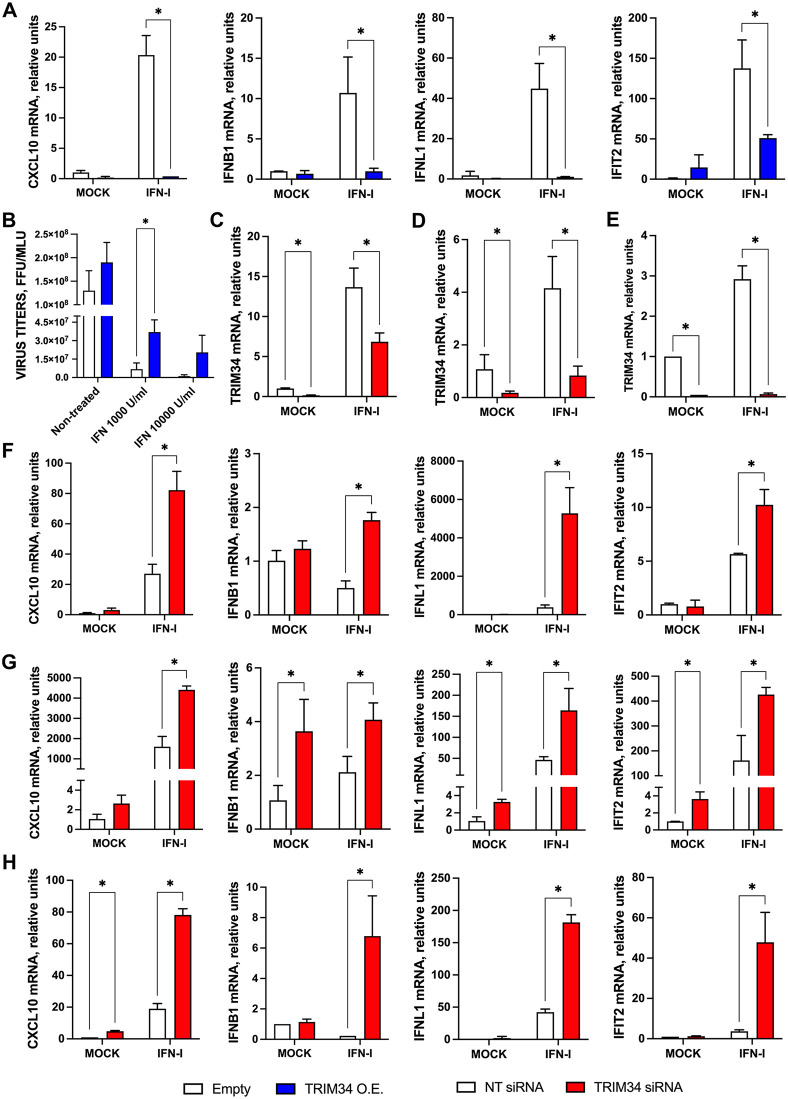
TRIM34 impairs innate immune responses induced by IFN. **(A, B)** Human 293T cells were transfected with the plasmid pCAGGs-TRIM34-FLAG or the empty control plasmid. **(A)** At 24h post-transfection, cells were non-treated or treated with type I IFN. At 24h post-IFN-treatment, CXCL10, IFNB1, IFNL1 and IFIT2 expression was measured by RT-qPCR and mRNA levels were expressed as fold-change (increases) in comparison to mock-treated cells transfected with the empty plasmid, used as control. **(B)** At 24h post-IFN-treatment, cells were infected with rVSV-GFP (MOI 0.1) and viral titers were measured by lysis plaque assay at 24hpi. Data represents means and SDs of results from triplicate wells. Three different experiments were performed, with similar results. *p < 0.05 for comparisons between empty and TRIM34 overexpressed cells using Student’s t-test with Holm-Šídák correction*.* Human 293T cells **(C, F)**, human lung adenocarcinoma-derived cells A549 cells (D, G), or the non-tumorigenic human bronchial epithelial BEAS-2B **(E, H)** were transfected twice with NT control siRNA or TRIM34 siRNA, 24h apart, for two consecutive days. On day 3, cells were treated with IFN for an additional 24h. **(C, D, E)** TRIM34, and **(F, G,** **H)** CXCL10, IFNB1, IFNL1 and IFIT2 expression was measured by RT-qPCR and mRNA levels were expressed as fold-change (increases) in comparison to mock-treated cells, transfected with the NT siRNA, used as control. **(A-H)** Data represents means and SDs of results from triplicate wells. Three different experiments were performed, with similar results. *p < 0.05 for comparisons between control and TRIM34 knocked-down cells using Student’s t-test with Holm-Šídák correction*.*

To assess the effect of TRIM34 on the interferon (IFN)-induced antiviral state, transcript levels of the pro-inflammatory cytokine CXCL10, type I IFN (IFNB1), type III IFN (IFNL1), and the ISG IFIT2 were measured by RT-qPCR in 293T cells transfected with either empty vector or the TRIM34-expressing plasmid, and subsequently treated with IFN. Overexpression of TRIM34 was confirmed by Western blot using a FLAG-specific antibody (Fig EA in S1 Appendix). As expected, IFN treatment induced the expression of CXCL10, IFNB1, IFNL1, and IFIT2 genes. Significantly, the expression of these genes was induced to a lower extent in TRIM34-overexpressing cells compared to control cells (Fig 6A), indicating that TRIM34 negatively affects the induction of innate immune responses. Alternatively, to confirm these results, IFN-treated 293T cells were infected with rVSV-GFP (MOI 0.1), a virus known to be highly sensitive to previous antiviral responses induced in the cells [68,69]. IFN treatment at two different concentrations significantly reduced rVSV-GFP titers by 18.9 and 114.7-fold compared to untreated cells (Fig 6B), as expected, due to the induction of an IFN-dependent antiviral state in the cells. Notably, overexpression of TRIM34 increased rVSV-GFP titers compared to cells transfected with the empty vector. The lower-dose IFN produced a significant 5.4-fold rise, while the higher-dose led to an 18-fold increase that was not statistically significant but remained biologically meaningful (Fig 6B), supporting that TRIM34 counteracts the induction of innate immune responses. Furthermore, to confirm these findings by a complementary approach, TRIM34 expression was knocked-down in 293T cells using a specific siRNA, while a NT siRNA served as a control. Efficient knock-down of TRIM34 was verified by RT-qPCR (Fig 6C). Upon IFN treatment, the expression of CXCL10, IFNB1, IFNL1, and IFIT2 was markedly higher in TRIM34-silenced 293T cells than in control cells (Fig 6F), supporting the notion that TRIM34 negatively affects the induction of IFN responses. In addition, to confirm these results, we silenced TRIM34 expression in two lung-derived cell lines, as IAV mainly infects the respiratory system: the epithelial lung adenocarcinoma-derived cells A549 cells (Fig 6D and 6G) and the non-tumorigenic human bronchial epithelial BEAS-2B cells (Fig 6E and 6H). Again, efficient TRIM34-knock-down was confirmed by RT-qPCR (Fig 6D and 6E).Upon IFN treatment, the expression of CXCL10, IFNB1, IFNL1, and IFIT2 was markedly higher in TRIM34-silenced A549 (Fig 6G) and BEAS-2B cells (Fig 6H) than in control cells. Together, these experiments support a conserved inhibitory role for TRIM34 in IFN-induced cells across distinct tissue-derived cellular contexts.

**Fig 7:**
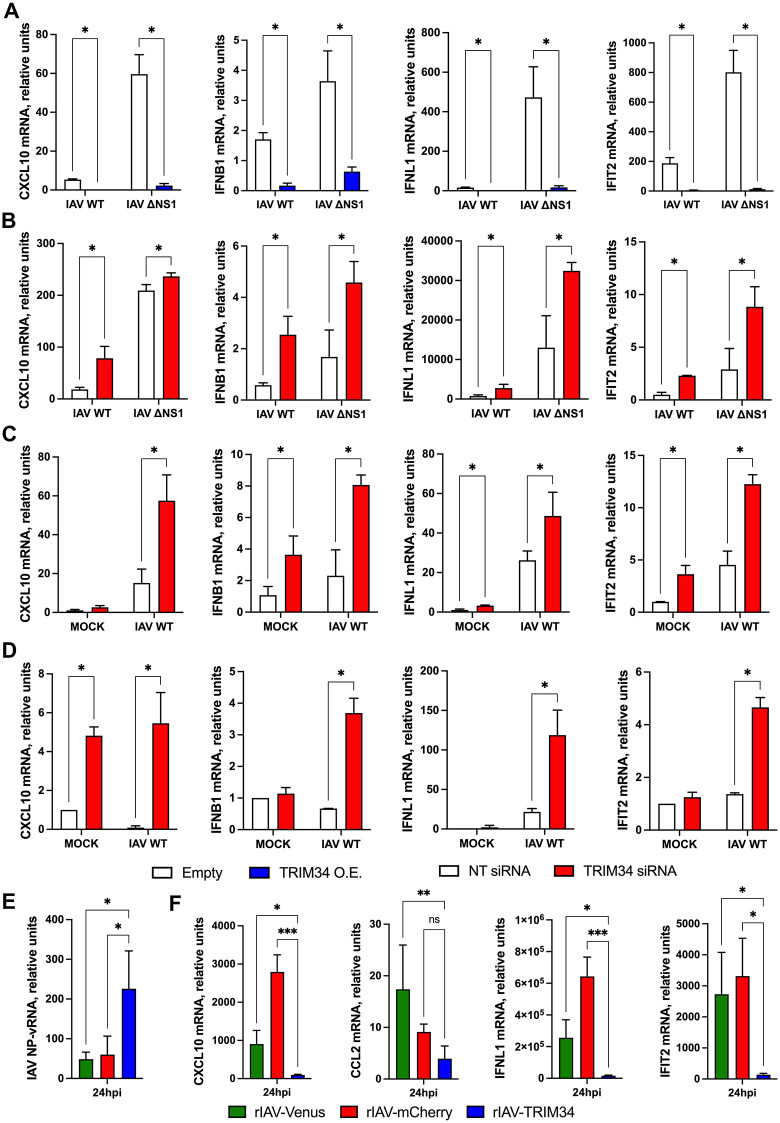
TRIM34 impairs innate immune responses induced by IAV. **(A)** Human 293T cells were transfected with the pCAGGS plasmids encoding TRIM34-FLAG or the empty plasmid, as control. At 24h post-transfection, cells were mock-infected or infected with IAV or IAV-ΔNS1. At 24hpi, the levels of CXCL10, IFNB1, IFNL1, and IFIT2 mRNAs were evaluated by RT-qPCR, and expressed as fold-change (increases) in comparison to mock-infected cells, transfected with the empty plasmid, used as control. **(B)** Human 293T cells were transfected twice with the NT control siRNA or TRIM34 siRNA every 24h for two consecutive days. On day 3, cells were infected with IAV or IAV-ΔNS1 (MOI 1) for an additional 24h. The levels of CXCL10, IFNB1, IFNL1 and IFIT2 were measured by RT-qPCR and mRNA levels were expressed as fold- change (increases) in comparison to mock-infected cells transfected with the NT siRNA, used as controls. Human A549 **(C)** or BEAS-2B **(D)** cells were transfected twice with the NT control siRNA or TRIM34 siRNA every 24h for two consecutive days. On day 3, cells were infected with IAV for an additional 24h. **(B, C, D)** The levels of CXCL10, IFNB1, IFNL1 and IFIT2 were measured by RT-qPCR and mRNA levels were expressed as fold change (increases) in comparison to mock-infected cells transfected with the NT siRNA, used as controls. **(A-D)** Data represents means and SDs of results from triplicate wells. Three different experiments were performed, with similar results. *p < 0.05 for comparisons between empty and TRIM34 overexpressed cells, or NT siRNA and TRIM34 knocked-down cells, using Student’s t-test with Holm-Šídák correction. **(E, F)** A549 cells were infected with the viruses rIAV-Venus, rIAV-mCherry and rIAV-TRIM34 at MOI 1, and the levels of **(E)** IAV NP-vRNA, and **(F)** CXCL10, CCL2, IFNL1 and IFIT2 mRNAs were determined at 24hpi. **(E, F)** Data represents means and SDs of results from triplicate wells. Three different experiments were performed, with similar results. *p < 0.05, **p < 0.01, ***p < 0.001 for comparisons between rIAV-TRIM34 with rIAV-Venus and rIAV-mCherry using one-way ANOVA followed by Dunnett’s post-hoc test.

To explore whether TRIM34 also modulates innate immune responses following viral infection, 293T cells were transfected with the TRIM34-expressing plasmid, and overexpression was confirmed by Western blot using a FLAG-specific antibody (Fig EB in S1 Appendix), or the cells were treated with siRNAs to knock-down TRIM34 expression. In both cases, cells were infected with either IAV or a mutant virus lacking the NS1 gene and expressing mCherry (IAV-ΔNS1-mCherry). Then, the mRNA levels of CXCL10, IFNB1, IFNL1, and IFIT2 were measured at 24 hpi. As expected, both IAV and IAV-ΔNS1-mCherry induced the expression of these genes; however, the induction was stronger with the mutant virus, consistent with the role of NS1 in attenuating the activation of innate immune response genes [55–59]. Interestingly, CXCL1, IFNB1, IFNL1, and IFIT2 gene induction was significantly attenuated in TRIM34-overexpressing cells compared to the cells transfected with the empty plasmid (Fig 7A), whereas the opposite effect was observed in TRIM34-silenced cells, which exhibited enhanced expression of CXCL10, IFNB1, IFNL1, and IFIT2 genes, compared to control cells transfected with the NT siRNA (Fig 7B). Alternatively, to analyze the effect of TRIM34 on lung-derived cells, the expression of TRIM34 was knocked-down on the lung adenocarcinoma-derived cells A549 cells (Fig 7C), and the non-tumorigenic human bronchial epithelial BEAS-2B (Fig 7D), using siRNAs, as above. TRIM34 mRNA levels were reduced by 12-fold in A549 cells (Fig EC in S1 Appendix) and 14-fold in BEAS-2B cells (Fig ED in S1 Appendix) following gene silencing, compared to the control cells transfected with the NT siRNA. Notably, after IAV infection, the expression of CXCL10, IFNB1, IFNL1, and IFIT2 was increased in the A549 and BEAS-2B TRIM34-silenced cells, compared to the control cells (Fig 7C and 7D), as observed in the 293T cells (Fig 7B), indicating that TRIM34 counteracts the innate immune responses in different cellular systems, and in non-tumorigenic cell lines.

Consistent with these observations, we quantified mRNA levels of CXCL10, CCL2, IFNL1, and IFIT2 at 24 hpi in A549 cells infected with recombinant viruses (rIAV-Venus, rIAV-mCherry, and rIAV-TRIM34) at an MOI of 1. Notably, rIAV-TRIM34 infection resulted in a markedly attenuated induction of these genes compared with cells infected with the control viruses (rIAV-Venus and rIAV-mCherry) (Fig 7F). However, IAV replication, assessed by NP-vRNA levels, was higher in rIAV-TRIM34–infected cells than in those infected with the control viruses (Fig 7E), as expected (Fig 2C, 2D, and 2E), indicating that the reduction in the expression of CXCL10, CCL2, IFNL1, and IFIT2 after rIAV-TRIM34 infection, is not due to lower levels of viral replication. Altogether, these data indicated that TRIM34 counteracts the induction of innate immune responses.

### Effect of TRIM34 on viral replication and the induction of innate immune responses in mice

**Fig 8:**
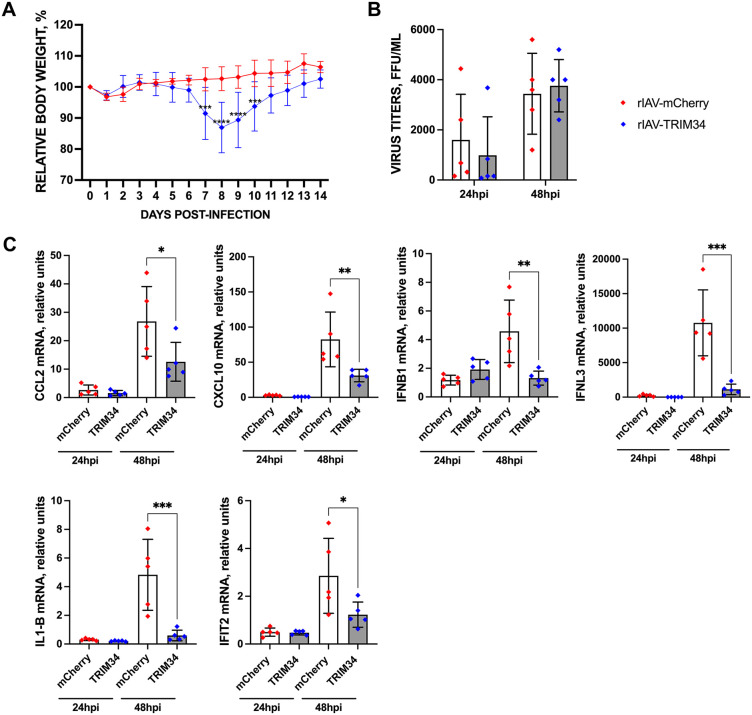
Effect of TRIM34 expression on the induction of innate immune responses *in vivo.* **(A, B, and** **C)** Mice (n = 5/group) were infected with the recombinant viruses rIAV-mCherry and rIAV-TRIM34 (2,000 FFU/mice). **(A)** Weight loss was monitored daily for 14 days post-infection. Differences in body weights between rIAV-mCherry and rIAV-TRIM34-infected mice were analyzed by two-way ANOVA; ***p < 0.001, ***p < 0.0001. **(B)** At 24, and 48 hpi, viral titers in mouse lungs were determined, and **(C)** CCL2, CXCL10, IFNB1, IFNL3, IL-1b and IFIT2 mRNA expression was evaluated in mouse lungs by RT-qPCR. Each dot corresponds to one rIAV-infected mouse. Increases in mRNA levels were expressed as fold-changes in comparison to mock-infected mice, used as controls. *p < 0.05, **p < 0.01, ***p < 0.001; two-way ANOVA followed by Tukey’s post-hoc test.

To analyze whether human TRIM34 affects virus pathogenicity and counteracts the innate immune responses *in vivo*, as observed in vitro (Figs 6 and 7), groups of C57BL/6 mice (n = 5 per group) were infected intranasally with 2,000 FFU/mouse of either rIAV-TRIM34 or rIAV-mCherry (control), and weight loss and survival were monitored daily for 14 days (Fig 8A). No mice died as a result of the infection. However, the rIAV-TRIM34 was slightly more virulent than the control rIAV-mCherry, as observed by a significant higher weight loss in the mice infected with the former virus, at days 7-, 8-, 9-, and 10-days post-infection (Fig 8A). In addition, groups of mice (n = 5 per group) were infected intranasally with the recombinant viruses (2,000 FFU/mouse), and viral titers in the lungs were measured at 24 and 48 hpi. No significant differences in viral titers were detected between groups at either time point (Fig 8B). We then evaluated the expression levels of CCL2, CXCL10, IFNB1, IFNL3, IL-1b, and IFIT2 at 24 and 48 hpi (Fig 8C). At 24 hpi, no significant upregulation of these genes was observed in infected mice compared to mock-infected control mice. However, at 48 hpi, a pronounced increase in CCL2, CXCL10, IFNB1, IFNL3, IL-1b, and IFIT2 expression was evident in the lungs of infected animals. Notably, the induction of these antiviral and pro-inflammatory genes was significantly lower in mice infected with rIAV-TRIM34 compared to those infected with rIAV-mCherry, used as control (Fig 8C). Given that lung viral titers at 24 and 48 hpi in the mice infected with the viruses expressing mCherry, as control, and with the TRIM34-expressing virus, show no differences (Fig 8A), it is very unlikely that the effects on the expression of innate immune genes observed with the TRIM34-overexpressing virus are due to differences in the replication dynamics of both viruses. However, the virus expressing the TRIM34 protein was slightly more virulent than the control virus expressing the mCherry protein, likely due to an inefficient innate immune response induced by the TRIM34-expressing virus at early times post-infection (Fig 8C). Thus, these *in vivo* findings align with the *in vitro* data presented in Figs 6 and 7, further supporting the conclusion that TRIM34 dampens innate immune responses both in cultured cells and in the animal model.

### TRIM34 induces nuclear retention of immune-regulated gene transcripts

**Fig 9:**
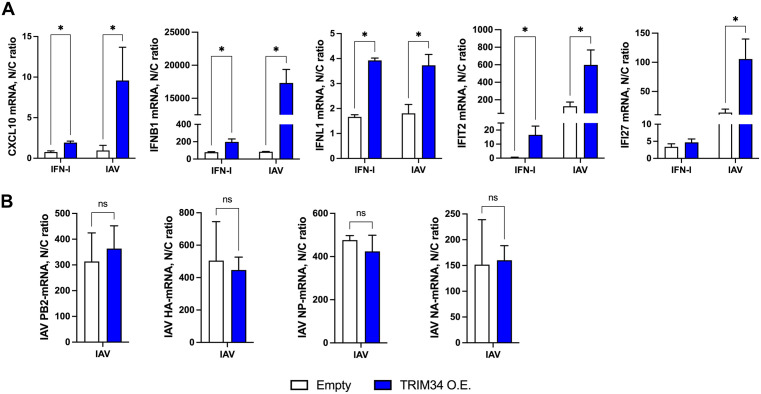
TRIM34 limits nuclear export of immune-regulated mRNAs. Human 293T cells were transfected with the pCAGGS plasmids encoding TRIM34-FLAG or the empty plasmid, as control. At 24h post-transfection, cells were treated with IFN or infected with IAV. At 24hpi, cells were fractionated into cytoplasm and nuclear RNA fractions. **(A)** Nuclear/Cytoplasmic (N/C) ratios of CXCL10, IFNB1, IFNL1, IFIT2, IFI27 and **(B)** IAV PB2, HA, NP and NA-mRNAs were quantified in TRIM34-overexpressed cells and in empty-plasmid transfected cells. **(A, B)** Data represents means and SDs of results from triplicate wells. Three different experiments were performed, with similar results. *p < 0.05 or ns (not-statistically significant*)* for comparisons between empty and TRIM34 overexpressed cells using Student’s t-test with Holm-Šídák correction.

Given that TRIM34 interacts with factors responsible for mRNA export from the nucleus to the cytoplasm (Figs 3 and 5A-5D, and Table 1), likely leading to host shut off (Figs 5G, 5H and Fig D in S1 Appendix), and based on our findings showing that innate immune responses are attenuated upon TRIM34 overexpression and increased upon its silencing – observed across various cell lines (293T, BEAS-2B and A549) (Figs 6 and 7) and even in mice (Fig 8) – we performed nuclear-cytoplasmic fractionation in 293T cells to assess the distribution of innate immune gene transcripts. This approach was also motivated by the fact that many viruses employ nuclear export blockade of immune-related transcripts as an immune evasion strategy, potentially disrupting bidirectional nucleocytoplasmic trafficking. Nuclear retention of host mRNAs impairs antiviral and immune protein synthesis, weakening host defenses [16,17,24,62,70–73]. After validating the nucleus/cytoplasm fractionation procedure by measuring GAPDH mRNA and MALAT1 long non-coding RNA levels at both fractions (Fig F in S1 Appendix), we found that TRIM34 overexpression significantly increased the nuclear-to-cytoplasmic (N/C) ratios of CXCL10, IFNB1, IFNL1, IFIT2, and IFI27 mRNAs following IFN treatment and IAV infection (Fig 9A), while IAV mRNAs from PB2, HA, NP and NA segment showed no significant changes (Fig 9B). These results suggest that TRIM34 may mediate crosstalk between nucleocytoplasmic transport and innate immune signaling, leading to nuclear sequestration of immune-related transcripts, without affecting the transport of viral mRNAs.

### E3 ubiquitin ligase activity of TRIM34 is not required for its effects on cellular gene expression

**Fig 10:**
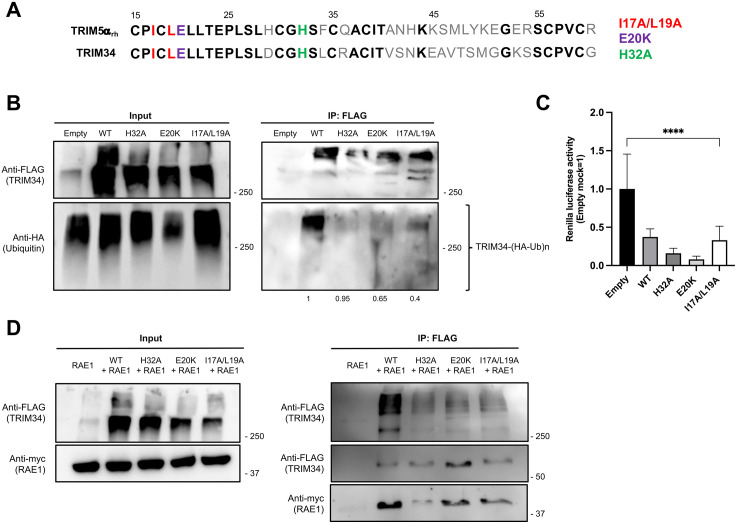
E3 ubiquitin ligase activity is not essential for TRIM34 effect on suppressing host gene expression or its interaction with RAE1. **(A)** Comparison of the first 60 amino acids in the RING domain between rhesus macaque TRIM5α and human TRIM34 proteins, highlighting sequence homology (in bold). The three selected mutants were: I17A/L19A (red), E20K (purple) and H32A (green). **Self-ubiquitylation of TRIM34.** **(B)** Human 293T cells were co-transfected with a plasmid expressing ubiquitin-HA together with the pCAGGS plasmids encoding the FLAG-tagged TRIM34 variants (H32A, E20K and I17L/L19A), the wild-type TRIM34 protein, or the empty plasmid, as control. An immunoprecipitation experiment using an anti-FLAG antibody, to pull down TRIM34 was performed. Ubiquitylated TRIM34 was analyzed by Western blotting with the anti-HA antibody and the expression of WT, H32A, E20K and I17L/L19A TRIM34 variants was detected using the anti-FLAG antibody in the cellular lysates (input) and after the co-IP. Molecular weight markers (in kilodaltons) are indicated on the right. Ubiquitylated TRIM34 protein bands were quantified using the ImageJ software and normalized to the levels of anti-FLAG (numbers below the blots). **(C)** Human 293T cells were co-transfected with a plasmid constitutively expressing Rluc and plasmids encoding the empty control, TRIM34 WT or TRIM34 mutants (H32A, E20K, I17L/L19A). At 24hpt, levels of Rluc were determined and normalized to the levels of cells transfected with the empty plasmid. Data represents means and SDs of results from triplicate wells. Three different experiments were performed, with similar results. ****p < 0.0001 for comparisons between empty and TRIM34 (WT or mutants) overexpressed cells using one-way ANOVA followed by Dunnett’s post-hoc test. **(D)** Human 293T cells were co-transfected with the pCAGGS plasmids encoding FLAG-tagged mutants (H32A, E20K and I17L/L19A) or wild-type TRIM34 (WT), and the pcDNA3.1-RAE1-myc, expressing RAE1 fused to a c-myc tag. Co-IP experiments using an anti-FLAG antibody, to pull-down TRIM34 were performed. The expression of TRIM34 (WT or mutants) was analyzed by Western blotting using the anti-FLAG antibody and RAE1 was detected with the c-myc tag antibody in the cellular lysates (input) and after the co-IP. Molecular weight markers (in kilodaltons) are indicated on the right.

As it was previously reported that TRIM34, as a member of the TRIM family, exhibits E3 ubiquitin ligase activity mediated by its RING domain [44,48], we investigated whether this activity is essential for its effects on cellular gene expression and/or its interaction with the nucleocytoplasmic transport machinery. To this end, we selected three mutants with specific amino acid substitutions (I17A/L19A, E20K and H32A) based on sequence homology with TRIM5α and previously published data showing that these substitutions abolish TRIM5α E3 ubiquitin ligase activity [74] (Fig 10A). We confirmed the loss of self-ubiquitylation capacity of the TRIM34 mutants by co-transfecting 293T cells with plasmids encoding wild-type (WT) or TRIM34 mutants, along with an HA-tagged ubiquitin plasmid, followed by co-immunoprecipitation with the anti-FLAG antibody, to pull-down the TRIM34 variants (Fig 10B). Interestingly, co-transfection of WT or mutant TRIM34 plasmids with a plasmid expressing the Rluc reporter revealed that the TRIM34 mutants defective in E3 ubiquitin ligase activity retain their ability to downregulate host gene expression in our cell model (Fig 10C), suggesting that the TRIM34 E3 ubiquitin ligase activity is not essential for the TRIM34-mediated inhibition of host gene expression. However, even though the three variants produced similar results, potential structural alterations or residual E3 ubiquitin ligase activity cannot be entirely excluded as confounding factors. Additionally, to assess whether the interaction with RAE1 was preserved in these TRIM34 variants, we co-transfected cells with either WT or TRIM34 mutant-overexpressing plasmids together with the RAE1-overexpressing plasmid. Co-immunoprecipitation using anti-FLAG agarose followed by Western blot analysis revealed that, although the amount of oligomerized TRIM34 recovered was reduced for the TRIM34 variants compared to TRIM34-WT, the levels of monomeric TRIM34 were not decreased. Moreover, the mutants’ proteins retained their capacity to interact with RAE1 (Fig 10D). The fact that the oligomerized TRIM34 form levels are reduced for the mutants defective in the E3 ubiquitin activity could be due to the fact that these amino acid changes likely affect oligomerization, as it has been shown for the same amino acid variants using the homologous rhesus macaque TRIM5 [74]. These findings strongly suggest that the E3 ubiquitin ligase activity of TRIM34 is not essential for either TRIM34 effect on suppressing host gene expression or its interaction with RAE1 (Fig 10C and 10D).

## Discussion

This work provides evidence for a novel function of the IFN-induced gene TRIM34 in modulating the innate immune response during IAV infection. Our cell culture-based overexpression and silencing experiments indicate a positive correlation between TRIM34 expression and IAV titers, revealing that TRIM34 plays a role in promoting viral replication (Figs 1 and 2). Moreover, we demonstrated that TRIM34 impairs innate immune responses mediated by IAV infection, not only *in vitro (*Fig 7) but also in mice (Fig 8), as well as in cells directly treated with IFN (Fig 6). Furthermore, we observed increased VSV titers in IFN-treated cells after TRIM34 overexpression (Fig 6). Given these data, the mechanism by which TRIM34 facilitates viral replication likely involves the negative modulation of innate immune responses, as supported by previous experiments using VSV as a sentinel virus, taking advantage of its dependence on the preexisting antiviral state to assess innate immune competence [68,69]. Remarkably, in addition to IAV infections, TRIM34 modulates innate immune responses after IFN treatment, strongly suggesting that this effect of TRIM34 is broad, and potentially applies to other viruses which induce the expression of host IFNs, and not only to IAV infections.

Notably, although innate immune responses are essential for controlling viral infections, excessive inflammation following infection can be detrimental to the host [12,13,75–78]. Consistent with this idea, our group previously reported that several ISGs – including IFI6, IFI27, IFI44, and IFI44L – exert feedback regulatory functions [19,52,79–81]. In this regard, TRIM34 could also act to limit exacerbated innate immune responses to viral challenges.

Our results indicate, for the first time, that TRIM34 interacts with the nucleocytoplasmic transport machinery, even in the absence of IAV infection (Fig 3 and Table 1), affecting the transport of mRNAs and proteins between the nucleus and the cytoplasm, providing a likely explanation for the effect of TRIM34 on negatively modulating the innate immune responses. In addition, our data strongly suggest that the interaction with these proteins involved in nucleocytoplasmic transport is not dependent on TRIM34 E3 ubiquitin ligase activity (Fig 10). In this context, numerous studies have established a connection between the induction of antiviral immune responses and the nucleocytoplasmic transport. This trafficking is essential for the proper activation, signaling, and execution of antiviral defenses – especially type I and type III interferon signaling – and viruses have evolved various strategies to manipulate it for immune evasion and replication enhancement [16,17]. In general, many RNA viruses encode viral proteins that interfere with the nuclear pore complex (NPC) components or with nucleocytoplasmic transport receptors, to improve viral replication. For instance, picornaviruses such as poliovirus and human rhinovirus (HRV) degrade nucleoporins (Nup62, Nup98, Nup153) [24,82–86], whereas encephalomyocarditis virus (EMCV) interferes with Ran-GTPase system and nucleoporin phosphorylation [24,70,87,88]. Similarly, flavivirus proteins such as Dengue virus NS3 and NS5 interact with importins and nucleoporins [89–91], vesicular stomatitis virus (VSV) matrix (M) protein inhibits the nuclear export of host mRNAs by disrupting the nuclear pore complex RAE1-Nup98 [92–94], Ebola virus (EBOV) VP24 viral protein blocks the nuclear translocation of tyrosine-phosphorylated STAT1 to inhibit host IFNs signaling [24,95,96], SARS-CoV ORF6 protein blocks STAT1 nuclear translocation [97,98], and SARS-CoV-2 ORF6 protein hijacks the NPC components RAE1 and Nup98 [99,100].

In particular, nucleocytoplasmic transport is essential for IAV infections due to the unique characteristics of its life cycle. Unlike most RNA viruses, IAV replicates and transcribes its genome within the host cell nucleus, requiring precise regulation of both the import and export of proteins and mRNAs between the nucleus and the cytoplasm. For this reason, IAV has evolved multiple strategies to manipulate this transport to ensure efficient viral replication while suppressing innate immunity [101–106]. IAV NP protein and polymerase-subunit PB2 directly engage α-importins to facilitate nuclear import of vRNPs [107], while nuclear export of vRNA is mediated via the CRM1/exportin-1 pathway [108], driven by the specific interaction between the viral NEP protein and CRM1 [26]. IAV NS1 protein inhibits host mRNA nuclear export by downregulating Nup98 expression and forming an inhibitory complex with mRNA export factors such as RAE1, NXT1, NXF1, and E1B-AP5 (or HNRNPUL1), thereby facilitating viral mRNA translation while suppressing host gene expression [23,29]. Accordingly, our results demonstrate that TRIM34 interacts with RAE1 (both in mock and IAV-infected cells) – confirmed by mass spectrometry, co-immunoprecipitation and Western blot, and partial co-localization by confocal microscopy (Fig 5, Table 1 and Table B in S2 Appendix) – with NXF1 (only in IAV-infected c ells), and with E1B-AP5 (or HNRNPUL1, both in mock and IAV-infected cells) (Table B in S2 Appendix), and negatively modulates host gene expression, even in the absence of IAV infection (Fig 5).

Additionally, the nuclear import of transcription factors such as IRF3, NF-κB, and STAT1/2 – essential for the activation of IFNs and ISGs – is regulated by importin α/β complexes and disruption of this nuclear import impairs antiviral gene expression [16,109]. Our data indicate that TRIM34 interacts with Nup93, demonstrated by mass spectrometry, co-immunoprecipitation and Western blot, and partial co-localization by confocal microscopy (Fig 4). Moreover, we have also confirmed that TRIM34 suppress the nuclear translocation of IRF3-5D and this restriction may be mediated by the interaction between TRIM34 and Nup93, as it has been shown that IRF3 nuclear translocation during RLR signaling was impaired in Nup93-deficient cells, exhibiting decreased expression of IFNs and inflammatory cytokines [20]. Therefore, the effect of TRIM34 on inhibiting the nuclear translocation of IRF3 (Fig 4F and 4G) is likely functionally relevant, as the transcription factor IRF3 contributes to the expression of IFNs and pro-inflammatory cytokines.

Notably, mass spectrometry identified other TRIM34-binding proteins, including importins (Table B in S2 Appendix), which may regulate the nucleocytoplasmic trafficking of these and other transcription factors, highlighting the need for further investigation.

Furthermore, as previously mentioned, inhibition of nuclear export of immune-related transcripts constitutes a prevalent viral immune evasion mechanism [32,110,111], notably exploited by IAV. Chemical inhibition experiments [112] demonstrate that selectively blocking nuclear export of host mRNAs – especially those involved in immunity, such as IFNs and ISGs – can dampen the antiviral response, causing these antiviral mRNAs to accumulate in the nucleus instead of reaching the cytoplasm for translation, without broadly affecting all mRNAs, underscoring the specificity and importance of this pathway for immune control during infection. By hindering the export of immune-related mRNAs, IAV not only evades detection and restriction by the innate immune system but also enhances replication and cytotoxicity in host cells [23,29].

Related to this, we have demonstrated that TRIM34 overexpression potentiates nuclear retention of host mRNAs encoding key innate immune effectors, including IFNs, pro-inflammatory cytokines, and ISGs (Fig 9), likely affecting their protein translation at the cytoplasm, dampening the host antiviral responses after IAV infection (Fig 7). However, TRIM34 could also influence the trafficking of other host RNAs, an aspect that requires deeper investigations. In IAV infection, inhibition of nuclear export is a highly robust virulence mechanism primarily mediated by the NS1 protein that directly interferes with nuclear export by binding components of the mRNA export pathway [23,29]. Interestingly, TRIM34 impairs the nucleocytoplasmic transport of innate immune mRNAs even in the absence of IAV infection, in IFN-stimulated cells (Figs 6 and 9). We further demonstrate that TRIM34 interacts with proteins involved in mRNA´s and protein´s nucleocytoplasmic transport in mock-infected cells (Fig 3 and Table B in S2 Appendix), revealing that this mechanism is IAV NS1-independent. Nevertheless, future work will be necessary to determine whether the nuclear retention of transcripts is selective for particular mRNAs or represents a global mechanism of regulation.

Consistent with our finding that TRIM34 selectively impairs host mRNA export without affecting IAV transcripts (Fig 9), the viral NS1, together with NS1-BP, recruits NXF1 to viral mRNAs, ensuring their nuclear export and promoting viral protein synthesis even with host mRNA export inhibition [61,112,113]. Although the effect of TRIM34 on nucleocytoplasmic trafficking is independent of IAV NS1 protein, as we also observe a TRIM34-mediated suppression on innate immune responses in cells treated with IFN (Fig 6) and in cells infected with an IAV lacking the NS1 protein (Fig 7), our mass spectrometry experiments revealed that TRIM34 interacts with both IAV NS1 and NS1-BP (Table B in S2 Appendix). Furthermore, the interaction between TRIM34 and IAV NS1 has been confirmed by co-IP (Fig C in S1 Appendix). Therefore, future studies may investigate these interactions to determine whether TRIM34 may serve as an intermediary between viral NS1 and host proteins involved in nucleocytoplasmic transport.

In summary, all these molecular mechanisms could explain how TRIM34, by interacting with proteins involved in the nucleocytoplasmic transport of host proteins and mRNAs, facilitates the impairment of innate immune responses, thereby enhancing IAV replication. Overall, our results suggest that modulating TRIM34 activity could provide a promising strategy for antiviral therapy against IAV and potentially other viral infections. Nevertheless, the risk of exacerbated inflammation must be carefully addressed in future studies.

## Materials and methods

### Ethics statement

Procedures involving animals were approved by the CSIC ethics committee for animal experimentation and by the Division of Animal Protection of the regional government of Madrid in compliance with the National and European Union legislation (PROEX343.0/23).

### Cells

Madin-Darby Canine Kidney (MDCK) epithelial (American Type Culture collection, ATCC CCL-34), human embryonic kidney 293T (ATCC CRL-11268), human lung epithelial carcinoma A549 (ATCC CCL-185), and African green monkey kidney epithelial Vero E6 (ATCC CRL-1586) cells were kindly provided by Prof. Luis Enjuanes (National Center for Biotechnology, CNB-CSIC, Spain). HeLa M WT (ATCC CCL-2) cells were kindly provided by Thomas Michiels (Université Catholique de Louvain, Brussels, Belgium) [114]. Non-tumorigenic human bronchial epithelial cell line BEAS-2B (ATCC CRL-9690), was obtained from Carlos López-Larrea (Health Research Institute of the Principality of Asturias (ISPA), Spain). All the cells, except the BEAS-2B cells, were grown at 37°C in air enriched with 5% CO_2_ using Dulbecco’s modiﬁed Eagle’s medium (DMEM, Gibco) supplemented with 10% fetal bovine serum (Gibco) and 50 μg/mL gentamicin (Gibco). The BEAS-2B cells were grown in LHC-9 medium (Thermo Fisher Scientific) supplemented with 50 μg/mL gentamicin (Gibco).

### Viruses and virus titrations

Virus stocks of IAV A/Puerto Rico/8/1934 H1N1 (PR8) (wild-type (WT) and recombinant viruses encoding Venus, mCherry or TRIM34-FLAG), as well as of the recombinant IAV lacking the NS1 protein and expressing the fluorescent protein mCherry (IAV-ΔNS1-mCherry), previously reported [115], were grown in MDCK cells [116] under BSL2 conditions. All IAV infections were performed in the presence of DMEM, containing 1 μg/mL of tosylsulfonyl phenylalanyl chloromethyl ketone (TPCK)-treated trypsin (Sigma), and 0.3% bovine serum albumin (BSA, Gibco). IAV was titrated by immunofocus assay (fluorescent focus units, FFU/mL), in confluent MDCK cells seeded in 96-well plates, as previously described [117]. The recombinant Vesicular Stomatitis Virus, Indiana strain, encoding the green ﬂuorescent protein (GFP) (rVSV-GFP) [118] was grown in Vero E6 cells, under BSL2 conditions. rVSV-GFP was titrated by plaque assay (plaque forming units, PFU/mL) in conﬂuent Vero E6 cells seeded in 24-well plates, as previously described [68].

### Plasmids

A polymerase II expression pCAGGS plasmid encoding TRIM34 (GenBank accession number NM_021616.6) fused to an N-terminal FLAG epitope tag (pCAGGS-TRIM34-FLAG) was generated. To this end, a DNA fragment encoding the open reading frame (ORF) from TRIM34 fused to the FLAG tag sequence, flanked by the unique restriction enzymes *SacI/SmaI* was synthesized (IDT DNA). Then, this fragment was cut with the restriction enzymes *SacI* and *SmaI* (New England Biolabs), and the fragment was cloned in the pCAGGS plasmid cut with the same enzymes. A pCAGGS plasmid encoding the IAV NS1 protein (A/PuertoRico/8/1934, PR8 strain) N-terminally tagged with HA was generated as previously described [119]. pEGFP-C1X expressing Nup93 (GenBank accession number BC034346.1) fused to the EGFP protein (pEGFP-C1X-Nup93) was a gift from Dr. Lei Lu (Addgene plasmid #87336) [120]. The plasmid pcDNA3.1 expressing Nup93 fused to a c-myc tag (pcDNA3.1-Nup93-myc) was generated. To this end, a DNA fragment encoding the open reading frame (ORF) from Nup93 fused to the c-myc tag sequence, flanked by the unique restriction enzymes *NheI/BamHI* was synthesized (IDT DNA). Then, this fragment was cut with the restriction enzymes *NheI* and *BamHI* (New England Biolabs), and the fragment was cloned in the pcDNA3.1 plasmid cut with the same enzymes. pcDNA3.1 expressing RAE1 (GenBank accession number NM_003610.4) fused to a c-myc tag (pcDNA3.1-RAE1-myc) was chemically synthesized by GenScript. The plasmid pcDNA3.1 expressing GBP1 (GenBank accession number NM_002053.3) fused to a FLAG tag (pcDNA3.1-GBP1-FLAG) was chemically synthesized by GenScript. The plasmid pEGFPC1 encoding a constitutive active form of IRF3 (IRF3-5D) fused to GFP (pEGFPC1-IRF3-5D-GFP) was kindly provided by Prof. Luis Martínez-Sobrido (Texas Biomedical Research Institute, San Antonio, TX, US). A pRK5 plasmid expressing ubiquitin fused to an HA tag was a gift from Dr. Ted Dawson (Addgene plasmid #17608) [121]. The pCAGGS plasmids expressing the TRIM34 mutants (I17L/L19A, E20K and H32A) were generated by cloning overlapping PCRs comprising the TRIM34 ORFs into the pCAGGS plasmid. These overlapping PCRs were generated using as template the pCAGGS-TRIM34-FLAG plasmid and primers encoding the sequences for amino acid changes. The pRL plasmid expressing Renilla luciferase (Rluc) under the control of a constitutive promoter (pRL-SV40) was obtained from Promega.

A plasmid encoding two different 2A autoproteolytic cleavage sites, from thosea asigna virus (TAV), and porcine teschovirus (PTV), and the IAV non-structural 1 (NS1) and nuclear export protein (NEP) genes expressed as independent ORFs (pDZ-NS-2x*BsmBI* plasmid), was previously generated [19]. This plasmid has the following elements: 5′-non-coding region (NCR)/NS1/link (GTRG)/TAV-2A/GSG-*BsmBI/BglII/BsmBI*-GSG/PTV-2A/NEP/3′- NCR. For generating recombinant IAV-Venus, IAV-mCherry and IAV-TRIM34, the plasmids pDZ-NSsplit-2x*BsmBI*-2A-Venus, pDZ-NSsplit-2x*BsmBI*-2A-mCherry and pDZ-NSsplit-2x*BsmBI*-2A-TRIM34 were used. The plasmids pDZ-NSsplit-2x*BsmB*I-2A-Venus and pDZ-NSsplit-2x*BsmBI*-2A-mCherry were previously generated [19]. To generate the plasmid pDZ-NSsplit-2x*BsmBI*-2A-TRIM34, the DNA fragment encoding TRIM34 C-terminally fused to a FLAG tag was ampliﬁed by PCR using speciﬁc primers ﬂanked by *BsmBI* restriction sites, using the plasmid pCAGGS-TRIM34-FLAG as template, the PCR product was digested with the restriction enzyme *BsmBI* (New England Biolabs) and was cloned in the pDZ-NSsplit2x*BsmBI*-2A plasmid cut with the same enzyme.

### Virus rescue

Co-cultures (1:1) of 293T and MDCK cells were co-transfected with 1 µg of the seven ambisense WT pHW plasmids encoding viral proteins PB2, PB1, PA, HA, NP, NA and M, from PR8 strain, plus the pDZ-NSsplit-2x*BsmBI*-2A-Venus, pDZ-NSsplit-2x*BsmBI*-2A-mCherry or pDZ-NSsplit-2x*BsmBI*-2A-TRIM34 plasmids, in 6-well plates, using lipofectamine 3000 (ThermoFisher Scientiﬁc). At 16 hours post-transfection (hpt), medium was replaced with DMEM containing antibiotics, 0.3% BSA, and 1 µg/mL of TPCK-treated trypsin (Sigma). At 48h, cell culture supernatants were collected and used to infect fresh conﬂuent monolayers of MDCK cells. At 3 days post-infection (dpi), recombinant viruses expressing mCherry, Venus or TRIM34-FLAG (rIAV-mCherry, rIAV-Venus, and rIAV-TRIM34) were plaque puriﬁed and a stock was generated by infecting MDCK cells at low multiplicity of infection (MOI 0.001). Viral stocks were titrated by immunofocus assay in MDCK cells, as previously described [117].

### Silencing of TRIM34

Human 293T, A549 or BEAS-2B cells (48-well plate format) were transfected with a “silencer select” small interfering RNA (siRNA) specific for human TRIM34 (556070, ThermoFisher Scientific), or with the non-targeting (NT) negative control siRNA (AM4635, ThermoFisher Scientific), twice, 24h apart. Both siRNAs were transfected at a final concentration of 20 nM, using lipofectamine RNAiMax (ThermoFisher Scientific), according to the manufacturer’s instructions.

### IFN response assays

To evaluate the effect of TRIM34 on IFN responses, human 293T, A549 or BEAS-2B cells (24-well plate format) were transfected with an siRNA specific for TRIM34, or the NT control siRNA, twice, 24h apart. Alternatively, 293T cells were transfected with the pCAGGS plasmid expressing TRIM34-FLAG protein or the pCAGGs empty control plasmid. Then, 293T, A549 or BEAS-2B cells were infected with IAV (MOI 1) for 24 and 48h. IAV titers were determined as described above. Furthermore, human 293T, A549 or BEAS-2B cells were treated with different concentrations of recombinant human type I IFN (11200–2, PBL Assay Science) during 24h. Total RNA was extracted, and RT-qPCRs were performed, as described below. In addition, at 24h after IFN treatment, 293T cells were infected with the rVSV-GFP [118] for 24h and viral titers in cell culture supernatants were determined in Vero E6 cells, as previously described [68,79,80].

### Quantitative PCR assays

Total RNAs were extracted using the total RNA extraction kit (Omega Biotek). Retrotranscriptase (RT) reactions were performed using the High-Capacity cDNA transcription kit (ThermoFisher Scientiﬁc) at 37°C for 2h, using random primers, and total RNA as template. qPCRs were performed using Taqman gene expression assays (Applied Biosystems) speciﬁc for human TRIM34 (Hs00363708_m1), human C-X-C motif chemokine ligand 10 (CXCL10) (Hs00171042_m1), human Interferon beta 1 (IFNB1) (Hs02621180_s1), human Interferon lambda 1 (IFNL1) (Hs00601677_g1), human Interferon-induced protein with tetratricopeptide repeats 2 (IFIT2) (Hs00533665_m1), and human Glyceraldehyde 3-phosphate dehydrogenase (GAPDH) (Hs02786624_g1) genes. Quantiﬁcation was achieved using the threshold cycle (2^-ΔΔCT^) methodology [122] and normalized with GAPDH expression levels.

To study IAV replication and/or transcription, RT-qPCR was performed following an adapted protocol [123–125] in which purified RNA was reverse-transcribed using the High-Capacity cDNA Reverse-Transcription kit (ThermoFisher Scientific) with tagged primers specific for IAV nucleoprotein (NP)-vRNA or for viral mRNAs encoding for polymerase basic protein 2 (PB2), hemagglutinin (HA), neuraminidase (NA) and NP (Table F in S2 Appendix) [123–125]. qPCR was performed with the SYBR Green PCR Master Mix (ThermoFisher Scientific) and gene-specific primers for the NP-vRNA or the viral mRNAs (Table G in S2 Appendix) [123–125]. The transcripts amplified were also quantified using the threshold cycle (2^− ΔΔCT^) method.

### Co-immunoprecipitation assays

Human 293T cells (100 mm-plate format) were transiently co-transfected with the plasmids pCAGGS-TRIM34-FLAG or pcDNA3.1-GBP1-FLAG, together with pcDNA3.1-RAE1-myc, pEGFP-C1X-Nup93 or pcDNA3.1-Nup93-myc; or with the plasmid pcDNA3.1-RAE1-myc alone, and/or the empty pCAGGS plasmid using lipofectamine 3000 (ThermoFisher Scientiﬁc), for 24 h. The total amount of transfected DNA plasmid was always the same, as the empty pCAGGS plasmid was co-transfected when needed. Then, 24h later, cells were left mock-infected or infected with IAV (MOI 1) for an additional 24h, and cells were lysed in co-immunoprecipitation (co-IP) buffer (NaCl 250 mM; EDTA 1 mM; 50 mM TrisHCl, pH 7.5; NP-40 0.5%) containing protease (ThermoFisher Scientific) and phosphatase (Merck) inhibitors, sonicated for 5 min in an ultrasonic bath sonicator (Fisherbrand FB15051) and cleared by centrifugation. Cleared cell lysates were incubated overnight at 4°C with anti-FLAG afﬁnity resin (Sigma-Aldrich, A2220) to retain TRIM34-FLAG and TRIM34-FLAG-bound proteins, or to retain GBP1-FLAG and GBP1-FLAG-bound proteins; or with an anti-c-myc affinity resin (Sigma-Aldrich, A7470) to pull-down RAE1 and RAE1-c-myc-bound proteins, or to retain Nup93 and Nup93-c-myc-bound proteins. After washing three times in TBS buffer containing 0.1% tween-20, precipitated proteins were dissociated using 0.1 M glycine buffer at pH 2.4, denatured in loading buffer and incubated at 95°C, during 5 min. Protein samples were analyzed by electrophoresis and Western blotting, as described below, using the indicated specific antibodies.

### Western blots

Cell lysates and/or co-immunoprecipitation eluates were mixed with Laemmli sample buffer (Biorad) containing 2.5% β-mercaptoethanol, and heated at 95°C for 5 min, before sodium dodecyl sulfate (SDS)-polyacrylamide gel electrophoresis (PAGE), under denature conditions. Proteins were transferred to nitrocellulose membranes (Biorad), and detected using the primary antibodies: anti-TRIM34 (anti-RNF21/IFP1 antibody ab180130, Abcam), anti-FLAG (F3165, Sigma-Aldrich) to detect TRIM34-FLAG-tagged and GBP1-FLAG-tagged proteins, anti-IAV NP (40208-R117 and 40208-R010, EliteRmab Sino Biological Inc.), anti-IAV nonstructural protein 1 (NS1) (GTX125990, GeneTex), anti-GFP (11814460001, Merck) to detect Nup93-GFP-tagged protein or the GFP protein, anti-c-myc (9E10, Invitrogen) to detect RAE1-myc-tagged protein or the Nup93-myc-tagged protein, anti-HA (H6908, Sigma-Aldrich) to detect ubiquitin-HA-tagged protein, and anti-GAPDH (sc-47724, Santa Cruz Biotechnology). Then, the membranes were incubated with a 1:4000 dilution of goat anti-rabbit (pAb) or anti-mouse (mAb) IgG antibodies conjugated to horseradish peroxidase (Sigma-Aldrich). Nitrocellulose membranes were revealed by chemiluminescence with the SuperSignal west femto maximum sensitivity substrate (ThermoFisher Scientiﬁc), according to the manufacturer’s recommendations. Where indicated, protein bands have been quantiﬁed by densitometry using the ImageJ (Fiji) software.

### Mass spectrometry analysis

Protein immunoprecipitates were first reduced with 50 mM TCEP (pH 8.0) for 60 min at 37°C, and then, and alkylated with 200 mM methyl methanethiosulphonate (MMTS, Pierce) for 10 min at room temperature. Subsequently, S-trap microcolumns (PROTIFI) were used to improve the tryptic digestion yield of low-abundance samples, as previously reported [126]. For protein identification by tandem mass spectrometry (LC–MS/MS Exploris 240), the peptide samples were analyzed on a nano liquid chromatography system (Ultimate 3000 nano HPLC system, Thermo Fisher Scientific) coupled to an Orbitrap Exploris 240 mass spectrometer (Thermo Fisher Scientific). Samples (5 µL) were injected on a C18 PepMap trap column (5 µm, 100 µm I.D. x 2 cm, Thermo Scientific) at 30 µL/min, in water containing 0.1% formic acid. Afterwards, the trap column was switched on-line to a C18 PepMap Easy-spray analytical column (2 µm, 100 Å, 75 µm I.D. x 50 cm, Thermo Scientific). Equilibration was done in mobile phase A (0.1% formic acid in water), and peptide elution was performed in a 30 min gradient from 4% - 35% B (0.1% formic acid in 80% acetonitrile) at 300 nL/min. Data acquisition was performed using a data-dependent top-25 method, in full scan positive mode (range of 350–1200 m/z). Survey scans were acquired at a resolution of 60,000 at m/z 200, with Normalized Automatic Gain Control (AGC) target of 300% and a maximum injection time (IT) = Auto. The top 25 most intense ions from each MS1 scan were selected and fragmented by Higher-energy collisional dissociation (HCD) of 30. Resolution for HCD spectra was set to 15,000 at m/z 200, with AGC target of 75% and maximum ion injection time = Auto. Precursor ions with single, unassigned, or six and higher charge states from fragmentation selection were excluded.

MS and MS/MS raw data was translated to mascot general file (mgf) format and searched using an in-house Mascot Server v. 2.7 (Matrix Science, London, U.K.) against a human database (reference proteome from Uniprot Knowledgebase). Search parameters considered fixed MMTS alkylation of cysteine, and the following variable modifications: methionine oxidation, pyroglutamic acid from glutamine and glutamic acid at the peptide N-terminus, deamidation of asparagine/glutamine and acetylation of the protein N-terminus. Peptide mass tolerance was set to 10 ppm and 0.02 Da, in MS and MS/MS mode, respectively, and 3 missed cleavages were allowed. The Mascot confidence interval for protein identification was set to ≥ 95% (P < 0.05) and only peptides with a significant individual ion score of at least 20 were considered.

These specific proteins found on TRIM34 interactome were grouped according to their Gene Ontology (GO) biological processes using NIH DAVID software (https://davidbioinformatics.nih.gov/), selecting significantly enriched terms based on an False Discovery Rate (FDR)-adjusted p-value lower than 0.05.

### Immunofluorescence and confocal microscopy

Conﬂuent monolayers of human 293T cells were grown on sterile glass coverslips (24-well format) and were transiently transfected, using lipofectamine 3000 (ThermoFisher Scientiﬁc), with the pCAGGS plasmids expressing TRIM34-FLAG and the plasmid pEGFP-C1X expressing Nup93 fused to the EGFP protein or the pcDNA3.1-RAE1-myc. Additionally, human HeLa cells were transiently transfected with the pCAGGS plasmids expressing TRIM34-FLAG and IRF3-5D-GFP. Moreover, MDCK cells were infected with the rIAV-Venus, rIAV-mCherry or rIAV-TRIM34. Cells were ﬁxed and permeabilized with 10% formaldehyde and 0.1% Triton-X100 during 20 min at RT. Then, cells were blocked with 10% fetal bovine serum in PBS during 1h at RT. TRIM34 was detected with a mouse anti-FLAG antibody (FG4R, Invitrogen), Nup93 and IRF3-5D were identified through the GFP fluorescence signal encoded by the plasmids, RAE1 was detected with a rabbit anti-c-myc antibody (71D10, Cell Signaling). An anti-IAV NS1 protein rabbit antibody (GTX125990, GeneTex) was used to confirm comparable levels of infection with the recombinant viruses (rIAV-Venus, rIAV-mCherry and rIAV-TRIM34). Expression of Venus and mCherry was verified by their fluorescence signals. Coverslips were washed 4 times with PBS and stained with secondary anti-mouse and anti-rabbit antibodies conjugated to Alexa Fluor 647 and 488 (Invitrogen), and nuclei were stained using DAPI (ThermoFisher Scientiﬁc), during 45 min at RT. Coverslips were mounted in ProLong Gold Antifade Reagent (Invitrogen) and imaged with a Leica STELLARIS 5 confocal microscope or a MicroFluor/ZOE Fluorescent Cell Imager. All images were acquired under identical instrument settings and analyzed using Fiji software.

### Rluc, Gluc, and GFP assays

Human 293T cells were co-transfected with the pRL plasmid expressing Rluc (pRL-Rluc) under the control of a constitutive promoter (pRL-SV40, Promega), with a pCAGGS plasmid expressing Gluc (pCAGGs-Gaussia), or with a pCAGGs plasmid expressing GFP (pCAGGS-GFP, kindly provided by Prof. Luis Martínez-Sobrido, Texas Biomedical Research Institute, San Antonio, TX, US), the empty control pCAGGS plasmid or plasmids encoding TRIM34-FLAG, GBP1-FLAG, or RAE1-myc. At 24h post-transfection, cells were non-infected (mock) or infected with IAV (MOI 1). At 24h post-infection, cells transfected with the pRL-Rluc or the pCAGGs-Gaussia plasmids were lysed for 30 min on ice using 1X Rluc assay lysis buffer (Rluc Assay System, Promega) or using an 1X Gluc assay lysis buffer (Pierce Gluc assay kit, Thermo Fisher Scientific). Cell lysates were clarified by centrifugation at 13,000 rpm for 10 min at 4°C, and an equal volume of Rluc Assay Reagent (Promega) or Gluc Assay Reagent (Thermo Fisher Scientific) was added. Alternatively, cell culture supernatants from the cells transfected with the Gluc plasmid were mixed with the Gluc Assay Reagent (Thermo Fisher Scientific), to measure the extracellular protein levels. Rluc and Gluc protein expression levels were quantified using a luminometer (Tecan Infinite M200 PRO) and normalized to the levels of mock or IAV-infected cells transfected with the empty plasmid, respectively. Alternatively, cells transfected with the pCAGGS-GFP plasmid were lysed for 30 min on ice using the co-IP lysis buffer (NaCl 250 mM; EDTA 1 mM; 50 mM TrisHCl, pH 7.5; NP-40 0.5%) containing protease (ThermoFisher Scientific) and phosphatase (Merck) inhibitors, and the cellular extracts were analyzed by electrophoresis and Western blot using the GFP-specific antibody, or the anti-FLAG antibody and GAPDH-specific antibodies, as controls, as indicated above.

### *In vivo* experiments

Female 6-week-old C57BL/6 mice were purchased from Envigo and maintained in the animal care facility at the National Center for Biotechnology in a pathogen-free environment during one week before starting the infections. Mice were slightly anesthetized with ketamine/xylacine and then, intranasally inoculated with 2,000 FFU of the recombinant viruses. Mouse survival and weight loss was evaluated daily (n = 5 per group) until 14 days post-infection. Virus replication was evaluated by assessing viral titers in the lungs at 24-, and 48-hours post-infection (hpi) (n = 5 per group). To that end, mice were sacriﬁced and the right lung lobules were extracted and homogenized. Virus titers were determined by immunofocus assay on MDCK cells, as speciﬁed above. In addition, levels of CXCL10, C-C motif chemokine ligand 2 (CCL2), Interferon lambda 3 (IFNL3), IFNB1, Interleukin-1 beta (IL-1b), and IFIT2 mRNAs were analyzed in the mouse lungs at 24 and 48 hpi. For this, the left lung lobules were extracted and incubated in RNAlater (Ambion) at 4°C during 24 h prior to adding the lungs to RNA lysis buffer and homogenizing the lungs using a BeadBug homogenizer (Benchmark). Total RNA was extracted from homogenized lungs using the total RNA kit (Omega Biotech). RT reactions were performed at 37°C, during 2 h using the High-Capacity cDNA transcription kit and random hexamers (ThermoFisher Scientific) to generate the cDNAs. qPCRs were performed using Taqman gene expression assays (Applied Biosystems) specific for the murine CXCL10 (Mm00445235_m1), CCL2 (Mm00441242_m1), IFNL3 (Mm00663660_g1), IFNB1 (Mm00439552_s1), IL-1b (Mm00434228_m1), IFIT2 (Mm00492606_m1) and GAPDH (Mm99999915_g1) genes. Data from qPCR was assessed following threshold cycle (2^-ΔΔCT^) methodology [122] and normalized with GAPDH expression levels.

### Nuclear and cytoplasmic fractionation

Human 293T cells (6-well plate format) were transiently transfected for 24h with the pCAGGS plasmid expressing TRIM34-FLAG protein or the empty control. Then, 293T cells were treated with IFN and infected with IAV (MOI 1) for an additional 24h. Subsequently, cells were harvested and fractionated into nuclear and cytoplasmic RNA, as previously described [127]. RNA was extracted using the TRIzol reagent, according to the manufacturer’s instructions (Invitrogen). Nuclear and cytoplasmic RNA was reverse-transcribed to cDNA using a High-Capacity cDNA Reverse-Transcription kit and random primers. Fractionation procedure was validated using GAPDH mRNA and MALAT1 long non-coding RNA, as cytoplasmic and nuclear markers, respectively, as reported previously [128,129]. cDNA was analyzed by qPCR with Power SYBR Green PCR mix (ThermoFisher Scientific) and primers designed to amplify *Homo sapiens* GAPDH mRNA (Forward: 5´-TGCACCACCAACTGCTTAGC-3 and Reverse: 5´-GGCATGGACTGTGGTCATGAG-3’) and MALAT1 long non-coding RNA (Forward: 5´-GGTGAATTGATAAGTAAAGGCAGAA-3’ and Reverse: 5´- TCAGTAGTAAGAATCTCAGGGTTATG-3’) [129]. In addition, qPCR was performed for the human genes CXCL10, IFNB1, IFNL1, IFIT2, and IFI27 (Hs01086373_g1) using TaqMan Gene Expression Assays (Applied Biosystems), and for viral mRNAs (PB2, HA, NA, and NP) using gene-specific primers (Tables F and G in S2 Appendix) with SYBR Green PCR Master Mix. All reactions were conducted as described above in the “Quantitative PCR Assays” section. For data analysis, qPCR results were expressed as the nuclear-to-cytoplasmic (nucleus/cytoplasm) mRNA ratio.

### Self-ubiquitylation assay

Human 293T cells (6-well plate format) were transiently co-transfected for 24h with pCAGGS plasmids encoding FLAG-tagged TRIM34 mutants (H32A, E20K and I17L/L19A), the wild-type (WT) TRIM34, or the empty control plasmid, together with the plasmid expressing ubiquitin-HA, using lipofectamine 3000 (ThermoFisher Scientiﬁc). Then, cells were lysed in the co-IP buffer and the cellular lysates were subjected to anti-FLAG immunoprecipitation as described above in the “Co-immunoprecipitation assays”. Immunoprecipitated proteins were analyzed by electrophoresis and Western blot as described above, using anti-FLAG (TRIM34) and anti-HA (ubiquitin) antibodies.

### Statistical analysis

All data are presented as the mean ± standard deviation (SD). Statistical significance was determined using Student’s t-test with Holm-Šídák correction, or one-way ANOVA with Dunnett’s post-hoc test, or two-way ANOVA with Tukey’s post-hoc test, as appropriate. Values of *p* < 0.05 were considered significant (*p < 0.05, **p < 0.01, ***p < 0.001, ****p < 0.0001). Analyses were performed in GraphPad Prism v10.4.2.

## Supporting information

S1 Appendix Fig A. (**A**) TRIM34 endogenous protein expression was detected in human A549 and 293T mock-infected cells by Western blot using a specific antibody for TRIM34. The anti-GAPDH antibody was used as a loading control. (**B**) The percentage of infected cells, by means of an immunofluorescence using an anti-NP specific antibody was determined in 293T cells transfected with the empty plasmid, or in cells transfected with the pCAGGs-TRIM34-FLAG plasmid. (**C**) TRIM34-FLAG protein levels were analyzed by Western blot, using an anti-FLAG antibody, in cells transfected with the empty plasmid or the pCAGGS-TRIM34-FLAG plasmid, at 24 or 48 h after IAV infection. **(D)** TRIM34 protein levels were analyzed by Western blot, using an anti-TRIM34 antibody, in cells transfected with the empty plasmid or the pCAGGS-TRIM34-FLAG plasmid. **(E)** TRIM34 mRNA levels were quantified by RT-qPCR in 293T cells transfected with the non-targeted (NT) siRNA or with the TRIM34-specific siRNA, at 24 and 48 h after IAV infection. *p < 0.05 for comparisons between NT siRNA and TRIM34 knocked-down cells, using Student’s t-test with Holm-Šídák correction. **(F)** TRIM34 endogenous protein expression was detected in human 293T transfected with the non-targeted (NT) siRNA or with the TRIM34-specific siRNA by Western blot using a specific antibody for TRIM34. An anti-GAPDH antibody served as a loading control, and an anti-IAV NP antibody was used to confirm infection. Protein bands in C, D, and F were quantified using the ImageJ software and normalized to the levels of GAPDH expression (numbers below the blots). Molecular weight markers (in kilodaltons) are indicated on the right. **Fig B.** Human 293T cells were transfected with the pCAGGS plasmid encoding GBP1-FLAG or the empty control plasmid. Co-immunoprecipitation (co-IP) experiments using agarose beads conjugated to an anti-FLAG antibody, to pull down GBP1 were performed. GBP1 was detected by Western blotting using an anti-FLAG antibody in the cellular lysates (input) and after the co-IP. Molecular weight markers (in kilodaltons) are indicated on the right. The lower band in the IP blot corresponds to the heavy chain of the antibody used for the co-IP (marked with an *). **Fig C.** Human 293T cells were co-transfected with the pCAGGS plasmid expressing the NS1 fused to an HA tag alone or together with the plasmid encoding TRIM34-FLAG. Co-immunoprecipitation (Co-IP) experiments were performed using **(A)** an anti-FLAG antibody, to pull down TRIM34, **(B)** or using an anti-HA antibody, to pull down NS1. **(A, B)** TRIM34 and NS1 were detected by Western blotting using anti-FLAG or anti-HA antibodies in the cellular lysates (input) and after the co-IP. Molecular weight markers (in kilodaltons) are indicated on the right. **Fig D.** Human 293T cells were co-transfected with a plasmid constitutively expressing GFP, and the plasmid encoding TRIM34-FLAG, or the plasmid encoding GBP1-FLAG, or the empty plasmid, as controls. At 24hpt, the levels of GFP expression were analyzed by Western blot using an anti-GFP antibody, and normalized to the levels of GAPDH expression, using the ImageJ software (numbers below the blot). Molecular weight markers (in kilodaltons) are indicated on the right. **Fig E.** (**A**) TRIM34-FLAG protein levels were analyzed by Western blot, using an anti-FLAG antibody, in cells transfected with the empty plasmid or the pCAGGS-TRIM34-FLAG plasmid, at 24h after mock-treatment or treatment with IFN. (**B**) TRIM34-FLAG protein levels were analyzed by Western blot, using an anti-FLAG antibody, in cells transfected with the empty plasmid or the pCAGGS-TRIM34-FLAG plasmid, at 24h after IAV WT or IAV ΔNS1 infection. Human A549 cells **(C)** and or BEAS-2B **(D)** were transfected twice with NT control siRNA or TRIM34 siRNA, 24h apart, for two consecutive days. On day 3, cells were treated with IFN for an additional 24h. **(C, D)** TRIM34 expression was measured by RT-qPCR and mRNA levels were expressed as fold-change (increases) in comparison to mock-treated cells, transfected with the NT siRNA, used as control. *p < 0.05 for comparisons between NT siRNA and TRIM34 knocked-down cells, using Student’s t-test with Holm-Šídák correction. **Fig F.** Human 293T cells were transfected with the pCAGGS plasmids encoding TRIM34-FLAG or the empty plasmid, as control. At 24h post-transfection, cells were treated with IFN or infected with IAV. At 24hpi, cells were fractionated into cytoplasm and nuclear RNA fractions. Nuclear-to-cytoplasmic (N/C) ratios of GAPDH mRNA (cytoplasmic marker) and MALAT1 long non-coding RNA (nuclear marker) levels were calculated to validate fractionation procedure.
(PDF)

S2 Appendix Table A. **Mass spectrometry identification of TRIM34 protein.** Human 293T cells were co-transfected with the pCAGGS plasmid encoding TRIM34-FLAG or the empty plasmid, as control. At 24hpt, cells were mock-infected o infected with IAV (MOI 1). Mass spectrometry identification of protein TRIM34 after FLAG pull-down. Protein accession, description, molecular weight, isoelectric point, sample, group ID, Mascot score, PSMs, peptides, unique peptides, and sequence coverage are shown. **Table B. Qualitative proteomic analysis of proteins detected exclusively in TRIM34-FLAG–overexpressing cells.** Proteins were identified by mass spectrometry following FLAG pull-down, and only those uniquely present in TRIM34-FLAG–overexpressing cells compared with control cells (transfected with the empty plasmid) are shown. Protein accession, description, molecular weight, isoelectric point, group ID, Mascot score, PSMs, peptides, unique peptides, and sequence coverage are shown, ordered by Mascot score. **Table C. Biological process organization of mass spectrometry data.** Gene Ontology (GO) enrichment analysis of TRIM34 interactome proteins using DAVID. Shown are enriched GO biological processes in mock and IAV samples with FDR < 0.05, including FDR, -log10(FDR), fold enrichment, genes, and counts, ordered by increasing FDR (most to least significant). **Table D. Mass spectrometry identification of GBP1 protein.** Human 293T cells were transfected with the pCAGGS plasmid encoding GBP1-FLAG or the empty plasmid, as control. At 24hpt, cells were mock-infected o infected with IAV (MOI 1). Mass spectrometry identification of protein GBP1 after FLAG pull-down. Protein accession, description, molecular weight, isoelectric point, sample, group ID, Mascot score, PSMs, peptides, unique peptides, and sequence coverage are shown. **Table E. Qualitative proteomic analysis of proteins detected exclusively in GBP1-FLAG–overexpressing cells.** Proteins were identified by mass spectrometry following FLAG pull-down, and only those uniquely present in GBP1-FLAG–overexpressing cells compared with control cells (transfected with the empty plasmid) are shown. Protein accession, description, molecular weight, isoelectric point, group ID, Mascot score, PSMs, peptides, unique peptides, and sequence coverage are shown, ordered by Mascot score. **Table F. Strand-specific primers used for reverse transcription (RT) Table G. Gene-specific primers used for qPCR analysis.**
(DOCX)

## References

[1] Atmar RL, Lindstrom SE. Influenza Viruses. In: Jorgensen JH, Carroll KC, Funke G, Pfaller MA, Landry ML, Richter SS, et al., editors. Manual of Clinical Microbiology [Internet]. Washington, DC, USA: ASM Press; 2015 [cited 2025 Sep 5]. p. 1470–86. Available from: http://doi.wiley.com/10.1128/9781555817381.ch84

[2] Kyaw MH, Chen SB, Wu S, Foo CY, Welch V, Boikos C, et al. Systematic review on influenza burden in emerging markets in 2018–2023—an evidence update to guide influenza vaccination recommendations. Vaccines. 2024;12(11):1251. doi: 10.3390/vaccines12111251 39591154 PMC11599016

[3] World Health Organization. Clinical practice guidelines for influenza [Internet]. Geneva: WHO; 2024. Report No. Available from: https://www.who.int/publications/i/item/9789240097759 39374347

[4] Schneider WM, Chevillotte MD, Rice CM. Interferon-stimulated genes: a complex web of host defenses. Annu Rev Immunol. 2014;32:513–45. doi: 10.1146/annurev-immunol-032713-120231 24555472 PMC4313732

[5] Schoggins JW. Interferon-Stimulated Genes: What Do They All Do?. Annual Review of Virology. 2019;6(1):567–84. doi: 10.1146/annurev-virology-092818-015756 31283436

[6] Xu Q, Tang Y, Huang G. Innate immune responses in RNA viral infection. Front Med. 2021;15(3):333–46. doi: 10.1007/s11684-020-0776-7 33263837 PMC7862985

[7] Wilkins C, Gale M Jr. Recognition of viruses by cytoplasmic sensors. Curr Opin Immunol. 2010;22(1):41–7. doi: 10.1016/j.coi.2009.12.003 20061127 PMC3172156

[8] Jensen S, Thomsen AR. Sensing of RNA viruses: a review of innate immune receptors involved in recognizing RNA virus invasion. J Virol. 2012 Mar;86(6):2900–10. 10.1128/JVI.05738-11 PMC3302314 22258243

[9] Gürtler C, Bowie AG. Innate immune detection of microbial nucleic acids. Trends Microbiol. 2013 Aug;21(8):413–20. 10.1016/j.tim.2013.04.004 PMC3735846 23726320

[10] Rehwinkel J, Gack MU. RIG-I-like receptors: their regulation and roles in RNA sensing. Nat Rev Immunol. 2020;20(9):537–51. doi: 10.1038/s41577-020-0288-3 32203325 PMC7094958

[11] Fleming SB. Viral Inhibition of the IFN-Induced JAK/STAT Signalling Pathway: Development of Live Attenuated Vaccines by Mutation of Viral-Encoded IFN-Antagonists. Vaccines (Basel). 2016;4(3):23. doi: 10.3390/vaccines4030023 27367734 PMC5041017

[12] Komuro A, Bamming D, Horvath CM. Negative regulation of cytoplasmic RNA-mediated antiviral signaling. Cytokine. 2008;43(3):350–8. doi: 10.1016/j.cyto.2008.07.011 18703349 PMC2575845

[13] Richards KH, Macdonald A. Putting the brakes on the anti-viral response: negative regulators of type I interferon (IFN) production. Microbes Infect. 2011;13(4):291–302. doi: 10.1016/j.micinf.2010.12.007 21256242

[14] Kikkert M. Innate immune evasion by human respiratory RNA viruses. J Innate Immun. 2020;12(1):4–20. doi: 10.1159/000503030 31610541 PMC6959104

[15] Chathuranga K, Weerawardhana A, Dodantenna N, Lee J-S. Regulation of antiviral innate immune signaling and viral evasion following viral genome sensing. Exp Mol Med. 2021;53(11):1647–68. doi: 10.1038/s12276-021-00691-y 34782737 PMC8592830

[16] Shen Q, Wang YE, Palazzo AF. Crosstalk between nucleocytoplasmic trafficking and the innate immune response to viral infection. J Biol Chem. 2021;297(1):100856. doi: 10.1016/j.jbc.2021.100856 34097873 PMC8254040

[17] Zhang X, Lim K, Qiu Y, Hazawa M, Wong RW. Strategies for the Viral Exploitation of Nuclear Pore Transport Pathways. Viruses. 2025;17(2):151. doi: 10.3390/v17020151 40006906 PMC11860923

[18] Michalska A, Blaszczyk K, Wesoly J, Bluyssen HAR. A positive feedback amplifier circuit that regulates interferon (IFN)-stimulated gene expression and controls type I and type II IFN responses. Frontiers in Immunology. 2018;9:1135. doi: 10.3389/fimmu.2018.01135 29892288 PMC5985295

[19] Villamayor L, Rivero V, López-García D, Topham DJ, Martínez-Sobrido L, Nogales A, et al. Interferon alpha inducible protein 6 is a negative regulator of innate immune responses by modulating RIG-I activation. Front Immunol. 2023;14:1105309. doi: 10.3389/fimmu.2023.1105309 36793726 PMC9923010

[20] Monwan W, Kawasaki T, Hasan MZ, Ori D, Kawai T. Identification of nucleoporin 93 (Nup93) that mediates antiviral innate immune responses. Biochem Biophys Res Commun. 2020;521(4):1077–82. doi: 10.1016/j.bbrc.2019.11.035 31733835

[21] Kumar KP, McBride KM, Weaver BK, Dingwall C, Reich NC. Regulated nuclear-cytoplasmic localization of interferon regulatory factor 3, a subunit of double-stranded RNA-activated factor 1. Mol Cell Biol. 2000;20(11):4159–68. doi: 10.1128/MCB.20.11.4159-4168.2000 10805757 PMC85785

[22] Thompson MR, Kaminski JJ, Kurt-Jones EA, Fitzgerald KA. Pattern recognition receptors and the innate immune response to viral infection. Viruses. 2011;3(6):920–40. doi: 10.3390/v3060920 21994762 PMC3186011

[23] Satterly N, Tsai PL, Van Deursen J, Nussenzveig DR, Wang Y, Faria PA. Influenza virus targets the mRNA export machinery and the nuclear pore complex. Proc Natl Acad Sci USA. 2007;104(6):1853–8. doi: 10.1073/pnas.0610977104 17267598 PMC1794296

[24] Yarbrough ML, Mata MA, Sakthivel R, Fontoura BMA. Viral subversion of nucleocytoplasmic trafficking. Traffic. 2014;15(2):127–40. doi: 10.1111/tra.12137 24289861 PMC3910510

[25] Paterson D, Fodor E. Emerging roles for the influenza A virus nuclear export protein (NEP). PLoS Pathog. 2012;8(12):e1003019. doi: 10.1371/journal.ppat.1003019 PMC3516560 23236273

[26] Cao L, She Z, Zhao Y, Cheng C, Li Y, Xu T, et al. Inhibition of RAN attenuates influenza a virus replication and nucleoprotein nuclear export. Emerg Microbes Infect. 2024;13(1):2387910. doi: 10.1080/22221751.2024.2387910 39087696 PMC11321118

[27] Jia D, Rahbar R, Chan RWY, Lee SMY, Chan MCW, Wang BX, et al. PLoS ONE. 2010;5(11):e13927. doi: 10.1371/journal.pone.0013927 PMC2978095 21085662

[28] Khanna M, Sharma K, Saxena SK, Sharma JG, Rajput R, Kumar B. Unravelling the interaction between Influenza virus and the nuclear pore complex: insights into viral replication and host immune response. Virusdisease. 2024;35(2):231–42. doi: 10.1007/s13337-024-00879-6 39071870 PMC11269558

[29] Zhang K, Xie Y, Muñoz-Moreno R, Wang J, Zhang L, Esparza M, et al. Structural basis for influenza virus NS1 protein block of mRNA nuclear export. Nat Microbiol. 2019;4(10):1671–9. doi: 10.1038/s41564-019-0482-x 31263181 PMC6754785

[30] Esparza M, Bhat P, Fontoura BM. Viral-host interactions during splicing and nuclear export of influenza virus mRNAs. Curr Opin Virol. 2022;55:101254. doi: 10.1016/j.coviro.2022.101254 35908311 PMC9945342

[31] Nemeroff ME, Barabino SM, Li Y, Keller W, Krug RM. Influenza virus NS1 protein interacts with the cellular 30 kDa subunit of CPSF and inhibits 3’end formation of cellular pre-mRNAs. Mol Cell. 1998;1(7):991–1000. doi: 10.1016/s1097-2765(00)80099-4 9651582

[32] Guo J, Zhu Y, Ma X, Shang G, Liu B, Zhang K. Int J Mol Sci. 2023;24(16):12593. doi: 10.3390/ijms241612593 37628773 PMC10454920

[33] Koepke L, Gack MU, Sparrer KM. The antiviral activities of TRIM proteins. Curr Opin Microbiol. 2021;59:50–7. doi: 10.1016/j.mib.2020.07.005 32829025 PMC7440025

[34] D’Cruz AA, Babon JJ, Norton RS, Nicola NA, Nicholson SE. Structure and function of the SPRY/B30.2 domain proteins involved in innate immunity. Protein Sci. 2013;22(1):1–10. doi: 10.1002/pro.2185 23139046 PMC3575854

[35] van Tol S, Hage A, Giraldo MI, Bharaj P, Rajsbaum R. The TRIMendous Role of TRIMs in Virus-Host Interactions. Vaccines (Basel). 2017;5(3):23. doi: 10.3390/vaccines5030023 28829373 PMC5620554

[36] Van Gent M, Sparrer KMJ, Gack MU. TRIM Proteins and Their Roles in Antiviral Host Defenses. Annual Review of Virology. 2018;5(1):385–405. doi: 10.1146/annurev-virology-092917-043323 PMC6186430 29949725

[37] Lee H-R, Lee MK, Kim CW, Kim M. TRIM Proteins and Their Roles in the Influenza Virus Life Cycle. Microorganisms. 2020;8(9):1424. doi: 10.3390/microorganisms8091424 32947942 PMC7565951

[38] Rajsbaum R, Stoye JP, O’Garra A. Type I interferon-dependent and -independent expression of tripartite motif proteins in immune cells. Eur J Immunol. 2008;38(3):619–30. doi: 10.1002/eji.200737916 18286572

[39] Orimo A, Tominaga N, Yoshimura K, Yamauchi Y, Nomura M, Sato M, et al. Molecular cloning of ring finger protein 21 (RNF21)/interferon-responsive finger protein (ifp1), which possesses two RING–B box–coiled coil domains in tandem. Genomics. 2000;69(1):143. doi: 10.1006/geno.2000.6318 11013086

[40] Sawyer SL, Emerman M, Malik HS. Discordant evolution of the adjacent antiretroviral genes TRIM22 and TRIM5 in mammals. PLoS Pathog. 2007;3(12):e197. doi: 10.1371/journal.ppat.0030197 18159944 PMC2151084

[41] Zhang F, Hatziioannou T, Perez-Caballero D, Derse D, Bieniasz PD. Antiretroviral potential of human tripartite motif-5 and related proteins. Virology. 2006;353(2):396–409. doi: 10.1016/j.virol.2006.05.035 16828831

[42] Li X, Gold B, O’hUigin C, Diaz-Griffero F, Song B, Si Z, et al. Unique features of TRIM5alpha among closely related human TRIM family members. Virology. 2007;360(2):419–33. doi: 10.1016/j.virol.2006.10.035 17156811

[43] Ohainle M, Kim K, Komurlu Keceli S, Felton A, Campbell E, Luban J, et al. PLoS Pathogens. 2020;16(4):e1008507. doi: 10.1371/journal.ppat.1008507 PMC7179944 32282853

[44] Wang X, Xiong J, Zhou D, Zhang S, Wang L, Tian Q, et al. TRIM34 modulates influenza virus-activated programmed cell death by targeting Z-DNA-binding protein 1 for K63-linked polyubiquitination. J Biol Chem. 2022;298(3):101611. doi: 10.1016/j.jbc.2022.101611 35065966 PMC8867111

[45] An X, Ji B, Sun D. TRIM34 localizes to the mitochondria and mediates apoptosis through the mitochondrial pathway in HEK293T cells. Heliyon. 2020;6(1):e03115. doi: 10.1016/j.heliyon.2019.e03115 31956709 PMC6956761

[46] Chaudhari K, Vasu VT, Golani A, Shaikh A, Nagariya N, Roy H. Interferon Induced Upregulation of Tripartite Motif 34 (TRIM34) Leads Apoptotic Cell Death in Lung Adenocarcinoma. J Biochem Mol Toxicol. 2024;38(12):e70072. doi: 10.1002/jbt.70072 39607040

[47] Zhang P, Chen Z, Li J, Mao H, Hu Y. TRIM34 suppresses non-small-cell lung carcinoma via inducing mTORC1-dependent glucose utilization and promoting cellular death. Arch Biochem Biophys. 2024;754:109925. doi: 10.1016/j.abb.2024.109925 38336254

[48] Yao F, Zhou S, Zhang R, Chen Y, Huang W, Yu K, et al. CRISPR/Cas9 screen reveals that targeting TRIM34 enhances ferroptosis sensitivity and augments immunotherapy efficacy in hepatocellular carcinoma. Cancer Lett. 2024;593:216935. doi: 10.1016/j.canlet.2024.216935 38704136

[49] Li Y, Shan J, Liu S, Shen Y, Niu L, Mao Q, et al. TRIM34 inhibits triple-negative breast cancer progression by attenuating fatty acid synthesis via facilitating FASN ubiquitination. Biochim Biophys Acta Mol Basis Dis. 2025;1871(7):167958. doi: 10.1016/j.bbadis.2025.167958 40543677

[50] Carthagena L, Bergamaschi A, Luna JM, David A, Uchil PD, Margottin-Goguet F. PLoS ONE. 2009;4(3):e4894. doi: 10.1371/journal.pone.0004894 PMC2654144 19290053

[51] Sun D, An X, Ji B. TRIM34 facilitates the formation of multinucleated giant cells by enhancing cell fusion and phagocytosis in epithelial cells. Exp Cell Res. 2019;384(1):111594. doi: 10.1016/j.yexcr.2019.111594 31487507

[52] Villamayor L, López-García D, Rivero V, Martínez-Sobrido L, Nogales A, DeDiego ML. The IFN-stimulated gene IFI27 counteracts innate immune responses after viral infections by interfering with RIG-I signaling. Front Microbiol. 2023;14:1176177. doi: 10.3389/fmicb.2023.1176177 37187533 PMC10175689

[53] Guo H, An S, Ward R, Yang Y, Liu Y, Guo X-X, et al. Methods used to study the oligomeric structure of G-protein-coupled receptors. Biosci Rep. 2017;37(2):BSR20160547. doi: 10.1042/BSR20160547 28062602 PMC5398257

[54] Khare S, Radian AD, Dorfleutner A, Stehlik C. Measuring NLR Oligomerization I: Size Exclusion Chromatography, Co-immunoprecipitation, and Cross-Linking. Methods Mol Biol. 2016;1417:131–43. doi: 10.1007/978-1-4939-3566-6_8 27221486 PMC5058782

[55] García-Sastre A, Egorov A, Matassov D, Brandt S, Levy DE, Durbin JE, et al. Influenza A virus lacking the NS1 gene replicates in interferon-deficient systems. Virology. 1998;252(2):324–30. doi: 10.1006/viro.1998.9508 9878611

[56] Haller O, Kochs G, Weber F. The interferon response circuit: induction and suppression by pathogenic viruses. Virology. 2006;344(1):119–30. doi: 10.1016/j.virol.2005.09.024 16364743 PMC7125643

[57] Opitz B, Rejaibi A, Dauber B, Eckhard J, Vinzing M, Schmeck B, et al. IFNbeta induction by influenza A virus is mediated by RIG-I which is regulated by the viral NS1 protein. Cell Microbiol. 2007;9(4):930–8. doi: 10.1111/j.1462-5822.2006.00841.x 17140406

[58] Nogales A, Martinez-Sobrido L, Topham DJ, DeDiego ML. Modulation of innate immune responses by the influenza A NS1 and PA-X proteins. Viruses. 2018;10(12):708. doi: 10.3390/v10120708 30545063 PMC6315843

[59] Nogales A, DeDiego ML, Martínez-Sobrido L. Live attenuated influenza A virus vaccines with modified NS1 proteins for veterinary use. Front Cell Infect Microbiol. 2022;12:954811. doi: 10.3389/fcimb.2022.954811 35937688 PMC9354547

[60] Zhang K, Shang G, Padavannil A, Wang J, Sakthivel R, Chen X. Structural–functional interactions of NS1-BP protein with the splicing and mRNA export machineries for viral and host gene expression. Proc Natl Acad Sci USA. 2018;115(52). doi: 10.1073/pnas.1818012115 PMC6310826 30538201

[61] Zhang K, Cagatay T, Xie D, Angelos AE, Cornelius S, Aksenova V, et al. Cellular NS1-BP protein interacts with the mRNA export receptor NXF1 to mediate nuclear export of influenza virus M mRNAs. J Biol Chem. 2024;300(11):107871. doi: 10.1016/j.jbc.2024.107871 39384042 PMC11570952

[62] Kuss S, Mata M, Zhang L, Fontoura B. Nuclear imprisonment: viral strategies to arrest host mRNA nuclear export. Viruses. 2013;5(7):1824–49. doi: 10.3390/v5071824 23872491 PMC3738964

[63] Lin R, Mamane Y, Hiscott J. Structural and functional analysis of interferon regulatory factor 3: localization of the transactivation and autoinhibitory domains. Mol Cell Biol. 1999;19(4):2465–74. doi: 10.1128/MCB.19.4.2465 10082512 PMC84039

[64] Schwanke H, Stempel M, Brinkmann MM. Of Keeping and Tipping the Balance: Host Regulation and Viral Modulation of IRF3-Dependent IFNB1 Expression. Viruses. 2020;12(7):733. doi: 10.3390/v12070733 32645843 PMC7411613

[65] Pritchard CE, Fornerod M, Kasper LH, van Deursen JM. RAE1 is a shuttling mRNA export factor that binds to a GLEBS-like NUP98 motif at the nuclear pore complex through multiple domains. J Cell Biol. 1999;145(2):237–54. doi: 10.1083/jcb.145.2.237 10209021 PMC2133102

[66] Faria PA, Chakraborty P, Levay A, Barber GN, Ezelle HJ, Enninga J. VSV Disrupts the Rae1/mrnp41 mRNA Nuclear Export Pathway. Molecular Cell. 2005;17(1):93–102. doi: 10.1016/j.molcel.2004.11.023 15629720

[67] Ren Y, Seo HS, Blobel G, Hoelz A. Structural and functional analysis of the interaction between the nucleoporin Nup98 and the mRNA export factor Rae1. Proc Natl Acad Sci USA. 2010;107(23):10406–11. doi: 10.1073/pnas.1005389107 20498086 PMC2890840

[68] DeDiego ML, Nogales A, Lambert-Emo K, Martinez-Sobrido L, Topham DJ. NS1 protein mutation I64T affects interferon responses and virulence of circulating H3N2 human influenza A viruses. J Virol. 2016;90(21):9693–711. doi: 10.1128/JVI.01039-16 27535054 PMC5068522

[69] Nogales A, Martinez-Sobrido L, Topham DJ, DeDiego ML. NS1 Protein Amino Acid Changes D189N and V194I Affect Interferon Responses, Thermosensitivity, and Virulence of Circulating H3N2 Human Influenza A Viruses. J Virol. 2017;91(5):e01930-16. doi: 10.1128/JVI.01930-16 28003482 PMC5309952

[70] Gustin KE. Inhibition of nucleo-cytoplasmic trafficking by RNA viruses: targeting the nuclear pore complex. Virus Res. 2003;95(1–2):35–44. doi: 10.1016/s0168-1702(03)00165-5 12921994 PMC7125697

[71] Han M, Ke H, Zhang Q, Yoo D. Nuclear imprisonment of host cellular mRNA by nsp1β protein of porcine reproductive and respiratory syndrome virus. Virology. 2017;505:42–55. doi: 10.1016/j.virol.2017.02.004 28235682 PMC7111332

[72] Sajidah ES, Lim K, Wong RW. How SARS-CoV-2 and other viruses build an invasion route to hijack the host nucleocytoplasmic trafficking system. Cells. 2021;10(6):1424. doi: 10.3390/cells10061424 34200500 PMC8230057

[73] Guillen JV, Glaunsinger BA. SARS-CoV-2 Nsp1 Traps RNA in the Nucleus to Escape Immune Detection. Proceedings of the National Academy of Sciences of the United States of America. 2024;121(25):e2408794121. doi: 10.1073/pnas.2408794121 PMC11194585 38843251

[74] Lienlaf M, Hayashi F, Di Nunzio F, Tochio N, Kigawa T, Yokoyama S, et al. Contribution of E3-ubiquitin ligase activity to HIV-1 restriction by TRIM5alpha(rh): structure of the RING domain of TRIM5alpha. J Virol. 2011;85(17):8725–37. doi: 10.1128/JVI.00497-11 21734049 PMC3165826

[75] Iwasaki A, Medzhitov R. Control of adaptive immunity by the innate immune system. Nat Immunol. 2015;16(4):343–53. doi: 10.1038/ni.3123 25789684 PMC4507498

[76] Medzhitov R. Origin and physiological roles of inflammation. Nature. 2008;454(7203):428–35. doi: 10.1038/nature07201 18650913

[77] Short KR, Kroeze EJBV, Fouchier RAM, Kuiken T. Pathogenesis of influenza-induced acute respiratory distress syndrome. Lancet Infect Dis. 2014;14(1):57–69. doi: 10.1016/S1473-3099(13)70286-X 24239327

[78] Tisoncik JR, Korth MJ, Simmons CP, Farrar J, Martin TR, Katze MG. Into the eye of the cytokine storm. Microbiol Mol Biol Rev. 2012;76(1):16–32. doi: 10.1128/MMBR.05015-11 22390970 PMC3294426

[79] DeDiego ML, Martinez-Sobrido L, Topham DJ. Novel functions of IFI44L as a feedback regulator of host antiviral responses. J Virol. 2019;93(21):e01159-19. doi: 10.1128/JVI.01159-19 PMC6803278 31434731

[80] DeDiego ML, Nogales A, Martinez-Sobrido L, Topham DJ. Interferon-Induced Protein 44 Interacts with Cellular FK506-Binding Protein 5, Negatively Regulates Host Antiviral Responses, and Supports Virus Replication. mBio. 2019;10(4):e01839-19. doi: 10.1128/mBio.01839-19 PMC6712396 31455651

[81] Rivero V, Carrión-Cruz J, López-García D, DeDiego ML. The IFN-induced protein IFI27 binds MDA5 and counteracts its activation after SARS-CoV-2 infection. Front Cell Infect Microbiol. 2024;14:1470924. doi: 10.3389/fcimb.2024.1470924 39431052 PMC11486742

[82] Castelló A, Izquierdo JM, Welnowska E, Carrasco L. RNA nuclear export is blocked by poliovirus 2A protease and is concomitant with nucleoporin cleavage. J Cell Sci. 2009;122(Pt 20):3799–809. doi: 10.1242/jcs.055988 19789179

[83] Gustin KE. Effects of poliovirus infection on nucleo-cytoplasmic trafficking and nuclear pore complex composition. EMBO J. 2001;20(1):240–9. doi: 10.1093/emboj/20.1.240 11226174 PMC140206

[84] Gustin KE, Sarnow P. Inhibition of nuclear import and alteration of nuclear pore complex composition by rhinovirus. J Virol. 2002;76(17):8787–96. doi: 10.1128/jvi.76.17.8787-8796.2002 12163599 PMC136411

[85] Park N, Skern T, Gustin KE. Specific cleavage of the nuclear pore complex protein Nup62 by a viral protease. J Biol Chem. 2010;285(37):28796–805. doi: 10.1074/jbc.M110.143404 20622012 PMC2937907

[86] Watters K, Palmenberg AC. Differential processing of nuclear pore complex proteins by rhinovirus 2A proteases from different species and serotypes. J Virol. 2011;85(20):10874–83. doi: 10.1128/JVI.00718-11 21835805 PMC3187490

[87] Porter FW, Bochkov YA, Albee AJ, Wiese C, Palmenberg AC. A picornavirus protein interacts with Ran-GTPase and disrupts nucleocytoplasmic transport. Proc Natl Acad Sci U S A. 2006;103(33):12417–22. doi: 10.1073/pnas.0605375103 16888036 PMC1567894

[88] Porter FW, Palmenberg AC. Leader-induced phosphorylation of nucleoporins correlates with nuclear trafficking inhibition by cardioviruses. J Virol. 2009;83(4):1941–51. doi: 10.1128/JVI.01752-08 19073724 PMC2643766

[89] Brooks AJ, Johansson M, Criswell E, Jans DA, Vasudevan SG. The interdomain region of dengue NS5 protein interacts with NS3 and host proteins. Dengue Bull. 2002;26:155–61.

[90] Cheng CX, Tan MJA, Chan KWK, Choy MMJ, Roman N, Arnold DDR, et al. Serotype-Specific Regulation of Dengue Virus NS5 Protein Subcellular Localization. ACS Infect Dis. 2024;10(6):2047–62. doi: 10.1021/acsinfecdis.4c00054 38811007 PMC11184549

[91] Johansson M, Brooks AJ, Jans DA, Vasudevan SG. A small region of the dengue virus-encoded RNA-dependent RNA polymerase, NS5, confers interaction with both the nuclear transport receptor importin-β and the viral helicase, NS3. J Gen Virol. 2001;82(4):735–45. doi: 10.1099/0022-1317-82-4-735 11257177

[92] Petersen JM, Her LS, Varvel V, Lund E, Dahlberg JE. The matrix protein of vesicular stomatitis virus inhibits nucleocytoplasmic transport when it is in the nucleus and associated with nuclear pore complexes. Mol Cell Biol. 2000;20(22):8590–601. doi: 10.1128/MCB.20.22.8590-8601.2000 11046154 PMC102164

[93] Rajani KR, Pettit Kneller EL, McKenzie MO, Horita DA, Chou JW, Lyles DS. PLoS Pathog. 2012;8(9):e1002929. doi: 10.1371/journal.ppat.1002929 PMC3460625 23028327

[94] von Kobbe C, van Deursen JM, Rodrigues JP, Sitterlin D, Bachi A, Wu X, et al. Vesicular stomatitis virus matrix protein inhibits host cell gene expression by targeting the nucleoporin Nup98. Mol Cell. 2000;6(5):1243–52. doi: 10.1016/s1097-2765(00)00120-9 11106761

[95] Mateo M, Reid SP, Leung LW, Basler CF, Volchkov VE. Ebolavirus VP24 Binding to Karyopherins Is Required for Inhibition of Interferon Signaling. J Virol. 2010;84(2):1169–75. doi: 10.1128/JVI.01372-09 19889762 PMC2798383

[96] Reid SP, Leung LW, Hartman AL, Martinez O, Shaw ML, Carbonnelle C, et al. Ebola virus VP24 binds karyopherin alpha1 and blocks STAT1 nuclear accumulation. J Virol. 2006;80(11):5156–67. doi: 10.1128/JVI.02349-05 16698996 PMC1472181

[97] Frieman M, Yount B, Heise M, Kopecky-Bromberg SA, Palese P, Baric RS. Severe acute respiratory syndrome coronavirus ORF6 antagonizes STAT1 function by sequestering nuclear import factors on the rough endoplasmic reticulum/Golgi membrane. J Virol. 2007;81(18):9812–24. doi: 10.1128/JVI.01012-07 17596301 PMC2045396

[98] Kopecky-Bromberg SA, Martínez-Sobrido L, Frieman M, Baric RA, Palese P. Severe acute respiratory syndrome coronavirus open reading frame (ORF) 3b, ORF 6, and nucleocapsid proteins function as interferon antagonists. J Virol. 2007;81(2):548–57. doi: 10.1128/JVI.01782-06 17108024 PMC1797484

[99] Makio T, Zhang K, Love N, Mast FD, Liu X, Elaish M, et al. Molecular Biology of the Cell. 2024;35(5):ar62. doi: 10.1091/mbc.E23-10-0386 PMC11151100 38507240

[100] Miorin L, Kehrer T, Sanchez-Aparicio MT, Zhang K, Cohen P, Patel RS, et al. Proceedings of the National Academy of Sciences of the United States of America. 2020;117(45):28344–54. doi: 10.1073/pnas.2016650117 33097660 PMC7668094

[101] Boulo S, Akarsu H, Ruigrok RWH, Baudin F. Nuclear traffic of influenza virus proteins and ribonucleoprotein complexes. Virus Res. 2007;124(1–2):12–21. doi: 10.1016/j.virusres.2006.09.013 17081640

[102] Dou D, Revol R, Östbye H, Wang H, Daniels R. Influenza A Virus Cell Entry, Replication, Virion Assembly and Movement. Front Immunol. 2018;9:1581. doi: 10.3389/fimmu.2018.01581 30079062 PMC6062596

[103] Eisfeld AJ, Neumann G, Kawaoka Y. At the centre: influenza A virus ribonucleoproteins. Nat Rev Microbiol. 2015;13(1):28–41. doi: 10.1038/nrmicro3367 25417656 PMC5619696

[104] Gao S, Wang S, Cao S, Sun L, Li J, Bi Y, et al. Characteristics of nucleocytoplasmic transport of H1N1 influenza A virus nuclear export protein. J Virol. 2014;88(13):7455–63. doi: 10.1128/JVI.00257-14 24741105 PMC4054460

[105] Hutchinson E, Fodor E. Transport of the influenza virus genome from nucleus to nucleus. Viruses. 2013;5(10):2424–46. doi: 10.3390/v5102424 24104053 PMC3814596

[106] Li J, Yu M, Zheng W, Liu W. Nucleocytoplasmic Shuttling of Influenza A Virus Proteins. Viruses. 2015;7(5):2668–82. doi: 10.3390/v7052668 26008706 PMC4452925

[107] Tessier TM, Dodge MJ, Prusinkiewicz MA, Mymryk JS. Viral Appropriation: Laying Claim to Host Nuclear Transport Machinery. Cells. 2019;8(6):559. doi: 10.3390/cells8060559 31181773 PMC6627039

[108] Watanabe K, Takizawa N, Katoh M, Hoshida K, Kobayashi N, Nagata K. Inhibition of nuclear export of ribonucleoprotein complexes of influenza virus by leptomycin B. Virus Res. 2001;77(1):31–42. doi: 10.1016/s0168-1702(01)00263-5 11451485

[109] Vogel OA, Forwood JK, Leung DW, Amarasinghe GK, Basler CF. Cells. 2023;13(1):71. doi: 10.3390/cells13010071 38201275 PMC10778312

[110] Feng H, Tian H, Wang Y, Zhang Q, Lin N, Liu S. Molecular mechanism underlying selective inhibition of mRNA nuclear export by herpesvirus protein ORF10. Proc Natl Acad Sci USA. 2020;117(43):26719–27. doi: 10.1073/pnas.2007774117 33033226 PMC7604486

[111] Mei M, Cupic A, Miorin L, Ye C, Cagatay T, Zhang K. Proc Natl Acad Sci USA. 2024;121(22):e2314166121. doi: 10.1073/pnas.2314166121 PMC11145185 38768348

[112] Esparza M, Mor A, Niederstrasser H, White K, White A, Zhang K. PLoS Pathog. 2020;16(4):e1008407. doi: 10.1371/journal.ppat.1008407 PMC7117665 32240278

[113] Pereira CF, Read EKC, Wise HM, Amorim MJ, Digard P. J Virol. 2017;91(15):e00528-17. doi: 10.1128/JVI.00528-17 PMC5651720 28515301

[114] Cesaro T, Hayashi Y, Borghese F, Vertommen D, Wavreil F, Michiels T. PKR activity modulation by phosphomimetic mutations of serine residues located three aminoacids upstream of double-stranded RNA binding motifs. Scientific Reports. 2021;11(1):9188. doi: 10.1038/s41598-021-88610-z 33911136 PMC8080564

[115] Nogales A, Schotsaert M, Rathnasinghe R, DeDiego ML, García-Sastre A, Martinez-Sobrido L. Viruses. 2021;13(4):698. doi: 10.3390/v13040698 33920517 PMC8072579

[116] Schickli JH, Flandorfer A, Nakaya T, Martinez-Sobrido L, García-Sastre A, Palese P. Plasmid-only rescue of influenza A virus vaccine candidates. Philos Trans R Soc Lond B Biol Sci. 2001;356(1416):1965–73. doi: 10.1098/rstb.2001.0979 11779399 PMC1088576

[117] Nogales A, Baker SF, Ortiz-Riaño E, Dewhurst S, Topham DJ, Martínez-Sobrido L. J Virol. 2014;88(18):10525–40. doi: 10.1128/JVI.01565-14 24965472 PMC4178899

[118] Stojdl DF, Lichty BD, tenOever BR, Paterson JM, Power AT, Knowles S, et al. VSV strains with defects in their ability to shutdown innate immunity are potent systemic anti-cancer agents. Cancer Cell. 2003;4(4):263–75. doi: 10.1016/s1535-6108(03)00241-1 14585354

[119] Nogales A, Villamayor L, Utrilla-Trigo S, Ortego J, Martinez-Sobrido L, DeDiego ML. Natural Selection of H5N1 Avian Influenza A Viruses with Increased PA-X and NS1 Shutoff Activity. Viruses. 2021;13(9):1760. doi: 10.3390/v13091760 34578340 PMC8472985

[120] Tie HC, Madugula V, Lu L. The development of a single molecule fluorescence standard and its application in estimating the stoichiometry of the nuclear pore complex. Biochem Biophys Res Commun. 2016;478(4):1694–9. doi: 10.1016/j.bbrc.2016.09.005 27613095

[121] Lim KL, Chew KCM, Tan JMM, Wang C, Chung KKK, Zhang Y, et al. Parkin mediates nonclassical, proteasomal-independent ubiquitination of synphilin-1: implications for Lewy body formation. J Neurosci. 2005;25(8):2002–9. doi: 10.1523/JNEUROSCI.4474-04.2005 15728840 PMC6726069

[122] Livak KJ, Schmittgen TD. Analysis of relative gene expression data using real-time quantitative PCR and the 2(-Delta Delta C(T)) Method. Methods. 2001;25(4):402–8. doi: 10.1006/meth.2001.1262 11846609

[123] Kawakami E, Watanabe T, Fujii K, Goto H, Watanabe S, Noda T, et al. Strand-specific real-time RT-PCR for distinguishing influenza vRNA, cRNA, and mRNA. J Virol Methods. 2011;173(1):1–6. doi: 10.1016/j.jviromet.2010.12.014 21185869 PMC3049850

[124] Chen KY, Santos Afonso ED, Enouf V, Isel C, Naffakh N. PLoS Pathog. 2019;15(10):e1008034. doi: 10.1371/journal.ppat.1008034 PMC6776259 31581279

[125] Horio Y, Shichiri M, Isegawa Y. Development of a method for evaluating the mRNA transcription activity of influenza virus RNA-dependent RNA polymerase through real-time reverse transcription polymerase chain reaction. Virol J. 2021;18(1):177. doi: 10.1186/s12985-021-01644-7 34454523 PMC8401337

[126] Navajas R, Ramos-Fernandez A, Herraiz I, Galindo A, Bartha JL, Corrales F, et al. Quantitative proteomic analysis of serum-purified exosomes identifies putative pre-eclampsia-associated biomarkers. Clin Proteomics. 2022;19(1):5. doi: 10.1186/s12014-022-09342-4 35144530 PMC8903615

[127] Wang Y, Zhu W, Levy DE. Nuclear and cytoplasmic mRNA quantification by SYBR green based real-time RT-PCR. Methods. 2006;39(4):356–62. doi: 10.1016/j.ymeth.2006.06.010 16893657

[128] Bhat P, Aksenova V, Gazzara M, Rex EA, Aslam S, Haddad C, et al. Influenza virus mRNAs encode determinants for nuclear export via the cellular TREX-2 complex. Nat Commun. 2023;14(1):2304. doi: 10.1038/s41467-023-37911-0 37085480 PMC10121598

[129] Jahn J, Chaudhry S, Affer M, Pardo A, Pardo G, Taylor J. Preparation of cytoplasmic and nuclear long RNAs from primary and cultured cells. J Vis Exp. 2023;194:64199. doi: 10.3791/64199 37092831

