# Supplementary material for: Tripartite motif-containing 34 (TRIM34) protein interacts with the nucleocytoplasmic transport machinery and negatively modulates antiviral responses
Source: PLoS Pathog. 2026 May 20;22(5):e1014142. doi: 10.1371/journal.ppat.1014142 (PMC13189347; doi:10.1371/journal.ppat.1014142)
Supplement: S1 Appendix — (A) TRIM34 endogenous protein expression was detected in human A549 and 293T mock-infected cells by Western blot using a specific antibody for TRIM34. The anti-GAPDH antibody was used as a loading control. (B) The percentage of infected cells, by means of an immunofluorescence using an anti-NP specific antibody was determined in 293T cells transfected with the empty plasmid, or in cells transfected with the pCAGGs-TRIM34-FLAG plasmid. (C) TRIM34-FLAG protein levels were analyzed by Western blot, using an anti-FLAG antibody, in cells transfected with the empty plasmid or the pCAGGS-TRIM34-FLAG plasmid, at 24 or 48 h after IAV infection. (D) TRIM34 protein levels were analyzed by Western blot, using an anti-TRIM34 antibody, in cells transfected with the empty plasmid or the pCAGGS-TRIM34-FLAG plasmid. (E) TRIM34 mRNA levels were quantified by RT-qPCR in 293T cells transfected with the non-targeted (NT) siRNA or with the TRIM34-specific siRNA, at 24 and 48 h after IAV infection. *p < 0.05 for comparisons between NT siRNA and TRIM34 knocked-down cells, using Student’s t-test with Holm-Šídák correction. (F) TRIM34 endogenous protein expression was detected in human 293T transfected with the non-targeted (NT) siRNA or with the TRIM34-specific siRNA by Western blot using a specific antibody for TRIM34. An anti-GAPDH antibody served as a loading control, and an anti-IAV NP antibody was used to confirm infection. Protein bands in C, D, and F were quantified using the ImageJ software and normalized to the levels of GAPDH expression (numbers below the blots). Molecular weight markers (in kilodaltons) are indicated on the right. Fig B. Human 293T cells were transfected with the pCAGGS plasmid encoding GBP1-FLAG or the empty control plasmid. Co-immunoprecipitation (co-IP) experiments using agarose beads conjugated to an anti-FLAG antibody, to pull down GBP1 were performed. GBP1 was detected by Western blotting using an anti-FLAG antibody in the cellular lysates (input) and after [file ppat.1014142.s001.pdf]

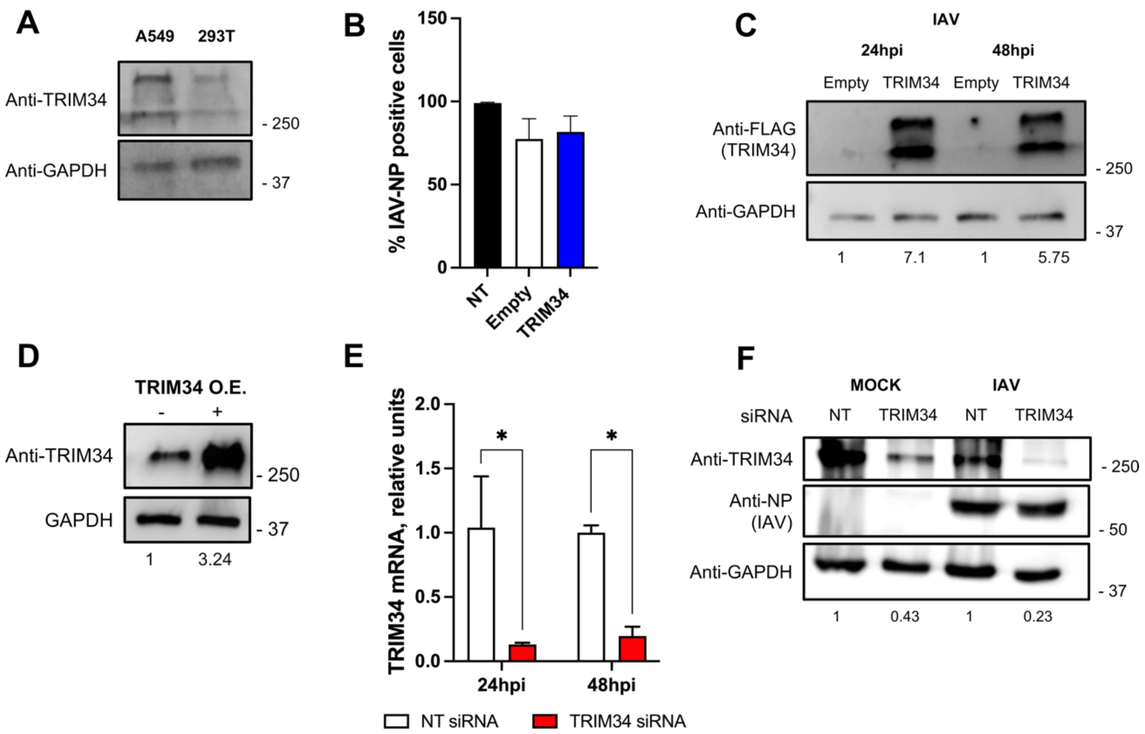

Figure A. (**A**) TRIM34 endogenous protein expression was detected in human A549 and 293T mock-infected cells by Western blot using a specific antibody for TRIM34. The anti-GAPDH antibody was used as a loading control. (**B**) The percentage of infected cells, by means of an immunofluorescence using an anti-NP specific antibody was determined in 293T cells transfected with the empty plasmid, or in cells transfected with the pCAGGs-TRIM34-FLAG plasmid. (**C**) TRIM34-FLAG protein levels were analyzed by Western blot, using an anti-FLAG antibody, in cells transfected with the empty plasmid or the pCAGGS-TRIM34-FLAG plasmid, at 24 or 48 h after IAV infection. **(D)** TRIM34 protein levels were analyzed by Western blot, using an anti-TRIM34 antibody, in cells transfected with the empty plasmid or the pCAGGS-TRIM34-FLAG plasmid. **(E)** TRIM34 mRNA levels were quantified by RT-qPCR in 293T cells transfected with the non-targeted (NT) siRNA or with the TRIM34-specific siRNA, at 24 and 48 h after IAV infection. *$p < 0.05$ for comparisons between NT siRNA and TRIM34 knocked-down cells, using Student's t-test with Holm-Šídák correction. **(F)** TRIM34 endogenous protein expression was detected in human 293T transfected with the non-targeted (NT) siRNA or with the TRIM34-specific siRNA by Western blot using a specific antibody for TRIM34. An anti-GAPDH antibody served as a loading control, and an anti-IAV NP antibody was used to confirm infection. Protein bands in C, D, and F were quantified using the ImageJ software and normalized to the levels of GAPDH expression (numbers below the blots). Molecular weight markers (in kilodaltons) are indicated on the right.

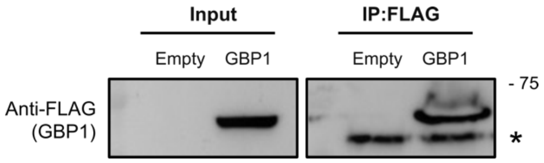

Figure B. Human 293T cells were transfected with the pCAGGS plasmid encoding GBP1-FLAG or the empty control plasmid. Co-immunoprecipitation (co-IP) experiments using agarose beads conjugated to an

anti-FLAG antibody, to pull down GBP1 were performed. GBP1 was detected by Western blotting using an anti-FLAG antibody in the cellular lysates (input) and after the co-IP. Molecular weight markers (in kilodaltons) are indicated on the right. The lower band in the IP blot corresponds to the heavy chain of the antibody used for the co-IP (marked with an *).

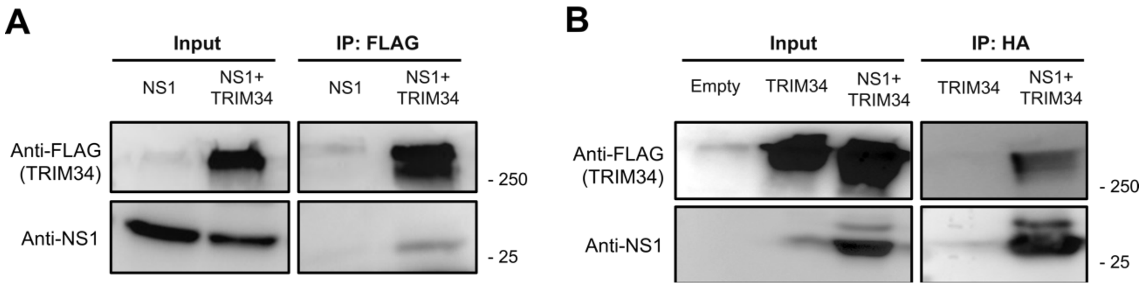

Figure C. Human 293T cells were co-transfected with the pCAGGS plasmid expressing the NS1 fused to an HA tag alone or together with the plasmid encoding TRIM34-FLAG. Co-immunoprecipitation (Co-IP) experiments were performed using **(A)** an anti-FLAG antibody, to pull down TRIM34, **(B)** or using an anti-HA antibody, to pull down NS1. **(A, B)** TRIM34 and NS1 were detected by Western blotting using anti-FLAG or anti-HA antibodies in the cellular lysates (input) and after the co-IP. Molecular weight markers (in kilodaltons) are indicated on the right.

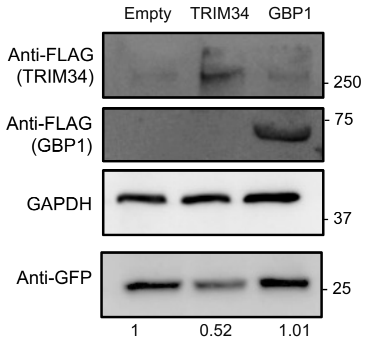

Figure D. Human 293T cells were co-transfected with a plasmid constitutively expressing GFP, and the plasmid encoding TRIM34-FLAG, or the plasmid encoding GBP1-FLAG, or the empty plasmid, as controls. At 24hpt, the levels of GFP expression were analyzed by Western blot using an anti-GFP antibody, and normalized to the levels of GAPDH expression, using the ImageJ software (numbers below the blot). Molecular weight markers (in kilodaltons) are indicated on the right.

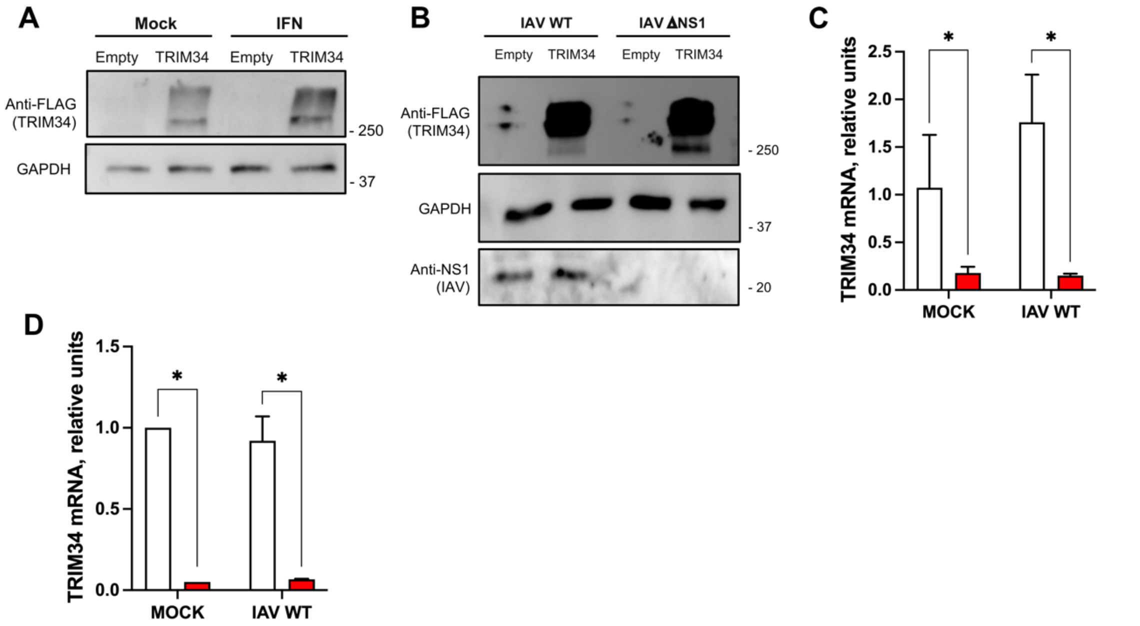

Figure E. (**A**) TRIM34-FLAG protein levels were analyzed by Western blot, using an anti-FLAG antibody, in cells transfected with the empty plasmid or the pCAGGS-TRIM34-FLAG plasmid, at 24h after mock-treatment or treatment with IFN. (**B**) TRIM34-FLAG protein levels were analyzed by Western blot, using an anti-FLAG antibody, in cells transfected with the empty plasmid or the pCAGGS-TRIM34-FLAG plasmid, at 24h after IAV WT or IAV ΔNS1 infection. Human A549 cells **(C)** and or BEAS-2B **(D)** were transfected twice with NT control siRNA or TRIM34 siRNA, 24h apart, for two consecutive days. On day 3, cells were treated with IFN for an additional 24h. **(C, D)** TRIM34 expression was measured by RT-qPCR and mRNA levels were expressed as fold-change (increases) in comparison to mock-treated cells, transfected with the NT siRNA, used as control. *$p < 0.05$ for comparisons between NT siRNA and TRIM34 knocked-down cells, using Student's t-test with Holm-Šídák correction.

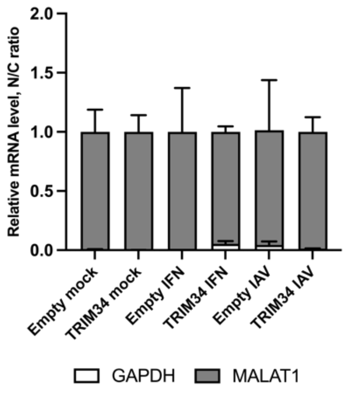

Figure F. Human 293T cells were transfected with the pCAGGS plasmids encoding TRIM34-FLAG or the empty plasmid, as control. At 24h post-transfection, cells were treated with IFN or infected with IAV. At 24hpi, cells were fractionated into cytoplasm and nuclear RNA fractions. Nuclear-to-cytoplasmic (N/C) ratios of GAPDH mRNA (cytoplasmic marker) and MALAT1 long non-coding RNA (nuclear marker) levels were calculated to validate fractionation procedure.
