# Supplementary material for: Tripartite motif-containing 34 (TRIM34) protein interacts with the nucleocytoplasmic transport machinery and negatively modulates antiviral responses
Source: PLoS Pathog. 2026 May 20;22(5):e1014142. doi: 10.1371/journal.ppat.1014142 (PMC13189347; doi:10.1371/journal.ppat.1014142)
Supplement: S2 Appendix — Mass spectrometry identification of TRIM34 protein. Human 293T cells were co-transfected with the pCAGGS plasmid encoding TRIM34-FLAG or the empty plasmid, as control. At 24hpt, cells were mock-infected o infected with IAV (MOI 1). Mass spectrometry identification of protein TRIM34 after FLAG pull-down. Protein accession, description, molecular weight, isoelectric point, sample, group ID, Mascot score, PSMs, peptides, unique peptides, and sequence coverage are shown. Table B. Qualitative proteomic analysis of proteins detected exclusively in TRIM34-FLAG–overexpressing cells. Proteins were identified by mass spectrometry following FLAG pull-down, and only those uniquely present in TRIM34-FLAG–overexpressing cells compared with control cells (transfected with the empty plasmid) are shown. Protein accession, description, molecular weight, isoelectric point, group ID, Mascot score, PSMs, peptides, unique peptides, and sequence coverage are shown, ordered by Mascot score. Table C. Biological process organization of mass spectrometry data. Gene Ontology (GO) enrichment analysis of TRIM34 interactome proteins using DAVID. Shown are enriched GO biological processes in mock and IAV samples with FDR < 0.05, including FDR, -log10(FDR), fold enrichment, genes, and counts, ordered by increasing FDR (most to least significant). Table D. Mass spectrometry identification of GBP1 protein. Human 293T cells were transfected with the pCAGGS plasmid encoding GBP1-FLAG or the empty plasmid, as control. At 24hpt, cells were mock-infected o infected with IAV (MOI 1). Mass spectrometry identification of protein GBP1 after FLAG pull-down. Protein accession, description, molecular weight, isoelectric point, sample, group ID, Mascot score, PSMs, peptides, unique peptides, and sequence coverage are shown. Table E. Qualitative proteomic analysis of proteins detected exclusively in GBP1-FLAG–overexpressing cells. Proteins were identified by mass spectrometry following FLAG pull-down, and only tho [file ppat.1014142.s002.docx]

| **ACCESSION** | **DESCRIPTION** | **MW [KDa]** | **calc. pI** | **SAMPLE** | **Protein Group IDs** | **Score Mascot: Mascot** | **# PSMs** | **# Peptides** | **# Unique Peptides** | **Coverage [%]** |
| --- | --- | --- | --- | --- | --- | --- | --- | --- | --- | --- |
| Q9BYJ4 | **E3 ubiquitin-protein ligase TRIM34**  **OS=Homo sapiens OX=9606 GN=TRIM34 PE=1 SV=2** | 56,8 | 7,44 | MOCK-infected cells | 95 | 1364 | 74 | 29 | 29 | 51 |
|  |  |  |  | IAV-infected cells | 112 | 1210 | 68 | 24 | 24 | 43 |

**Table A. Mass spectrometry identification of TRIM34 protein.** Human 293T cells were co-transfected with the pCAGGS plasmid encoding TRIM34-FLAG or the empty plasmid, as control. At 24hpt, cells were mock-infected o infected with IAV (MOI 1). Mass spectrometry identification of protein TRIM34 after FLAG pull-down. Protein accession, description, molecular weight, isoelectric point, sample, group ID, Mascot score, PSMs, peptides, unique peptides, and sequence coverage are shown.

| **MOCK** | | | | | | | | | | | |
| --- | --- | --- | --- | --- | --- | --- | --- | --- | --- | --- | --- |
| **ACCESSION** | **DESCRIPTION** | **MW [kDa]** | **calc. pI** | **Protein Group IDs** | **Score Mascot: Mascot** | **#PSMs (by Search Engine): Mascot** | **#Peptides (by Search Engine): Mascot** | **#PSMs** | **#Peptides** | **#Unique Peptides** | **Coverage [%]** |
| O95071 | E3 ubiquitin-protein ligase UBR5 OS=Homo sapiens OX=9606 GN=UBR5 PE=1 SV=2 | 309,2 | 5,85 | 836 | 2208 | 54 | 48 | 54 | 48 | 48 | 23 |
| Q9BYJ4 | **E3 ubiquitin-protein ligase TRIM34** OS=Homo sapiens OX=9606 GN=TRIM34 PE=1 SV=2 | 56,8 | 7,44 | 95 | 1364 | 74 | 29 | 74 | 29 | 29 | 51 |
| Q7Z6Z7 | E3 ubiquitin-protein ligase HUWE1 OS=Homo sapiens OX=9606 GN=HUWE1 PE=1 SV=3 | 481,6 | 5,22 | 967 | 1279 | 29 | 28 | 29 | 28 | 28 | 9 |
| Q15154 | Pericentriolar material 1 protein OS=Homo sapiens OX=9606 GN=PCM1 PE=1 SV=5 | 228,4 | 5,02 | 749 | 928 | 20 | 18 | 20 | 18 | 18 | 13 |
| P07814 | Bifunctional glutamate/proline--tRNA ligase OS=Homo sapiens OX=9606 GN=EPRS1 PE=1 SV=5 | 170,5 | 7,33 | 594 | 847 | 19 | 19 | 19 | 19 | 19 | 18 |
| Q92900 | Regulator of nonsense transcripts 1 OS=Homo sapiens OX=9606 GN=UPF1 PE=1 SV=2 | 124,3 | 6,61 | 446 | 740 | 17 | 17 | 17 | 17 | 17 | 17 |
| P07900 | Heat shock protein HSP 90-alpha OS=Homo sapiens OX=9606 GN=HSP90AA1 PE=1 SV=5 | 84,6 | 5,02 | 117 | 733 | 17 | 16 | 17 | 16 | 7 | 25 |
| Q9BQE3 | Tubulin alpha-1C chain OS=Homo sapiens OX=9606 GN=TUBA1C PE=1 SV=1 | 49,9 | 5,1 | 907 | 629 | 17 | 13 | 17 | 13 | 2 | 43 |
| Q9NVI7 | ATPase family AAA domain-containing protein 3A OS=Homo sapiens OX=9606 GN=ATAD3A PE=1 SV=2 | 71,3 | 8,98 | 986 | 579 | 13 | 13 | 13 | 13 | 5 | 24 |
| O14654 | Insulin receptor substrate 4 OS=Homo sapiens OX=9606 GN=IRS4 PE=1 SV=1 | 133,7 | 8,44 | 690 | 566 | 13 | 13 | 13 | 13 | 13 | 14 |
| Q6S8J3 | POTE ankyrin domain family member E OS=Homo sapiens OX=9606 GN=POTEE PE=2 SV=3 | 121,3 | 6,2 | 683 | 559 | 217 | 9 | 217 | 9 | 1 | 9 |
| P57678 | Gem-associated protein 4 OS=Homo sapiens OX=9606 GN=GEMIN4 PE=1 SV=2 | 120 | 6,04 | 420 | 550 | 14 | 13 | 14 | 13 | 13 | 15 |
| P29508 | Serpin B3 OS=Homo sapiens OX=9606 GN=SERPINB3 PE=1 SV=2 | 44,5 | 6,81 | 144 | 437 | 10 | 9 | 10 | 9 | 4 | 29 |
| Q562R1 | Beta-actin-like protein 2 OS=Homo sapiens OX=9606 GN=ACTBL2 PE=1 SV=2 | 42 | 5,59 | 770 | 434 | 188 | 8 | 188 | 8 | 2 | 23 |
| Q9HCE1 | Helicase MOV-10 OS=Homo sapiens OX=9606 GN=MOV10 PE=1 SV=2 | 113,6 | 8,82 | 343 | 410 | 9 | 9 | 9 | 9 | 9 | 10 |
| Q9UJS0 | Electrogenic aspartate/glutamate antiporter SLC25A13, mitochondrial OS=Homo sapiens OX=9606 GN=SLC25A13 PE=1 SV=2 | 74,1 | 8,62 | 710 | 406 | 11 | 11 | 11 | 11 | 6 | 18 |
| P16615 | Sarcoplasmic/endoplasmic reticulum calcium ATPase 2 OS=Homo sapiens OX=9606 GN=ATP2A2 PE=1 SV=1 | 114,7 | 5,34 | 608 | 405 | 11 | 11 | 11 | 11 | 11 | 13 |
| P31689 | DnaJ homolog subfamily A member 1 OS=Homo sapiens OX=9606 GN=DNAJA1 PE=1 SV=2 | 44,8 | 7,08 | 696 | 393 | 11 | 9 | 11 | 9 | 9 | 29 |
| Q9BUF5 | Tubulin beta-6 chain OS=Homo sapiens OX=9606 GN=TUBB6 PE=1 SV=1 | 49,8 | 4,88 | 434 | 381 | 14 | 9 | 14 | 9 | 4 | 22 |
| Q9NNW5 | WD repeat-containing protein 6 OS=Homo sapiens OX=9606 GN=WDR6 PE=1 SV=1 | 121,6 | 6,87 | 1035 | 362 | 11 | 11 | 11 | 11 | 11 | 13 |
| P17812 | CTP synthase 1 OS=Homo sapiens OX=9606 GN=CTPS1 PE=1 SV=2 | 66,6 | 6,46 | 989 | 326 | 8 | 8 | 8 | 8 | 8 | 15 |
| P35658 | Nuclear pore complex protein Nup214 OS=Homo sapiens OX=9606 GN=NUP214 PE=1 SV=2 | 213,5 | 7,47 | 24 | 323 | 8 | 8 | 8 | 8 | 8 | 6 |
| Q92841 | Probable ATP-dependent RNA helicase DDX17 OS=Homo sapiens OX=9606 GN=DDX17 PE=1 SV=2 | 80,2 | 8,27 | 647 | 311 | 6 | 6 | 6 | 6 | 2 | 9 |
| P11177 | Pyruvate dehydrogenase E1 component subunit beta, mitochondrial OS=Homo sapiens OX=9606 GN=PDHB PE=1 SV=3 | 39,2 | 6,65 | 779 | 305 | 6 | 6 | 6 | 6 | 6 | 22 |
| P05455 | Lupus La protein OS=Homo sapiens OX=9606 GN=SSB PE=1 SV=2 | 46,8 | 7,12 | 949 | 287 | 7 | 7 | 7 | 7 | 7 | 20 |
| Q15393 | Splicing factor 3B subunit 3 OS=Homo sapiens OX=9606 GN=SF3B3 PE=1 SV=4 | 135,5 | 5,26 | 98 | 286 | 6 | 6 | 6 | 6 | 6 | 7 |
| O75533 | Splicing factor 3B subunit 1 OS=Homo sapiens OX=9606 GN=SF3B1 PE=1 SV=3 | 145,7 | 7,09 | 320 | 283 | 6 | 6 | 6 | 6 | 6 | 7 |
| Q9Y3F4 | Serine-threonine kinase receptor-associated protein OS=Homo sapiens OX=9606 GN=STRAP PE=1 SV=1 | 38,4 | 5,12 | 787 | 281 | 7 | 7 | 7 | 7 | 7 | 29 |
| Q92621 | Nuclear pore complex protein Nup205 OS=Homo sapiens OX=9606 GN=NUP205 PE=1 SV=3 | 227,8 | 6,19 | 20 | 277 | 8 | 7 | 8 | 7 | 7 | 3 |
| P12235 | ADP/ATP translocase 1 OS=Homo sapiens OX=9606 GN=SLC25A4 PE=1 SV=4 | 33 | 9,76 | 535 | 270 | 9 | 8 | 9 | 8 | 1 | 26 |
| Q09028 | Histone-binding protein RBBP4 OS=Homo sapiens OX=9606 GN=RBBP4 PE=1 SV=3 | 47,6 | 4,89 | 903 | 269 | 5 | 5 | 5 | 5 | 2 | 14 |
| P62191 | 26S proteasome regulatory subunit 4 OS=Homo sapiens OX=9606 GN=PSMC1 PE=1 SV=1 | 49,2 | 6,21 | 971 | 254 | 6 | 6 | 6 | 6 | 5 | 20 |
| P17980 | 26S proteasome regulatory subunit 6A OS=Homo sapiens OX=9606 GN=PSMC3 PE=1 SV=3 | 49,2 | 5,24 | 76 | 251 | 7 | 7 | 7 | 7 | 7 | 24 |
| Q13501 | Sequestosome-1 OS=Homo sapiens OX=9606 GN=SQSTM1 PE=1 SV=1 | 47,7 | 5,22 | 648 | 243 | 5 | 5 | 5 | 5 | 5 | 16 |
| P56192 | Methionine--tRNA ligase, cytoplasmic OS=Homo sapiens OX=9606 GN=MARS1 PE=1 SV=2 | 101,1 | 6,16 | 124 | 240 | 5 | 5 | 5 | 5 | 5 | 7 |
| Q14739 | Delta(14)-sterol reductase LBR OS=Homo sapiens OX=9606 GN=LBR PE=1 SV=2 | 70,7 | 9,36 | 230 | 237 | 4 | 4 | 4 | 4 | 4 | 8 |
| O75746 | Electrogenic aspartate/glutamate antiporter SLC25A12, mitochondrial OS=Homo sapiens OX=9606 GN=SLC25A12 PE=1 SV=2 | 74,7 | 8,38 | 439 | 236 | 6 | 6 | 6 | 6 | 1 | 9 |
| P60891 | Ribose-phosphate pyrophosphokinase 1 OS=Homo sapiens OX=9606 GN=PRPS1 PE=1 SV=2 | 34,8 | 6,98 | 715 | 230 | 5 | 5 | 5 | 5 | 5 | 17 |
| Q9UHI6 | Probable ATP-dependent RNA helicase DDX20 OS=Homo sapiens OX=9606 GN=DDX20 PE=1 SV=2 | 92,2 | 6,95 | 184 | 230 | 6 | 6 | 6 | 6 | 6 | 8 |
| Q99567 | Nuclear pore complex protein Nup88 OS=Homo sapiens OX=9606 GN=NUP88 PE=1 SV=2 | 83,5 | 5,69 | 57 | 227 | 5 | 4 | 5 | 4 | 4 | 9 |
| P38919 | Eukaryotic initiation factor 4A-III OS=Homo sapiens OX=9606 GN=EIF4A3 PE=1 SV=4 | 46,8 | 6,73 | 831 | 223 | 6 | 6 | 6 | 6 | 6 | 14 |
| P52292 | Importin subunit alpha-1 OS=Homo sapiens OX=9606 GN=KPNA2 PE=1 SV=1 | 57,8 | 5,4 | 254 | 222 | 4 | 4 | 4 | 4 | 4 | 10 |
| Q14257 | Reticulocalbin-2 OS=Homo sapiens OX=9606 GN=RCN2 PE=1 SV=1 | 36,9 | 4,4 | 447 | 222 | 4 | 4 | 4 | 4 | 4 | 24 |
| Q96N67 | Dedicator of cytokinesis protein 7 OS=Homo sapiens OX=9606 GN=DOCK7 PE=1 SV=4 | 242,4 | 6,8 | 344 | 216 | 8 | 7 | 8 | 7 | 7 | 4 |
| P23458 | Tyrosine-protein kinase JAK1 OS=Homo sapiens OX=9606 GN=JAK1 PE=1 SV=2 | 133,2 | 7,55 | 777 | 215 | 5 | 5 | 5 | 5 | 5 | 5 |
| P51398 | 28S ribosomal protein S29, mitochondrial OS=Homo sapiens OX=9606 GN=DAP3 PE=1 SV=1 | 45,5 | 8,88 | 312 | 215 | 6 | 6 | 6 | 6 | 6 | 18 |
| P30837 | Aldehyde dehydrogenase X, mitochondrial OS=Homo sapiens OX=9606 GN=ALDH1B1 PE=1 SV=4 | 57,2 | 6,99 | 981 | 212 | 6 | 6 | 6 | 6 | 6 | 15 |
| Q13155 | Aminoacyl tRNA synthase complex-interacting multifunctional protein 2 OS=Homo sapiens OX=9606 GN=AIMP2 PE=1 SV=2 | 35,3 | 8,22 | 620 | 211 | 5 | 4 | 5 | 4 | 4 | 20 |
| P17858 | ATP-dependent 6-phosphofructokinase, liver type OS=Homo sapiens OX=9606 GN=PFKL PE=1 SV=6 | 85 | 7,5 | 636 | 207 | 5 | 5 | 5 | 5 | 4 | 10 |
| O60832 | H/ACA ribonucleoprotein complex subunit DKC1 OS=Homo sapiens OX=9606 GN=DKC1 PE=1 SV=3 | 57,6 | 9,42 | 432 | 204 | 6 | 6 | 6 | 6 | 6 | 16 |
| O14980 | Exportin-1 OS=Homo sapiens OX=9606 GN=XPO1 PE=1 SV=1 | 123,3 | 6,06 | 466 | 202 | 6 | 6 | 6 | 6 | 6 | 7 |
| P37198 | Nuclear pore glycoprotein p62 OS=Homo sapiens OX=9606 GN=NUP62 PE=1 SV=3 | 53,2 | 5,31 | 704 | 199 | 5 | 5 | 5 | 5 | 5 | 16 |
| P54136 | Arginine--tRNA ligase, cytoplasmic OS=Homo sapiens OX=9606 GN=RARS1 PE=1 SV=2 | 75,3 | 6,68 | 630 | 198 | 6 | 6 | 6 | 6 | 6 | 10 |
| Q9UKM9 | RNA-binding protein Raly OS=Homo sapiens OX=9606 GN=RALY PE=1 SV=1 | 32,4 | 9,17 | 905 | 197 | 6 | 6 | 6 | 6 | 6 | 23 |
| P20700 | Lamin-B1 OS=Homo sapiens OX=9606 GN=LMNB1 PE=1 SV=2 | 66,4 | 5,16 | 346 | 194 | 5 | 5 | 5 | 5 | 4 | 9 |
| Q92522 | Histone H1.10 OS=Homo sapiens OX=9606 GN=H1-10 PE=1 SV=1 | 22,5 | 10,76 | 173 | 192 | 4 | 4 | 4 | 4 | 4 | 18 |
| P43686 | 26S proteasome regulatory subunit 6B OS=Homo sapiens OX=9606 GN=PSMC4 PE=1 SV=2 | 47,3 | 5,21 | 830 | 186 | 6 | 6 | 6 | 6 | 6 | 20 |
| P63173 | 60S ribosomal protein L38 OS=Homo sapiens OX=9606 GN=RPL38 PE=1 SV=2 | 8,2 | 10,1 | 590 | 186 | 4 | 4 | 4 | 4 | 4 | 50 |
| O75592 | E3 ubiquitin-protein ligase MYCBP2 OS=Homo sapiens OX=9606 GN=MYCBP2 PE=1 SV=4 | 513,3 | 7,02 | 541 | 184 | 4 | 4 | 4 | 4 | 4 | 1 |
| P22695 | Cytochrome b-c1 complex subunit 2, mitochondrial OS=Homo sapiens OX=9606 GN=UQCRC2 PE=1 SV=3 | 48,4 | 8,63 | 563 | 184 | 4 | 4 | 4 | 4 | 4 | 12 |
| Q92616 | eIF-2-alpha kinase activator GCN1 OS=Homo sapiens OX=9606 GN=GCN1 PE=1 SV=7 | 292,5 | 7,43 | 180 | 184 | 5 | 5 | 5 | 5 | 5 | 2 |
| Q9UBX3 | Mitochondrial dicarboxylate carrier OS=Homo sapiens OX=9606 GN=SLC25A10 PE=1 SV=2 | 31,3 | 9,54 | 646 | 181 | 6 | 6 | 6 | 6 | 6 | 26 |
| Q9BVP2 | Guanine nucleotide-binding protein-like 3 OS=Homo sapiens OX=9606 GN=GNL3 PE=1 SV=2 | 62 | 9,16 | 50 | 179 | 6 | 6 | 6 | 6 | 6 | 12 |
| P46379 | Large proline-rich protein BAG6 OS=Homo sapiens OX=9606 GN=BAG6 PE=1 SV=2 | 119,3 | 5,6 | 1008 | 174 | 4 | 4 | 4 | 4 | 4 | 4 |
| Q3ZCQ8 | Mitochondrial import inner membrane translocase subunit TIM50 OS=Homo sapiens OX=9606 GN=TIMM50 PE=1 SV=2 | 39,6 | 8,37 | 775 | 171 | 4 | 4 | 4 | 4 | 4 | 14 |
| P02786 | Transferrin receptor protein 1 OS=Homo sapiens OX=9606 GN=TFRC PE=1 SV=2 | 84,8 | 6,61 | 950 | 163 | 4 | 4 | 4 | 4 | 4 | 6 |
| P35606 | Coatomer subunit beta' OS=Homo sapiens OX=9606 GN=COPB2 PE=1 SV=2 | 102,4 | 5,27 | 888 | 163 | 5 | 5 | 5 | 5 | 5 | 7 |
| Q08J23 | RNA cytosine C(5)-methyltransferase NSUN2 OS=Homo sapiens OX=9606 GN=NSUN2 PE=1 SV=2 | 86,4 | 6,77 | 217 | 163 | 4 | 4 | 4 | 4 | 4 | 7 |
| P33991 | DNA replication licensing factor MCM4 OS=Homo sapiens OX=9606 GN=MCM4 PE=1 SV=5 | 96,5 | 6,74 | 436 | 161 | 3 | 3 | 3 | 3 | 3 | 5 |
| Q9Y2X3 | Nucleolar protein 58 OS=Homo sapiens OX=9606 GN=NOP58 PE=1 SV=1 | 59,5 | 8,92 | 128 | 160 | 3 | 3 | 3 | 3 | 3 | 8 |
| Q7L2E3 | ATP-dependent RNA helicase DHX30 OS=Homo sapiens OX=9606 GN=DHX30 PE=1 SV=1 | 133,9 | 8,78 | 597 | 159 | 4 | 4 | 4 | 4 | 4 | 4 |
| Q8N1F7 | Nuclear pore complex protein Nup93 OS=Homo sapiens OX=9606 GN=NUP93 PE=1 SV=2 | 93,4 | 5,72 | 1021 | 159 | 5 | 5 | 5 | 5 | 5 | 6 |
| P61289 | Proteasome activator complex subunit 3 OS=Homo sapiens OX=9606 GN=PSME3 PE=1 SV=1 | 29,5 | 5,95 | 998 | 158 | 3 | 3 | 3 | 3 | 3 | 14 |
| P04844 | Dolichyl-diphosphooligosaccharide--protein glycosyltransferase subunit 2 OS=Homo sapiens OX=9606 GN=RPN2 PE=1 SV=3 | 69,2 | 5,69 | 362 | 155 | 4 | 4 | 4 | 4 | 4 | 10 |
| Q13813 | Spectrin alpha chain, non-erythrocytic 1 OS=Homo sapiens OX=9606 GN=SPTAN1 PE=1 SV=3 | 284,4 | 5,35 | 514 | 152 | 4 | 4 | 4 | 4 | 4 | 2 |
| Q9HB71 | Calcyclin-binding protein OS=Homo sapiens OX=9606 GN=CACYBP PE=1 SV=2 | 26,2 | 8,25 | 613 | 152 | 4 | 4 | 4 | 4 | 4 | 23 |
| P36873 | Serine/threonine-protein phosphatase PP1-gamma catalytic subunit OS=Homo sapiens OX=9606 GN=PPP1CC PE=1 SV=1 | 37 | 6,54 | 437 | 149 | 4 | 4 | 4 | 4 | 2 | 15 |
| Q9Y3Z3 | Deoxynucleoside triphosphate triphosphohydrolase SAMHD1 OS=Homo sapiens OX=9606 GN=SAMHD1 PE=1 SV=2 | 72,2 | 7,14 | 101 | 148 | 3 | 3 | 3 | 3 | 3 | 8 |
| P82650 | 28S ribosomal protein S22, mitochondrial OS=Homo sapiens OX=9606 GN=MRPS22 PE=1 SV=1 | 41,3 | 7,9 | 136 | 147 | 4 | 3 | 4 | 3 | 3 | 13 |
| P49720 | Proteasome subunit beta type-3 OS=Homo sapiens OX=9606 GN=PSMB3 PE=1 SV=2 | 22,9 | 6,55 | 133 | 146 | 4 | 3 | 4 | 3 | 3 | 23 |
| P63241 | Eukaryotic translation initiation factor 5A-1 OS=Homo sapiens OX=9606 GN=EIF5A PE=1 SV=2 | 16,8 | 5,24 | 658 | 146 | 4 | 3 | 4 | 3 | 3 | 34 |
| Q9NUD5 | Zinc finger CCHC domain-containing protein 3 OS=Homo sapiens OX=9606 GN=ZCCHC3 PE=1 SV=2 | 43,5 | 8,53 | 612 | 146 | 3 | 3 | 3 | 3 | 3 | 10 |
| Q9BUJ2 | Heterogeneous nuclear ribonucleoprotein U-like protein 1 OS=Homo sapiens OX=9606 GN=HNRNPUL1 PE=1 SV=2 | 95,7 | 6,92 | 407 | 145 | 5 | 5 | 5 | 5 | 5 | 9 |
| Q7L5D6 | Golgi to ER traffic protein 4 homolog OS=Homo sapiens OX=9606 GN=GET4 PE=1 SV=1 | 36,5 | 5,41 | 163 | 143 | 3 | 3 | 3 | 3 | 3 | 11 |
| A8K2U0 | Alpha-2-macroglobulin-like protein 1 OS=Homo sapiens OX=9606 GN=A2ML1 PE=1 SV=3 | 161 | 5,73 | 653 | 142 | 3 | 3 | 3 | 3 | 3 | 2 |
| P52597 | Heterogeneous nuclear ribonucleoprotein F OS=Homo sapiens OX=9606 GN=HNRNPF PE=1 SV=3 | 45,6 | 5,58 | 662 | 141 | 4 | 4 | 4 | 4 | 3 | 19 |
| Q86VP6 | Cullin-associated NEDD8-dissociated protein 1 OS=Homo sapiens OX=9606 GN=CAND1 PE=1 SV=2 | 136,3 | 5,78 | 37 | 139 | 3 | 3 | 3 | 3 | 3 | 3 |
| P04083 | Annexin A1 OS=Homo sapiens OX=9606 GN=ANXA1 PE=1 SV=2 | 38,7 | 7,02 | 795 | 137 | 2 | 2 | 2 | 2 | 2 | 8 |
| P55786 | Puromycin-sensitive aminopeptidase OS=Homo sapiens OX=9606 GN=NPEPPS PE=1 SV=2 | 103,2 | 5,72 | 769 | 137 | 5 | 4 | 5 | 4 | 4 | 5 |
| Q9H444 | Charged multivesicular body protein 4b OS=Homo sapiens OX=9606 GN=CHMP4B PE=1 SV=1 | 24,9 | 4,82 | 41 | 137 | 3 | 3 | 3 | 3 | 3 | 15 |
| Q0ZGT2 | Nexilin OS=Homo sapiens OX=9606 GN=NEXN PE=1 SV=1 | 80,6 | 5,33 | 731 | 135 | 3 | 3 | 3 | 3 | 3 | 5 |
| Q9Y295 | Developmentally-regulated GTP-binding protein 1 OS=Homo sapiens OX=9606 GN=DRG1 PE=1 SV=1 | 40,5 | 8,9 | 363 | 134 | 2 | 2 | 2 | 2 | 2 | 8 |
| Q9Y5V3 | Melanoma-associated antigen D1 OS=Homo sapiens OX=9606 GN=MAGED1 PE=1 SV=3 | 86,1 | 5,83 | 585 | 132 | 4 | 4 | 4 | 4 | 4 | 4 |
| Q9NYK5 | 39S ribosomal protein L39, mitochondrial OS=Homo sapiens OX=9606 GN=MRPL39 PE=1 SV=3 | 38,7 | 7,65 | 641 | 130 | 4 | 3 | 4 | 3 | 3 | 12 |
| P48729 | Casein kinase I isoform alpha OS=Homo sapiens OX=9606 GN=CSNK1A1 PE=1 SV=2 | 38,9 | 9,57 | 752 | 129 | 3 | 3 | 3 | 3 | 3 | 10 |
| Q9BQG0 | Myb-binding protein 1A OS=Homo sapiens OX=9606 GN=MYBBP1A PE=1 SV=2 | 148,8 | 9,28 | 842 | 129 | 4 | 4 | 4 | 4 | 4 | 3 |
| O15212 | Prefoldin subunit 6 OS=Homo sapiens OX=9606 GN=PFDN6 PE=1 SV=1 | 14,6 | 8,88 | 972 | 127 | 3 | 3 | 3 | 3 | 3 | 23 |
| O95831 | Apoptosis-inducing factor 1, mitochondrial OS=Homo sapiens OX=9606 GN=AIFM1 PE=1 SV=1 | 66,9 | 8,95 | 204 | 127 | 4 | 4 | 4 | 4 | 4 | 9 |
| Q92665 | 28S ribosomal protein S31, mitochondrial OS=Homo sapiens OX=9606 GN=MRPS31 PE=1 SV=3 | 45,3 | 9,29 | 821 | 126 | 2 | 2 | 2 | 2 | 2 | 8 |
| P15927 | Replication protein A 32 kDa subunit OS=Homo sapiens OX=9606 GN=RPA2 PE=1 SV=1 | 29,2 | 6,15 | 1007 | 124 | 2 | 2 | 2 | 2 | 2 | 13 |
| P21912 | Succinate dehydrogenase [ubiquinone] iron-sulfur subunit, mitochondrial OS=Homo sapiens OX=9606 GN=SDHB PE=1 SV=3 | 31,6 | 8,76 | 36 | 124 | 4 | 4 | 4 | 4 | 4 | 16 |
| P60866 | 40S ribosomal protein S20 OS=Homo sapiens OX=9606 GN=RPS20 PE=1 SV=1 | 13,4 | 9,94 | 212 | 124 | 3 | 3 | 3 | 3 | 3 | 18 |
| Q96I24 | Far upstream element-binding protein 3 OS=Homo sapiens OX=9606 GN=FUBP3 PE=1 SV=2 | 61,6 | 8,38 | 339 | 124 | 4 | 4 | 4 | 4 | 4 | 10 |
| Q9NVP1 | ATP-dependent RNA helicase DDX18 OS=Homo sapiens OX=9606 GN=DDX18 PE=1 SV=2 | 75,4 | 9,5 | 132 | 124 | 3 | 3 | 3 | 3 | 3 | 6 |
| Q9Y5S9 | RNA-binding protein 8A OS=Homo sapiens OX=9606 GN=RBM8A PE=1 SV=1 | 19,9 | 5,72 | 262 | 124 | 3 | 3 | 3 | 3 | 3 | 32 |
| Q14498 | RNA-binding protein 39 OS=Homo sapiens OX=9606 GN=RBM39 PE=1 SV=2 | 59,3 | 10,1 | 722 | 123 | 3 | 3 | 3 | 3 | 3 | 8 |
| O76021 | Ribosomal L1 domain-containing protein 1 OS=Homo sapiens OX=9606 GN=RSL1D1 PE=1 SV=3 | 54,9 | 10,13 | 449 | 121 | 3 | 3 | 3 | 3 | 3 | 8 |
| P40616 | ADP-ribosylation factor-like protein 1 OS=Homo sapiens OX=9606 GN=ARL1 PE=1 SV=1 | 20,4 | 5,72 | 978 | 121 | 3 | 3 | 3 | 3 | 3 | 17 |
| P30153 | Serine/threonine-protein phosphatase 2A 65 kDa regulatory subunit A alpha isoform OS=Homo sapiens OX=9606 GN=PPP2R1A PE=1 SV=4 | 65,3 | 5,11 | 492 | 120 | 2 | 2 | 2 | 2 | 2 | 4 |
| P46977 | Dolichyl-diphosphooligosaccharide--protein glycosyltransferase subunit STT3A OS=Homo sapiens OX=9606 GN=STT3A PE=1 SV=2 | 80,5 | 8,07 | 12 | 120 | 4 | 3 | 4 | 3 | 3 | 4 |
| Q13547 | Histone deacetylase 1 OS=Homo sapiens OX=9606 GN=HDAC1 PE=1 SV=1 | 55,1 | 5,48 | 378 | 120 | 2 | 2 | 2 | 2 | 2 | 8 |
| O75190 | DnaJ homolog subfamily B member 6 OS=Homo sapiens OX=9606 GN=DNAJB6 PE=1 SV=2 | 36,1 | 9,16 | 356 | 118 | 3 | 3 | 3 | 3 | 2 | 12 |
| Q9Y5M8 | Signal recognition particle receptor subunit beta OS=Homo sapiens OX=9606 GN=SRPRB PE=1 SV=3 | 29,7 | 9,04 | 154 | 118 | 3 | 3 | 3 | 3 | 3 | 16 |
| P55084 | Trifunctional enzyme subunit beta, mitochondrial OS=Homo sapiens OX=9606 GN=HADHB PE=1 SV=3 | 51,3 | 9,41 | 781 | 117 | 3 | 3 | 3 | 3 | 3 | 10 |
| O94906 | Pre-mRNA-processing factor 6 OS=Homo sapiens OX=9606 GN=PRPF6 PE=1 SV=1 | 106,9 | 8,25 | 540 | 116 | 3 | 3 | 3 | 3 | 3 | 3 |
| P78406 | mRNA export factor RAE1 OS=Homo sapiens OX=9606 GN=RAE1 PE=1 SV=1 | 40,9 | 7,83 | 774 | 115 | 3 | 3 | 3 | 3 | 3 | 11 |
| P08621 | U1 small nuclear ribonucleoprotein 70 kDa OS=Homo sapiens OX=9606 GN=SNRNP70 PE=1 SV=2 | 51,5 | 9,94 | 42 | 114 | 3 | 3 | 3 | 3 | 3 | 8 |
| O94905 | Erlin-2 OS=Homo sapiens OX=9606 GN=ERLIN2 PE=1 SV=1 | 37,8 | 5,62 | 606 | 113 | 2 | 2 | 2 | 2 | 2 | 7 |
| P62258 | 14-3-3 protein epsilon OS=Homo sapiens OX=9606 GN=YWHAE PE=1 SV=1 | 29,2 | 4,74 | 988 | 113 | 3 | 3 | 3 | 3 | 2 | 16 |
| O00487 | 26S proteasome non-ATPase regulatory subunit 14 OS=Homo sapiens OX=9606 GN=PSMD14 PE=1 SV=1 | 34,6 | 6,52 | 761 | 112 | 4 | 4 | 4 | 4 | 4 | 20 |
| P57088 | Transmembrane protein 33 OS=Homo sapiens OX=9606 GN=TMEM33 PE=1 SV=2 | 28 | 9,7 | 235 | 111 | 2 | 2 | 2 | 2 | 2 | 9 |
| Q01813 | ATP-dependent 6-phosphofructokinase, platelet type OS=Homo sapiens OX=9606 GN=PFKP PE=1 SV=2 | 85,5 | 7,55 | 399 | 111 | 3 | 3 | 3 | 3 | 2 | 5 |
| Q9H0E2 | Toll-interacting protein OS=Homo sapiens OX=9606 GN=TOLLIP PE=1 SV=1 | 30,3 | 5,97 | 607 | 110 | 2 | 2 | 2 | 2 | 2 | 8 |
| Q9P035 | Very-long-chain (3R)-3-hydroxyacyl-CoA dehydratase 3 OS=Homo sapiens OX=9606 GN=HACD3 PE=1 SV=2 | 43,1 | 8,94 | 56 | 110 | 3 | 3 | 3 | 3 | 3 | 10 |
| Q02880 | DNA topoisomerase 2-beta OS=Homo sapiens OX=9606 GN=TOP2B PE=1 SV=3 | 183,2 | 8 | 126 | 109 | 3 | 3 | 3 | 3 | 3 | 2 |
| Q14677 | Clathrin interactor 1 OS=Homo sapiens OX=9606 GN=CLINT1 PE=1 SV=1 | 68,2 | 6,42 | 364 | 109 | 3 | 3 | 3 | 3 | 3 | 7 |
| Q8NI60 | Atypical kinase COQ8A, mitochondrial OS=Homo sapiens OX=9606 GN=COQ8A PE=1 SV=1 | 71,9 | 6,99 | 176 | 109 | 3 | 3 | 3 | 3 | 3 | 5 |
| P52434 | DNA-directed RNA polymerases I, II, and III subunit RPABC3 OS=Homo sapiens OX=9606 GN=POLR2H PE=1 SV=4 | 17,1 | 4,68 | 980 | 108 | 2 | 2 | 2 | 2 | 2 | 16 |
| Q96DH6 | RNA-binding protein Musashi homolog 2 OS=Homo sapiens OX=9606 GN=MSI2 PE=1 SV=1 | 35,2 | 8,48 | 164 | 108 | 3 | 3 | 3 | 3 | 2 | 9 |
| P07305 | Histone H1.0 OS=Homo sapiens OX=9606 GN=H1-0 PE=1 SV=3 | 20,9 | 10,84 | 33 | 107 | 2 | 2 | 2 | 2 | 2 | 13 |
| P51571 | Translocon-associated protein subunit delta OS=Homo sapiens OX=9606 GN=SSR4 PE=1 SV=1 | 19 | 6,15 | 350 | 103 | 2 | 2 | 2 | 2 | 2 | 14 |
| Q16637 | Survival motor neuron protein OS=Homo sapiens OX=9606 GN=SMN1 PE=1 SV=1 | 31,8 | 6,55 | 329 | 103 | 3 | 2 | 3 | 2 | 2 | 11 |
| Q9H0A0 | RNA cytidine acetyltransferase OS=Homo sapiens OX=9606 GN=NAT10 PE=1 SV=2 | 115,7 | 8,27 | 109 | 103 | 2 | 2 | 2 | 2 | 2 | 2 |
| O60884 | DnaJ homolog subfamily A member 2 OS=Homo sapiens OX=9606 GN=DNAJA2 PE=1 SV=1 | 45,7 | 6,48 | 850 | 102 | 2 | 2 | 2 | 2 | 2 | 7 |
| P22061 | Protein-L-isoaspartate(D-aspartate) O-methyltransferase OS=Homo sapiens OX=9606 GN=PCMT1 PE=1 SV=4 | 24,6 | 7,21 | 189 | 102 | 2 | 2 | 2 | 2 | 2 | 16 |
| Q13835 | Plakophilin-1 OS=Homo sapiens OX=9606 GN=PKP1 PE=1 SV=2 | 82,8 | 9,13 | 510 | 102 | 2 | 2 | 2 | 2 | 2 | 3 |
| O95433 | Activator of 90 kDa heat shock protein ATPase homolog 1 OS=Homo sapiens OX=9606 GN=AHSA1 PE=1 SV=1 | 38,3 | 5,53 | 657 | 101 | 2 | 2 | 2 | 2 | 2 | 9 |
| P60900 | Proteasome subunit alpha type-6 OS=Homo sapiens OX=9606 GN=PSMA6 PE=1 SV=1 | 27,4 | 6,76 | 550 | 101 | 3 | 3 | 3 | 3 | 3 | 14 |
| Q16540 | 39S ribosomal protein L23, mitochondrial OS=Homo sapiens OX=9606 GN=MRPL23 PE=1 SV=1 | 17,8 | 9,69 | 706 | 101 | 1 | 1 | 1 | 1 | 1 | 8 |
| Q9Y383 | Putative RNA-binding protein Luc7-like 2 OS=Homo sapiens OX=9606 GN=LUC7L2 PE=1 SV=2 | 46,5 | 10,01 | 193 | 101 | 2 | 2 | 2 | 2 | 2 | 6 |
| O14880 | Microsomal glutathione S-transferase 3 OS=Homo sapiens OX=9606 GN=MGST3 PE=1 SV=1 | 16,5 | 9,38 | 555 | 100 | 2 | 2 | 2 | 2 | 2 | 18 |
| O95299 | NADH dehydrogenase [ubiquinone] 1 alpha subcomplex subunit 10, mitochondrial OS=Homo sapiens OX=9606 GN=NDUFA10 PE=1 SV=1 | 40,7 | 8,48 | 405 | 100 | 3 | 3 | 3 | 3 | 3 | 9 |
| Q04637 | Eukaryotic translation initiation factor 4 gamma 1 OS=Homo sapiens OX=9606 GN=EIF4G1 PE=1 SV=4 | 175,4 | 5,33 | 200 | 100 | 2 | 2 | 2 | 2 | 2 | 1 |
| P62995 | Transformer-2 protein homolog beta OS=Homo sapiens OX=9606 GN=TRA2B PE=1 SV=1 | 33,6 | 11,25 | 1022 | 99 | 2 | 2 | 2 | 2 | 2 | 10 |
| P40938 | Replication factor C subunit 3 OS=Homo sapiens OX=9606 GN=RFC3 PE=1 SV=2 | 40,5 | 8,34 | 595 | 98 | 2 | 2 | 2 | 2 | 2 | 6 |
| Q9UNM6 | 26S proteasome non-ATPase regulatory subunit 13 OS=Homo sapiens OX=9606 GN=PSMD13 PE=1 SV=2 | 42,9 | 5,81 | 739 | 98 | 2 | 2 | 2 | 2 | 2 | 6 |
| Q8TDN6 | Ribosome biogenesis protein BRX1 homolog OS=Homo sapiens OX=9606 GN=BRIX1 PE=1 SV=2 | 41,4 | 9,92 | 331 | 97 | 2 | 2 | 2 | 2 | 2 | 6 |
| Q13162 | Peroxiredoxin-4 OS=Homo sapiens OX=9606 GN=PRDX4 PE=1 SV=1 | 30,5 | 6,29 | 654 | 96 | 2 | 2 | 2 | 2 | 1 | 7 |
| Q5JPE7 | BOS complex subunit NOMO2 OS=Homo sapiens OX=9606 GN=NOMO2 PE=1 SV=1 | 139,4 | 5,76 | 214 | 96 | 3 | 3 | 3 | 3 | 3 | 3 |
| Q96IX5 | ATP synthase membrane subunit K, mitochondrial OS=Homo sapiens OX=9606 GN=ATP5MK PE=1 SV=1 | 6,5 | 9,76 | 829 | 96 | 1 | 1 | 1 | 1 | 1 | 26 |
| Q9UHB9 | Signal recognition particle subunit SRP68 OS=Homo sapiens OX=9606 GN=SRP68 PE=1 SV=2 | 70,7 | 8,56 | 351 | 96 | 3 | 3 | 3 | 3 | 3 | 5 |
| Q9BYD6 | 39S ribosomal protein L1, mitochondrial OS=Homo sapiens OX=9606 GN=MRPL1 PE=1 SV=2 | 36,9 | 8,78 | 340 | 95 | 2 | 2 | 2 | 2 | 2 | 6 |
| Q9UKD2 | mRNA turnover protein 4 homolog OS=Homo sapiens OX=9606 GN=MRTO4 PE=1 SV=2 | 27,5 | 8,29 | 328 | 95 | 3 | 3 | 3 | 3 | 3 | 13 |
| O43837 | Isocitrate dehydrogenase [NAD] subunit beta, mitochondrial OS=Homo sapiens OX=9606 GN=IDH3B PE=1 SV=2 | 42,2 | 8,46 | 443 | 94 | 2 | 2 | 2 | 2 | 2 | 8 |
| O96019 | Actin-like protein 6A OS=Homo sapiens OX=9606 GN=ACTL6A PE=1 SV=1 | 47,4 | 5,6 | 725 | 93 | 2 | 2 | 2 | 2 | 2 | 7 |
| P82673 | 28S ribosomal protein S35, mitochondrial OS=Homo sapiens OX=9606 GN=MRPS35 PE=1 SV=1 | 36,8 | 8,24 | 368 | 92 | 3 | 3 | 3 | 3 | 3 | 11 |
| P62314 | Small nuclear ribonucleoprotein Sm D1 OS=Homo sapiens OX=9606 GN=SNRPD1 PE=1 SV=1 | 13,3 | 11,56 | 237 | 91 | 2 | 2 | 2 | 2 | 2 | 29 |
| Q15645 | Pachytene checkpoint protein 2 homolog OS=Homo sapiens OX=9606 GN=TRIP13 PE=1 SV=2 | 48,5 | 6,09 | 870 | 91 | 2 | 2 | 2 | 2 | 2 | 5 |
| P25686 | DnaJ homolog subfamily B member 2 OS=Homo sapiens OX=9606 GN=DNAJB2 PE=1 SV=3 | 35,6 | 5,95 | 377 | 90 | 2 | 2 | 2 | 2 | 1 | 7 |
| P39656 | Dolichyl-diphosphooligosaccharide--protein glycosyltransferase 48 kDa subunit OS=Homo sapiens OX=9606 GN=DDOST PE=1 SV=4 | 50,8 | 6,55 | 160 | 89 | 3 | 3 | 3 | 3 | 3 | 10 |
| P28072 | Proteasome subunit beta type-6 OS=Homo sapiens OX=9606 GN=PSMB6 PE=1 SV=4 | 25,3 | 4,92 | 233 | 88 | 2 | 2 | 2 | 2 | 2 | 9 |
| P28288 | ATP-binding cassette sub-family D member 3 OS=Homo sapiens OX=9606 GN=ABCD3 PE=1 SV=1 | 75,4 | 9,36 | 179 | 88 | 3 | 3 | 3 | 3 | 3 | 6 |
| Q15020 | Squamous cell carcinoma antigen recognized by T-cells 3 OS=Homo sapiens OX=9606 GN=SART3 PE=1 SV=1 | 109,9 | 5,57 | 263 | 88 | 2 | 2 | 2 | 2 | 2 | 2 |
| Q9Y285 | Phenylalanine--tRNA ligase alpha subunit OS=Homo sapiens OX=9606 GN=FARSA PE=1 SV=3 | 57,5 | 7,8 | 1020 | 88 | 3 | 3 | 3 | 3 | 3 | 6 |
| O43242 | 26S proteasome non-ATPase regulatory subunit 3 OS=Homo sapiens OX=9606 GN=PSMD3 PE=1 SV=2 | 60,9 | 8,44 | 728 | 87 | 3 | 3 | 3 | 3 | 3 | 7 |
| P55735 | Protein SEC13 homolog OS=Homo sapiens OX=9606 GN=SEC13 PE=1 SV=3 | 35,5 | 5,48 | 637 | 87 | 1 | 1 | 1 | 1 | 1 | 8 |
| Q9BRS2 | Serine/threonine-protein kinase RIO1 OS=Homo sapiens OX=9606 GN=RIOK1 PE=1 SV=2 | 65,5 | 6,19 | 557 | 87 | 2 | 2 | 2 | 2 | 2 | 3 |
| P53999 | Activated RNA polymerase II transcriptional coactivator p15 OS=Homo sapiens OX=9606 GN=SUB1 PE=1 SV=3 | 14,4 | 9,6 | 511 | 86 | 2 | 2 | 2 | 2 | 2 | 16 |
| Q12904 | Aminoacyl tRNA synthase complex-interacting multifunctional protein 1 OS=Homo sapiens OX=9606 GN=AIMP1 PE=1 SV=2 | 34,3 | 8,43 | 308 | 86 | 2 | 2 | 2 | 2 | 2 | 11 |
| Q2M1P5 | Kinesin-like protein KIF7 OS=Homo sapiens OX=9606 GN=KIF7 PE=1 SV=2 | 150,5 | 6,79 | 185 | 86 | 3 | 3 | 3 | 3 | 3 | 3 |
| Q53GQ0 | Very-long-chain 3-oxoacyl-CoA reductase OS=Homo sapiens OX=9606 GN=HSD17B12 PE=1 SV=2 | 34,3 | 9,32 | 338 | 86 | 2 | 2 | 2 | 2 | 2 | 6 |
| Q9H0U3 | Magnesium transporter protein 1 OS=Homo sapiens OX=9606 GN=MAGT1 PE=1 SV=1 | 38 | 9,63 | 85 | 85 | 2 | 2 | 2 | 2 | 2 | 6 |
| Q96GC5 | 39S ribosomal protein L48, mitochondrial OS=Homo sapiens OX=9606 GN=MRPL48 PE=1 SV=2 | 23,9 | 8,98 | 226 | 84 | 2 | 2 | 2 | 2 | 2 | 12 |
| P07339 | Cathepsin D OS=Homo sapiens OX=9606 GN=CTSD PE=1 SV=1 | 44,5 | 6,54 | 990 | 83 | 2 | 2 | 2 | 2 | 2 | 6 |
| P24666 | Low molecular weight phosphotyrosine protein phosphatase OS=Homo sapiens OX=9606 GN=ACP1 PE=1 SV=3 | 18 | 6,74 | 805 | 83 | 1 | 1 | 1 | 1 | 1 | 11 |
| Q8IWS0 | PHD finger protein 6 OS=Homo sapiens OX=9606 GN=PHF6 PE=1 SV=1 | 41,3 | 8,68 | 502 | 83 | 2 | 2 | 2 | 2 | 2 | 8 |
| Q9Y2W2 | WW domain-binding protein 11 OS=Homo sapiens OX=9606 GN=WBP11 PE=1 SV=1 | 70 | 8,38 | 758 | 83 | 3 | 3 | 3 | 3 | 3 | 7 |
| Q14247 | Src substrate cortactin OS=Homo sapiens OX=9606 GN=CTTN PE=1 SV=2 | 61,5 | 5,4 | 178 | 80 | 2 | 2 | 2 | 2 | 2 | 4 |
| Q9Y3B7 | 39S ribosomal protein L11, mitochondrial OS=Homo sapiens OX=9606 GN=MRPL11 PE=1 SV=1 | 20,7 | 9,91 | 929 | 80 | 2 | 2 | 2 | 2 | 2 | 13 |
| O96005 | Putative lipid scramblase CLPTM1 OS=Homo sapiens OX=9606 GN=CLPTM1 PE=1 SV=1 | 76 | 6,3 | 427 | 79 | 2 | 2 | 2 | 2 | 2 | 2 |
| P28290 | Protein ITPRID2 OS=Homo sapiens OX=9606 GN=ITPRID2 PE=1 SV=3 | 138,3 | 5,19 | 159 | 79 | 2 | 2 | 2 | 2 | 2 | 2 |
| P42167 | Lamina-associated polypeptide 2, isoforms beta/gamma OS=Homo sapiens OX=9606 GN=TMPO PE=1 SV=2 | 50,6 | 9,38 | 552 | 79 | 2 | 2 | 2 | 2 | 2 | 6 |
| P68400 | Casein kinase II subunit alpha OS=Homo sapiens OX=9606 GN=CSNK2A1 PE=1 SV=1 | 45,1 | 7,74 | 782 | 79 | 2 | 2 | 2 | 2 | 2 | 7 |
| Q969V3 | BOS complex subunit NCLN OS=Homo sapiens OX=9606 GN=NCLN PE=1 SV=2 | 62,9 | 6,89 | 797 | 79 | 2 | 2 | 2 | 2 | 2 | 4 |
| Q99615 | DnaJ homolog subfamily C member 7 OS=Homo sapiens OX=9606 GN=DNAJC7 PE=1 SV=2 | 56,4 | 6,96 | 151 | 79 | 2 | 2 | 2 | 2 | 2 | 5 |
| Q9H9B4 | Sideroflexin-1 OS=Homo sapiens OX=9606 GN=SFXN1 PE=1 SV=4 | 35,6 | 9,07 | 721 | 79 | 2 | 2 | 2 | 2 | 2 | 8 |
| Q14318 | Peptidyl-prolyl cis-trans isomerase FKBP8 OS=Homo sapiens OX=9606 GN=FKBP8 PE=1 SV=2 | 44,5 | 4,84 | 267 | 78 | 1 | 1 | 1 | 1 | 1 | 4 |
| Q6P1L8 | 39S ribosomal protein L14, mitochondrial OS=Homo sapiens OX=9606 GN=MRPL14 PE=1 SV=1 | 15,9 | 10,24 | 886 | 78 | 3 | 2 | 3 | 2 | 2 | 17 |
| Q969G3 | SWI/SNF-related matrix-associated actin-dependent regulator of chromatin subfamily E member 1 OS=Homo sapiens OX=9606 GN=SMARCE1 PE=1 SV=2 | 46,6 | 4,88 | 280 | 77 | 1 | 1 | 1 | 1 | 1 | 3 |
| Q9UBB4 | Ataxin-10 OS=Homo sapiens OX=9606 GN=ATXN10 PE=1 SV=1 | 53,5 | 5,25 | 542 | 76 | 2 | 2 | 2 | 2 | 2 | 5 |
| O75643 | U5 small nuclear ribonucleoprotein 200 kDa helicase OS=Homo sapiens OX=9606 GN=SNRNP200 PE=1 SV=2 | 244,4 | 6,06 | 894 | 74 | 2 | 2 | 2 | 2 | 2 | 1 |
| O95373 | Importin-7 OS=Homo sapiens OX=9606 GN=IPO7 PE=1 SV=1 | 119,4 | 4,82 | 811 | 74 | 2 | 2 | 2 | 2 | 2 | 3 |
| Q93008 | Probable ubiquitin carboxyl-terminal hydrolase FAF-X OS=Homo sapiens OX=9606 GN=USP9X PE=1 SV=4 | 290,3 | 5,8 | 481 | 74 | 2 | 2 | 2 | 2 | 2 | 1 |
| Q9BXW9 | Fanconi anemia group D2 protein OS=Homo sapiens OX=9606 GN=FANCD2 PE=1 SV=2 | 164 | 5,88 | 485 | 74 | 2 | 2 | 2 | 2 | 2 | 2 |
| Q14204 | Cytoplasmic dynein 1 heavy chain 1 OS=Homo sapiens OX=9606 GN=DYNC1H1 PE=1 SV=5 | 532,1 | 6,4 | 384 | 73 | 2 | 2 | 2 | 2 | 2 | 1 |
| Q6IBS0 | Twinfilin-2 OS=Homo sapiens OX=9606 GN=TWF2 PE=1 SV=2 | 39,5 | 6,84 | 712 | 73 | 2 | 2 | 2 | 2 | 1 | 7 |
| P61962 | DDB1- and CUL4-associated factor 7 OS=Homo sapiens OX=9606 GN=DCAF7 PE=1 SV=1 | 38,9 | 5,52 | 198 | 72 | 1 | 1 | 1 | 1 | 1 | 4 |
| Q9Y221 | 60S ribosome subunit biogenesis protein NIP7 homolog OS=Homo sapiens OX=9606 GN=NIP7 PE=1 SV=1 | 20,4 | 8,51 | 391 | 72 | 1 | 1 | 1 | 1 | 1 | 6 |
| P24390 | ER lumen protein-retaining receptor 1 OS=Homo sapiens OX=9606 GN=KDELR1 PE=1 SV=1 | 24,5 | 8,62 | 488 | 71 | 2 | 2 | 2 | 2 | 2 | 14 |
| Q8IZL8 | Proline-, glutamic acid- and leucine-rich protein 1 OS=Homo sapiens OX=9606 GN=PELP1 PE=1 SV=2 | 119,6 | 4,34 | 265 | 71 | 2 | 2 | 2 | 2 | 2 | 2 |
| Q9UG63 | ATP-binding cassette sub-family F member 2 OS=Homo sapiens OX=9606 GN=ABCF2 PE=1 SV=2 | 71,2 | 7,37 | 679 | 71 | 2 | 2 | 2 | 2 | 2 | 3 |
| P38159 | RNA-binding motif protein, X chromosome OS=Homo sapiens OX=9606 GN=RBMX PE=1 SV=3 | 42,3 | 10,05 | 247 | 70 | 1 | 1 | 1 | 1 | 1 | 3 |
| P28070 | Proteasome subunit beta type-4 OS=Homo sapiens OX=9606 GN=PSMB4 PE=1 SV=4 | 29,2 | 5,97 | 635 | 69 | 1 | 1 | 1 | 1 | 1 | 4 |
| P62861 | FAU ubiquitin-like and ribosomal protein S30 OS=Homo sapiens OX=9606 GN=FAU PE=1 SV=2 | 14,4 | 10,17 | 625 | 69 | 2 | 2 | 2 | 2 | 2 | 8 |
| Q7KZN9 | Cytochrome c oxidase assembly protein COX15 homolog OS=Homo sapiens OX=9606 GN=COX15 PE=1 SV=1 | 46 | 9,82 | 426 | 69 | 2 | 2 | 2 | 2 | 2 | 6 |
| Q9H9J2 | 39S ribosomal protein L44, mitochondrial OS=Homo sapiens OX=9606 GN=MRPL44 PE=1 SV=1 | 37,5 | 8,4 | 689 | 69 | 2 | 2 | 2 | 2 | 2 | 5 |
| Q9Y2W1 | Thyroid hormone receptor-associated protein 3 OS=Homo sapiens OX=9606 GN=THRAP3 PE=1 SV=2 | 108,6 | 10,15 | 477 | 69 | 2 | 2 | 2 | 2 | 2 | 3 |
| O15269 | Serine palmitoyltransferase 1 OS=Homo sapiens OX=9606 GN=SPTLC1 PE=1 SV=1 | 52,7 | 6,01 | 798 | 68 | 1 | 1 | 1 | 1 | 1 | 3 |
| P25787 | Proteasome subunit alpha type-2 OS=Homo sapiens OX=9606 GN=PSMA2 PE=1 SV=2 | 25,9 | 7,43 | 175 | 68 | 1 | 1 | 1 | 1 | 1 | 6 |
| Q14151 | Scaffold attachment factor B2 OS=Homo sapiens OX=9606 GN=SAFB2 PE=1 SV=1 | 107,4 | 6,16 | 583 | 68 | 2 | 2 | 2 | 2 | 2 | 2 |
| Q14197 | Peptidyl-tRNA hydrolase ICT1, mitochondrial OS=Homo sapiens OX=9606 GN=MRPL58 PE=1 SV=1 | 23,6 | 10,07 | 358 | 68 | 2 | 2 | 2 | 2 | 2 | 8 |
| Q8IXM3 | 39S ribosomal protein L41, mitochondrial OS=Homo sapiens OX=9606 GN=MRPL41 PE=1 SV=1 | 15,4 | 9,57 | 832 | 68 | 2 | 2 | 2 | 2 | 2 | 16 |
| Q96AG4 | Leucine-rich repeat-containing protein 59 OS=Homo sapiens OX=9606 GN=LRRC59 PE=1 SV=1 | 34,9 | 9,57 | 307 | 68 | 2 | 2 | 2 | 2 | 2 | 8 |
| Q9BQ67 | Glutamate-rich WD repeat-containing protein 1 OS=Homo sapiens OX=9606 GN=GRWD1 PE=1 SV=1 | 49,4 | 4,92 | 9 | 68 | 1 | 1 | 1 | 1 | 1 | 4 |
| Q9UNE7 | E3 ubiquitin-protein ligase CHIP OS=Homo sapiens OX=9606 GN=STUB1 PE=1 SV=2 | 34,8 | 5,87 | 948 | 68 | 3 | 3 | 3 | 3 | 3 | 13 |
| P43243 | Matrin-3 OS=Homo sapiens OX=9606 GN=MATR3 PE=1 SV=2 | 94,6 | 6,25 | 579 | 67 | 3 | 2 | 3 | 2 | 2 | 3 |
| P52926 | High mobility group protein HMGI-C OS=Homo sapiens OX=9606 GN=HMGA2 PE=1 SV=1 | 11,8 | 10,62 | 413 | 67 | 2 | 2 | 2 | 2 | 2 | 21 |
| P60228 | Eukaryotic translation initiation factor 3 subunit E OS=Homo sapiens OX=9606 GN=EIF3E PE=1 SV=1 | 52,2 | 6,04 | 414 | 67 | 2 | 2 | 2 | 2 | 2 | 7 |
| Q9Y6M1 | Insulin-like growth factor 2 mRNA-binding protein 2 OS=Homo sapiens OX=9606 GN=IGF2BP2 PE=1 SV=2 | 66,1 | 8,46 | 65 | 67 | 2 | 2 | 2 | 2 | 1 | 4 |
| O43347 | RNA-binding protein Musashi homolog 1 OS=Homo sapiens OX=9606 GN=MSI1 PE=1 SV=1 | 39,1 | 7,85 | 1001 | 66 | 2 | 2 | 2 | 2 | 1 | 5 |
| P14373 | Zinc finger protein RFP OS=Homo sapiens OX=9606 GN=TRIM27 PE=1 SV=1 | 58,5 | 6,21 | 366 | 66 | 2 | 2 | 2 | 2 | 2 | 4 |
| Q9NZT1 | Calmodulin-like protein 5 OS=Homo sapiens OX=9606 GN=CALML5 PE=1 SV=2 | 15,9 | 4,44 | 656 | 66 | 1 | 1 | 1 | 1 | 1 | 10 |
| Q01650 | Large neutral amino acids transporter small subunit 1 OS=Homo sapiens OX=9606 GN=SLC7A5 PE=1 SV=2 | 55 | 7,72 | 354 | 65 | 1 | 1 | 1 | 1 | 1 | 3 |
| Q9BYD3 | 39S ribosomal protein L4, mitochondrial OS=Homo sapiens OX=9606 GN=MRPL4 PE=1 SV=1 | 34,9 | 9,72 | 125 | 65 | 2 | 2 | 2 | 2 | 2 | 9 |
| Q14683 | Structural maintenance of chromosomes protein 1A OS=Homo sapiens OX=9606 GN=SMC1A PE=1 SV=2 | 143,1 | 7,64 | 49 | 64 | 2 | 2 | 2 | 2 | 2 | 2 |
| Q7Z7F7 | 39S ribosomal protein L55, mitochondrial OS=Homo sapiens OX=9606 GN=MRPL55 PE=1 SV=1 | 15,1 | 11,15 | 232 | 64 | 2 | 2 | 2 | 2 | 2 | 19 |
| Q8WXD5 | Gem-associated protein 6 OS=Homo sapiens OX=9606 GN=GEMIN6 PE=1 SV=1 | 18,8 | 5,12 | 292 | 64 | 3 | 2 | 3 | 2 | 2 | 13 |
| O00232 | 26S proteasome non-ATPase regulatory subunit 12 OS=Homo sapiens OX=9606 GN=PSMD12 PE=1 SV=3 | 52,9 | 7,65 | 361 | 63 | 2 | 2 | 2 | 2 | 2 | 4 |
| O14617 | AP-3 complex subunit delta-1 OS=Homo sapiens OX=9606 GN=AP3D1 PE=1 SV=1 | 130,1 | 8,48 | 194 | 63 | 2 | 2 | 2 | 2 | 2 | 2 |
| P46821 | Microtubule-associated protein 1B OS=Homo sapiens OX=9606 GN=MAP1B PE=1 SV=2 | 270,5 | 4,81 | 170 | 63 | 3 | 2 | 3 | 2 | 2 | 1 |
| P82921 | 28S ribosomal protein S21, mitochondrial OS=Homo sapiens OX=9606 GN=MRPS21 PE=1 SV=3 | 10,7 | 9,92 | 778 | 63 | 1 | 1 | 1 | 1 | 1 | 14 |
| Q07955 | Serine/arginine-rich splicing factor 1 OS=Homo sapiens OX=9606 GN=SRSF1 PE=1 SV=2 | 27,7 | 10,36 | 785 | 63 | 2 | 2 | 2 | 2 | 2 | 8 |
| Q8TEM1 | Nuclear pore membrane glycoprotein 210 OS=Homo sapiens OX=9606 GN=NUP210 PE=1 SV=3 | 205 | 6,81 | 626 | 63 | 2 | 2 | 2 | 2 | 2 | 1 |
| O00148 | ATP-dependent RNA helicase DDX39A OS=Homo sapiens OX=9606 GN=DDX39A PE=1 SV=2 | 49,1 | 5,68 | 349 | 62 | 2 | 2 | 2 | 2 | 2 | 7 |
| Q15758 | Neutral amino acid transporter B(0) OS=Homo sapiens OX=9606 GN=SLC1A5 PE=1 SV=2 | 56,6 | 5,48 | 129 | 62 | 1 | 1 | 1 | 1 | 1 | 2 |
| P60468 | Protein transport protein Sec61 subunit beta OS=Homo sapiens OX=9606 GN=SEC61B PE=1 SV=2 | 10 | 11,56 | 509 | 61 | 2 | 2 | 2 | 2 | 2 | 26 |
| P63220 | 40S ribosomal protein S21 OS=Homo sapiens OX=9606 GN=RPS21 PE=1 SV=1 | 9,1 | 8,5 | 120 | 61 | 1 | 1 | 1 | 1 | 1 | 18 |
| Q14684 | Ribosomal RNA processing protein 1 homolog B OS=Homo sapiens OX=9606 GN=RRP1B PE=1 SV=3 | 84,4 | 9,76 | 750 | 61 | 2 | 2 | 2 | 2 | 2 | 5 |
| Q96EY7 | Pentatricopeptide repeat domain-containing protein 3, mitochondrial OS=Homo sapiens OX=9606 GN=PTCD3 PE=1 SV=3 | 78,5 | 6,42 | 593 | 61 | 2 | 1 | 2 | 1 | 1 | 2 |
| Q9BZE4 | GTP-binding protein 4 OS=Homo sapiens OX=9606 GN=GTPBP4 PE=1 SV=3 | 73,9 | 9,5 | 91 | 61 | 2 | 2 | 2 | 2 | 2 | 4 |
| P08237 | ATP-dependent 6-phosphofructokinase, muscle type OS=Homo sapiens OX=9606 GN=PFKM PE=1 SV=2 | 85,1 | 7,99 | 692 | 60 | 2 | 2 | 2 | 2 | 2 | 3 |
| P35250 | Replication factor C subunit 2 OS=Homo sapiens OX=9606 GN=RFC2 PE=1 SV=3 | 39,1 | 6,44 | 818 | 60 | 2 | 2 | 2 | 2 | 2 | 6 |
| Q7Z2W4 | Zinc finger CCCH-type antiviral protein 1 OS=Homo sapiens OX=9606 GN=ZC3HAV1 PE=1 SV=3 | 101,4 | 8,4 | 55 | 59 | 1 | 1 | 1 | 1 | 1 | 2 |
| Q86YT6 | E3 ubiquitin-protein ligase MIB1 OS=Homo sapiens OX=9606 GN=MIB1 PE=1 SV=1 | 110,1 | 6,92 | 531 | 59 | 2 | 2 | 2 | 2 | 2 | 2 |
| Q9BRP8 | Partner of Y14 and mago OS=Homo sapiens OX=9606 GN=PYM1 PE=1 SV=1 | 22,6 | 9,45 | 444 | 59 | 2 | 2 | 2 | 2 | 2 | 14 |
| Q9H6S0 | 3'-5' RNA helicase YTHDC2 OS=Homo sapiens OX=9606 GN=YTHDC2 PE=1 SV=2 | 160,1 | 8,4 | 1009 | 59 | 1 | 1 | 1 | 1 | 1 | 1 |
| Q9NUL3 | Double-stranded RNA-binding protein Staufen homolog 2 OS=Homo sapiens OX=9606 GN=STAU2 PE=1 SV=2 | 62,6 | 9,61 | 922 | 59 | 2 | 2 | 2 | 2 | 2 | 4 |
| O75663 | TIP41-like protein OS=Homo sapiens OX=9606 GN=TIPRL PE=1 SV=2 | 31,4 | 5,91 | 424 | 58 | 2 | 2 | 2 | 2 | 2 | 8 |
| P09651 | Heterogeneous nuclear ribonucleoprotein A1 OS=Homo sapiens OX=9606 GN=HNRNPA1 PE=1 SV=5 | 38,7 | 9,13 | 993 | 58 | 2 | 2 | 2 | 2 | 1 | 5 |
| Q9BV38 | WD repeat-containing protein 18 OS=Homo sapiens OX=9606 GN=WDR18 PE=1 SV=2 | 47,4 | 6,7 | 624 | 58 | 1 | 1 | 1 | 1 | 1 | 3 |
| Q9H307 | Pinin OS=Homo sapiens OX=9606 GN=PNN PE=1 SV=5 | 81,6 | 7,14 | 868 | 58 | 2 | 2 | 2 | 2 | 2 | 4 |
| A5YKK6 | CCR4-NOT transcription complex subunit 1 OS=Homo sapiens OX=9606 GN=CNOT1 PE=1 SV=2 | 266,8 | 7,11 | 319 | 57 | 2 | 2 | 2 | 2 | 2 | 1 |
| P24539 | ATP synthase F(0) complex subunit B1, mitochondrial OS=Homo sapiens OX=9606 GN=ATP5PB PE=1 SV=2 | 28,9 | 9,36 | 25 | 57 | 2 | 2 | 2 | 2 | 2 | 11 |
| Q14676 | Mediator of DNA damage checkpoint protein 1 OS=Homo sapiens OX=9606 GN=MDC1 PE=1 SV=3 | 226,5 | 5,47 | 139 | 57 | 2 | 2 | 2 | 2 | 2 | 2 |
| Q92769 | Histone deacetylase 2 OS=Homo sapiens OX=9606 GN=HDAC2 PE=1 SV=2 | 55,3 | 5,91 | 788 | 57 | 1 | 1 | 1 | 1 | 1 | 4 |
| Q96A65 | Exocyst complex component 4 OS=Homo sapiens OX=9606 GN=EXOC4 PE=1 SV=1 | 110,4 | 6,49 | 393 | 57 | 1 | 1 | 1 | 1 | 1 | 1 |
| Q15003 | Condensin complex subunit 2 OS=Homo sapiens OX=9606 GN=NCAPH PE=1 SV=3 | 82,5 | 5,06 | 921 | 56 | 1 | 1 | 1 | 1 | 1 | 1 |
| Q9UQ35 | Serine/arginine repetitive matrix protein 2 OS=Homo sapiens OX=9606 GN=SRRM2 PE=1 SV=2 | 299,4 | 12,06 | 453 | 56 | 2 | 2 | 2 | 2 | 2 | 1 |
| Q9UQ80 | Proliferation-associated protein 2G4 OS=Homo sapiens OX=9606 GN=PA2G4 PE=1 SV=3 | 43,8 | 6,55 | 765 | 56 | 2 | 2 | 2 | 2 | 2 | 7 |
| Q9NZB2 | Constitutive coactivator of PPAR-gamma-like protein 1 OS=Homo sapiens OX=9606 GN=FAM120A PE=1 SV=2 | 121,8 | 8,88 | 822 | 55 | 2 | 2 | 2 | 2 | 2 | 2 |
| Q9UL15 | BAG family molecular chaperone regulator 5 OS=Homo sapiens OX=9606 GN=BAG5 PE=1 SV=1 | 51,2 | 6,05 | 152 | 55 | 2 | 2 | 2 | 2 | 2 | 7 |
| Q9UL25 | Ras-related protein Rab-21 OS=Homo sapiens OX=9606 GN=RAB21 PE=1 SV=3 | 24,3 | 7,94 | 615 | 55 | 1 | 1 | 1 | 1 | 1 | 7 |
| P51570 | Galactokinase OS=Homo sapiens OX=9606 GN=GALK1 PE=1 SV=1 | 42,2 | 6,46 | 735 | 54 | 2 | 2 | 2 | 2 | 2 | 8 |
| Q14694 | Ubiquitin carboxyl-terminal hydrolase 10 OS=Homo sapiens OX=9606 GN=USP10 PE=1 SV=2 | 87,1 | 5,31 | 445 | 54 | 2 | 2 | 2 | 2 | 2 | 3 |
| Q92973 | Transportin-1 OS=Homo sapiens OX=9606 GN=TNPO1 PE=1 SV=2 | 102,3 | 4,98 | 417 | 54 | 1 | 1 | 1 | 1 | 1 | 1 |
| Q9NRZ9 | Lymphoid-specific helicase OS=Homo sapiens OX=9606 GN=HELLS PE=1 SV=1 | 97 | 7,93 | 87 | 54 | 2 | 2 | 2 | 2 | 2 | 3 |
| O00541 | Pescadillo homolog OS=Homo sapiens OX=9606 GN=PES1 PE=1 SV=1 | 68 | 7,33 | 737 | 53 | 2 | 2 | 2 | 2 | 2 | 3 |
| Q2NL82 | Pre-rRNA-processing protein TSR1 homolog OS=Homo sapiens OX=9606 GN=TSR1 PE=1 SV=1 | 91,8 | 7,42 | 169 | 53 | 2 | 2 | 2 | 2 | 2 | 3 |
| O75380 | NADH dehydrogenase [ubiquinone] iron-sulfur protein 6, mitochondrial OS=Homo sapiens OX=9606 GN=NDUFS6 PE=1 SV=1 | 13,7 | 8,28 | 359 | 52 | 1 | 1 | 1 | 1 | 1 | 12 |
| P14174 | Macrophage migration inhibitory factor OS=Homo sapiens OX=9606 GN=MIF PE=1 SV=4 | 12,5 | 7,88 | 92 | 52 | 1 | 1 | 1 | 1 | 1 | 8 |
| Q9NTJ3 | Structural maintenance of chromosomes protein 4 OS=Homo sapiens OX=9606 GN=SMC4 PE=1 SV=2 | 147,1 | 6,79 | 302 | 52 | 2 | 2 | 2 | 2 | 2 | 1 |
| Q9P2E9 | Ribosome-binding protein 1 OS=Homo sapiens OX=9606 GN=RRBP1 PE=1 SV=5 | 152,4 | 8,6 | 764 | 52 | 1 | 1 | 1 | 1 | 1 | 1 |
| Q9UDY4 | DnaJ homolog subfamily B member 4 OS=Homo sapiens OX=9606 GN=DNAJB4 PE=1 SV=1 | 37,8 | 8,5 | 854 | 52 | 1 | 1 | 1 | 1 | 1 | 4 |
| O15027 | Protein transport protein Sec16A OS=Homo sapiens OX=9606 GN=SEC16A PE=1 SV=4 | 251,7 | 5,8 | 655 | 51 | 2 | 2 | 2 | 2 | 2 | 1 |
| P43490 | Nicotinamide phosphoribosyltransferase OS=Homo sapiens OX=9606 GN=NAMPT PE=1 SV=1 | 55,5 | 7,15 | 794 | 51 | 2 | 2 | 2 | 2 | 2 | 3 |
| Q13724 | Mannosyl-oligosaccharide glucosidase OS=Homo sapiens OX=9606 GN=MOGS PE=1 SV=5 | 91,9 | 8,9 | 602 | 51 | 1 | 1 | 1 | 1 | 1 | 1 |
| Q15427 | Splicing factor 3B subunit 4 OS=Homo sapiens OX=9606 GN=SF3B4 PE=1 SV=1 | 44,4 | 8,56 | 773 | 51 | 2 | 1 | 2 | 1 | 1 | 3 |
| Q9NZ01 | Very-long-chain enoyl-CoA reductase OS=Homo sapiens OX=9606 GN=TECR PE=1 SV=1 | 36 | 9,45 | 945 | 51 | 2 | 2 | 2 | 2 | 2 | 6 |
| O75489 | NADH dehydrogenase [ubiquinone] iron-sulfur protein 3, mitochondrial OS=Homo sapiens OX=9606 GN=NDUFS3 PE=1 SV=1 | 30,2 | 7,5 | 524 | 50 | 2 | 1 | 2 | 1 | 1 | 5 |
| P60953 | Cell division control protein 42 homolog OS=Homo sapiens OX=9606 GN=CDC42 PE=1 SV=2 | 21,2 | 6,55 | 494 | 50 | 2 | 2 | 2 | 2 | 2 | 15 |
| O14893 | Gem-associated protein 2 OS=Homo sapiens OX=9606 GN=GEMIN2 PE=1 SV=1 | 31,6 | 5,58 | 573 | 49 | 1 | 1 | 1 | 1 | 1 | 3 |
| P28074 | Proteasome subunit beta type-5 OS=Homo sapiens OX=9606 GN=PSMB5 PE=1 SV=3 | 28,5 | 6,92 | 61 | 49 | 1 | 1 | 1 | 1 | 1 | 5 |
| P46777 | 60S ribosomal protein L5 OS=Homo sapiens OX=9606 GN=RPL5 PE=1 SV=3 | 34,3 | 9,72 | 703 | 49 | 1 | 1 | 1 | 1 | 1 | 4 |
| P63167 | Dynein light chain 1, cytoplasmic OS=Homo sapiens OX=9606 GN=DYNLL1 PE=1 SV=1 | 10,4 | 7,4 | 932 | 49 | 1 | 1 | 1 | 1 | 1 | 25 |
| Q8N684 | Cleavage and polyadenylation specificity factor subunit 7 OS=Homo sapiens OX=9606 GN=CPSF7 PE=1 SV=1 | 52 | 8 | 223 | 49 | 1 | 1 | 1 | 1 | 1 | 2 |
| Q9P0J0 | NADH dehydrogenase [ubiquinone] 1 alpha subcomplex subunit 13 OS=Homo sapiens OX=9606 GN=NDUFA13 PE=1 SV=3 | 16,7 | 8,43 | 670 | 49 | 2 | 2 | 2 | 2 | 2 | 16 |
| Q96P70 | Importin-9 OS=Homo sapiens OX=9606 GN=IPO9 PE=1 SV=3 | 115,9 | 4,81 | 865 | 48 | 1 | 1 | 1 | 1 | 1 | 2 |
| Q9UDW1 | Cytochrome b-c1 complex subunit 9 OS=Homo sapiens OX=9606 GN=UQCR10 PE=1 SV=3 | 7,3 | 9,47 | 792 | 48 | 1 | 1 | 1 | 1 | 1 | 11 |
| Q9UN86 | Ras GTPase-activating protein-binding protein 2 OS=Homo sapiens OX=9606 GN=G3BP2 PE=1 SV=2 | 54,1 | 5,55 | 191 | 48 | 1 | 1 | 1 | 1 | 1 | 3 |
| O43592 | Exportin-T OS=Homo sapiens OX=9606 GN=XPOT PE=1 SV=2 | 109,9 | 5,39 | 925 | 47 | 1 | 1 | 1 | 1 | 1 | 1 |
| P00403 | Cytochrome c oxidase subunit 2 OS=Homo sapiens OX=9606 GN=MT-CO2 PE=1 SV=1 | 25,5 | 4,82 | 258 | 47 | 1 | 1 | 1 | 1 | 1 | 3 |
| Q9BXW7 | Haloacid dehalogenase-like hydrolase domain-containing 5 OS=Homo sapiens OX=9606 GN=HDHD5 PE=1 SV=1 | 46,3 | 8,13 | 153 | 47 | 1 | 1 | 1 | 1 | 1 | 4 |
| Q9NW13 | RNA-binding protein 28 OS=Homo sapiens OX=9606 GN=RBM28 PE=1 SV=3 | 85,7 | 9,22 | 671 | 47 | 1 | 1 | 1 | 1 | 1 | 2 |
| O95347 | Structural maintenance of chromosomes protein 2 OS=Homo sapiens OX=9606 GN=SMC2 PE=1 SV=2 | 135,6 | 8,43 | 955 | 46 | 2 | 2 | 2 | 2 | 2 | 2 |
| P19404 | NADH dehydrogenase [ubiquinone] flavoprotein 2, mitochondrial OS=Homo sapiens OX=9606 GN=NDUFV2 PE=1 SV=2 | 27,4 | 8,06 | 996 | 46 | 1 | 1 | 1 | 1 | 1 | 4 |
| P26368 | Splicing factor U2AF 65 kDa subunit OS=Homo sapiens OX=9606 GN=U2AF2 PE=1 SV=4 | 53,5 | 9,09 | 719 | 46 | 1 | 1 | 1 | 1 | 1 | 2 |
| P56545 | C-terminal-binding protein 2 OS=Homo sapiens OX=9606 GN=CTBP2 PE=1 SV=1 | 48,9 | 6,95 | 890 | 46 | 1 | 1 | 1 | 1 | 1 | 2 |
| P63151 | Serine/threonine-protein phosphatase 2A 55 kDa regulatory subunit B alpha isoform OS=Homo sapiens OX=9606 GN=PPP2R2A PE=1 SV=1 | 51,7 | 6,2 | 1014 | 46 | 1 | 1 | 1 | 1 | 1 | 2 |
| P47897 | Glutamine--tRNA ligase OS=Homo sapiens OX=9606 GN=QARS1 PE=1 SV=1 | 87,7 | 7,15 | 469 | 45 | 1 | 1 | 1 | 1 | 1 | 1 |
| P62714 | Serine/threonine-protein phosphatase 2A catalytic subunit beta isoform OS=Homo sapiens OX=9606 GN=PPP2CB PE=1 SV=1 | 35,6 | 5,43 | 123 | 45 | 1 | 1 | 1 | 1 | 1 | 4 |
| P67870 | Casein kinase II subunit beta OS=Homo sapiens OX=9606 GN=CSNK2B PE=1 SV=1 | 24,9 | 5,55 | 245 | 45 | 1 | 1 | 1 | 1 | 1 | 5 |
| Q5W0B1 | ORC ubiquitin ligase 1 OS=Homo sapiens OX=9606 GN=OBI1 PE=1 SV=1 | 81,1 | 5,72 | 1024 | 45 | 1 | 1 | 1 | 1 | 1 | 1 |
| Q9UBB6 | Neurochondrin OS=Homo sapiens OX=9606 GN=NCDN PE=1 SV=1 | 78,8 | 5,48 | 899 | 45 | 1 | 1 | 1 | 1 | 1 | 2 |
| O75153 | Clustered mitochondria protein homolog OS=Homo sapiens OX=9606 GN=CLUH PE=1 SV=2 | 146,6 | 6,13 | 969 | 44 | 1 | 1 | 1 | 1 | 1 | 1 |
| P08243 | Asparagine synthetase [glutamine-hydrolyzing] OS=Homo sapiens OX=9606 GN=ASNS PE=1 SV=4 | 64,3 | 6,86 | 652 | 44 | 1 | 1 | 1 | 1 | 1 | 2 |
| P62304 | Small nuclear ribonucleoprotein E OS=Homo sapiens OX=9606 GN=SNRPE PE=1 SV=1 | 10,8 | 9,44 | 273 | 44 | 2 | 2 | 2 | 2 | 2 | 25 |
| Q16610 | Extracellular matrix protein 1 OS=Homo sapiens OX=9606 GN=ECM1 PE=1 SV=2 | 60,6 | 6,71 | 1018 | 44 | 1 | 1 | 1 | 1 | 1 | 2 |
| Q9H3G5 | Probable serine carboxypeptidase CPVL OS=Homo sapiens OX=9606 GN=CPVL PE=1 SV=2 | 54,1 | 5,62 | 418 | 44 | 1 | 1 | 1 | 1 | 1 | 2 |
| Q9Y3B9 | RRP15-like protein OS=Homo sapiens OX=9606 GN=RRP15 PE=1 SV=2 | 31,5 | 5,52 | 901 | 44 | 1 | 1 | 1 | 1 | 1 | 4 |
| O14735 | CDP-diacylglycerol--inositol 3-phosphatidyltransferase OS=Homo sapiens OX=9606 GN=CDIPT PE=1 SV=1 | 23,5 | 8,03 | 588 | 43 | 1 | 1 | 1 | 1 | 1 | 5 |
| P61026 | Ras-related protein Rab-10 OS=Homo sapiens OX=9606 GN=RAB10 PE=1 SV=1 | 22,5 | 8,38 | 13 | 43 | 1 | 1 | 1 | 1 | 1 | 6 |
| Q8IYB3 | Serine/arginine repetitive matrix protein 1 OS=Homo sapiens OX=9606 GN=SRRM1 PE=1 SV=2 | 102,3 | 11,84 | 203 | 43 | 1 | 1 | 1 | 1 | 1 | 2 |
| Q9H1K4 | Mitochondrial glutamate carrier 2 OS=Homo sapiens OX=9606 GN=SLC25A18 PE=1 SV=1 | 33,8 | 9,25 | 271 | 43 | 1 | 1 | 1 | 1 | 1 | 3 |
| O94992 | Protein HEXIM1 OS=Homo sapiens OX=9606 GN=HEXIM1 PE=1 SV=1 | 40,6 | 4,89 | 533 | 42 | 1 | 1 | 1 | 1 | 1 | 5 |
| Q13595 | Transformer-2 protein homolog alpha OS=Homo sapiens OX=9606 GN=TRA2A PE=1 SV=1 | 32,7 | 11,27 | 266 | 42 | 1 | 1 | 1 | 1 | 1 | 5 |
| Q8IZT6 | Abnormal spindle-like microcephaly-associated protein OS=Homo sapiens OX=9606 GN=ASPM PE=1 SV=2 | 409,5 | 10,45 | 59 | 42 | 1 | 1 | 1 | 1 | 1 | 0 |
| Q8NB16 | Mixed lineage kinase domain-like protein OS=Homo sapiens OX=9606 GN=MLKL PE=1 SV=1 | 54,4 | 8,82 | 520 | 42 | 1 | 1 | 1 | 1 | 1 | 1 |
| Q9NVI1 | Fanconi anemia group I protein OS=Homo sapiens OX=9606 GN=FANCI PE=1 SV=4 | 149,2 | 6,74 | 17 | 42 | 1 | 1 | 1 | 1 | 1 | 1 |
| P35321 | Cornifin-A OS=Homo sapiens OX=9606 GN=SPRR1A PE=1 SV=2 | 9,9 | 8,48 | 883 | 41 | 1 | 1 | 1 | 1 | 1 | 19 |
| Q13561 | Dynactin subunit 2 OS=Homo sapiens OX=9606 GN=DCTN2 PE=1 SV=4 | 44,2 | 5,21 | 39 | 41 | 1 | 1 | 1 | 1 | 1 | 5 |
| Q16527 | Cysteine and glycine-rich protein 2 OS=Homo sapiens OX=9606 GN=CSRP2 PE=1 SV=3 | 20,9 | 8,62 | 1036 | 41 | 1 | 1 | 1 | 1 | 1 | 5 |
| Q6NUP7 | Serine/threonine-protein phosphatase 4 regulatory subunit 4 OS=Homo sapiens OX=9606 GN=PPP4R4 PE=1 SV=1 | 99,4 | 7,8 | 960 | 41 | 1 | 1 | 1 | 1 | 1 | 1 |
| Q8WXW3 | Progesterone-induced-blocking factor 1 OS=Homo sapiens OX=9606 GN=PIBF1 PE=1 SV=2 | 89,8 | 6,02 | 919 | 41 | 1 | 1 | 1 | 1 | 1 | 2 |
| Q9BVG3 | E3 ubiquitin-protein ligase TRIM62 OS=Homo sapiens OX=9606 GN=TRIM62 PE=1 SV=1 | 54,2 | 6,62 | 748 | 41 | 1 | 1 | 1 | 1 | 1 | 1 |
| O14925 | Mitochondrial import inner membrane translocase subunit Tim23 OS=Homo sapiens OX=9606 GN=TIMM23 PE=1 SV=1 | 21,9 | 8,6 | 746 | 40 | 1 | 1 | 1 | 1 | 1 | 5 |
| P21964 | Catechol O-methyltransferase OS=Homo sapiens OX=9606 GN=COMT PE=1 SV=2 | 30 | 5,47 | 99 | 40 | 1 | 1 | 1 | 1 | 1 | 3 |
| P22528 | Cornifin-B OS=Homo sapiens OX=9606 GN=SPRR1B PE=1 SV=2 | 9,9 | 8,48 | 400 | 40 | 1 | 1 | 1 | 1 | 1 | 19 |
| Q5T011 | KICSTOR complex protein SZT2 OS=Homo sapiens OX=9606 GN=SZT2 PE=1 SV=3 | 377,8 | 6,27 | 157 | 40 | 1 | 1 | 1 | 1 | 1 | 0 |
| Q6ZN17 | Protein lin-28 homolog B OS=Homo sapiens OX=9606 GN=LIN28B PE=1 SV=1 | 27,1 | 8,91 | 733 | 40 | 1 | 1 | 1 | 1 | 1 | 4 |
| Q96TA2 | ATP-dependent zinc metalloprotease YME1L1 OS=Homo sapiens OX=9606 GN=YME1L1 PE=1 SV=2 | 86,4 | 8,76 | 1039 | 40 | 1 | 1 | 1 | 1 | 1 | 2 |
| Q9NUP9 | Protein lin-7 homolog C OS=Homo sapiens OX=9606 GN=LIN7C PE=1 SV=1 | 21,8 | 8,43 | 789 | 40 | 1 | 1 | 1 | 1 | 1 | 4 |
| P23588 | Eukaryotic translation initiation factor 4B OS=Homo sapiens OX=9606 GN=EIF4B PE=1 SV=2 | 69,1 | 5,73 | 589 | 39 | 1 | 1 | 1 | 1 | 1 | 2 |
| P49593 | Protein phosphatase 1F OS=Homo sapiens OX=9606 GN=PPM1F PE=1 SV=3 | 49,8 | 5,1 | 780 | 39 | 1 | 1 | 1 | 1 | 1 | 2 |
| Q12789 | General transcription factor 3C polypeptide 1 OS=Homo sapiens OX=9606 GN=GTF3C1 PE=1 SV=4 | 238,7 | 7,3 | 600 | 39 | 1 | 1 | 1 | 1 | 1 | 1 |
| Q15773 | Myeloid leukemia factor 2 OS=Homo sapiens OX=9606 GN=MLF2 PE=1 SV=1 | 28,1 | 6,9 | 800 | 39 | 1 | 1 | 1 | 1 | 1 | 4 |
| Q6VN20 | Ran-binding protein 10 OS=Homo sapiens OX=9606 GN=RANBP10 PE=1 SV=1 | 67,2 | 6,77 | 521 | 39 | 1 | 1 | 1 | 1 | 1 | 2 |
| Q7L576 | Cytoplasmic FMR1-interacting protein 1 OS=Homo sapiens OX=9606 GN=CYFIP1 PE=1 SV=1 | 145,1 | 6,9 | 801 | 39 | 1 | 1 | 1 | 1 | 1 | 1 |
| Q86SK9 | Stearoyl-CoA desaturase 5 OS=Homo sapiens OX=9606 GN=SCD5 PE=1 SV=2 | 37,6 | 9,61 | 297 | 39 | 1 | 1 | 1 | 1 | 1 | 5 |
| Q8IZP2 | Putative protein FAM10A4 OS=Homo sapiens OX=9606 GN=ST13P4 PE=5 SV=1 | 27,4 | 5,08 | 158 | 39 | 1 | 1 | 1 | 1 | 1 | 6 |
| Q99942 | E3 ubiquitin-protein ligase RNF5 OS=Homo sapiens OX=9606 GN=RNF5 PE=1 SV=1 | 19,9 | 6,65 | 700 | 39 | 1 | 1 | 1 | 1 | 1 | 8 |
| Q9BW19 | Kinesin-like protein KIFC1 OS=Homo sapiens OX=9606 GN=KIFC1 PE=1 SV=2 | 73,7 | 8,98 | 736 | 39 | 1 | 1 | 1 | 1 | 1 | 2 |
| Q9H7H0 | Methyltransferase-like protein 17, mitochondrial OS=Homo sapiens OX=9606 GN=METTL17 PE=1 SV=1 | 50,7 | 9,33 | 103 | 39 | 1 | 1 | 1 | 1 | 1 | 2 |
| O95232 | Luc7-like protein 3 OS=Homo sapiens OX=9606 GN=LUC7L3 PE=1 SV=2 | 51,4 | 9,79 | 714 | 38 | 1 | 1 | 1 | 1 | 1 | 2 |
| P01893 | Putative HLA class I histocompatibility antigen, alpha chain H OS=Homo sapiens OX=9606 GN=HLA-H PE=5 SV=3 | 40,9 | 6,3 | 517 | 38 | 1 | 1 | 1 | 1 | 1 | 7 |
| P12277 | Creatine kinase B-type OS=Homo sapiens OX=9606 GN=CKB PE=1 SV=1 | 42,6 | 5,59 | 631 | 38 | 1 | 1 | 1 | 1 | 1 | 4 |
| P26640 | Valine--tRNA ligase OS=Homo sapiens OX=9606 GN=VARS1 PE=1 SV=4 | 140,4 | 7,59 | 171 | 38 | 1 | 1 | 1 | 1 | 1 | 1 |
| P48556 | 26S proteasome non-ATPase regulatory subunit 8 OS=Homo sapiens OX=9606 GN=PSMD8 PE=1 SV=2 | 39,6 | 9,7 | 32 | 38 | 1 | 1 | 1 | 1 | 1 | 2 |
| P55884 | Eukaryotic translation initiation factor 3 subunit B OS=Homo sapiens OX=9606 GN=EIF3B PE=1 SV=3 | 92,4 | 5 | 732 | 38 | 1 | 1 | 1 | 1 | 1 | 1 |
| Q13642 | Four and a half LIM domains protein 1 OS=Homo sapiens OX=9606 GN=FHL1 PE=1 SV=4 | 36,2 | 8,97 | 394 | 38 | 1 | 1 | 1 | 1 | 1 | 4 |
| O00746 | Nucleoside diphosphate kinase, mitochondrial OS=Homo sapiens OX=9606 GN=NME4 PE=1 SV=1 | 20,6 | 10,29 | 910 | 37 | 1 | 1 | 1 | 1 | 1 | 7 |
| P30876 | DNA-directed RNA polymerase II subunit RPB2 OS=Homo sapiens OX=9606 GN=POLR2B PE=1 SV=1 | 133,8 | 6,87 | 882 | 37 | 1 | 1 | 1 | 1 | 1 | 1 |
| Q71RC2 | La-related protein 4 OS=Homo sapiens OX=9606 GN=LARP4 PE=1 SV=3 | 80,5 | 6,61 | 6 | 37 | 1 | 1 | 1 | 1 | 1 | 1 |
| Q9H7E9 | UPF0488 protein C8orf33 OS=Homo sapiens OX=9606 GN=C8orf33 PE=1 SV=1 | 25 | 9,95 | 840 | 37 | 1 | 1 | 1 | 1 | 1 | 10 |
| Q9HCN8 | Stromal cell-derived factor 2-like protein 1 OS=Homo sapiens OX=9606 GN=SDF2L1 PE=1 SV=2 | 23,6 | 7,03 | 222 | 37 | 1 | 1 | 1 | 1 | 1 | 9 |
| Q9NRP0 | Oligosaccharyltransferase complex subunit OSTC OS=Homo sapiens OX=9606 GN=OSTC PE=1 SV=1 | 16,8 | 9,13 | 924 | 37 | 1 | 1 | 1 | 1 | 1 | 8 |
| Q9NX63 | MICOS complex subunit MIC19 OS=Homo sapiens OX=9606 GN=CHCHD3 PE=1 SV=1 | 26,1 | 8,28 | 759 | 37 | 1 | 1 | 1 | 1 | 1 | 4 |
| Q9Y262 | Eukaryotic translation initiation factor 3 subunit L OS=Homo sapiens OX=9606 GN=EIF3L PE=1 SV=1 | 66,7 | 6,34 | 666 | 37 | 1 | 1 | 1 | 1 | 1 | 1 |
| Q9Y3D3 | 28S ribosomal protein S16, mitochondrial OS=Homo sapiens OX=9606 GN=MRPS16 PE=1 SV=1 | 15,3 | 9,5 | 965 | 37 | 1 | 1 | 1 | 1 | 1 | 13 |
| P09543 | 2',3'-cyclic-nucleotide 3'-phosphodiesterase OS=Homo sapiens OX=9606 GN=CNP PE=1 SV=2 | 47,5 | 9,07 | 482 | 36 | 1 | 1 | 1 | 1 | 1 | 2 |
| Q13185 | Chromobox protein homolog 3 OS=Homo sapiens OX=9606 GN=CBX3 PE=1 SV=4 | 20,8 | 5,33 | 4 | 36 | 1 | 1 | 1 | 1 | 1 | 8 |
| O94854 | Microtubule-actin cross-linking factor 1, isoforms 6/7 OS=Homo sapiens OX=9606 GN=MACF1 PE=2 SV=5 | 388,4 | 4,87 | 270 | 35 | 1 | 1 | 1 | 1 | 1 | 1 |
| P00966 | Argininosuccinate synthase OS=Homo sapiens OX=9606 GN=ASS1 PE=1 SV=2 | 46,5 | 8,02 | 215 | 35 | 1 | 1 | 1 | 1 | 1 | 2 |
| P50454 | Serpin H1 OS=Homo sapiens OX=9606 GN=SERPINH1 PE=1 SV=2 | 46,4 | 8,69 | 914 | 35 | 1 | 1 | 1 | 1 | 1 | 4 |
| P54886 | Delta-1-pyrroline-5-carboxylate synthase OS=Homo sapiens OX=9606 GN=ALDH18A1 PE=1 SV=2 | 87,2 | 7,12 | 293 | 35 | 1 | 1 | 1 | 1 | 1 | 1 |
| Q5XKP0 | MICOS complex subunit MIC13 OS=Homo sapiens OX=9606 GN=MICOS13 PE=1 SV=1 | 13,1 | 9,42 | 936 | 35 | 1 | 1 | 1 | 1 | 1 | 5 |
| Q6ZN55 | Zinc finger protein 574 OS=Homo sapiens OX=9606 GN=ZNF574 PE=1 SV=2 | 98,8 | 8,07 | 596 | 35 | 1 | 1 | 1 | 1 | 1 | 1 |
| Q9BVK6 | Transmembrane emp24 domain-containing protein 9 OS=Homo sapiens OX=9606 GN=TMED9 PE=1 SV=2 | 27,3 | 8,02 | 567 | 35 | 1 | 1 | 1 | 1 | 1 | 4 |
| Q9NRW3 | DNA dC->dU-editing enzyme APOBEC-3C OS=Homo sapiens OX=9606 GN=APOBEC3C PE=1 SV=2 | 22,8 | 7,59 | 820 | 35 | 1 | 1 | 1 | 1 | 1 | 7 |
| Q9NTK5 | Obg-like ATPase 1 OS=Homo sapiens OX=9606 GN=OLA1 PE=1 SV=2 | 44,7 | 7,81 | 145 | 35 | 1 | 1 | 1 | 1 | 1 | 2 |
| O15372 | Eukaryotic translation initiation factor 3 subunit H OS=Homo sapiens OX=9606 GN=EIF3H PE=1 SV=1 | 39,9 | 6,54 | 618 | 34 | 1 | 1 | 1 | 1 | 1 | 3 |
| O75832 | 26S proteasome non-ATPase regulatory subunit 10 OS=Homo sapiens OX=9606 GN=PSMD10 PE=1 SV=1 | 24,4 | 6,1 | 1031 | 34 | 1 | 1 | 1 | 1 | 1 | 4 |
| O95989 | Diphosphoinositol polyphosphate phosphohydrolase 1 OS=Homo sapiens OX=9606 GN=NUDT3 PE=1 SV=1 | 19,5 | 6,34 | 313 | 34 | 1 | 1 | 1 | 1 | 1 | 4 |
| P01859 | Immunoglobulin heavy constant gamma 2 OS=Homo sapiens OX=9606 GN=IGHG2 PE=1 SV=3 | 43,8 | 6,52 | 11 | 34 | 2 | 1 | 2 | 1 | 1 | 2 |
| P28331 | NADH-ubiquinone oxidoreductase 75 kDa subunit, mitochondrial OS=Homo sapiens OX=9606 GN=NDUFS1 PE=1 SV=3 | 79,4 | 6,23 | 70 | 34 | 1 | 1 | 1 | 1 | 1 | 3 |
| P52298 | Nuclear cap-binding protein subunit 2 OS=Homo sapiens OX=9606 GN=NCBP2 PE=1 SV=1 | 18 | 8,21 | 75 | 34 | 1 | 1 | 1 | 1 | 1 | 7 |
| P82664 | 28S ribosomal protein S10, mitochondrial OS=Homo sapiens OX=9606 GN=MRPS10 PE=1 SV=2 | 23 | 8 | 745 | 34 | 1 | 1 | 1 | 1 | 1 | 9 |
| Q04837 | Single-stranded DNA-binding protein, mitochondrial OS=Homo sapiens OX=9606 GN=SSBP1 PE=1 SV=1 | 17,2 | 9,6 | 110 | 34 | 1 | 1 | 1 | 1 | 1 | 10 |
| Q8WUM0 | Nuclear pore complex protein Nup133 OS=Homo sapiens OX=9606 GN=NUP133 PE=1 SV=2 | 128,9 | 5,1 | 150 | 34 | 1 | 1 | 1 | 1 | 1 | 1 |
| Q9BUL9 | Ribonuclease P protein subunit p25 OS=Homo sapiens OX=9606 GN=RPP25 PE=1 SV=1 | 20,6 | 9,61 | 570 | 34 | 1 | 1 | 1 | 1 | 1 | 7 |
| Q9H1R2 | Dual specificity protein phosphatase 15 OS=Homo sapiens OX=9606 GN=DUSP15 PE=1 SV=4 | 31,9 | 8,38 | 979 | 34 | 1 | 1 | 1 | 1 | 1 | 2 |
| O14579 | Coatomer subunit epsilon OS=Homo sapiens OX=9606 GN=COPE PE=1 SV=3 | 34,5 | 5,12 | 347 | 33 | 1 | 1 | 1 | 1 | 1 | 5 |
| O75323 | Protein NipSnap homolog 2 OS=Homo sapiens OX=9606 GN=NIPSNAP2 PE=1 SV=1 | 33,7 | 9,36 | 310 | 33 | 1 | 1 | 1 | 1 | 1 | 2 |
| P38432 | Coilin OS=Homo sapiens OX=9606 GN=COIL PE=1 SV=1 | 62,6 | 9,07 | 963 | 33 | 1 | 1 | 1 | 1 | 1 | 2 |
| P49721 | Proteasome subunit beta type-2 OS=Homo sapiens OX=9606 GN=PSMB2 PE=1 SV=1 | 22,8 | 7,02 | 452 | 33 | 1 | 1 | 1 | 1 | 1 | 4 |
| P51114 | RNA-binding protein FXR1 OS=Homo sapiens OX=9606 GN=FXR1 PE=1 SV=3 | 69,7 | 6,15 | 423 | 33 | 1 | 1 | 1 | 1 | 1 | 1 |
| P51659 | Peroxisomal multifunctional enzyme type 2 OS=Homo sapiens OX=9606 GN=HSD17B4 PE=1 SV=3 | 79,6 | 8,84 | 451 | 33 | 1 | 1 | 1 | 1 | 1 | 2 |
| P61758 | Prefoldin subunit 3 OS=Homo sapiens OX=9606 GN=VBP1 PE=1 SV=4 | 22,6 | 7,11 | 14 | 33 | 1 | 1 | 1 | 1 | 1 | 6 |
| Q05193 | Dynamin-1 OS=Homo sapiens OX=9606 GN=DNM1 PE=1 SV=2 | 97,3 | 7,17 | 473 | 33 | 1 | 1 | 1 | 1 | 1 | 1 |
| Q15382 | GTP-binding protein Rheb OS=Homo sapiens OX=9606 GN=RHEB PE=1 SV=1 | 20,5 | 5,92 | 940 | 33 | 1 | 1 | 1 | 1 | 1 | 4 |
| Q6P5R6 | 60S ribosomal protein L22-like 1 OS=Homo sapiens OX=9606 GN=RPL22L1 PE=1 SV=2 | 14,6 | 9,38 | 796 | 33 | 1 | 1 | 1 | 1 | 1 | 10 |
| Q92499 | ATP-dependent RNA helicase DDX1 OS=Homo sapiens OX=9606 GN=DDX1 PE=1 SV=2 | 82,4 | 7,23 | 512 | 33 | 1 | 1 | 1 | 1 | 1 | 1 |
| Q9BYC9 | 39S ribosomal protein L20, mitochondrial OS=Homo sapiens OX=9606 GN=MRPL20 PE=1 SV=1 | 17,4 | 10,86 | 1017 | 33 | 1 | 1 | 1 | 1 | 1 | 7 |
| O14929 | Histone acetyltransferase type B catalytic subunit OS=Homo sapiens OX=9606 GN=HAT1 PE=1 SV=2 | 49,5 | 5,69 | 251 | 32 | 1 | 1 | 1 | 1 | 1 | 3 |
| O75475 | PC4 and SFRS1-interacting protein OS=Homo sapiens OX=9606 GN=PSIP1 PE=1 SV=1 | 60,1 | 9,13 | 367 | 32 | 1 | 1 | 1 | 1 | 1 | 2 |
| P20742 | Pregnancy zone protein OS=Homo sapiens OX=9606 GN=PZP PE=1 SV=4 | 163,8 | 6,38 | 845 | 32 | 1 | 1 | 1 | 1 | 1 | 1 |
| P82912 | 28S ribosomal protein S11, mitochondrial OS=Homo sapiens OX=9606 GN=MRPS11 PE=1 SV=2 | 20,6 | 10,81 | 828 | 32 | 1 | 1 | 1 | 1 | 1 | 9 |
| Q53H12 | Acylglycerol kinase, mitochondrial OS=Homo sapiens OX=9606 GN=AGK PE=1 SV=2 | 47,1 | 8,09 | 891 | 32 | 1 | 1 | 1 | 1 | 1 | 3 |
| Q8TEX9 | Importin-4 OS=Homo sapiens OX=9606 GN=IPO4 PE=1 SV=2 | 118,6 | 4,96 | 827 | 32 | 1 | 1 | 1 | 1 | 1 | 1 |
| Q8WWC4 | m-AAA protease-interacting protein 1, mitochondrial OS=Homo sapiens OX=9606 GN=MAIP1 PE=1 SV=1 | 32,5 | 9,17 | 519 | 32 | 1 | 1 | 1 | 1 | 1 | 3 |
| Q9BZI7 | Regulator of nonsense transcripts 3B OS=Homo sapiens OX=9606 GN=UPF3B PE=1 SV=1 | 57,7 | 9,48 | 282 | 32 | 1 | 1 | 1 | 1 | 1 | 2 |
| Q14978 | Nucleolar and coiled-body phosphoprotein 1 OS=Homo sapiens OX=9606 GN=NOLC1 PE=1 SV=2 | 73,6 | 9,47 | 629 | 31 | 1 | 1 | 1 | 1 | 1 | 1 |
| Q15629 | Translocating chain-associated membrane protein 1 OS=Homo sapiens OX=9606 GN=TRAM1 PE=1 SV=3 | 43 | 9,63 | 142 | 31 | 1 | 1 | 1 | 1 | 1 | 3 |
| Q15651 | High mobility group nucleosome-binding domain-containing protein 3 OS=Homo sapiens OX=9606 GN=HMGN3 PE=1 SV=2 | 10,7 | 9,66 | 111 | 31 | 1 | 1 | 1 | 1 | 1 | 13 |
| Q3KQU3 | MAP7 domain-containing protein 1 OS=Homo sapiens OX=9606 GN=MAP7D1 PE=1 SV=1 | 92,8 | 10,11 | 973 | 31 | 1 | 1 | 1 | 1 | 1 | 1 |
| Q6AI12 | Ankyrin repeat domain-containing protein 40 OS=Homo sapiens OX=9606 GN=ANKRD40 PE=1 SV=2 | 41,1 | 4,97 | 370 | 31 | 2 | 1 | 2 | 1 | 1 | 3 |
| Q7Z3B4 | Nucleoporin p54 OS=Homo sapiens OX=9606 GN=NUP54 PE=1 SV=2 | 55,4 | 7,02 | 290 | 31 | 1 | 1 | 1 | 1 | 1 | 3 |
| Q96QR8 | Transcriptional activator protein Pur-beta OS=Homo sapiens OX=9606 GN=PURB PE=1 SV=3 | 33,2 | 5,43 | 677 | 31 | 1 | 1 | 1 | 1 | 1 | 4 |
| Q96T37 | RNA-binding protein 15 OS=Homo sapiens OX=9606 GN=RBM15 PE=1 SV=2 | 107,1 | 10,08 | 419 | 31 | 1 | 1 | 1 | 1 | 1 | 1 |
| Q9NWZ8 | Gem-associated protein 8 OS=Homo sapiens OX=9606 GN=GEMIN8 PE=1 SV=1 | 28,6 | 6,8 | 416 | 31 | 1 | 1 | 1 | 1 | 1 | 5 |
| O14545 | TRAF-type zinc finger domain-containing protein 1 OS=Homo sapiens OX=9606 GN=TRAFD1 PE=1 SV=1 | 64,8 | 5,29 | 264 | 30 | 1 | 1 | 1 | 1 | 1 | 2 |
| O14828 | Secretory carrier-associated membrane protein 3 OS=Homo sapiens OX=9606 GN=SCAMP3 PE=1 SV=3 | 38,3 | 7,64 | 314 | 30 | 1 | 1 | 1 | 1 | 1 | 4 |
| O43684 | Mitotic checkpoint protein BUB3 OS=Homo sapiens OX=9606 GN=BUB3 PE=1 SV=1 | 37,1 | 6,84 | 84 | 30 | 1 | 1 | 1 | 1 | 1 | 2 |
| P05386 | 60S acidic ribosomal protein P1 OS=Homo sapiens OX=9606 GN=RPLP1 PE=1 SV=1 | 11,5 | 4,32 | 291 | 30 | 1 | 1 | 1 | 1 | 1 | 14 |
| P35520 | Cystathionine beta-synthase OS=Homo sapiens OX=9606 GN=CBS PE=1 SV=2 | 60,5 | 6,65 | 131 | 30 | 1 | 1 | 1 | 1 | 1 | 1 |
| Q8NE71 | ATP-binding cassette sub-family F member 1 OS=Homo sapiens OX=9606 GN=ABCF1 PE=1 SV=2 | 95,9 | 6,8 | 73 | 30 | 1 | 1 | 1 | 1 | 1 | 1 |
| Q8NEN0 | Armadillo repeat-containing protein 2 OS=Homo sapiens OX=9606 GN=ARMC2 PE=1 SV=4 | 96,8 | 8,22 | 345 | 30 | 1 | 1 | 1 | 1 | 1 | 1 |
| Q8WXE1 | ATR-interacting protein OS=Homo sapiens OX=9606 GN=ATRIP PE=1 SV=1 | 85,8 | 6,32 | 691 | 30 | 1 | 1 | 1 | 1 | 1 | 1 |
| Q96A35 | 39S ribosomal protein L24, mitochondrial OS=Homo sapiens OX=9606 GN=MRPL24 PE=1 SV=1 | 24,9 | 9,29 | 1037 | 30 | 1 | 1 | 1 | 1 | 1 | 6 |
| Q99436 | Proteasome subunit beta type-7 OS=Homo sapiens OX=9606 GN=PSMB7 PE=1 SV=1 | 29,9 | 7,68 | 186 | 30 | 1 | 1 | 1 | 1 | 1 | 4 |
| Q9H4I3 | TraB domain-containing protein OS=Homo sapiens OX=9606 GN=TRABD PE=1 SV=1 | 42,3 | 8 | 645 | 30 | 1 | 1 | 1 | 1 | 1 | 3 |
| Q9NZQ3 | NCK-interacting protein with SH3 domain OS=Homo sapiens OX=9606 GN=NCKIPSD PE=1 SV=1 | 78,9 | 6,38 | 467 | 30 | 1 | 1 | 1 | 1 | 1 | 1 |
| Q12769 | Nuclear pore complex protein Nup160 OS=Homo sapiens OX=9606 GN=NUP160 PE=1 SV=3 | 162 | 5,5 | 26 | 29 | 1 | 1 | 1 | 1 | 1 | 1 |
| Q8WUA4 | General transcription factor 3C polypeptide 2 OS=Homo sapiens OX=9606 GN=GTF3C2 PE=1 SV=2 | 100,6 | 7,31 | 643 | 29 | 1 | 1 | 1 | 1 | 1 | 1 |
| Q8WYP5 | Protein ELYS OS=Homo sapiens OX=9606 GN=AHCTF1 PE=1 SV=3 | 252,3 | 6,6 | 442 | 29 | 1 | 1 | 1 | 1 | 1 | 1 |
| Q99733 | Nucleosome assembly protein 1-like 4 OS=Homo sapiens OX=9606 GN=NAP1L4 PE=1 SV=1 | 42,8 | 4,69 | 565 | 29 | 1 | 1 | 1 | 1 | 1 | 5 |
| Q9NZJ7 | Mitochondrial carrier homolog 1 OS=Homo sapiens OX=9606 GN=MTCH1 PE=1 SV=1 | 41,5 | 9,32 | 516 | 29 | 1 | 1 | 1 | 1 | 1 | 3 |
| Q9UMS4 | Pre-mRNA-processing factor 19 OS=Homo sapiens OX=9606 GN=PRPF19 PE=1 SV=1 | 55,1 | 6,61 | 395 | 29 | 1 | 1 | 1 | 1 | 1 | 2 |
| Q9Y679 | Lipid droplet-regulating VLDL assembly factor AUP1 OS=Homo sapiens OX=9606 GN=AUP1 PE=1 SV=2 | 45,8 | 8,65 | 539 | 29 | 1 | 1 | 1 | 1 | 1 | 3 |
| O43615 | Mitochondrial import inner membrane translocase subunit TIM44 OS=Homo sapiens OX=9606 GN=TIMM44 PE=1 SV=2 | 51,3 | 8,32 | 220 | 28 | 1 | 1 | 1 | 1 | 1 | 3 |
| O60662 | Kelch-like protein 41 OS=Homo sapiens OX=9606 GN=KLHL41 PE=1 SV=2 | 68 | 5,29 | 67 | 28 | 1 | 1 | 1 | 1 | 1 | 1 |
| O60684 | Importin subunit alpha-7 OS=Homo sapiens OX=9606 GN=KPNA6 PE=1 SV=1 | 60 | 4,98 | 754 | 28 | 1 | 1 | 1 | 1 | 1 | 1 |
| P02810 | Salivary acidic proline-rich phosphoprotein 1/2 OS=Homo sapiens OX=9606 GN=PRH1 PE=1 SV=3 | 17 | 4,96 | 847 | 28 | 1 | 1 | 1 | 1 | 1 | 10 |
| P08574 | Cytochrome c1, heme protein, mitochondrial OS=Homo sapiens OX=9606 GN=CYC1 PE=1 SV=3 | 35,4 | 9 | 863 | 28 | 1 | 1 | 1 | 1 | 1 | 2 |
| P09012 | U1 small nuclear ribonucleoprotein A OS=Homo sapiens OX=9606 GN=SNRPA PE=1 SV=3 | 31,3 | 9,83 | 114 | 28 | 1 | 1 | 1 | 1 | 1 | 11 |
| P22102 | Trifunctional purine biosynthetic protein adenosine-3 OS=Homo sapiens OX=9606 GN=GART PE=1 SV=1 | 107,7 | 6,7 | 342 | 28 | 1 | 1 | 1 | 1 | 1 | 2 |
| P61009 | Signal peptidase complex subunit 3 OS=Homo sapiens OX=9606 GN=SPCS3 PE=1 SV=1 | 20,3 | 8,62 | 311 | 28 | 1 | 1 | 1 | 1 | 1 | 7 |
| Q16795 | NADH dehydrogenase [ubiquinone] 1 alpha subcomplex subunit 9, mitochondrial OS=Homo sapiens OX=9606 GN=NDUFA9 PE=1 SV=2 | 42,5 | 9,8 | 915 | 28 | 1 | 1 | 1 | 1 | 1 | 3 |
| Q7L0Y3 | tRNA methyltransferase 10 homolog C OS=Homo sapiens OX=9606 GN=TRMT10C PE=1 SV=2 | 47,3 | 9,36 | 229 | 28 | 1 | 1 | 1 | 1 | 1 | 2 |
| Q8WUY1 | Protein THEM6 OS=Homo sapiens OX=9606 GN=THEM6 PE=1 SV=2 | 23,9 | 9,55 | 513 | 28 | 1 | 1 | 1 | 1 | 1 | 4 |
| Q99471 | Prefoldin subunit 5 OS=Homo sapiens OX=9606 GN=PFDN5 PE=1 SV=2 | 17,3 | 6,33 | 309 | 28 | 1 | 1 | 1 | 1 | 1 | 10 |
| E9PRG8 | Uncharacterized protein C11orf98 OS=Homo sapiens OX=9606 GN=C11orf98 PE=4 SV=2 | 14,2 | 11,53 | 195 | 27 | 1 | 1 | 1 | 1 | 1 | 11 |
| O15260 | Surfeit locus protein 4 OS=Homo sapiens OX=9606 GN=SURF4 PE=1 SV=3 | 30,4 | 7,78 | 74 | 27 | 1 | 1 | 1 | 1 | 1 | 3 |
| O43159 | Ribosomal RNA-processing protein 8 OS=Homo sapiens OX=9606 GN=RRP8 PE=1 SV=2 | 50,7 | 9,42 | 892 | 27 | 1 | 1 | 1 | 1 | 1 | 2 |
| O43164 | E3 ubiquitin-protein ligase Praja-2 OS=Homo sapiens OX=9606 GN=PJA2 PE=1 SV=4 | 78,2 | 4,39 | 702 | 27 | 1 | 1 | 1 | 1 | 1 | 3 |
| O75874 | Isocitrate dehydrogenase [NADP] cytoplasmic OS=Homo sapiens OX=9606 GN=IDH1 PE=1 SV=2 | 46,6 | 7,01 | 240 | 27 | 2 | 1 | 2 | 1 | 1 | 3 |
| O95202 | Mitochondrial proton/calcium exchanger protein OS=Homo sapiens OX=9606 GN=LETM1 PE=1 SV=1 | 83,3 | 6,7 | 381 | 27 | 1 | 1 | 1 | 1 | 1 | 2 |
| P09936 | Ubiquitin carboxyl-terminal hydrolase isozyme L1 OS=Homo sapiens OX=9606 GN=UCHL1 PE=1 SV=2 | 24,8 | 5,48 | 202 | 27 | 1 | 1 | 1 | 1 | 1 | 4 |
| P35249 | Replication factor C subunit 4 OS=Homo sapiens OX=9606 GN=RFC4 PE=1 SV=2 | 39,7 | 8,02 | 295 | 27 | 1 | 1 | 1 | 1 | 1 | 3 |
| P53041 | Serine/threonine-protein phosphatase 5 OS=Homo sapiens OX=9606 GN=PPP5C PE=1 SV=1 | 56,8 | 6,28 | 768 | 27 | 1 | 1 | 1 | 1 | 1 | 3 |
| P78346 | Ribonuclease P protein subunit p30 OS=Homo sapiens OX=9606 GN=RPP30 PE=1 SV=1 | 29,3 | 8,91 | 499 | 27 | 1 | 1 | 1 | 1 | 1 | 2 |
| Q00577 | Transcriptional activator protein Pur-alpha OS=Homo sapiens OX=9606 GN=PURA PE=1 SV=2 | 34,9 | 6,44 | 3 | 27 | 1 | 1 | 1 | 1 | 1 | 3 |
| Q8TB61 | Adenosine 3'-phospho 5'-phosphosulfate transporter 1 OS=Homo sapiens OX=9606 GN=SLC35B2 PE=1 SV=1 | 47,5 | 9,16 | 564 | 27 | 1 | 1 | 1 | 1 | 1 | 3 |
| Q9P0M9 | 39S ribosomal protein L27, mitochondrial OS=Homo sapiens OX=9606 GN=MRPL27 PE=1 SV=1 | 16,1 | 10,42 | 332 | 27 | 1 | 1 | 1 | 1 | 1 | 7 |
| Q9UBQ5 | Eukaryotic translation initiation factor 3 subunit K OS=Homo sapiens OX=9606 GN=EIF3K PE=1 SV=1 | 25 | 4,93 | 296 | 27 | 1 | 1 | 1 | 1 | 1 | 6 |
| O43896 | Kinesin-like protein KIF1C OS=Homo sapiens OX=9606 GN=KIF1C PE=1 SV=3 | 122,9 | 6,9 | 219 | 26 | 1 | 1 | 1 | 1 | 1 | 1 |
| P0C7P4 | Putative cytochrome b-c1 complex subunit Rieske-like protein 1 OS=Homo sapiens OX=9606 GN=UQCRFS1P1 PE=5 SV=1 | 30,8 | 8,87 | 322 | 26 | 1 | 1 | 1 | 1 | 1 | 3 |
| P49821 | NADH dehydrogenase [ubiquinone] flavoprotein 1, mitochondrial OS=Homo sapiens OX=9606 GN=NDUFV1 PE=1 SV=4 | 50,8 | 8,21 | 341 | 26 | 1 | 1 | 1 | 1 | 1 | 2 |
| P53803 | DNA-directed RNA polymerases I, II, and III subunit RPABC4 OS=Homo sapiens OX=9606 GN=POLR2K PE=1 SV=1 | 7 | 9,06 | 35 | 26 | 1 | 1 | 1 | 1 | 1 | 12 |
| P54727 | UV excision repair protein RAD23 homolog B OS=Homo sapiens OX=9606 GN=RAD23B PE=1 SV=1 | 43,1 | 4,84 | 983 | 26 | 1 | 1 | 1 | 1 | 1 | 2 |
| P56556 | NADH dehydrogenase [ubiquinone] 1 alpha subcomplex subunit 6 OS=Homo sapiens OX=9606 GN=NDUFA6 PE=1 SV=4 | 15,1 | 9,98 | 791 | 26 | 1 | 1 | 1 | 1 | 1 | 6 |
| Q13601 | KRR1 small subunit processome component homolog OS=Homo sapiens OX=9606 GN=KRR1 PE=1 SV=4 | 43,6 | 9,77 | 938 | 26 | 1 | 1 | 1 | 1 | 1 | 2 |
| Q5SRE5 | Nucleoporin NUP188 OS=Homo sapiens OX=9606 GN=NUP188 PE=1 SV=1 | 195,9 | 6,73 | 373 | 26 | 1 | 1 | 1 | 1 | 1 | 1 |
| Q96DV4 | 39S ribosomal protein L38, mitochondrial OS=Homo sapiens OX=9606 GN=MRPL38 PE=1 SV=2 | 44,6 | 7,53 | 321 | 26 | 1 | 1 | 1 | 1 | 1 | 3 |
| Q96T88 | E3 ubiquitin-protein ligase UHRF1 OS=Homo sapiens OX=9606 GN=UHRF1 PE=1 SV=1 | 89,8 | 7,56 | 523 | 26 | 1 | 1 | 1 | 1 | 1 | 2 |
| Q9NR09 | Baculoviral IAP repeat-containing protein 6 OS=Homo sapiens OX=9606 GN=BIRC6 PE=1 SV=3 | 529,9 | 6,05 | 807 | 26 | 1 | 1 | 1 | 1 | 1 | 0 |
| Q9NRN7 | L-aminoadipate-semialdehyde dehydrogenase-phosphopantetheinyl transferase OS=Homo sapiens OX=9606 GN=AASDHPPT PE=1 SV=2 | 35,8 | 6,8 | 201 | 26 | 1 | 1 | 1 | 1 | 1 | 6 |
| O60783 | 28S ribosomal protein S14, mitochondrial OS=Homo sapiens OX=9606 GN=MRPS14 PE=1 SV=1 | 15,1 | 11,41 | 441 | 25 | 1 | 1 | 1 | 1 | 1 | 10 |
| P30566 | Adenylosuccinate lyase OS=Homo sapiens OX=9606 GN=ADSL PE=1 SV=2 | 54,9 | 7,11 | 860 | 25 | 1 | 1 | 1 | 1 | 1 | 2 |
| P51784 | Ubiquitin carboxyl-terminal hydrolase 11 OS=Homo sapiens OX=9606 GN=USP11 PE=1 SV=3 | 109,7 | 5,45 | 479 | 25 | 1 | 1 | 1 | 1 | 1 | 1 |
| Q15392 | Delta(24)-sterol reductase OS=Homo sapiens OX=9606 GN=DHCR24 PE=1 SV=2 | 60,1 | 8,16 | 726 | 25 | 1 | 1 | 1 | 1 | 1 | 2 |
| Q6UB35 | Monofunctional C1-tetrahydrofolate synthase, mitochondrial OS=Homo sapiens OX=9606 GN=MTHFD1L PE=1 SV=1 | 105,7 | 8,06 | 575 | 25 | 1 | 1 | 1 | 1 | 1 | 1 |
| Q8N163 | Cell cycle and apoptosis regulator protein 2 OS=Homo sapiens OX=9606 GN=CCAR2 PE=1 SV=2 | 102,8 | 5,22 | 141 | 25 | 1 | 1 | 1 | 1 | 1 | 1 |
| Q9BRX2 | Protein pelota homolog OS=Homo sapiens OX=9606 GN=PELO PE=1 SV=2 | 43,3 | 6,34 | 864 | 25 | 1 | 1 | 1 | 1 | 1 | 3 |
| Q9BSD7 | Cancer-related nucleoside-triphosphatase OS=Homo sapiens OX=9606 GN=NTPCR PE=1 SV=1 | 20,7 | 9,54 | 997 | 25 | 1 | 1 | 1 | 1 | 1 | 6 |
| Q9UNQ2 | Probable dimethyladenosine transferase OS=Homo sapiens OX=9606 GN=DIMT1 PE=1 SV=1 | 35,2 | 9,99 | 581 | 25 | 1 | 1 | 1 | 1 | 1 | 4 |
| A1L390 | Pleckstrin homology domain-containing family G member 3 OS=Homo sapiens OX=9606 GN=PLEKHG3 PE=1 SV=1 | 134,3 | 6,55 | 465 | 24 | 1 | 1 | 1 | 1 | 1 | 1 |
| O43660 | Pleiotropic regulator 1 OS=Homo sapiens OX=9606 GN=PLRG1 PE=1 SV=1 | 57,2 | 9,17 | 927 | 24 | 1 | 1 | 1 | 1 | 1 | 2 |
| O95140 | Mitofusin-2 OS=Homo sapiens OX=9606 GN=MFN2 PE=1 SV=3 | 86,3 | 6,98 | 463 | 24 | 1 | 1 | 1 | 1 | 1 | 1 |
| O95573 | Fatty acid CoA ligase Acsl3 OS=Homo sapiens OX=9606 GN=ACSL3 PE=1 SV=3 | 80,4 | 8,38 | 380 | 24 | 1 | 1 | 1 | 1 | 1 | 2 |
| P17900 | Ganglioside GM2 activator OS=Homo sapiens OX=9606 GN=GM2A PE=1 SV=4 | 20,8 | 5,31 | 1003 | 24 | 1 | 1 | 1 | 1 | 1 | 5 |
| P78417 | Glutathione S-transferase omega-1 OS=Homo sapiens OX=9606 GN=GSTO1 PE=1 SV=2 | 27,5 | 6,6 | 31 | 24 | 1 | 1 | 1 | 1 | 1 | 4 |
| Q07157 | Tight junction protein ZO-1 OS=Homo sapiens OX=9606 GN=TJP1 PE=1 SV=3 | 195,3 | 6,7 | 833 | 24 | 1 | 1 | 1 | 1 | 1 | 1 |
| Q8N5C6 | S1 RNA-binding domain-containing protein 1 OS=Homo sapiens OX=9606 GN=SRBD1 PE=1 SV=2 | 111,7 | 8,72 | 429 | 24 | 1 | 1 | 1 | 1 | 1 | 1 |
| Q96JM3 | Chromosome alignment-maintaining phosphoprotein 1 OS=Homo sapiens OX=9606 GN=CHAMP1 PE=1 SV=2 | 89 | 8,44 | 401 | 24 | 1 | 1 | 1 | 1 | 1 | 1 |
| Q9BTD8 | RNA-binding protein 42 OS=Homo sapiens OX=9606 GN=RBM42 PE=1 SV=1 | 50,4 | 9,63 | 838 | 24 | 1 | 1 | 1 | 1 | 1 | 2 |
| Q9P0J6 | 39S ribosomal protein L36, mitochondrial OS=Homo sapiens OX=9606 GN=MRPL36 PE=1 SV=1 | 11,8 | 11,27 | 183 | 24 | 1 | 1 | 1 | 1 | 1 | 6 |
| Q9Y2L1 | Exosome complex exonuclease RRP44 OS=Homo sapiens OX=9606 GN=DIS3 PE=1 SV=2 | 108,9 | 7,14 | 134 | 24 | 1 | 1 | 1 | 1 | 1 | 2 |
| O00178 | GTP-binding protein 1 OS=Homo sapiens OX=9606 GN=GTPBP1 PE=1 SV=3 | 72,4 | 8,34 | 206 | 23 | 1 | 1 | 1 | 1 | 1 | 2 |
| O43824 | Putative GTP-binding protein 6 OS=Homo sapiens OX=9606 GN=GTPBP6 PE=1 SV=4 | 56,9 | 9,42 | 16 | 23 | 1 | 1 | 1 | 1 | 1 | 2 |
| P11310 | Medium-chain specific acyl-CoA dehydrogenase, mitochondrial OS=Homo sapiens OX=9606 GN=ACADM PE=1 SV=1 | 46,6 | 8,37 | 713 | 23 | 1 | 1 | 1 | 1 | 1 | 2 |
| P11441 | Ubiquitin-like protein 4A OS=Homo sapiens OX=9606 GN=UBL4A PE=1 SV=1 | 17,8 | 8,66 | 448 | 23 | 1 | 1 | 1 | 1 | 1 | 6 |
| P43246 | DNA mismatch repair protein Msh2 OS=Homo sapiens OX=9606 GN=MSH2 PE=1 SV=1 | 104,7 | 5,77 | 244 | 23 | 1 | 1 | 1 | 1 | 1 | 1 |
| P48651 | Phosphatidylserine synthase 1 OS=Homo sapiens OX=9606 GN=PTDSS1 PE=1 SV=1 | 55,5 | 8,43 | 682 | 23 | 1 | 1 | 1 | 1 | 1 | 2 |
| P48730 | Casein kinase I isoform delta OS=Homo sapiens OX=9606 GN=CSNK1D PE=1 SV=2 | 47,3 | 9,74 | 537 | 23 | 1 | 1 | 1 | 1 | 1 | 3 |
| P78347 | General transcription factor II-I OS=Homo sapiens OX=9606 GN=GTF2I PE=1 SV=2 | 112,3 | 6,39 | 665 | 23 | 1 | 1 | 1 | 1 | 1 | 1 |
| Q13084 | 39S ribosomal protein L28, mitochondrial OS=Homo sapiens OX=9606 GN=MRPL28 PE=1 SV=4 | 30,1 | 8,29 | 852 | 23 | 1 | 1 | 1 | 1 | 1 | 4 |
| Q15386 | Ubiquitin-protein ligase E3C OS=Homo sapiens OX=9606 GN=UBE3C PE=1 SV=3 | 123,8 | 6,71 | 268 | 23 | 1 | 1 | 1 | 1 | 1 | 1 |
| Q8WVX9 | Fatty acyl-CoA reductase 1 OS=Homo sapiens OX=9606 GN=FAR1 PE=1 SV=1 | 59,3 | 9,17 | 599 | 23 | 1 | 1 | 1 | 1 | 1 | 3 |
| Q96CS3 | FAS-associated factor 2 OS=Homo sapiens OX=9606 GN=FAF2 PE=1 SV=2 | 52,6 | 5,62 | 586 | 23 | 1 | 1 | 1 | 1 | 1 | 3 |
| Q9UBF2 | Coatomer subunit gamma-2 OS=Homo sapiens OX=9606 GN=COPG2 PE=1 SV=1 | 97,6 | 5,81 | 525 | 23 | 1 | 1 | 1 | 1 | 1 | 1 |
| Q9UI42 | Carboxypeptidase A4 OS=Homo sapiens OX=9606 GN=CPA4 PE=1 SV=2 | 47,3 | 6,7 | 22 | 23 | 1 | 1 | 1 | 1 | 1 | 2 |
| Q9UPN4 | Centrosomal protein of 131 kDa OS=Homo sapiens OX=9606 GN=CEP131 PE=1 SV=3 | 122,1 | 8,69 | 486 | 23 | 1 | 1 | 1 | 1 | 1 | 1 |
| O00767 | Stearoyl-CoA desaturase OS=Homo sapiens OX=9606 GN=SCD PE=1 SV=2 | 41,5 | 9 | 959 | 22 | 1 | 1 | 1 | 1 | 1 | 4 |
| O75351 | Vacuolar protein sorting-associated protein 4B OS=Homo sapiens OX=9606 GN=VPS4B PE=1 SV=2 | 49,3 | 7,23 | 334 | 22 | 1 | 1 | 1 | 1 | 1 | 3 |
| P02765 | Alpha-2-HS-glycoprotein OS=Homo sapiens OX=9606 GN=AHSG PE=1 SV=2 | 39,3 | 5,72 | 79 | 22 | 1 | 1 | 1 | 1 | 1 | 4 |
| P82932 | 28S ribosomal protein S6, mitochondrial OS=Homo sapiens OX=9606 GN=MRPS6 PE=1 SV=3 | 14,2 | 9,26 | 205 | 22 | 1 | 1 | 1 | 1 | 1 | 6 |
| Q13610 | Periodic tryptophan protein 1 homolog OS=Homo sapiens OX=9606 GN=PWP1 PE=1 SV=1 | 55,8 | 4,77 | 908 | 22 | 1 | 1 | 1 | 1 | 1 | 2 |
| Q13867 | Bleomycin hydrolase OS=Homo sapiens OX=9606 GN=BLMH PE=1 SV=1 | 52,5 | 6,27 | 823 | 22 | 1 | 1 | 1 | 1 | 1 | 4 |
| Q15005 | Signal peptidase complex subunit 2 OS=Homo sapiens OX=9606 GN=SPCS2 PE=1 SV=3 | 25 | 8,47 | 698 | 22 | 1 | 1 | 1 | 1 | 1 | 5 |
| Q6NSI4 | RPA-related protein RADX OS=Homo sapiens OX=9606 GN=RADX PE=1 SV=2 | 97,5 | 8,41 | 325 | 22 | 1 | 1 | 1 | 1 | 1 | 1 |
| Q9BYN8 | 28S ribosomal protein S26, mitochondrial OS=Homo sapiens OX=9606 GN=MRPS26 PE=1 SV=1 | 24,2 | 10,39 | 569 | 22 | 1 | 1 | 1 | 1 | 1 | 5 |
| O60762 | Dolichol-phosphate mannosyltransferase subunit 1 OS=Homo sapiens OX=9606 GN=DPM1 PE=1 SV=1 | 29,6 | 9,57 | 634 | 21 | 1 | 1 | 1 | 1 | 1 | 8 |
| P05091 | Aldehyde dehydrogenase, mitochondrial OS=Homo sapiens OX=9606 GN=ALDH2 PE=1 SV=2 | 56,3 | 7,05 | 601 | 21 | 1 | 1 | 1 | 1 | 1 | 3 |
| P15170 | Eukaryotic peptide chain release factor GTP-binding subunit ERF3A OS=Homo sapiens OX=9606 GN=GSPT1 PE=1 SV=1 | 55,7 | 5,62 | 476 | 21 | 1 | 1 | 1 | 1 | 1 | 2 |
| P82663 | 28S ribosomal protein S25, mitochondrial OS=Homo sapiens OX=9606 GN=MRPS25 PE=1 SV=1 | 20,1 | 8,82 | 688 | 21 | 1 | 1 | 1 | 1 | 1 | 5 |
| Q4G0J3 | La-related protein 7 OS=Homo sapiens OX=9606 GN=LARP7 PE=1 SV=1 | 66,9 | 9,55 | 387 | 21 | 1 | 1 | 1 | 1 | 1 | 2 |
| Q9HD33 | 39S ribosomal protein L47, mitochondrial OS=Homo sapiens OX=9606 GN=MRPL47 PE=1 SV=2 | 29,4 | 10,37 | 1032 | 21 | 1 | 1 | 1 | 1 | 1 | 4 |
| Q9Y4W2 | Ribosomal biogenesis protein LAS1L OS=Homo sapiens OX=9606 GN=LAS1L PE=1 SV=2 | 83 | 4,73 | 1002 | 21 | 1 | 1 | 1 | 1 | 1 | 2 |
| **Influenza A virus (IAV)** | | | | | | | | | | | |
| **ACCESSION** | **DESCRIPTION** | **MW [kDa]** | **calc. pI** | **Protein Group IDs** | **Score Mascot: Mascot** | **#PSMs (by Search Engine): Mascot** | **#Peptides (by Search Engine): Mascot** | **#PSMs** | **#Peptides** | **#Unique Peptides** | **Coverage [%]** |
| O95071 | E3 ubiquitin-protein ligase UBR5 OS=Homo sapiens OX=9606 GN=UBR5 PE=1 SV=2 | 309,2 | 5,85 | 907 | 1484 | 35 | 31 | 35 | 31 | 31 | 14 |
| P27708 | CAD protein OS=Homo sapiens OX=9606 GN=CAD PE=1 SV=3 | 242,8 | 6,46 | 206 | 1422 | 34 | 32 | 34 | 32 | 32 | 19 |
| P02538 | Keratin, type II cytoskeletal 6A OS=Homo sapiens OX=9606 GN=KRT6A PE=1 SV=3 | 60 | 8 | 343 | 1248 | 32 | 25 | 32 | 25 | 4 | 40 |
| Q9BYJ4 | **E3 ubiquitin-protein ligase TRIM34** OS=Homo sapiens OX=9606 GN=TRIM34 PE=1 SV=2 | 56,8 | 7,44 | 112 | 1210 | 68 | 24 | 68 | 24 | 24 | 43 |
| P19013 | Keratin, type II cytoskeletal 4 OS=Homo sapiens OX=9606 GN=KRT4 PE=1 SV=5 | 56,1 | 6,61 | 1033 | 1063 | 22 | 20 | 22 | 20 | 13 | 39 |
| O14654 | Insulin receptor substrate 4 OS=Homo sapiens OX=9606 GN=IRS4 PE=1 SV=1 | 133,7 | 8,44 | 746 | 848 | 20 | 19 | 20 | 19 | 19 | 21 |
| Q13885 | Tubulin beta-2A chain OS=Homo sapiens OX=9606 GN=TUBB2A PE=1 SV=1 | 49,9 | 4,89 | 134 | 801 | 32 | 14 | 32 | 14 | 1 | 39 |
| P10809 | 60 kDa heat shock protein, mitochondrial OS=Homo sapiens OX=9606 GN=HSPD1 PE=1 SV=2 | 61 | 5,87 | 928 | 788 | 16 | 15 | 16 | 15 | 15 | 34 |
| Q04695 | Keratin, type I cytoskeletal 17 OS=Homo sapiens OX=9606 GN=KRT17 PE=1 SV=2 | 48,1 | 5,02 | 929 | 762 | 27 | 15 | 27 | 15 | 4 | 30 |
| P13646 | Keratin, type I cytoskeletal 13 OS=Homo sapiens OX=9606 GN=KRT13 PE=1 SV=4 | 49,6 | 4,96 | 916 | 745 | 28 | 16 | 28 | 16 | 12 | 36 |
| Q9BQE3 | Tubulin alpha-1C chain OS=Homo sapiens OX=9606 GN=TUBA1C PE=1 SV=1 | 49,9 | 5,1 | 976 | 738 | 22 | 16 | 22 | 16 | 6 | 45 |
| Q7Z6Z7 | E3 ubiquitin-protein ligase HUWE1 OS=Homo sapiens OX=9606 GN=HUWE1 PE=1 SV=3 | 481,6 | 5,22 | 1045 | 718 | 19 | 19 | 19 | 19 | 19 | 5 |
| Q9Y230 | RuvB-like 2 OS=Homo sapiens OX=9606 GN=RUVBL2 PE=1 SV=3 | 51,1 | 5,64 | 82 | 701 | 17 | 16 | 17 | 16 | 16 | 38 |
| Q13200 | 26S proteasome non-ATPase regulatory subunit 2 OS=Homo sapiens OX=9606 GN=PSMD2 PE=1 SV=3 | 100,1 | 5,2 | 203 | 683 | 15 | 15 | 15 | 15 | 15 | 21 |
| P11021 | Endoplasmic reticulum chaperone BiP OS=Homo sapiens OX=9606 GN=HSPA5 PE=1 SV=2 | 72,3 | 5,16 | 619 | 678 | 16 | 13 | 16 | 13 | 11 | 26 |
| Q02413 | Desmoglein-1 OS=Homo sapiens OX=9606 GN=DSG1 PE=1 SV=2 | 113,7 | 5,03 | 454 | 646 | 15 | 12 | 15 | 12 | 12 | 16 |
| P08238 | Heat shock protein HSP 90-beta OS=Homo sapiens OX=9606 GN=HSP90AB1 PE=1 SV=4 | 83,2 | 5,03 | 429 | 611 | 17 | 14 | 17 | 14 | 7 | 23 |
| P68366 | Tubulin alpha-4A chain OS=Homo sapiens OX=9606 GN=TUBA4A PE=1 SV=1 | 49,9 | 5,06 | 1115 | 607 | 20 | 14 | 20 | 14 | 4 | 42 |
| P12035 | Keratin, type II cytoskeletal 3 OS=Homo sapiens OX=9606 GN=KRT3 PE=1 SV=3 | 64,4 | 6,48 | 611 | 586 | 15 | 12 | 15 | 12 | 1 | 13 |
| Q99460 | 26S proteasome non-ATPase regulatory subunit 1 OS=Homo sapiens OX=9606 GN=PSMD1 PE=1 SV=2 | 105,8 | 5,39 | 286 | 562 | 13 | 13 | 13 | 13 | 13 | 17 |
| P52272 | Heterogeneous nuclear ribonucleoprotein M OS=Homo sapiens OX=9606 GN=HNRNPM PE=1 SV=3 | 77,5 | 8,7 | 940 | 560 | 12 | 12 | 12 | 12 | 12 | 19 |
| Q15154 | Pericentriolar material 1 protein OS=Homo sapiens OX=9606 GN=PCM1 PE=1 SV=5 | 228,4 | 5,02 | 808 | 522 | 13 | 13 | 13 | 13 | 13 | 10 |
| P31943 | Heterogeneous nuclear ribonucleoprotein H OS=Homo sapiens OX=9606 GN=HNRNPH1 PE=1 SV=4 | 49,2 | 6,3 | 504 | 503 | 13 | 12 | 13 | 12 | 6 | 35 |
| Q9NR30 | Nucleolar RNA helicase 2 OS=Homo sapiens OX=9606 GN=DDX21 PE=1 SV=5 | 87,3 | 9,28 | 92 | 498 | 12 | 12 | 12 | 12 | 12 | 17 |
| Q14204 | Cytoplasmic dynein 1 heavy chain 1 OS=Homo sapiens OX=9606 GN=DYNC1H1 PE=1 SV=5 | 532,1 | 6,4 | 421 | 488 | 16 | 15 | 16 | 15 | 15 | 4 |
| Q9NZI8 | Insulin-like growth factor 2 mRNA-binding protein 1 OS=Homo sapiens OX=9606 GN=IGF2BP1 PE=1 SV=2 | 63,4 | 9,2 | 68 | 471 | 8 | 8 | 8 | 8 | 6 | 17 |
| P14868 | Aspartate--tRNA ligase, cytoplasmic OS=Homo sapiens OX=9606 GN=DARS1 PE=1 SV=2 | 57,1 | 6,55 | 871 | 468 | 12 | 12 | 12 | 12 | 12 | 29 |
| P13639 | Elongation factor 2 OS=Homo sapiens OX=9606 GN=EEF2 PE=1 SV=4 | 95,3 | 6,83 | 817 | 465 | 10 | 10 | 10 | 10 | 10 | 14 |
| P36578 | 60S ribosomal protein L4 OS=Homo sapiens OX=9606 GN=RPL4 PE=1 SV=5 | 47,7 | 11,06 | 326 | 465 | 13 | 11 | 13 | 11 | 11 | 28 |
| P17844 | Probable ATP-dependent RNA helicase DDX5 OS=Homo sapiens OX=9606 GN=DDX5 PE=1 SV=1 | 69,1 | 8,92 | 915 | 458 | 14 | 12 | 14 | 12 | 9 | 20 |
| Q7Z406 | Myosin-14 OS=Homo sapiens OX=9606 GN=MYH14 PE=1 SV=2 | 227,7 | 5,6 | 944 | 457 | 10 | 10 | 10 | 10 | 2 | 5 |
| P49368 | T-complex protein 1 subunit gamma OS=Homo sapiens OX=9606 GN=CCT3 PE=1 SV=4 | 60,5 | 6,49 | 999 | 442 | 10 | 10 | 10 | 10 | 10 | 19 |
| P31689 | DnaJ homolog subfamily A member 1 OS=Homo sapiens OX=9606 GN=DNAJA1 PE=1 SV=2 | 44,8 | 7,08 | 753 | 438 | 11 | 9 | 11 | 9 | 9 | 29 |
| O00571 | ATP-dependent RNA helicase DDX3X OS=Homo sapiens OX=9606 GN=DDX3X PE=1 SV=3 | 73,2 | 7,18 | 93 | 431 | 12 | 10 | 12 | 10 | 9 | 19 |
| P25205 | DNA replication licensing factor MCM3 OS=Homo sapiens OX=9606 GN=MCM3 PE=1 SV=3 | 90,9 | 5,77 | 304 | 430 | 12 | 11 | 12 | 11 | 11 | 16 |
| Q9UJV9 | Probable ATP-dependent RNA helicase DDX41 OS=Homo sapiens OX=9606 GN=DDX41 PE=1 SV=2 | 69,8 | 6,84 | 139 | 415 | 9 | 9 | 9 | 9 | 9 | 18 |
| P16615 | Sarcoplasmic/endoplasmic reticulum calcium ATPase 2 OS=Homo sapiens OX=9606 GN=ATP2A2 PE=1 SV=1 | 114,7 | 5,34 | 657 | 403 | 10 | 9 | 10 | 9 | 9 | 10 |
| Q16875 | 6-phosphofructo-2-kinase/fructose-2,6-bisphosphatase 3 OS=Homo sapiens OX=9606 GN=PFKFB3 PE=1 SV=1 | 59,6 | 8,21 | 935 | 400 | 10 | 10 | 10 | 10 | 9 | 23 |
| P07355 | Annexin A2 OS=Homo sapiens OX=9606 GN=ANXA2 PE=1 SV=2 | 38,6 | 7,75 | 583 | 399 | 10 | 10 | 10 | 10 | 10 | 32 |
| P46379 | Large proline-rich protein BAG6 OS=Homo sapiens OX=9606 GN=BAG6 PE=1 SV=2 | 119,3 | 5,6 | 1096 | 397 | 12 | 11 | 12 | 11 | 11 | 13 |
| O75643 | U5 small nuclear ribonucleoprotein 200 kDa helicase OS=Homo sapiens OX=9606 GN=SNRNP200 PE=1 SV=2 | 244,4 | 6,06 | 964 | 389 | 9 | 8 | 9 | 8 | 8 | 4 |
| Q92841 | Probable ATP-dependent RNA helicase DDX17 OS=Homo sapiens OX=9606 GN=DDX17 PE=1 SV=2 | 80,2 | 8,27 | 702 | 377 | 11 | 9 | 11 | 9 | 6 | 14 |
| P12236 | ADP/ATP translocase 3 OS=Homo sapiens OX=9606 GN=SLC25A6 PE=1 SV=4 | 32,8 | 9,74 | 125 | 358 | 11 | 9 | 11 | 9 | 2 | 28 |
| P41252 | Isoleucine--tRNA ligase, cytoplasmic OS=Homo sapiens OX=9606 GN=IARS1 PE=1 SV=2 | 144,4 | 6,15 | 882 | 349 | 9 | 9 | 9 | 9 | 9 | 8 |
| P04843 | Dolichyl-diphosphooligosaccharide--protein glycosyltransferase subunit 1 OS=Homo sapiens OX=9606 GN=RPN1 PE=1 SV=1 | 68,5 | 6,38 | 731 | 342 | 9 | 9 | 9 | 9 | 9 | 18 |
| P05023 | Sodium/potassium-transporting ATPase subunit alpha-1 OS=Homo sapiens OX=9606 GN=ATP1A1 PE=1 SV=1 | 112,8 | 5,49 | 438 | 342 | 8 | 8 | 8 | 8 | 8 | 10 |
| P20700 | Lamin-B1 OS=Homo sapiens OX=9606 GN=LMNB1 PE=1 SV=2 | 66,4 | 5,16 | 390 | 339 | 9 | 9 | 9 | 9 | 8 | 17 |
| Q9BUF5 | Tubulin beta-6 chain OS=Homo sapiens OX=9606 GN=TUBB6 PE=1 SV=1 | 49,8 | 4,88 | 484 | 334 | 14 | 7 | 14 | 7 | 1 | 16 |
| Q9NVI7 | ATPase family AAA domain-containing protein 3A OS=Homo sapiens OX=9606 GN=ATAD3A PE=1 SV=2 | 71,3 | 8,98 | 1074 | 333 | 8 | 7 | 8 | 7 | 4 | 13 |
| P14923 | Junction plakoglobin OS=Homo sapiens OX=9606 GN=JUP PE=1 SV=3 | 81,7 | 6,14 | 628 | 327 | 8 | 8 | 8 | 8 | 8 | 12 |
| O43143 | ATP-dependent RNA helicase DHX15 OS=Homo sapiens OX=9606 GN=DHX15 PE=1 SV=2 | 90,9 | 7,46 | 908 | 326 | 7 | 7 | 7 | 7 | 7 | 9 |
| P14618 | Pyruvate kinase PKM OS=Homo sapiens OX=9606 GN=PKM PE=1 SV=4 | 57,9 | 7,84 | 970 | 326 | 9 | 9 | 9 | 9 | 9 | 25 |
| Q9P2R3 | Rabankyrin-5 OS=Homo sapiens OX=9606 GN=ANKFY1 PE=1 SV=2 | 128,3 | 6,1 | 854 | 326 | 7 | 7 | 7 | 7 | 7 | 7 |
| O95793 | Double-stranded RNA-binding protein Staufen homolog 1 OS=Homo sapiens OX=9606 GN=STAU1 PE=1 SV=2 | 63,1 | 9,44 | 130 | 323 | 7 | 7 | 7 | 7 | 7 | 14 |
| Q6P2Q9 | Pre-mRNA-processing-splicing factor 8 OS=Homo sapiens OX=9606 GN=PRPF8 PE=1 SV=2 | 273,4 | 8,84 | 885 | 322 | 12 | 12 | 12 | 12 | 12 | 5 |
| P49411 | Elongation factor Tu, mitochondrial OS=Homo sapiens OX=9606 GN=TUFM PE=1 SV=3 | 49,8 | 7,61 | 293 | 320 | 9 | 9 | 9 | 9 | 9 | 25 |
| P35998 | 26S proteasome regulatory subunit 7 OS=Homo sapiens OX=9606 GN=PSMC2 PE=1 SV=3 | 48,6 | 5,95 | 124 | 319 | 7 | 7 | 7 | 7 | 7 | 17 |
| Q16531 | DNA damage-binding protein 1 OS=Homo sapiens OX=9606 GN=DDB1 PE=1 SV=1 | 126,9 | 5,26 | 138 | 314 | 8 | 8 | 8 | 8 | 8 | 9 |
| P16402 | Histone H1.3 OS=Homo sapiens OX=9606 GN=H1-3 PE=1 SV=2 | 22,3 | 11,02 | 729 | 310 | 8 | 7 | 8 | 7 | 1 | 24 |
| Q9UJS0 | Electrogenic aspartate/glutamate antiporter SLC25A13, mitochondrial OS=Homo sapiens OX=9606 GN=SLC25A13 PE=1 SV=2 | 74,1 | 8,62 | 767 | 308 | 9 | 8 | 9 | 8 | 8 | 14 |
| O43318 | Mitogen-activated protein kinase kinase kinase 7 OS=Homo sapiens OX=9606 GN=MAP3K7 PE=1 SV=1 | 67,2 | 7,11 | 357 | 286 | 7 | 7 | 7 | 7 | 7 | 14 |
| P12235 | ADP/ATP translocase 1 OS=Homo sapiens OX=9606 GN=SLC25A4 PE=1 SV=4 | 33 | 9,76 | 581 | 286 | 9 | 7 | 9 | 7 | 1 | 22 |
| P62258 | 14-3-3 protein epsilon OS=Homo sapiens OX=9606 GN=YWHAE PE=1 SV=1 | 29,2 | 4,74 | 1076 | 286 | 7 | 6 | 7 | 6 | 6 | 25 |
| Q9UHI6 | Probable ATP-dependent RNA helicase DDX20 OS=Homo sapiens OX=9606 GN=DDX20 PE=1 SV=2 | 92,2 | 6,95 | 209 | 286 | 7 | 7 | 7 | 7 | 7 | 10 |
| P17066 | Heat shock 70 kDa protein 6 OS=Homo sapiens OX=9606 GN=HSPA6 PE=1 SV=2 | 71 | 6,14 | 309 | 283 | 8 | 6 | 8 | 6 | 1 | 11 |
| P10515 | Dihydrolipoyllysine-residue acetyltransferase component of pyruvate dehydrogenase complex, mitochondrial OS=Homo sapiens OX=9606 GN=DLAT PE=1 SV=3 | 69 | 7,84 | 790 | 276 | 7 | 7 | 7 | 7 | 7 | 13 |
| P33993 | DNA replication licensing factor MCM7 OS=Homo sapiens OX=9606 GN=MCM7 PE=1 SV=4 | 81,3 | 6,46 | 546 | 272 | 9 | 8 | 9 | 8 | 8 | 15 |
| O60506 | Heterogeneous nuclear ribonucleoprotein Q OS=Homo sapiens OX=9606 GN=SYNCRIP PE=1 SV=2 | 69,6 | 8,59 | 539 | 267 | 8 | 7 | 8 | 7 | 4 | 15 |
| P31153 | S-adenosylmethionine synthase isoform type-2 OS=Homo sapiens OX=9606 GN=MAT2A PE=1 SV=1 | 43,6 | 6,48 | 456 | 265 | 7 | 6 | 7 | 6 | 6 | 18 |
| Q09028 | Histone-binding protein RBBP4 OS=Homo sapiens OX=9606 GN=RBBP4 PE=1 SV=3 | 47,6 | 4,89 | 972 | 265 | 5 | 5 | 5 | 5 | 1 | 12 |
| P27816 | Microtubule-associated protein 4 OS=Homo sapiens OX=9606 GN=MAP4 PE=1 SV=3 | 120,9 | 5,43 | 59 | 260 | 6 | 6 | 6 | 6 | 6 | 7 |
| Q92616 | eIF-2-alpha kinase activator GCN1 OS=Homo sapiens OX=9606 GN=GCN1 PE=1 SV=7 | 292,5 | 7,43 | 205 | 259 | 6 | 6 | 6 | 6 | 6 | 2 |
| P55036 | 26S proteasome non-ATPase regulatory subunit 4 OS=Homo sapiens OX=9606 GN=PSMD4 PE=1 SV=1 | 40,7 | 4,79 | 256 | 257 | 6 | 6 | 6 | 6 | 6 | 24 |
| P42167 | Lamina-associated polypeptide 2, isoforms beta/gamma OS=Homo sapiens OX=9606 GN=TMPO PE=1 SV=2 | 50,6 | 9,38 | 602 | 255 | 7 | 7 | 7 | 7 | 7 | 25 |
| P53621 | Coatomer subunit alpha OS=Homo sapiens OX=9606 GN=COPA PE=1 SV=2 | 138,3 | 7,66 | 975 | 253 | 6 | 6 | 6 | 6 | 6 | 5 |
| Q16576 | Histone-binding protein RBBP7 OS=Homo sapiens OX=9606 GN=RBBP7 PE=1 SV=1 | 47,8 | 5,05 | 71 | 252 | 5 | 5 | 5 | 5 | 1 | 12 |
| P48634 | Protein PRRC2A OS=Homo sapiens OX=9606 GN=PRRC2A PE=1 SV=3 | 228,7 | 9,45 | 508 | 250 | 7 | 6 | 7 | 6 | 6 | 5 |
| P55795 | Heterogeneous nuclear ribonucleoprotein H2 OS=Homo sapiens OX=9606 GN=HNRNPH2 PE=1 SV=1 | 49,2 | 6,3 | 1007 | 250 | 6 | 6 | 6 | 6 | 1 | 18 |
| Q14257 | Reticulocalbin-2 OS=Homo sapiens OX=9606 GN=RCN2 PE=1 SV=1 | 36,9 | 4,4 | 494 | 247 | 5 | 5 | 5 | 5 | 5 | 27 |
| Q9Y3I0 | RNA-splicing ligase RtcB homolog OS=Homo sapiens OX=9606 GN=RTCB PE=1 SV=1 | 55,2 | 7,23 | 402 | 245 | 7 | 7 | 7 | 7 | 7 | 15 |
| Q9Y3F4 | Serine-threonine kinase receptor-associated protein OS=Homo sapiens OX=9606 GN=STRAP PE=1 SV=1 | 38,4 | 5,12 | 847 | 244 | 7 | 5 | 7 | 5 | 5 | 21 |
| Q13347 | Eukaryotic translation initiation factor 3 subunit I OS=Homo sapiens OX=9606 GN=EIF3I PE=1 SV=1 | 36,5 | 5,64 | 85 | 239 | 5 | 5 | 5 | 5 | 5 | 18 |
| Q9UKV3 | Apoptotic chromatin condensation inducer in the nucleus OS=Homo sapiens OX=9606 GN=ACIN1 PE=1 SV=2 | 151,8 | 6,43 | 75 | 239 | 7 | 7 | 7 | 7 | 7 | 6 |
| P78316 | Nucleolar protein 14 OS=Homo sapiens OX=9606 GN=NOP14 PE=1 SV=3 | 97,6 | 7,58 | 850 | 238 | 6 | 6 | 6 | 6 | 6 | 8 |
| O00425 | Insulin-like growth factor 2 mRNA-binding protein 3 OS=Homo sapiens OX=9606 GN=IGF2BP3 PE=1 SV=2 | 63,7 | 8,87 | 604 | 237 | 5 | 5 | 5 | 5 | 3 | 11 |
| P30153 | Serine/threonine-protein phosphatase 2A 65 kDa regulatory subunit A alpha isoform OS=Homo sapiens OX=9606 GN=PPP2R1A PE=1 SV=4 | 65,3 | 5,11 | 536 | 235 | 5 | 5 | 5 | 5 | 5 | 8 |
| O95831 | Apoptosis-inducing factor 1, mitochondrial OS=Homo sapiens OX=9606 GN=AIFM1 PE=1 SV=1 | 66,9 | 8,95 | 231 | 231 | 6 | 6 | 6 | 6 | 6 | 11 |
| Q00325 | Phosphate carrier protein, mitochondrial OS=Homo sapiens OX=9606 GN=SLC25A3 PE=1 SV=2 | 40,1 | 9,38 | 1094 | 231 | 7 | 7 | 7 | 7 | 7 | 17 |
| P25788 | Proteasome subunit alpha type-3 OS=Homo sapiens OX=9606 GN=PSMA3 PE=1 SV=2 | 28,4 | 5,33 | 550 | 228 | 4 | 4 | 4 | 4 | 4 | 20 |
| Q96PK6 | RNA-binding protein 14 OS=Homo sapiens OX=9606 GN=RBM14 PE=1 SV=2 | 69,4 | 9,67 | 242 | 228 | 7 | 6 | 7 | 6 | 6 | 13 |
| P46781 | 40S ribosomal protein S9 OS=Homo sapiens OX=9606 GN=RPS9 PE=1 SV=3 | 22,6 | 10,65 | 844 | 227 | 7 | 6 | 7 | 6 | 6 | 22 |
| P23526 | Adenosylhomocysteinase OS=Homo sapiens OX=9606 GN=AHCY PE=1 SV=4 | 47,7 | 6,34 | 863 | 225 | 5 | 5 | 5 | 5 | 5 | 14 |
| Q93008 | Probable ubiquitin carboxyl-terminal hydrolase FAF-X OS=Homo sapiens OX=9606 GN=USP9X PE=1 SV=4 | 290,3 | 5,8 | 520 | 222 | 6 | 6 | 6 | 6 | 6 | 2 |
| O43242 | 26S proteasome non-ATPase regulatory subunit 3 OS=Homo sapiens OX=9606 GN=PSMD3 PE=1 SV=2 | 60,9 | 8,44 | 788 | 218 | 6 | 6 | 6 | 6 | 6 | 12 |
| P60842 | Eukaryotic initiation factor 4A-I OS=Homo sapiens OX=9606 GN=EIF4A1 PE=1 SV=1 | 46,1 | 5,48 | 474 | 215 | 5 | 5 | 5 | 5 | 3 | 16 |
| Q5T9A4 | ATPase family AAA domain-containing protein 3B OS=Homo sapiens OX=9606 GN=ATAD3B PE=1 SV=1 | 72,5 | 9,2 | 784 | 214 | 6 | 4 | 6 | 4 | 1 | 6 |
| P27635 | 60S ribosomal protein L10 OS=Homo sapiens OX=9606 GN=RPL10 PE=1 SV=5 | 24,6 | 10,08 | 1009 | 213 | 4 | 4 | 4 | 4 | 4 | 16 |
| P46977 | Dolichyl-diphosphooligosaccharide--protein glycosyltransferase subunit STT3A OS=Homo sapiens OX=9606 GN=STT3A PE=1 SV=2 | 80,5 | 8,07 | 18 | 211 | 5 | 5 | 5 | 5 | 4 | 6 |
| Q9P2J5 | Leucine--tRNA ligase, cytoplasmic OS=Homo sapiens OX=9606 GN=LARS1 PE=1 SV=2 | 134,4 | 7,3 | 403 | 211 | 7 | 7 | 7 | 7 | 7 | 7 |
| O14980 | Exportin-1 OS=Homo sapiens OX=9606 GN=XPO1 PE=1 SV=1 | 123,3 | 6,06 | 509 | 208 | 5 | 5 | 5 | 5 | 5 | 6 |
| Q13310 | Polyadenylate-binding protein 4 OS=Homo sapiens OX=9606 GN=PABPC4 PE=1 SV=1 | 70,7 | 9,26 | 437 | 205 | 6 | 5 | 6 | 5 | 2 | 10 |
| O00264 | Membrane-associated progesterone receptor component 1 OS=Homo sapiens OX=9606 GN=PGRMC1 PE=1 SV=3 | 21,7 | 4,7 | 639 | 204 | 5 | 5 | 5 | 5 | 5 | 34 |
| Q96T37 | RNA-binding protein 15 OS=Homo sapiens OX=9606 GN=RBM15 PE=1 SV=2 | 107,1 | 10,08 | 469 | 204 | 4 | 4 | 4 | 4 | 4 | 6 |
| P17858 | ATP-dependent 6-phosphofructokinase, liver type OS=Homo sapiens OX=9606 GN=PFKL PE=1 SV=6 | 85 | 7,5 | 690 | 203 | 5 | 5 | 5 | 5 | 2 | 7 |
| P45880 | Voltage-dependent anion-selective channel protein 2 OS=Homo sapiens OX=9606 GN=VDAC2 PE=1 SV=2 | 31,5 | 7,56 | 416 | 202 | 4 | 4 | 4 | 4 | 4 | 18 |
| Q15084 | Protein disulfide-isomerase A6 OS=Homo sapiens OX=9606 GN=PDIA6 PE=1 SV=1 | 48,1 | 5,08 | 946 | 202 | 3 | 3 | 3 | 3 | 3 | 9 |
| Q13263 | Transcription intermediary factor 1-beta OS=Homo sapiens OX=9606 GN=TRIM28 PE=1 SV=5 | 88,5 | 5,77 | 453 | 201 | 6 | 5 | 6 | 5 | 5 | 9 |
| Q99567 | Nuclear pore complex protein Nup88 OS=Homo sapiens OX=9606 GN=NUP88 PE=1 SV=2 | 83,5 | 5,69 | 64 | 201 | 5 | 5 | 5 | 5 | 5 | 9 |
| Q9UQ35 | Serine/arginine repetitive matrix protein 2 OS=Homo sapiens OX=9606 GN=SRRM2 PE=1 SV=2 | 299,4 | 12,06 | 500 | 200 | 6 | 6 | 6 | 6 | 6 | 3 |
| Q92598 | Heat shock protein 105 kDa OS=Homo sapiens OX=9606 GN=HSPH1 PE=1 SV=1 | 96,8 | 5,39 | 948 | 199 | 6 | 6 | 6 | 6 | 5 | 8 |
| O75592 | E3 ubiquitin-protein ligase MYCBP2 OS=Homo sapiens OX=9606 GN=MYCBP2 PE=1 SV=4 | 513,3 | 7,02 | 586 | 195 | 6 | 6 | 6 | 6 | 6 | 1 |
| O95229 | ZW10 interactor OS=Homo sapiens OX=9606 GN=ZWINT PE=1 SV=2 | 31,3 | 5,15 | 618 | 195 | 4 | 4 | 4 | 4 | 4 | 17 |
| O60825 | 6-phosphofructo-2-kinase/fructose-2,6-bisphosphatase 2 OS=Homo sapiens OX=9606 GN=PFKFB2 PE=1 SV=2 | 58,4 | 8,38 | 463 | 194 | 4 | 4 | 4 | 4 | 3 | 8 |
| Q9P035 | Very-long-chain (3R)-3-hydroxyacyl-CoA dehydratase 3 OS=Homo sapiens OX=9606 GN=HACD3 PE=1 SV=2 | 43,1 | 8,94 | 63 | 194 | 5 | 5 | 5 | 5 | 5 | 17 |
| Q01813 | ATP-dependent 6-phosphofructokinase, platelet type OS=Homo sapiens OX=9606 GN=PFKP PE=1 SV=2 | 85,5 | 7,55 | 440 | 192 | 5 | 5 | 5 | 5 | 2 | 8 |
| Q8IWX8 | Calcium homeostasis endoplasmic reticulum protein OS=Homo sapiens OX=9606 GN=CHERP PE=1 SV=3 | 103,6 | 9,04 | 258 | 191 | 6 | 6 | 6 | 6 | 6 | 7 |
| Q9H307 | Pinin OS=Homo sapiens OX=9606 GN=PNN PE=1 SV=5 | 81,6 | 7,14 | 939 | 190 | 6 | 6 | 6 | 6 | 6 | 9 |
| P28066 | Proteasome subunit alpha type-5 OS=Homo sapiens OX=9606 GN=PSMA5 PE=1 SV=3 | 26,4 | 4,79 | 383 | 189 | 4 | 4 | 4 | 4 | 4 | 20 |
| P48047 | ATP synthase subunit O, mitochondrial OS=Homo sapiens OX=9606 GN=ATP5PO PE=1 SV=1 | 23,3 | 9,96 | 874 | 189 | 5 | 5 | 5 | 5 | 5 | 26 |
| P62195 | 26S proteasome regulatory subunit 8 OS=Homo sapiens OX=9606 GN=PSMC5 PE=1 SV=1 | 45,6 | 7,55 | 1029 | 189 | 5 | 5 | 5 | 5 | 4 | 13 |
| Q9Y6Y0 | Influenza virus NS1A-binding protein OS=Homo sapiens OX=9606 GN=IVNS1ABP PE=1 SV=3 | 71,7 | 5,53 | 865 | 188 | 5 | 5 | 5 | 5 | 5 | 11 |
| P17980 | 26S proteasome regulatory subunit 6A OS=Homo sapiens OX=9606 GN=PSMC3 PE=1 SV=3 | 49,2 | 5,24 | 89 | 186 | 6 | 5 | 6 | 5 | 5 | 14 |
| P52292 | Importin subunit alpha-1 OS=Homo sapiens OX=9606 GN=KPNA2 PE=1 SV=1 | 57,8 | 5,4 | 287 | 186 | 3 | 3 | 3 | 3 | 3 | 9 |
| P22695 | Cytochrome b-c1 complex subunit 2, mitochondrial OS=Homo sapiens OX=9606 GN=UQCRC2 PE=1 SV=3 | 48,4 | 8,63 | 615 | 185 | 3 | 3 | 3 | 3 | 3 | 9 |
| P62191 | 26S proteasome regulatory subunit 4 OS=Homo sapiens OX=9606 GN=PSMC1 PE=1 SV=1 | 49,2 | 6,21 | 1051 | 184 | 5 | 5 | 5 | 5 | 4 | 13 |
| P08708 | 40S ribosomal protein S17 OS=Homo sapiens OX=9606 GN=RPS17 PE=1 SV=2 | 15,5 | 9,85 | 1026 | 183 | 5 | 4 | 5 | 4 | 4 | 33 |
| P47897 | Glutamine--tRNA ligase OS=Homo sapiens OX=9606 GN=QARS1 PE=1 SV=1 | 87,7 | 7,15 | 513 | 183 | 5 | 5 | 5 | 5 | 5 | 7 |
| Q9Y224 | RNA transcription, translation and transport factor protein OS=Homo sapiens OX=9606 GN=RTRAF PE=1 SV=1 | 28,1 | 6,65 | 122 | 183 | 5 | 4 | 5 | 4 | 4 | 20 |
| O15084 | Serine/threonine-protein phosphatase 6 regulatory ankyrin repeat subunit A OS=Homo sapiens OX=9606 GN=ANKRD28 PE=1 SV=5 | 112,9 | 6,25 | 1018 | 182 | 4 | 4 | 4 | 4 | 4 | 5 |
| P27824 | Calnexin OS=Homo sapiens OX=9606 GN=CANX PE=1 SV=2 | 67,5 | 4,6 | 730 | 182 | 5 | 5 | 5 | 5 | 5 | 11 |
| O43660 | Pleiotropic regulator 1 OS=Homo sapiens OX=9606 GN=PLRG1 PE=1 SV=1 | 57,2 | 9,17 | 1002 | 181 | 5 | 5 | 5 | 5 | 5 | 13 |
| P78406 | mRNA export factor RAE1 OS=Homo sapiens OX=9606 GN=RAE1 PE=1 SV=1 | 40,9 | 7,83 | 834 | 178 | 4 | 4 | 4 | 4 | 4 | 15 |
| P41091 | Eukaryotic translation initiation factor 2 subunit 3 OS=Homo sapiens OX=9606 GN=EIF2S3 PE=1 SV=3 | 51,1 | 8,4 | 640 | 177 | 5 | 4 | 5 | 4 | 4 | 11 |
| Q15008 | 26S proteasome non-ATPase regulatory subunit 6 OS=Homo sapiens OX=9606 GN=PSMD6 PE=1 SV=1 | 45,5 | 5,62 | 989 | 173 | 5 | 4 | 5 | 4 | 4 | 12 |
| P11586 | C-1-tetrahydrofolate synthase, cytoplasmic OS=Homo sapiens OX=9606 GN=MTHFD1 PE=1 SV=4 | 101,5 | 7,3 | 823 | 172 | 4 | 4 | 4 | 4 | 4 | 4 |
| Q8IXB1 | DnaJ homolog subfamily C member 10 OS=Homo sapiens OX=9606 GN=DNAJC10 PE=1 SV=2 | 91 | 7,18 | 958 | 171 | 5 | 5 | 5 | 5 | 5 | 6 |
| Q8WXF1 | Paraspeckle component 1 OS=Homo sapiens OX=9606 GN=PSPC1 PE=1 SV=1 | 58,7 | 6,67 | 340 | 171 | 5 | 5 | 5 | 5 | 5 | 12 |
| Q99615 | DnaJ homolog subfamily C member 7 OS=Homo sapiens OX=9606 GN=DNAJC7 PE=1 SV=2 | 56,4 | 6,96 | 171 | 171 | 4 | 4 | 4 | 4 | 4 | 9 |
| P62829 | 60S ribosomal protein L23 OS=Homo sapiens OX=9606 GN=RPL23 PE=1 SV=1 | 14,9 | 10,51 | 398 | 170 | 4 | 3 | 4 | 3 | 3 | 24 |
| Q13561 | Dynactin subunit 2 OS=Homo sapiens OX=9606 GN=DCTN2 PE=1 SV=4 | 44,2 | 5,21 | 41 | 170 | 5 | 5 | 5 | 5 | 5 | 18 |
| P07237 | Protein disulfide-isomerase OS=Homo sapiens OX=9606 GN=P4HB PE=1 SV=3 | 57,1 | 4,87 | 408 | 169 | 3 | 3 | 3 | 3 | 3 | 7 |
| Q9Y295 | Developmentally-regulated GTP-binding protein 1 OS=Homo sapiens OX=9606 GN=DRG1 PE=1 SV=1 | 40,5 | 8,9 | 407 | 169 | 4 | 4 | 4 | 4 | 4 | 13 |
| Q96A65 | Exocyst complex component 4 OS=Homo sapiens OX=9606 GN=EXOC4 PE=1 SV=1 | 110,4 | 6,49 | 432 | 167 | 4 | 4 | 4 | 4 | 4 | 6 |
| Q9NP73 | Putative bifunctional UDP-N-acetylglucosamine transferase and deubiquitinase ALG13 OS=Homo sapiens OX=9606 GN=ALG13 PE=1 SV=2 | 126 | 6,74 | 995 | 167 | 4 | 4 | 4 | 4 | 3 | 4 |
| Q9NNW5 | WD repeat-containing protein 6 OS=Homo sapiens OX=9606 GN=WDR6 PE=1 SV=1 | 121,6 | 6,87 | 1127 | 165 | 5 | 5 | 5 | 5 | 5 | 7 |
| Q9P258 | Protein RCC2 OS=Homo sapiens OX=9606 GN=RCC2 PE=1 SV=2 | 56 | 8,78 | 658 | 165 | 5 | 5 | 5 | 5 | 5 | 10 |
| P31040 | Succinate dehydrogenase [ubiquinone] flavoprotein subunit, mitochondrial OS=Homo sapiens OX=9606 GN=SDHA PE=1 SV=2 | 72,6 | 7,39 | 576 | 164 | 4 | 4 | 4 | 4 | 4 | 7 |
| O75821 | Eukaryotic translation initiation factor 3 subunit G OS=Homo sapiens OX=9606 GN=EIF3G PE=1 SV=2 | 35,6 | 6,13 | 797 | 163 | 4 | 4 | 4 | 4 | 4 | 18 |
| P04181 | Ornithine aminotransferase, mitochondrial OS=Homo sapiens OX=9606 GN=OAT PE=1 SV=1 | 48,5 | 7,03 | 1093 | 162 | 4 | 4 | 4 | 4 | 4 | 14 |
| P04844 | Dolichyl-diphosphooligosaccharide--protein glycosyltransferase subunit 2 OS=Homo sapiens OX=9606 GN=RPN2 PE=1 SV=3 | 69,2 | 5,69 | 406 | 162 | 4 | 4 | 4 | 4 | 4 | 9 |
| Q13835 | Plakophilin-1 OS=Homo sapiens OX=9606 GN=PKP1 PE=1 SV=2 | 82,8 | 9,13 | 557 | 162 | 3 | 3 | 3 | 3 | 3 | 4 |
| O00567 | Nucleolar protein 56 OS=Homo sapiens OX=9606 GN=NOP56 PE=1 SV=4 | 66 | 9,19 | 890 | 161 | 3 | 3 | 3 | 3 | 3 | 6 |
| P11908 | Ribose-phosphate pyrophosphokinase 2 OS=Homo sapiens OX=9606 GN=PRPS2 PE=1 SV=2 | 34,7 | 6,61 | 145 | 161 | 4 | 3 | 4 | 3 | 2 | 15 |
| Q96SB4 | SRSF protein kinase 1 OS=Homo sapiens OX=9606 GN=SRPK1 PE=1 SV=2 | 74,3 | 6,16 | 1055 | 161 | 3 | 3 | 3 | 3 | 3 | 5 |
| Q9Y5S9 | RNA-binding protein 8A OS=Homo sapiens OX=9606 GN=RBM8A PE=1 SV=1 | 19,9 | 5,72 | 296 | 160 | 4 | 3 | 4 | 3 | 3 | 32 |
| O95347 | Structural maintenance of chromosomes protein 2 OS=Homo sapiens OX=9606 GN=SMC2 PE=1 SV=2 | 135,6 | 8,43 | 1031 | 159 | 5 | 5 | 5 | 5 | 5 | 4 |
| P08195 | 4F2 cell-surface antigen heavy chain OS=Homo sapiens OX=9606 GN=SLC3A2 PE=1 SV=3 | 68 | 5,01 | 510 | 159 | 5 | 4 | 5 | 4 | 4 | 7 |
| P09661 | U2 small nuclear ribonucleoprotein A' OS=Homo sapiens OX=9606 GN=SNRPA1 PE=1 SV=2 | 28,4 | 8,62 | 911 | 159 | 4 | 4 | 4 | 4 | 4 | 18 |
| P39656 | Dolichyl-diphosphooligosaccharide--protein glycosyltransferase 48 kDa subunit OS=Homo sapiens OX=9606 GN=DDOST PE=1 SV=4 | 50,8 | 6,55 | 180 | 159 | 3 | 3 | 3 | 3 | 3 | 8 |
| Q07020 | 60S ribosomal protein L18 OS=Homo sapiens OX=9606 GN=RPL18 PE=1 SV=2 | 21,6 | 11,72 | 537 | 159 | 3 | 3 | 3 | 3 | 3 | 19 |
| Q9Y262 | Eukaryotic translation initiation factor 3 subunit L OS=Homo sapiens OX=9606 GN=EIF3L PE=1 SV=1 | 66,7 | 6,34 | 722 | 159 | 4 | 4 | 4 | 4 | 4 | 7 |
| Q69YN4 | Protein virilizer homolog OS=Homo sapiens OX=9606 GN=VIRMA PE=1 SV=2 | 201,9 | 5,01 | 986 | 157 | 5 | 5 | 5 | 5 | 5 | 3 |
| Q96EY1 | DnaJ homolog subfamily A member 3, mitochondrial OS=Homo sapiens OX=9606 GN=DNAJA3 PE=1 SV=2 | 52,5 | 9,26 | 136 | 157 | 6 | 4 | 6 | 4 | 4 | 11 |
| Q86VP6 | Cullin-associated NEDD8-dissociated protein 1 OS=Homo sapiens OX=9606 GN=CAND1 PE=1 SV=2 | 136,3 | 5,78 | 40 | 156 | 5 | 5 | 5 | 5 | 5 | 5 |
| O43592 | Exportin-T OS=Homo sapiens OX=9606 GN=XPOT PE=1 SV=2 | 109,9 | 5,39 | 1001 | 155 | 5 | 4 | 5 | 4 | 4 | 5 |
| P55209 | Nucleosome assembly protein 1-like 1 OS=Homo sapiens OX=9606 GN=NAP1L1 PE=1 SV=1 | 45,3 | 4,46 | 306 | 155 | 6 | 4 | 6 | 4 | 3 | 14 |
| P13489 | Ribonuclease inhibitor OS=Homo sapiens OX=9606 GN=RNH1 PE=1 SV=2 | 49,9 | 4,82 | 436 | 153 | 4 | 4 | 4 | 4 | 4 | 11 |
| P52597 | Heterogeneous nuclear ribonucleoprotein F OS=Homo sapiens OX=9606 GN=HNRNPF PE=1 SV=3 | 45,6 | 5,58 | 714 | 153 | 5 | 5 | 5 | 5 | 3 | 17 |
| P00367 | Glutamate dehydrogenase 1, mitochondrial OS=Homo sapiens OX=9606 GN=GLUD1 PE=1 SV=2 | 61,4 | 7,8 | 338 | 151 | 3 | 3 | 3 | 3 | 3 | 5 |
| P04637 | Cellular tumor antigen p53 OS=Homo sapiens OX=9606 GN=TP53 PE=1 SV=4 | 43,6 | 6,79 | 735 | 151 | 4 | 4 | 4 | 4 | 4 | 13 |
| P06702 | Protein S100-A9 OS=Homo sapiens OX=9606 GN=S100A9 PE=1 SV=1 | 13,2 | 6,13 | 534 | 151 | 3 | 3 | 3 | 3 | 3 | 20 |
| Q14203 | Dynactin subunit 1 OS=Homo sapiens OX=9606 GN=DCTN1 PE=1 SV=3 | 141,6 | 5,81 | 53 | 151 | 4 | 4 | 4 | 4 | 4 | 3 |
| O15294 | UDP-N-acetylglucosamine--peptide N-acetylglucosaminyltransferase 110 kDa subunit OS=Homo sapiens OX=9606 GN=OGT PE=1 SV=3 | 116,9 | 6,7 | 394 | 150 | 4 | 4 | 4 | 4 | 4 | 5 |
| P48594 | Serpin B4 OS=Homo sapiens OX=9606 GN=SERPINB4 PE=1 SV=2 | 44,8 | 6,21 | 56 | 149 | 3 | 3 | 3 | 3 | 1 | 8 |
| P63220 | 40S ribosomal protein S21 OS=Homo sapiens OX=9606 GN=RPS21 PE=1 SV=1 | 9,1 | 8,5 | 135 | 149 | 5 | 4 | 5 | 4 | 4 | 46 |
| Q53GQ0 | Very-long-chain 3-oxoacyl-CoA reductase OS=Homo sapiens OX=9606 GN=HSD17B12 PE=1 SV=2 | 34,3 | 9,32 | 385 | 149 | 4 | 4 | 4 | 4 | 4 | 14 |
| P00403 | Cytochrome c oxidase subunit 2 OS=Homo sapiens OX=9606 GN=MT-CO2 PE=1 SV=1 | 25,5 | 4,82 | 291 | 148 | 4 | 4 | 4 | 4 | 4 | 19 |
| Q96I25 | Splicing factor 45 OS=Homo sapiens OX=9606 GN=RBM17 PE=1 SV=1 | 44,9 | 5,97 | 1054 | 148 | 3 | 3 | 3 | 3 | 3 | 7 |
| Q14498 | RNA-binding protein 39 OS=Homo sapiens OX=9606 GN=RBM39 PE=1 SV=2 | 59,3 | 10,1 | 781 | 147 | 4 | 4 | 4 | 4 | 4 | 9 |
| P23458 | Tyrosine-protein kinase JAK1 OS=Homo sapiens OX=9606 GN=JAK1 PE=1 SV=2 | 133,2 | 7,55 | 838 | 146 | 4 | 4 | 4 | 4 | 4 | 4 |
| P62244 | 40S ribosomal protein S15a OS=Homo sapiens OX=9606 GN=RPS15A PE=1 SV=2 | 14,8 | 10,13 | 1022 | 146 | 4 | 4 | 4 | 4 | 4 | 28 |
| Q9Y277 | Voltage-dependent anion-selective channel protein 3 OS=Homo sapiens OX=9606 GN=VDAC3 PE=1 SV=1 | 30,6 | 8,66 | 862 | 146 | 4 | 4 | 4 | 4 | 3 | 17 |
| Q9Y3D9 | 28S ribosomal protein S23, mitochondrial OS=Homo sapiens OX=9606 GN=MRPS23 PE=1 SV=2 | 21,8 | 8,9 | 1034 | 146 | 3 | 3 | 3 | 3 | 3 | 16 |
| O76021 | Ribosomal L1 domain-containing protein 1 OS=Homo sapiens OX=9606 GN=RSL1D1 PE=1 SV=3 | 54,9 | 10,13 | 498 | 145 | 5 | 5 | 5 | 5 | 5 | 12 |
| Q9Y285 | Phenylalanine--tRNA ligase alpha subunit OS=Homo sapiens OX=9606 GN=FARSA PE=1 SV=3 | 57,5 | 7,8 | 1108 | 145 | 3 | 3 | 3 | 3 | 3 | 7 |
| O14818 | Proteasome subunit alpha type-7 OS=Homo sapiens OX=9606 GN=PSMA7 PE=1 SV=1 | 27,9 | 8,46 | 1039 | 144 | 4 | 4 | 4 | 4 | 4 | 14 |
| P12956 | X-ray repair cross-complementing protein 6 OS=Homo sapiens OX=9606 GN=XRCC6 PE=1 SV=2 | 69,8 | 6,64 | 713 | 144 | 4 | 4 | 4 | 4 | 4 | 8 |
| P30837 | Aldehyde dehydrogenase X, mitochondrial OS=Homo sapiens OX=9606 GN=ALDH1B1 PE=1 SV=4 | 57,2 | 6,99 | 1064 | 144 | 4 | 4 | 4 | 4 | 4 | 9 |
| P50402 | Emerin OS=Homo sapiens OX=9606 GN=EMD PE=1 SV=1 | 29 | 5,5 | 292 | 144 | 3 | 3 | 3 | 3 | 3 | 12 |
| Q08188 | Protein-glutamine gamma-glutamyltransferase E OS=Homo sapiens OX=9606 GN=TGM3 PE=1 SV=4 | 76,6 | 5,86 | 73 | 144 | 4 | 4 | 4 | 4 | 4 | 7 |
| P08237 | ATP-dependent 6-phosphofructokinase, muscle type OS=Homo sapiens OX=9606 GN=PFKM PE=1 SV=2 | 85,1 | 7,99 | 748 | 143 | 5 | 5 | 5 | 5 | 3 | 8 |
| P82650 | 28S ribosomal protein S22, mitochondrial OS=Homo sapiens OX=9606 GN=MRPS22 PE=1 SV=1 | 41,3 | 7,9 | 159 | 143 | 4 | 4 | 4 | 4 | 4 | 10 |
| Q9NY12 | H/ACA ribonucleoprotein complex subunit 1 OS=Homo sapiens OX=9606 GN=GAR1 PE=1 SV=1 | 22,3 | 10,92 | 27 | 143 | 3 | 3 | 3 | 3 | 3 | 13 |
| Q9Y399 | 28S ribosomal protein S2, mitochondrial OS=Homo sapiens OX=9606 GN=MRPS2 PE=1 SV=1 | 33,2 | 9,26 | 866 | 143 | 4 | 4 | 4 | 4 | 4 | 13 |
| Q08554 | Desmocollin-1 OS=Homo sapiens OX=9606 GN=DSC1 PE=1 SV=2 | 99,9 | 5,43 | 1069 | 142 | 3 | 3 | 3 | 3 | 3 | 4 |
| Q13363 | C-terminal-binding protein 1 OS=Homo sapiens OX=9606 GN=CTBP1 PE=1 SV=2 | 47,5 | 6,77 | 988 | 141 | 3 | 3 | 3 | 3 | 3 | 6 |
| Q15287 | RNA-binding protein with serine-rich domain 1 OS=Homo sapiens OX=9606 GN=RNPS1 PE=1 SV=1 | 34,2 | 11,84 | 978 | 141 | 4 | 4 | 4 | 4 | 4 | 14 |
| Q8N1F7 | Nuclear pore complex protein Nup93 OS=Homo sapiens OX=9606 GN=NUP93 PE=1 SV=2 | 93,4 | 5,72 | 1109 | 141 | 4 | 4 | 4 | 4 | 4 | 6 |
| Q9UHV9 | Prefoldin subunit 2 OS=Homo sapiens OX=9606 GN=PFDN2 PE=1 SV=1 | 16,6 | 6,58 | 413 | 141 | 2 | 2 | 2 | 2 | 2 | 17 |
| Q9Y520 | Protein PRRC2C OS=Homo sapiens OX=9606 GN=PRRC2C PE=1 SV=4 | 316,7 | 9,13 | 446 | 141 | 3 | 3 | 3 | 3 | 3 | 1 |
| P47756 | F-actin-capping protein subunit beta OS=Homo sapiens OX=9606 GN=CAPZB PE=1 SV=5 | 30,6 | 6 | 502 | 140 | 4 | 4 | 4 | 4 | 4 | 21 |
| Q99459 | Cell division cycle 5-like protein OS=Homo sapiens OX=9606 GN=CDC5L PE=1 SV=2 | 92,2 | 8,18 | 108 | 140 | 3 | 3 | 3 | 3 | 3 | 4 |
| O75369 | Filamin-B OS=Homo sapiens OX=9606 GN=FLNB PE=1 SV=2 | 278 | 5,73 | 806 | 138 | 4 | 4 | 4 | 4 | 1 | 1 |
| P68400 | Casein kinase II subunit alpha OS=Homo sapiens OX=9606 GN=CSNK2A1 PE=1 SV=1 | 45,1 | 7,74 | 842 | 138 | 3 | 3 | 3 | 3 | 3 | 9 |
| Q07955 | Serine/arginine-rich splicing factor 1 OS=Homo sapiens OX=9606 GN=SRSF1 PE=1 SV=2 | 27,7 | 10,36 | 845 | 138 | 3 | 3 | 3 | 3 | 3 | 13 |
| Q9BQG0 | Myb-binding protein 1A OS=Homo sapiens OX=9606 GN=MYBBP1A PE=1 SV=2 | 148,8 | 9,28 | 913 | 138 | 4 | 4 | 4 | 4 | 4 | 4 |
| Q9NTJ3 | Structural maintenance of chromosomes protein 4 OS=Homo sapiens OX=9606 GN=SMC4 PE=1 SV=2 | 147,1 | 6,79 | 339 | 138 | 4 | 4 | 4 | 4 | 4 | 3 |
| Q9UKM9 | RNA-binding protein Raly OS=Homo sapiens OX=9606 GN=RALY PE=1 SV=1 | 32,4 | 9,17 | 974 | 138 | 5 | 4 | 5 | 4 | 4 | 16 |
| P34932 | Heat shock 70 kDa protein 4 OS=Homo sapiens OX=9606 GN=HSPA4 PE=1 SV=4 | 94,3 | 5,19 | 532 | 137 | 4 | 4 | 4 | 4 | 4 | 7 |
| Q13428 | Treacle protein OS=Homo sapiens OX=9606 GN=TCOF1 PE=1 SV=3 | 152 | 9,04 | 1112 | 137 | 4 | 4 | 4 | 4 | 4 | 3 |
| P24534 | Elongation factor 1-beta OS=Homo sapiens OX=9606 GN=EEF1B2 PE=1 SV=3 | 24,7 | 4,67 | 672 | 136 | 2 | 2 | 2 | 2 | 2 | 11 |
| P25786 | Proteasome subunit alpha type-1 OS=Homo sapiens OX=9606 GN=PSMA1 PE=1 SV=1 | 29,5 | 6,61 | 224 | 136 | 3 | 3 | 3 | 3 | 3 | 13 |
| Q13868 | Exosome complex component RRP4 OS=Homo sapiens OX=9606 GN=EXOSC2 PE=1 SV=2 | 32,8 | 7,5 | 1041 | 136 | 3 | 3 | 3 | 3 | 3 | 13 |
| Q15029 | 116 kDa U5 small nuclear ribonucleoprotein component OS=Homo sapiens OX=9606 GN=EFTUD2 PE=1 SV=1 | 109,4 | 5 | 1083 | 136 | 4 | 4 | 4 | 4 | 4 | 6 |
| Q7L5D6 | Golgi to ER traffic protein 4 homolog OS=Homo sapiens OX=9606 GN=GET4 PE=1 SV=1 | 36,5 | 5,41 | 184 | 136 | 4 | 4 | 4 | 4 | 4 | 15 |
| O00231 | 26S proteasome non-ATPase regulatory subunit 11 OS=Homo sapiens OX=9606 GN=PSMD11 PE=1 SV=3 | 47,4 | 6,48 | 523 | 134 | 4 | 4 | 4 | 4 | 4 | 12 |
| P14649 | Myosin light chain 6B OS=Homo sapiens OX=9606 GN=MYL6B PE=1 SV=1 | 22,8 | 5,73 | 1044 | 134 | 3 | 3 | 3 | 3 | 2 | 17 |
| P60228 | Eukaryotic translation initiation factor 3 subunit E OS=Homo sapiens OX=9606 GN=EIF3E PE=1 SV=1 | 52,2 | 6,04 | 457 | 134 | 4 | 4 | 4 | 4 | 4 | 9 |
| Q15366 | Poly(rC)-binding protein 2 OS=Homo sapiens OX=9606 GN=PCBP2 PE=1 SV=1 | 38,6 | 6,79 | 515 | 134 | 3 | 3 | 3 | 3 | 1 | 12 |
| Q9ULX6 | A-kinase anchor protein 8-like OS=Homo sapiens OX=9606 GN=AKAP8L PE=1 SV=4 | 71,6 | 5,05 | 715 | 134 | 4 | 4 | 4 | 4 | 4 | 8 |
| P22234 | Bifunctional phosphoribosylaminoimidazole carboxylase/phosphoribosylaminoimidazole succinocarboxamide synthetase OS=Homo sapiens OX=9606 GN=PAICS PE=1 SV=3 | 47 | 7,23 | 490 | 133 | 3 | 3 | 3 | 3 | 3 | 9 |
| P54136 | Arginine--tRNA ligase, cytoplasmic OS=Homo sapiens OX=9606 GN=RARS1 PE=1 SV=2 | 75,3 | 6,68 | 681 | 133 | 4 | 4 | 4 | 4 | 4 | 7 |
| O95816 | BAG family molecular chaperone regulator 2 OS=Homo sapiens OX=9606 GN=BAG2 PE=1 SV=1 | 23,8 | 6,7 | 316 | 131 | 3 | 3 | 3 | 3 | 3 | 13 |
| P06733 | Alpha-enolase OS=Homo sapiens OX=9606 GN=ENO1 PE=1 SV=2 | 47,1 | 7,39 | 926 | 131 | 4 | 3 | 4 | 3 | 3 | 11 |
| P26196 | Probable ATP-dependent RNA helicase DDX6 OS=Homo sapiens OX=9606 GN=DDX6 PE=1 SV=2 | 54,4 | 8,66 | 47 | 131 | 4 | 4 | 4 | 4 | 4 | 12 |
| O94906 | Pre-mRNA-processing factor 6 OS=Homo sapiens OX=9606 GN=PRPF6 PE=1 SV=1 | 106,9 | 8,25 | 585 | 130 | 4 | 4 | 4 | 4 | 4 | 5 |
| P55884 | Eukaryotic translation initiation factor 3 subunit B OS=Homo sapiens OX=9606 GN=EIF3B PE=1 SV=3 | 92,4 | 5 | 791 | 130 | 4 | 4 | 4 | 4 | 4 | 5 |
| P61964 | WD repeat-containing protein 5 OS=Homo sapiens OX=9606 GN=WDR5 PE=1 SV=1 | 36,6 | 8,27 | 268 | 130 | 3 | 3 | 3 | 3 | 3 | 11 |
| P21796 | Voltage-dependent anion-selective channel protein 1 OS=Homo sapiens OX=9606 GN=VDAC1 PE=1 SV=2 | 30,8 | 8,54 | 503 | 129 | 4 | 4 | 4 | 4 | 3 | 15 |
| Q9BSD7 | Cancer-related nucleoside-triphosphatase OS=Homo sapiens OX=9606 GN=NTPCR PE=1 SV=1 | 20,7 | 9,54 | 1085 | 129 | 4 | 3 | 4 | 3 | 3 | 19 |
| Q9NWU5 | 39S ribosomal protein L22, mitochondrial OS=Homo sapiens OX=9606 GN=MRPL22 PE=1 SV=1 | 23,6 | 9,94 | 886 | 129 | 3 | 3 | 3 | 3 | 3 | 16 |
| Q14697 | Neutral alpha-glucosidase AB OS=Homo sapiens OX=9606 GN=GANAB PE=1 SV=3 | 106,8 | 6,14 | 380 | 128 | 3 | 3 | 3 | 3 | 3 | 4 |
| P36542 | ATP synthase subunit gamma, mitochondrial OS=Homo sapiens OX=9606 GN=ATP5F1C PE=1 SV=1 | 33 | 9,22 | 809 | 127 | 4 | 4 | 4 | 4 | 4 | 15 |
| P53007 | Tricarboxylate transport protein, mitochondrial OS=Homo sapiens OX=9606 GN=SLC25A1 PE=1 SV=2 | 34 | 9,89 | 574 | 127 | 4 | 4 | 4 | 4 | 4 | 14 |
| P57678 | Gem-associated protein 4 OS=Homo sapiens OX=9606 GN=GEMIN4 PE=1 SV=2 | 120 | 6,04 | 470 | 127 | 4 | 4 | 4 | 4 | 4 | 5 |
| P24666 | Low molecular weight phosphotyrosine protein phosphatase OS=Homo sapiens OX=9606 GN=ACP1 PE=1 SV=3 | 18 | 6,74 | 870 | 126 | 3 | 3 | 3 | 3 | 3 | 28 |
| Q9HB71 | Calcyclin-binding protein OS=Homo sapiens OX=9606 GN=CACYBP PE=1 SV=2 | 26,2 | 8,25 | 661 | 126 | 4 | 4 | 4 | 4 | 4 | 19 |
| P53618 | Coatomer subunit beta OS=Homo sapiens OX=9606 GN=COPB1 PE=1 SV=3 | 107,1 | 6,05 | 541 | 124 | 5 | 4 | 5 | 4 | 4 | 5 |
| Q9Y5M8 | Signal recognition particle receptor subunit beta OS=Homo sapiens OX=9606 GN=SRPRB PE=1 SV=3 | 29,7 | 9,04 | 174 | 124 | 3 | 3 | 3 | 3 | 3 | 17 |
| O00232 | 26S proteasome non-ATPase regulatory subunit 12 OS=Homo sapiens OX=9606 GN=PSMD12 PE=1 SV=3 | 52,9 | 7,65 | 404 | 123 | 3 | 3 | 3 | 3 | 3 | 6 |
| P55786 | Puromycin-sensitive aminopeptidase OS=Homo sapiens OX=9606 GN=NPEPPS PE=1 SV=2 | 103,2 | 5,72 | 830 | 123 | 4 | 4 | 4 | 4 | 4 | 5 |
| Q92552 | 28S ribosomal protein S27, mitochondrial OS=Homo sapiens OX=9606 GN=MRPS27 PE=1 SV=3 | 47,6 | 6,18 | 965 | 123 | 3 | 3 | 3 | 3 | 3 | 8 |
| P62140 | Serine/threonine-protein phosphatase PP1-beta catalytic subunit OS=Homo sapiens OX=9606 GN=PPP1CB PE=1 SV=3 | 37,2 | 6,19 | 873 | 121 | 4 | 3 | 4 | 3 | 1 | 11 |
| P05109 | Protein S100-A8 OS=Homo sapiens OX=9606 GN=S100A8 PE=1 SV=1 | 10,8 | 7,03 | 315 | 120 | 4 | 3 | 4 | 3 | 3 | 31 |
| P29966 | Myristoylated alanine-rich C-kinase substrate OS=Homo sapiens OX=9606 GN=MARCKS PE=1 SV=4 | 31,5 | 4,45 | 1063 | 120 | 3 | 3 | 3 | 3 | 3 | 20 |
| P61224 | Ras-related protein Rap-1b OS=Homo sapiens OX=9606 GN=RAP1B PE=1 SV=1 | 20,8 | 5,78 | 459 | 120 | 2 | 2 | 2 | 2 | 2 | 9 |
| P36957 | Dihydrolipoyllysine-residue succinyltransferase component of 2-oxoglutarate dehydrogenase complex, mitochondrial OS=Homo sapiens OX=9606 GN=DLST PE=1 SV=4 | 48,7 | 8,95 | 218 | 119 | 4 | 4 | 4 | 4 | 4 | 10 |
| P52907 | F-actin-capping protein subunit alpha-1 OS=Homo sapiens OX=9606 GN=CAPZA1 PE=1 SV=3 | 32,9 | 5,69 | 1012 | 119 | 3 | 3 | 3 | 3 | 3 | 13 |
| Q9Y2X3 | Nucleolar protein 58 OS=Homo sapiens OX=9606 GN=NOP58 PE=1 SV=1 | 59,5 | 8,92 | 150 | 119 | 3 | 3 | 3 | 3 | 3 | 8 |
| P63208 | S-phase kinase-associated protein 1 OS=Homo sapiens OX=9606 GN=SKP1 PE=1 SV=2 | 18,6 | 4,54 | 697 | 118 | 2 | 2 | 2 | 2 | 2 | 12 |
| Q9Y2R9 | 28S ribosomal protein S7, mitochondrial OS=Homo sapiens OX=9606 GN=MRPS7 PE=1 SV=2 | 28,1 | 9,99 | 592 | 118 | 3 | 3 | 3 | 3 | 3 | 13 |
| P29508 | Serpin B3 OS=Homo sapiens OX=9606 GN=SERPINB3 PE=1 SV=2 | 44,5 | 6,81 | 165 | 117 | 3 | 3 | 3 | 3 | 1 | 8 |
| Q5H9R7 | Serine/threonine-protein phosphatase 6 regulatory subunit 3 OS=Homo sapiens OX=9606 GN=PPP6R3 PE=1 SV=2 | 97,6 | 4,6 | 877 | 117 | 3 | 3 | 3 | 3 | 3 | 4 |
| Q7Z2W4 | Zinc finger CCCH-type antiviral protein 1 OS=Homo sapiens OX=9606 GN=ZC3HAV1 PE=1 SV=3 | 101,4 | 8,4 | 61 | 117 | 4 | 4 | 4 | 4 | 4 | 6 |
| P46087 | Probable 28S rRNA (cytosine(4447)-C(5))-methyltransferase OS=Homo sapiens OX=9606 GN=NOP2 PE=1 SV=2 | 89,2 | 9,23 | 816 | 116 | 3 | 3 | 3 | 3 | 3 | 4 |
| P62805 | Histone H4 OS=Homo sapiens OX=9606 GN=H4C1 PE=1 SV=2 | 11,4 | 11,36 | 668 | 115 | 4 | 4 | 4 | 4 | 4 | 42 |
| Q5JPE7 | BOS complex subunit NOMO2 OS=Homo sapiens OX=9606 GN=NOMO2 PE=1 SV=1 | 139,4 | 5,76 | 237 | 115 | 3 | 3 | 3 | 3 | 3 | 3 |
| Q9BUJ2 | Heterogeneous nuclear ribonucleoprotein U-like protein 1 OS=Homo sapiens OX=9606 GN=HNRNPUL1 PE=1 SV=2 | 95,7 | 6,92 | 451 | 115 | 3 | 3 | 3 | 3 | 3 | 4 |
| P24539 | ATP synthase F(0) complex subunit B1, mitochondrial OS=Homo sapiens OX=9606 GN=ATP5PB PE=1 SV=2 | 28,9 | 9,36 | 31 | 114 | 4 | 3 | 4 | 3 | 3 | 14 |
| P42677 | 40S ribosomal protein S27 OS=Homo sapiens OX=9606 GN=RPS27 PE=1 SV=3 | 9,5 | 9,45 | 485 | 114 | 3 | 3 | 3 | 3 | 3 | 31 |
| O00178 | GTP-binding protein 1 OS=Homo sapiens OX=9606 GN=GTPBP1 PE=1 SV=3 | 72,4 | 8,34 | 232 | 113 | 2 | 2 | 2 | 2 | 2 | 3 |
| P62873 | Guanine nucleotide-binding protein G(I)/G(S)/G(T) subunit beta-1 OS=Homo sapiens OX=9606 GN=GNB1 PE=1 SV=3 | 37,4 | 6 | 468 | 113 | 3 | 3 | 3 | 3 | 3 | 8 |
| Q5T3I0 | G patch domain-containing protein 4 OS=Homo sapiens OX=9606 GN=GPATCH4 PE=1 SV=2 | 50,4 | 9,63 | 588 | 113 | 3 | 3 | 3 | 3 | 3 | 11 |
| Q9NZ01 | Very-long-chain enoyl-CoA reductase OS=Homo sapiens OX=9606 GN=TECR PE=1 SV=1 | 36 | 9,45 | 1021 | 113 | 3 | 3 | 3 | 3 | 3 | 9 |
| O15027 | Protein transport protein Sec16A OS=Homo sapiens OX=9606 GN=SEC16A PE=1 SV=4 | 251,7 | 5,8 | 708 | 112 | 4 | 4 | 4 | 4 | 4 | 2 |
| Q13867 | Bleomycin hydrolase OS=Homo sapiens OX=9606 GN=BLMH PE=1 SV=1 | 52,5 | 6,27 | 893 | 112 | 2 | 2 | 2 | 2 | 2 | 5 |
| P09543 | 2',3'-cyclic-nucleotide 3'-phosphodiesterase OS=Homo sapiens OX=9606 GN=CNP PE=1 SV=2 | 47,5 | 9,07 | 521 | 111 | 3 | 3 | 3 | 3 | 3 | 9 |
| P11177 | Pyruvate dehydrogenase E1 component subunit beta, mitochondrial OS=Homo sapiens OX=9606 GN=PDHB PE=1 SV=3 | 39,2 | 6,65 | 840 | 111 | 4 | 4 | 4 | 4 | 4 | 15 |
| P29692 | Elongation factor 1-delta OS=Homo sapiens OX=9606 GN=EEF1D PE=1 SV=5 | 31,1 | 5,01 | 25 | 111 | 2 | 2 | 2 | 2 | 2 | 13 |
| Q9UKD2 | mRNA turnover protein 4 homolog OS=Homo sapiens OX=9606 GN=MRTO4 PE=1 SV=2 | 27,5 | 8,29 | 373 | 111 | 3 | 3 | 3 | 3 | 3 | 14 |
| P13797 | Plastin-3 OS=Homo sapiens OX=9606 GN=PLS3 PE=1 SV=4 | 70,8 | 5,6 | 922 | 111 | 4 | 4 | 4 | 4 | 4 | 6 |
| Q7Z417 | FMR1-interacting protein NUFIP2 OS=Homo sapiens OX=9606 GN=NUFIP2 PE=1 SV=1 | 76,1 | 8,7 | 189 | 110 | 3 | 3 | 3 | 3 | 3 | 5 |
| Q92945 | Far upstream element-binding protein 2 OS=Homo sapiens OX=9606 GN=KHSRP PE=1 SV=4 | 73,1 | 7,3 | 599 | 110 | 3 | 3 | 3 | 3 | 3 | 6 |
| Q9BYG3 | MKI67 FHA domain-interacting nucleolar phosphoprotein OS=Homo sapiens OX=9606 GN=NIFK PE=1 SV=1 | 34,2 | 9,88 | 554 | 110 | 3 | 3 | 3 | 3 | 3 | 14 |
| Q9H7D7 | WD repeat-containing protein 26 OS=Homo sapiens OX=9606 GN=WDR26 PE=1 SV=3 | 72,1 | 6,16 | 665 | 110 | 2 | 2 | 2 | 2 | 2 | 3 |
| Q9UNE7 | E3 ubiquitin-protein ligase CHIP OS=Homo sapiens OX=9606 GN=STUB1 PE=1 SV=2 | 34,8 | 5,87 | 1024 | 110 | 3 | 3 | 3 | 3 | 3 | 12 |
| P09493 | Tropomyosin alpha-1 chain OS=Homo sapiens OX=9606 GN=TPM1 PE=1 SV=2 | 32,7 | 4,74 | 712 | 108 | 4 | 3 | 4 | 3 | 2 | 13 |
| P62333 | 26S proteasome regulatory subunit 10B OS=Homo sapiens OX=9606 GN=PSMC6 PE=1 SV=1 | 44,1 | 7,49 | 445 | 108 | 5 | 3 | 5 | 3 | 3 | 9 |
| Q7Z4H7 | HAUS augmin-like complex subunit 6 OS=Homo sapiens OX=9606 GN=HAUS6 PE=1 SV=2 | 108,6 | 6,47 | 522 | 108 | 3 | 3 | 3 | 3 | 3 | 4 |
| O75947 | ATP synthase subunit d, mitochondrial OS=Homo sapiens OX=9606 GN=ATP5PD PE=1 SV=3 | 18,5 | 5,3 | 487 | 107 | 3 | 3 | 3 | 3 | 3 | 25 |
| P08559 | Pyruvate dehydrogenase E1 component subunit alpha, somatic form, mitochondrial OS=Homo sapiens OX=9606 GN=PDHA1 PE=1 SV=3 | 43,3 | 8,06 | 202 | 107 | 3 | 3 | 3 | 3 | 3 | 8 |
| P48556 | 26S proteasome non-ATPase regulatory subunit 8 OS=Homo sapiens OX=9606 GN=PSMD8 PE=1 SV=2 | 39,6 | 9,7 | 37 | 107 | 3 | 3 | 3 | 3 | 3 | 7 |
| Q92665 | 28S ribosomal protein S31, mitochondrial OS=Homo sapiens OX=9606 GN=MRPS31 PE=1 SV=3 | 45,3 | 9,29 | 891 | 107 | 3 | 3 | 3 | 3 | 3 | 8 |
| P51571 | Translocon-associated protein subunit delta OS=Homo sapiens OX=9606 GN=SSR4 PE=1 SV=1 | 19 | 6,15 | 392 | 106 | 2 | 2 | 2 | 2 | 2 | 14 |
| O96019 | Actin-like protein 6A OS=Homo sapiens OX=9606 GN=ACTL6A PE=1 SV=1 | 47,4 | 5,6 | 785 | 104 | 3 | 3 | 3 | 3 | 3 | 8 |
| Q13601 | KRR1 small subunit processome component homolog OS=Homo sapiens OX=9606 GN=KRR1 PE=1 SV=4 | 43,6 | 9,77 | 1013 | 104 | 2 | 2 | 2 | 2 | 2 | 6 |
| Q96P70 | Importin-9 OS=Homo sapiens OX=9606 GN=IPO9 PE=1 SV=3 | 115,9 | 4,81 | 937 | 104 | 3 | 3 | 3 | 3 | 3 | 4 |
| P62854 | 40S ribosomal protein S26 OS=Homo sapiens OX=9606 GN=RPS26 PE=1 SV=3 | 13 | 11 | 811 | 104 | 2 | 2 | 2 | 2 | 2 | 21 |
| P18085 | ADP-ribosylation factor 4 OS=Homo sapiens OX=9606 GN=ARF4 PE=1 SV=3 | 20,5 | 7,14 | 282 | 103 | 3 | 3 | 3 | 3 | 3 | 18 |
| P35520 | Cystathionine beta-synthase OS=Homo sapiens OX=9606 GN=CBS PE=1 SV=2 | 60,5 | 6,65 | 153 | 103 | 3 | 3 | 3 | 3 | 3 | 7 |
| P62937 | Peptidyl-prolyl cis-trans isomerase A OS=Homo sapiens OX=9606 GN=PPIA PE=1 SV=2 | 18 | 7,81 | 716 | 103 | 3 | 3 | 3 | 3 | 3 | 18 |
| Q8WUD4 | Coiled-coil domain-containing protein 12 OS=Homo sapiens OX=9606 GN=CCDC12 PE=1 SV=1 | 19,2 | 7,34 | 747 | 103 | 3 | 3 | 3 | 3 | 3 | 23 |
| Q93009 | Ubiquitin carboxyl-terminal hydrolase 7 OS=Homo sapiens OX=9606 GN=USP7 PE=1 SV=2 | 128,2 | 5,55 | 447 | 103 | 3 | 3 | 3 | 3 | 3 | 2 |
| Q9NZB2 | Constitutive coactivator of PPAR-gamma-like protein 1 OS=Homo sapiens OX=9606 GN=FAM120A PE=1 SV=2 | 121,8 | 8,88 | 892 | 103 | 1 | 1 | 1 | 1 | 1 | 2 |
| Q14739 | Delta(14)-sterol reductase LBR OS=Homo sapiens OX=9606 GN=LBR PE=1 SV=2 | 70,7 | 9,36 | 261 | 102 | 3 | 3 | 3 | 3 | 3 | 6 |
| Q16186 | Proteasomal ubiquitin receptor ADRM1 OS=Homo sapiens OX=9606 GN=ADRM1 PE=1 SV=2 | 42,1 | 5,07 | 864 | 102 | 2 | 2 | 2 | 2 | 2 | 8 |
| P09211 | Glutathione S-transferase P OS=Homo sapiens OX=9606 GN=GSTP1 PE=1 SV=2 | 23,3 | 5,64 | 910 | 101 | 2 | 2 | 2 | 2 | 2 | 13 |
| P82933 | 28S ribosomal protein S9, mitochondrial OS=Homo sapiens OX=9606 GN=MRPS9 PE=1 SV=2 | 45,8 | 9,51 | 1038 | 101 | 2 | 2 | 2 | 2 | 2 | 6 |
| Q92499 | ATP-dependent RNA helicase DDX1 OS=Homo sapiens OX=9606 GN=DDX1 PE=1 SV=2 | 82,4 | 7,23 | 561 | 101 | 3 | 3 | 3 | 3 | 3 | 4 |
| Q96DI7 | U5 small nuclear ribonucleoprotein 40 kDa protein OS=Homo sapiens OX=9606 GN=SNRNP40 PE=1 SV=1 | 39,3 | 8,1 | 106 | 101 | 2 | 2 | 2 | 2 | 2 | 6 |
| P35250 | Replication factor C subunit 2 OS=Homo sapiens OX=9606 GN=RFC2 PE=1 SV=3 | 39,1 | 6,44 | 888 | 100 | 3 | 3 | 3 | 3 | 3 | 11 |
| Q10570 | Cleavage and polyadenylation specificity factor subunit 1 OS=Homo sapiens OX=9606 GN=CPSF1 PE=1 SV=2 | 160,8 | 6,4 | 424 | 100 | 3 | 3 | 3 | 3 | 3 | 3 |
| P35613 | Basigin OS=Homo sapiens OX=9606 GN=BSG PE=1 SV=2 | 42,2 | 5,66 | 959 | 99 | 3 | 3 | 3 | 3 | 3 | 11 |
| P37198 | Nuclear pore glycoprotein p62 OS=Homo sapiens OX=9606 GN=NUP62 PE=1 SV=3 | 53,2 | 5,31 | 763 | 99 | 3 | 3 | 3 | 3 | 3 | 7 |
| P40939 | Trifunctional enzyme subunit alpha, mitochondrial OS=Homo sapiens OX=9606 GN=HADHA PE=1 SV=2 | 82,9 | 9,04 | 591 | 99 | 2 | 2 | 2 | 2 | 2 | 3 |
| Q99873 | Protein arginine N-methyltransferase 1 OS=Homo sapiens OX=9606 GN=PRMT1 PE=1 SV=3 | 42,4 | 5,35 | 558 | 99 | 2 | 2 | 2 | 2 | 2 | 7 |
| O43324 | Eukaryotic translation elongation factor 1 epsilon-1 OS=Homo sapiens OX=9606 GN=EEF1E1 PE=1 SV=1 | 19,8 | 8,54 | 366 | 98 | 3 | 3 | 3 | 3 | 3 | 20 |
| P30101 | Protein disulfide-isomerase A3 OS=Homo sapiens OX=9606 GN=PDIA3 PE=1 SV=4 | 56,7 | 6,35 | 664 | 98 | 3 | 3 | 3 | 3 | 3 | 7 |
| Q8N983 | 39S ribosomal protein L43, mitochondrial OS=Homo sapiens OX=9606 GN=MRPL43 PE=1 SV=1 | 23,4 | 8,65 | 1101 | 98 | 2 | 2 | 2 | 2 | 2 | 8 |
| P49458 | Signal recognition particle 9 kDa protein OS=Homo sapiens OX=9606 GN=SRP9 PE=1 SV=2 | 10,1 | 7,97 | 360 | 97 | 2 | 2 | 2 | 2 | 2 | 26 |
| P67936 | Tropomyosin alpha-4 chain OS=Homo sapiens OX=9606 GN=TPM4 PE=1 SV=3 | 28,5 | 4,69 | 569 | 97 | 3 | 3 | 3 | 3 | 2 | 14 |
| P82921 | 28S ribosomal protein S21, mitochondrial OS=Homo sapiens OX=9606 GN=MRPS21 PE=1 SV=3 | 10,7 | 9,92 | 839 | 97 | 2 | 2 | 2 | 2 | 2 | 30 |
| Q969G3 | SWI/SNF-related matrix-associated actin-dependent regulator of chromatin subfamily E member 1 OS=Homo sapiens OX=9606 GN=SMARCE1 PE=1 SV=2 | 46,6 | 4,88 | 317 | 97 | 3 | 3 | 3 | 3 | 3 | 10 |
| Q96B26 | Exosome complex component RRP43 OS=Homo sapiens OX=9606 GN=EXOSC8 PE=1 SV=1 | 30 | 5,3 | 511 | 97 | 3 | 3 | 3 | 3 | 3 | 14 |
| Q9H6F5 | Coiled-coil domain-containing protein 86 OS=Homo sapiens OX=9606 GN=CCDC86 PE=1 SV=1 | 40,2 | 10,33 | 415 | 96 | 4 | 3 | 4 | 3 | 3 | 8 |
| P46777 | 60S ribosomal protein L5 OS=Homo sapiens OX=9606 GN=RPL5 PE=1 SV=3 | 34,3 | 9,72 | 761 | 96 | 3 | 3 | 3 | 3 | 3 | 12 |
| P57088 | Transmembrane protein 33 OS=Homo sapiens OX=9606 GN=TMEM33 PE=1 SV=2 | 28 | 9,7 | 266 | 95 | 2 | 2 | 2 | 2 | 2 | 9 |
| P60900 | Proteasome subunit alpha type-6 OS=Homo sapiens OX=9606 GN=PSMA6 PE=1 SV=1 | 27,4 | 6,76 | 598 | 95 | 3 | 3 | 3 | 3 | 3 | 15 |
| Q96SI9 | Spermatid perinuclear RNA-binding protein OS=Homo sapiens OX=9606 GN=STRBP PE=1 SV=1 | 73,6 | 8,72 | 869 | 94 | 3 | 3 | 3 | 3 | 1 | 5 |
| Q9BVP2 | Guanine nucleotide-binding protein-like 3 OS=Homo sapiens OX=9606 GN=GNL3 PE=1 SV=2 | 62 | 9,16 | 54 | 94 | 3 | 3 | 3 | 3 | 3 | 5 |
| P62820 | Ras-related protein Rab-1A OS=Homo sapiens OX=9606 GN=RAB1A PE=1 SV=3 | 22,7 | 6,21 | 42 | 93 | 2 | 2 | 2 | 2 | 2 | 8 |
| P14866 | Heterogeneous nuclear ribonucleoprotein L OS=Homo sapiens OX=9606 GN=HNRNPL PE=1 SV=2 | 64,1 | 8,22 | 733 | 92 | 3 | 3 | 3 | 3 | 3 | 7 |
| Q9H583 | HEAT repeat-containing protein 1 OS=Homo sapiens OX=9606 GN=HEATR1 PE=1 SV=3 | 242,2 | 6,54 | 931 | 92 | 2 | 2 | 2 | 2 | 2 | 1 |
| Q9UBM7 | 7-dehydrocholesterol reductase OS=Homo sapiens OX=9606 GN=DHCR7 PE=1 SV=1 | 54,5 | 8,7 | 930 | 92 | 3 | 3 | 3 | 3 | 3 | 6 |
| Q14558 | Phosphoribosyl pyrophosphate synthase-associated protein 1 OS=Homo sapiens OX=9606 GN=PRPSAP1 PE=1 SV=2 | 39,4 | 7,2 | 943 | 91 | 2 | 2 | 2 | 2 | 1 | 7 |
| Q9NVP1 | ATP-dependent RNA helicase DDX18 OS=Homo sapiens OX=9606 GN=DDX18 PE=1 SV=2 | 75,4 | 9,5 | 154 | 91 | 2 | 2 | 2 | 2 | 2 | 3 |
| P43686 | 26S proteasome regulatory subunit 6B OS=Homo sapiens OX=9606 GN=PSMC4 PE=1 SV=2 | 47,3 | 5,21 | 901 | 90 | 3 | 3 | 3 | 3 | 3 | 7 |
| P51114 | RNA-binding protein FXR1 OS=Homo sapiens OX=9606 GN=FXR1 PE=1 SV=3 | 69,7 | 6,15 | 475 | 90 | 2 | 2 | 2 | 2 | 2 | 3 |
| P55072 | Transitional endoplasmic reticulum ATPase OS=Homo sapiens OX=9606 GN=VCP PE=1 SV=4 | 89,3 | 5,26 | 147 | 90 | 3 | 3 | 3 | 3 | 3 | 3 |
| Q14974 | Importin subunit beta-1 OS=Homo sapiens OX=9606 GN=KPNB1 PE=1 SV=2 | 97,1 | 4,78 | 121 | 90 | 3 | 3 | 3 | 3 | 3 | 4 |
| Q5BKZ1 | DBIRD complex subunit ZNF326 OS=Homo sapiens OX=9606 GN=ZNF326 PE=1 SV=2 | 65,6 | 5,15 | 332 | 90 | 2 | 2 | 2 | 2 | 2 | 4 |
| Q8ND56 | Protein LSM14 homolog A OS=Homo sapiens OX=9606 GN=LSM14A PE=1 SV=3 | 50,5 | 9,52 | 201 | 90 | 2 | 2 | 2 | 2 | 2 | 4 |
| Q99714 | 3-hydroxyacyl-CoA dehydrogenase type-2 OS=Homo sapiens OX=9606 GN=HSD17B10 PE=1 SV=3 | 26,9 | 7,78 | 751 | 90 | 3 | 3 | 3 | 3 | 3 | 14 |
| O75306 | NADH dehydrogenase [ubiquinone] iron-sulfur protein 2, mitochondrial OS=Homo sapiens OX=9606 GN=NDUFS2 PE=1 SV=2 | 52,5 | 7,55 | 544 | 89 | 2 | 2 | 2 | 2 | 2 | 6 |
| P26368 | Splicing factor U2AF 65 kDa subunit OS=Homo sapiens OX=9606 GN=U2AF2 PE=1 SV=4 | 53,5 | 9,09 | 777 | 88 | 2 | 2 | 2 | 2 | 2 | 4 |
| P26599 | Polypyrimidine tract-binding protein 1 OS=Homo sapiens OX=9606 GN=PTBP1 PE=1 SV=2 | 59,6 | 9,16 | 795 | 88 | 2 | 2 | 2 | 2 | 2 | 6 |
| P32969 | 60S ribosomal protein L9 OS=Homo sapiens OX=9606 GN=RPL9 PE=1 SV=1 | 21,9 | 9,95 | 529 | 88 | 2 | 2 | 2 | 2 | 2 | 13 |
| P81605 | Dermcidin OS=Homo sapiens OX=9606 GN=DCD PE=1 SV=2 | 11,3 | 6,54 | 887 | 88 | 2 | 2 | 2 | 2 | 2 | 20 |
| P51665 | 26S proteasome non-ATPase regulatory subunit 7 OS=Homo sapiens OX=9606 GN=PSMD7 PE=1 SV=2 | 37 | 6,77 | 320 | 87 | 3 | 3 | 3 | 3 | 3 | 7 |
| Q8N5F7 | NF-kappa-B-activating protein OS=Homo sapiens OX=9606 GN=NKAP PE=1 SV=1 | 47,1 | 10,11 | 1047 | 87 | 2 | 2 | 2 | 2 | 2 | 6 |
| Q9H9B4 | Sideroflexin-1 OS=Homo sapiens OX=9606 GN=SFXN1 PE=1 SV=4 | 35,6 | 9,07 | 779 | 87 | 3 | 2 | 3 | 2 | 2 | 7 |
| P06753 | Tropomyosin alpha-3 chain OS=Homo sapiens OX=9606 GN=TPM3 PE=1 SV=2 | 32,9 | 4,72 | 1046 | 86 | 2 | 2 | 2 | 2 | 1 | 9 |
| Q13573 | SNW domain-containing protein 1 OS=Homo sapiens OX=9606 GN=SNW1 PE=1 SV=1 | 61,5 | 9,52 | 601 | 86 | 3 | 3 | 3 | 3 | 3 | 7 |
| Q15459 | Splicing factor 3A subunit 1 OS=Homo sapiens OX=9606 GN=SF3A1 PE=1 SV=1 | 88,8 | 5,22 | 903 | 86 | 3 | 3 | 3 | 3 | 3 | 5 |
| Q9UBX3 | Mitochondrial dicarboxylate carrier OS=Homo sapiens OX=9606 GN=SLC25A10 PE=1 SV=2 | 31,3 | 9,54 | 701 | 85 | 2 | 2 | 2 | 2 | 2 | 6 |
| Q9UNF1 | Melanoma-associated antigen D2 OS=Homo sapiens OX=9606 GN=MAGED2 PE=1 SV=2 | 64,9 | 9,32 | 194 | 85 | 2 | 2 | 2 | 2 | 2 | 4 |
| P62273 | 40S ribosomal protein S29 OS=Homo sapiens OX=9606 GN=RPS29 PE=1 SV=2 | 6,7 | 10,13 | 241 | 85 | 3 | 2 | 3 | 2 | 2 | 32 |
| Q15758 | Neutral amino acid transporter B(0) OS=Homo sapiens OX=9606 GN=SLC1A5 PE=1 SV=2 | 56,6 | 5,48 | 151 | 84 | 2 | 2 | 2 | 2 | 2 | 4 |
| Q8IZP2 | Putative protein FAM10A4 OS=Homo sapiens OX=9606 GN=ST13P4 PE=5 SV=1 | 27,4 | 5,08 | 179 | 84 | 2 | 2 | 2 | 2 | 2 | 10 |
| P11387 | DNA topoisomerase 1 OS=Homo sapiens OX=9606 GN=TOP1 PE=1 SV=2 | 90,7 | 9,31 | 1126 | 83 | 2 | 2 | 2 | 2 | 2 | 2 |
| P14927 | Cytochrome b-c1 complex subunit 7 OS=Homo sapiens OX=9606 GN=UQCRB PE=1 SV=2 | 13,5 | 8,78 | 300 | 83 | 2 | 2 | 2 | 2 | 2 | 25 |
| Q99497 | Parkinson disease protein 7 OS=Homo sapiens OX=9606 GN=PARK7 PE=1 SV=2 | 19,9 | 6,79 | 1090 | 83 | 3 | 3 | 3 | 3 | 3 | 30 |
| P09012 | U1 small nuclear ribonucleoprotein A OS=Homo sapiens OX=9606 GN=SNRPA PE=1 SV=3 | 31,3 | 9,83 | 129 | 82 | 3 | 2 | 3 | 2 | 2 | 8 |
| P25787 | Proteasome subunit alpha type-2 OS=Homo sapiens OX=9606 GN=PSMA2 PE=1 SV=2 | 25,9 | 7,43 | 199 | 82 | 1 | 1 | 1 | 1 | 1 | 6 |
| P60468 | Protein transport protein Sec61 subunit beta OS=Homo sapiens OX=9606 GN=SEC61B PE=1 SV=2 | 10 | 11,56 | 555 | 82 | 2 | 2 | 2 | 2 | 2 | 22 |
| P61313 | 60S ribosomal protein L15 OS=Homo sapiens OX=9606 GN=RPL15 PE=1 SV=2 | 24,1 | 11,62 | 897 | 82 | 3 | 3 | 3 | 3 | 3 | 14 |
| Q9H0U6 | 39S ribosomal protein L18, mitochondrial OS=Homo sapiens OX=9606 GN=MRPL18 PE=1 SV=1 | 20,6 | 9,54 | 799 | 82 | 2 | 2 | 2 | 2 | 2 | 12 |
| O95757 | Heat shock 70 kDa protein 4L OS=Homo sapiens OX=9606 GN=HSPA4L PE=1 SV=3 | 94,5 | 5,88 | 308 | 81 | 2 | 2 | 2 | 2 | 1 | 3 |
| P09001 | 39S ribosomal protein L3, mitochondrial OS=Homo sapiens OX=9606 GN=MRPL3 PE=1 SV=1 | 38,6 | 9,48 | 444 | 81 | 3 | 3 | 3 | 3 | 3 | 10 |
| P28331 | NADH-ubiquinone oxidoreductase 75 kDa subunit, mitochondrial OS=Homo sapiens OX=9606 GN=NDUFS1 PE=1 SV=3 | 79,4 | 6,23 | 81 | 80 | 2 | 2 | 2 | 2 | 2 | 3 |
| P51398 | 28S ribosomal protein S29, mitochondrial OS=Homo sapiens OX=9606 GN=DAP3 PE=1 SV=1 | 45,5 | 8,88 | 354 | 80 | 3 | 2 | 3 | 2 | 2 | 8 |
| P04792 | Heat shock protein beta-1 OS=Homo sapiens OX=9606 GN=HSPB1 PE=1 SV=2 | 22,8 | 6,4 | 175 | 79 | 2 | 2 | 2 | 2 | 2 | 10 |
| P11310 | Medium-chain specific acyl-CoA dehydrogenase, mitochondrial OS=Homo sapiens OX=9606 GN=ACADM PE=1 SV=1 | 46,6 | 8,37 | 770 | 79 | 2 | 2 | 2 | 2 | 2 | 5 |
| P62699 | Protein yippee-like 5 OS=Homo sapiens OX=9606 GN=YPEL5 PE=1 SV=1 | 13,8 | 7,31 | 367 | 79 | 2 | 2 | 2 | 2 | 2 | 18 |
| Q12874 | Splicing factor 3A subunit 3 OS=Homo sapiens OX=9606 GN=SF3A3 PE=1 SV=1 | 58,8 | 5,38 | 590 | 79 | 2 | 2 | 2 | 2 | 2 | 4 |
| Q13283 | Ras GTPase-activating protein-binding protein 1 OS=Homo sapiens OX=9606 GN=G3BP1 PE=1 SV=1 | 52,1 | 5,52 | 170 | 79 | 2 | 2 | 2 | 2 | 2 | 6 |
| Q15024 | Exosome complex component RRP42 OS=Homo sapiens OX=9606 GN=EXOSC7 PE=1 SV=3 | 31,8 | 5,19 | 401 | 79 | 2 | 2 | 2 | 2 | 2 | 11 |
| Q9P2E9 | Ribosome-binding protein 1 OS=Homo sapiens OX=9606 GN=RRBP1 PE=1 SV=5 | 152,4 | 8,6 | 825 | 79 | 1 | 1 | 1 | 1 | 1 | 1 |
| P01040 | Cystatin-A OS=Homo sapiens OX=9606 GN=CSTA PE=1 SV=1 | 11 | 5,5 | 698 | 78 | 1 | 1 | 1 | 1 | 1 | 18 |
| P62841 | 40S ribosomal protein S15 OS=Homo sapiens OX=9606 GN=RPS15 PE=1 SV=2 | 17 | 10,39 | 393 | 78 | 2 | 2 | 2 | 2 | 2 | 21 |
| Q13409 | Cytoplasmic dynein 1 intermediate chain 2 OS=Homo sapiens OX=9606 GN=DYNC1I2 PE=1 SV=3 | 71,4 | 5,2 | 573 | 78 | 2 | 2 | 2 | 2 | 2 | 6 |
| O75489 | NADH dehydrogenase [ubiquinone] iron-sulfur protein 3, mitochondrial OS=Homo sapiens OX=9606 GN=NDUFS3 PE=1 SV=1 | 30,2 | 7,5 | 571 | 77 | 2 | 2 | 2 | 2 | 2 | 10 |
| Q9H0S4 | Probable ATP-dependent RNA helicase DDX47 OS=Homo sapiens OX=9606 GN=DDX47 PE=1 SV=1 | 50,6 | 9,1 | 238 | 77 | 1 | 1 | 1 | 1 | 1 | 4 |
| Q9NXS2 | Glutaminyl-peptide cyclotransferase-like protein OS=Homo sapiens OX=9606 GN=QPCTL PE=1 SV=2 | 42,9 | 9,82 | 33 | 77 | 2 | 2 | 2 | 2 | 2 | 6 |
| Q9UG63 | ATP-binding cassette sub-family F member 2 OS=Homo sapiens OX=9606 GN=ABCF2 PE=1 SV=2 | 71,2 | 7,37 | 734 | 77 | 2 | 2 | 2 | 2 | 2 | 4 |
| Q9Y5K5 | Ubiquitin carboxyl-terminal hydrolase isozyme L5 OS=Homo sapiens OX=9606 GN=UCHL5 PE=1 SV=3 | 37,6 | 5,33 | 358 | 77 | 2 | 2 | 2 | 2 | 2 | 5 |
| O15212 | Prefoldin subunit 6 OS=Homo sapiens OX=9606 GN=PFDN6 PE=1 SV=1 | 14,6 | 8,88 | 1052 | 76 | 2 | 2 | 2 | 2 | 2 | 16 |
| P67870 | Casein kinase II subunit beta OS=Homo sapiens OX=9606 GN=CSNK2B PE=1 SV=1 | 24,9 | 5,55 | 277 | 76 | 2 | 2 | 2 | 2 | 2 | 12 |
| Q9BRJ6 | Uncharacterized protein C7orf50 OS=Homo sapiens OX=9606 GN=C7orf50 PE=1 SV=1 | 22,1 | 9,64 | 198 | 76 | 3 | 2 | 3 | 2 | 2 | 19 |
| Q9UNQ2 | Probable dimethyladenosine transferase OS=Homo sapiens OX=9606 GN=DIMT1 PE=1 SV=1 | 35,2 | 9,99 | 636 | 76 | 2 | 2 | 2 | 2 | 2 | 6 |
| O75940 | Survival of motor neuron-related-splicing factor 30 OS=Homo sapiens OX=9606 GN=SMNDC1 PE=1 SV=1 | 26,7 | 7,24 | 120 | 75 | 2 | 2 | 2 | 2 | 2 | 9 |
| P05783 | Keratin, type I cytoskeletal 18 OS=Homo sapiens OX=9606 GN=KRT18 PE=1 SV=2 | 48 | 5,45 | 465 | 75 | 9 | 2 | 9 | 2 | 1 | 4 |
| P54727 | UV excision repair protein RAD23 homolog B OS=Homo sapiens OX=9606 GN=RAD23B PE=1 SV=1 | 43,1 | 4,84 | 1068 | 75 | 2 | 2 | 2 | 2 | 2 | 10 |
| Q16629 | Serine/arginine-rich splicing factor 7 OS=Homo sapiens OX=9606 GN=SRSF7 PE=1 SV=1 | 27,4 | 11,82 | 234 | 75 | 2 | 2 | 2 | 2 | 2 | 8 |
| Q16769 | Glutaminyl-peptide cyclotransferase OS=Homo sapiens OX=9606 GN=QPCT PE=1 SV=1 | 40,9 | 6,61 | 704 | 75 | 2 | 2 | 2 | 2 | 2 | 7 |
| Q99733 | Nucleosome assembly protein 1-like 4 OS=Homo sapiens OX=9606 GN=NAP1L4 PE=1 SV=1 | 42,8 | 4,69 | 617 | 75 | 2 | 2 | 2 | 2 | 1 | 7 |
| O15371 | Eukaryotic translation initiation factor 3 subunit D OS=Homo sapiens OX=9606 GN=EIF3D PE=1 SV=1 | 63,9 | 6,05 | 323 | 74 | 2 | 2 | 2 | 2 | 2 | 2 |
| P20618 | Proteasome subunit beta type-1 OS=Homo sapiens OX=9606 GN=PSMB1 PE=1 SV=2 | 26,5 | 8,13 | 276 | 74 | 2 | 2 | 2 | 2 | 2 | 10 |
| P25685 | DnaJ homolog subfamily B member 1 OS=Homo sapiens OX=9606 GN=DNAJB1 PE=1 SV=4 | 38 | 8,63 | 1130 | 74 | 2 | 2 | 2 | 2 | 2 | 6 |
| P52789 | Hexokinase-2 OS=Homo sapiens OX=9606 GN=HK2 PE=1 SV=2 | 102,3 | 6,05 | 221 | 74 | 3 | 3 | 3 | 3 | 3 | 4 |
| P55084 | Trifunctional enzyme subunit beta, mitochondrial OS=Homo sapiens OX=9606 GN=HADHB PE=1 SV=3 | 51,3 | 9,41 | 841 | 74 | 2 | 2 | 2 | 2 | 2 | 5 |
| P56385 | ATP synthase subunit e, mitochondrial OS=Homo sapiens OX=9606 GN=ATP5ME PE=1 SV=2 | 7,9 | 9,35 | 1079 | 74 | 2 | 2 | 2 | 2 | 2 | 30 |
| P61289 | Proteasome activator complex subunit 3 OS=Homo sapiens OX=9606 GN=PSME3 PE=1 SV=1 | 29,5 | 5,95 | 1086 | 74 | 1 | 1 | 1 | 1 | 1 | 4 |
| Q03252 | Lamin-B2 OS=Homo sapiens OX=9606 GN=LMNB2 PE=1 SV=4 | 69,9 | 5,59 | 656 | 74 | 2 | 2 | 2 | 2 | 1 | 3 |
| Q9NQT4 | Exosome complex component RRP46 OS=Homo sapiens OX=9606 GN=EXOSC5 PE=1 SV=1 | 25,2 | 7,59 | 1042 | 74 | 2 | 2 | 2 | 2 | 2 | 9 |
| Q9UPN9 | E3 ubiquitin-protein ligase TRIM33 OS=Homo sapiens OX=9606 GN=TRIM33 PE=1 SV=3 | 122,5 | 6,67 | 804 | 74 | 2 | 2 | 2 | 2 | 2 | 2 |
| P09496 | Clathrin light chain A OS=Homo sapiens OX=9606 GN=CLTA PE=1 SV=1 | 27,1 | 4,51 | 1104 | 73 | 2 | 2 | 2 | 2 | 2 | 6 |
| P09972 | Fructose-bisphosphate aldolase C OS=Homo sapiens OX=9606 GN=ALDOC PE=1 SV=2 | 39,4 | 6,87 | 248 | 73 | 2 | 2 | 2 | 2 | 1 | 9 |
| P63151 | Serine/threonine-protein phosphatase 2A 55 kDa regulatory subunit B alpha isoform OS=Homo sapiens OX=9606 GN=PPP2R2A PE=1 SV=1 | 51,7 | 6,2 | 1102 | 73 | 1 | 1 | 1 | 1 | 1 | 2 |
| Q13247 | Serine/arginine-rich splicing factor 6 OS=Homo sapiens OX=9606 GN=SRSF6 PE=1 SV=2 | 39,6 | 11,43 | 696 | 73 | 2 | 2 | 2 | 2 | 1 | 6 |
| Q14103 | Heterogeneous nuclear ribonucleoprotein D0 OS=Homo sapiens OX=9606 GN=HNRNPD PE=1 SV=1 | 38,4 | 7,81 | 831 | 73 | 2 | 2 | 2 | 2 | 2 | 6 |
| O75396 | Vesicle-trafficking protein SEC22b OS=Homo sapiens OX=9606 GN=SEC22B PE=1 SV=5 | 24,7 | 8,51 | 526 | 72 | 2 | 2 | 2 | 2 | 2 | 9 |
| P04080 | Cystatin-B OS=Homo sapiens OX=9606 GN=CSTB PE=1 SV=2 | 11,1 | 7,56 | 737 | 72 | 2 | 2 | 2 | 2 | 2 | 34 |
| Q8WXD5 | Gem-associated protein 6 OS=Homo sapiens OX=9606 GN=GEMIN6 PE=1 SV=1 | 18,8 | 5,12 | 329 | 72 | 2 | 2 | 2 | 2 | 2 | 16 |
| Q16891 | MICOS complex subunit MIC60 OS=Homo sapiens OX=9606 GN=IMMT PE=1 SV=1 | 83,6 | 6,48 | 1116 | 71 | 2 | 2 | 2 | 2 | 2 | 3 |
| Q9HCY8 | Protein S100-A14 OS=Homo sapiens OX=9606 GN=S100A14 PE=1 SV=1 | 11,7 | 5,24 | 627 | 71 | 1 | 1 | 1 | 1 | 1 | 14 |
| Q9NRX2 | 39S ribosomal protein L17, mitochondrial OS=Homo sapiens OX=9606 GN=MRPL17 PE=1 SV=1 | 20 | 10,11 | 982 | 71 | 2 | 2 | 2 | 2 | 2 | 10 |
| Q9Y3C1 | Nucleolar protein 16 OS=Homo sapiens OX=9606 GN=NOP16 PE=1 SV=2 | 21,2 | 9,94 | 410 | 71 | 2 | 2 | 2 | 2 | 2 | 12 |
| Q9Y4P3 | Transducin beta-like protein 2 OS=Homo sapiens OX=9606 GN=TBL2 PE=1 SV=1 | 49,8 | 9,44 | 252 | 71 | 1 | 1 | 1 | 1 | 1 | 3 |
| O14979 | Heterogeneous nuclear ribonucleoprotein D-like OS=Homo sapiens OX=9606 GN=HNRNPDL PE=1 SV=3 | 46,4 | 9,57 | 846 | 70 | 2 | 2 | 2 | 2 | 2 | 5 |
| P31946 | 14-3-3 protein beta/alpha OS=Homo sapiens OX=9606 GN=YWHAB PE=1 SV=3 | 28,1 | 4,83 | 141 | 70 | 2 | 2 | 2 | 2 | 2 | 9 |
| Q92621 | Nuclear pore complex protein Nup205 OS=Homo sapiens OX=9606 GN=NUP205 PE=1 SV=3 | 227,8 | 6,19 | 26 | 70 | 2 | 2 | 2 | 2 | 2 | 1 |
| Q9BQ67 | Glutamate-rich WD repeat-containing protein 1 OS=Homo sapiens OX=9606 GN=GRWD1 PE=1 SV=1 | 49,4 | 4,92 | 12 | 70 | 2 | 2 | 2 | 2 | 2 | 7 |
| P35249 | Replication factor C subunit 4 OS=Homo sapiens OX=9606 GN=RFC4 PE=1 SV=2 | 39,7 | 8,02 | 330 | 69 | 2 | 2 | 2 | 2 | 2 | 5 |
| Q7L0Y3 | tRNA methyltransferase 10 homolog C OS=Homo sapiens OX=9606 GN=TRMT10C PE=1 SV=2 | 47,3 | 9,36 | 260 | 69 | 2 | 2 | 2 | 2 | 2 | 4 |
| Q9UKN8 | General transcription factor 3C polypeptide 4 OS=Homo sapiens OX=9606 GN=GTF3C4 PE=1 SV=2 | 91,9 | 6,65 | 875 | 69 | 2 | 2 | 2 | 2 | 2 | 2 |
| Q9UNL2 | Translocon-associated protein subunit gamma OS=Homo sapiens OX=9606 GN=SSR3 PE=1 SV=1 | 21,1 | 9,61 | 895 | 69 | 2 | 1 | 2 | 1 | 1 | 8 |
| O60925 | Prefoldin subunit 1 OS=Homo sapiens OX=9606 GN=PFDN1 PE=1 SV=2 | 14,2 | 6,81 | 489 | 68 | 2 | 2 | 2 | 2 | 2 | 17 |
| Q1ED39 | Lysine-rich nucleolar protein 1 OS=Homo sapiens OX=9606 GN=KNOP1 PE=1 SV=1 | 51,6 | 9,86 | 137 | 68 | 1 | 1 | 1 | 1 | 1 | 2 |
| Q6UN15 | Pre-mRNA 3'-end-processing factor FIP1 OS=Homo sapiens OX=9606 GN=FIP1L1 PE=1 SV=1 | 66,5 | 5,59 | 638 | 68 | 2 | 2 | 2 | 2 | 2 | 6 |
| Q8NBS9 | Thioredoxin domain-containing protein 5 OS=Homo sapiens OX=9606 GN=TXNDC5 PE=1 SV=2 | 47,6 | 5,97 | 631 | 68 | 2 | 2 | 2 | 2 | 2 | 5 |
| Q8TCJ2 | Dolichyl-diphosphooligosaccharide--protein glycosyltransferase subunit STT3B OS=Homo sapiens OX=9606 GN=STT3B PE=1 SV=1 | 93,6 | 8,91 | 253 | 68 | 2 | 2 | 2 | 2 | 1 | 2 |
| Q9C0J8 | pre-mRNA 3' end processing protein WDR33 OS=Homo sapiens OX=9606 GN=WDR33 PE=1 SV=2 | 145,8 | 9,17 | 5 | 68 | 2 | 2 | 2 | 2 | 2 | 2 |
| Q8NCA5 | Protein FAM98A OS=Homo sapiens OX=9606 GN=FAM98A PE=1 SV=2 | 55,2 | 8,95 | 1105 | 67 | 3 | 2 | 3 | 2 | 2 | 5 |
| Q96S59 | Ran-binding protein 9 OS=Homo sapiens OX=9606 GN=RANBP9 PE=1 SV=1 | 77,8 | 6,79 | 815 | 67 | 2 | 2 | 2 | 2 | 2 | 3 |
| Q9HCN4 | GPN-loop GTPase 1 OS=Homo sapiens OX=9606 GN=GPN1 PE=1 SV=1 | 41,7 | 4,92 | 556 | 67 | 1 | 1 | 1 | 1 | 1 | 4 |
| O60762 | Dolichol-phosphate mannosyltransferase subunit 1 OS=Homo sapiens OX=9606 GN=DPM1 PE=1 SV=1 | 29,6 | 9,57 | 688 | 66 | 1 | 1 | 1 | 1 | 1 | 4 |
| P56134 | ATP synthase subunit f, mitochondrial OS=Homo sapiens OX=9606 GN=ATP5MF PE=1 SV=3 | 10,9 | 9,67 | 418 | 66 | 1 | 1 | 1 | 1 | 1 | 12 |
| Q15046 | Lysine--tRNA ligase OS=Homo sapiens OX=9606 GN=KARS1 PE=1 SV=3 | 68 | 6,35 | 230 | 66 | 2 | 2 | 2 | 2 | 2 | 4 |
| Q9H9J2 | 39S ribosomal protein L44, mitochondrial OS=Homo sapiens OX=9606 GN=MRPL44 PE=1 SV=1 | 37,5 | 8,4 | 745 | 66 | 2 | 2 | 2 | 2 | 2 | 8 |
| O00487 | 26S proteasome non-ATPase regulatory subunit 14 OS=Homo sapiens OX=9606 GN=PSMD14 PE=1 SV=1 | 34,6 | 6,52 | 822 | 65 | 2 | 2 | 2 | 2 | 2 | 13 |
| O43920 | NADH dehydrogenase [ubiquinone] iron-sulfur protein 5 OS=Homo sapiens OX=9606 GN=NDUFS5 PE=1 SV=3 | 12,5 | 9,14 | 728 | 65 | 2 | 2 | 2 | 2 | 2 | 18 |
| P21912 | Succinate dehydrogenase [ubiquinone] iron-sulfur subunit, mitochondrial OS=Homo sapiens OX=9606 GN=SDHB PE=1 SV=3 | 31,6 | 8,76 | 39 | 65 | 2 | 2 | 2 | 2 | 2 | 9 |
| Q04637 | Eukaryotic translation initiation factor 4 gamma 1 OS=Homo sapiens OX=9606 GN=EIF4G1 PE=1 SV=4 | 175,4 | 5,33 | 226 | 65 | 2 | 2 | 2 | 2 | 2 | 2 |
| Q15645 | Pachytene checkpoint protein 2 homolog OS=Homo sapiens OX=9606 GN=TRIP13 PE=1 SV=2 | 48,5 | 6,09 | 941 | 65 | 2 | 2 | 2 | 2 | 2 | 4 |
| Q15392 | Delta(24)-sterol reductase OS=Homo sapiens OX=9606 GN=DHCR24 PE=1 SV=2 | 60,1 | 8,16 | 786 | 64 | 2 | 2 | 2 | 2 | 2 | 3 |
| Q5JTH9 | RRP12-like protein OS=Homo sapiens OX=9606 GN=RRP12 PE=1 SV=2 | 143,6 | 8,75 | 755 | 64 | 1 | 1 | 1 | 1 | 1 | 1 |
| Q99471 | Prefoldin subunit 5 OS=Homo sapiens OX=9606 GN=PFDN5 PE=1 SV=2 | 17,3 | 6,33 | 350 | 64 | 1 | 1 | 1 | 1 | 1 | 10 |
| Q9H1K4 | Mitochondrial glutamate carrier 2 OS=Homo sapiens OX=9606 GN=SLC25A18 PE=1 SV=1 | 33,8 | 9,25 | 307 | 64 | 2 | 2 | 2 | 2 | 2 | 7 |
| Q9Y4L1 | Hypoxia up-regulated protein 1 OS=Homo sapiens OX=9606 GN=HYOU1 PE=1 SV=1 | 111,3 | 5,22 | 257 | 64 | 2 | 2 | 2 | 2 | 2 | 3 |
| Q15293 | Reticulocalbin-1 OS=Homo sapiens OX=9606 GN=RCN1 PE=1 SV=1 | 38,9 | 5 | 1067 | 63 | 3 | 2 | 3 | 2 | 2 | 6 |
| Q96GC5 | 39S ribosomal protein L48, mitochondrial OS=Homo sapiens OX=9606 GN=MRPL48 PE=1 SV=2 | 23,9 | 8,98 | 255 | 63 | 1 | 1 | 1 | 1 | 1 | 6 |
| Q9BV68 | E3 ubiquitin-protein ligase RNF126 OS=Homo sapiens OX=9606 GN=RNF126 PE=1 SV=2 | 33,8 | 5,47 | 434 | 63 | 2 | 2 | 2 | 2 | 2 | 7 |
| Q9NYK5 | 39S ribosomal protein L39, mitochondrial OS=Homo sapiens OX=9606 GN=MRPL39 PE=1 SV=3 | 38,7 | 7,65 | 695 | 63 | 2 | 2 | 2 | 2 | 2 | 8 |
| Q9P2R7 | Succinate--CoA ligase [ADP-forming] subunit beta, mitochondrial OS=Homo sapiens OX=9606 GN=SUCLA2 PE=1 SV=3 | 50,3 | 7,42 | 924 | 63 | 2 | 2 | 2 | 2 | 2 | 4 |
| O43447 | Peptidyl-prolyl cis-trans isomerase H OS=Homo sapiens OX=9606 GN=PPIH PE=1 SV=1 | 19,2 | 8,07 | 1073 | 62 | 2 | 2 | 2 | 2 | 2 | 13 |
| O75380 | NADH dehydrogenase [ubiquinone] iron-sulfur protein 6, mitochondrial OS=Homo sapiens OX=9606 GN=NDUFS6 PE=1 SV=1 | 13,7 | 8,28 | 400 | 62 | 1 | 1 | 1 | 1 | 1 | 12 |
| P06396 | Gelsolin OS=Homo sapiens OX=9606 GN=GSN PE=1 SV=1 | 85,6 | 6,28 | 524 | 62 | 2 | 2 | 2 | 2 | 2 | 3 |
| P19388 | DNA-directed RNA polymerases I, II, and III subunit RPABC1 OS=Homo sapiens OX=9606 GN=POLR2E PE=1 SV=4 | 24,5 | 5,95 | 813 | 62 | 2 | 2 | 2 | 2 | 2 | 11 |
| Q96AG4 | Leucine-rich repeat-containing protein 59 OS=Homo sapiens OX=9606 GN=LRRC59 PE=1 SV=1 | 34,9 | 9,57 | 347 | 62 | 2 | 2 | 2 | 2 | 2 | 7 |
| Q99436 | Proteasome subunit beta type-7 OS=Homo sapiens OX=9606 GN=PSMB7 PE=1 SV=1 | 29,9 | 7,68 | 211 | 62 | 1 | 1 | 1 | 1 | 1 | 4 |
| Q9Y3C6 | Peptidyl-prolyl cis-trans isomerase-like 1 OS=Homo sapiens OX=9606 GN=PPIL1 PE=1 SV=1 | 18,2 | 7,99 | 855 | 62 | 1 | 1 | 1 | 1 | 1 | 6 |
| P61204 | ADP-ribosylation factor 3 OS=Homo sapiens OX=9606 GN=ARF3 PE=1 SV=2 | 20,6 | 7,43 | 104 | 61 | 1 | 1 | 1 | 1 | 1 | 6 |
| P61326 | Protein mago nashi homolog OS=Homo sapiens OX=9606 GN=MAGOH PE=1 SV=1 | 17,2 | 6,11 | 359 | 61 | 2 | 2 | 2 | 2 | 1 | 12 |
| Q3ZCQ8 | Mitochondrial import inner membrane translocase subunit TIM50 OS=Homo sapiens OX=9606 GN=TIMM50 PE=1 SV=2 | 39,6 | 8,37 | 836 | 61 | 3 | 2 | 3 | 2 | 2 | 7 |
| Q6UWP8 | Suprabasin OS=Homo sapiens OX=9606 GN=SBSN PE=1 SV=2 | 60,5 | 7,01 | 774 | 61 | 1 | 1 | 1 | 1 | 1 | 3 |
| Q70IA6 | MOB kinase activator 2 OS=Homo sapiens OX=9606 GN=MOB2 PE=1 SV=1 | 26,9 | 6,79 | 950 | 61 | 2 | 2 | 2 | 2 | 2 | 6 |
| Q96A72 | Protein mago nashi homolog 2 OS=Homo sapiens OX=9606 GN=MAGOHB PE=1 SV=1 | 17,3 | 6,39 | 782 | 61 | 2 | 2 | 2 | 2 | 1 | 13 |
| Q9BYN8 | 28S ribosomal protein S26, mitochondrial OS=Homo sapiens OX=9606 GN=MRPS26 PE=1 SV=1 | 24,2 | 10,39 | 622 | 61 | 2 | 2 | 2 | 2 | 2 | 10 |
| Q9NQ92 | Coordinator of PRMT5 and differentiation stimulator OS=Homo sapiens OX=9606 GN=COPRS PE=1 SV=3 | 20,1 | 4,18 | 542 | 61 | 2 | 2 | 2 | 2 | 2 | 14 |
| O14893 | Gem-associated protein 2 OS=Homo sapiens OX=9606 GN=GEMIN2 PE=1 SV=1 | 31,6 | 5,58 | 626 | 60 | 2 | 2 | 2 | 2 | 2 | 5 |
| O94776 | Metastasis-associated protein MTA2 OS=Homo sapiens OX=9606 GN=MTA2 PE=1 SV=1 | 75 | 9,66 | 674 | 60 | 2 | 2 | 2 | 2 | 2 | 3 |
| Q07065 | Cytoskeleton-associated protein 4 OS=Homo sapiens OX=9606 GN=CKAP4 PE=1 SV=2 | 66 | 5,92 | 1081 | 60 | 2 | 2 | 2 | 2 | 2 | 6 |
| Q99942 | E3 ubiquitin-protein ligase RNF5 OS=Homo sapiens OX=9606 GN=RNF5 PE=1 SV=1 | 19,9 | 6,65 | 757 | 60 | 1 | 1 | 1 | 1 | 1 | 8 |
| Q9Y3B2 | Exosome complex component CSL4 OS=Homo sapiens OX=9606 GN=EXOSC1 PE=1 SV=1 | 21,4 | 8,24 | 512 | 60 | 2 | 2 | 2 | 2 | 2 | 14 |
| Q9Y3D3 | 28S ribosomal protein S16, mitochondrial OS=Homo sapiens OX=9606 GN=MRPS16 PE=1 SV=1 | 15,3 | 9,5 | 1040 | 60 | 2 | 2 | 2 | 2 | 2 | 19 |
| O76094 | Signal recognition particle subunit SRP72 OS=Homo sapiens OX=9606 GN=SRP72 PE=1 SV=3 | 74,6 | 9,26 | 439 | 59 | 2 | 2 | 2 | 2 | 2 | 4 |
| P10644 | cAMP-dependent protein kinase type I-alpha regulatory subunit OS=Homo sapiens OX=9606 GN=PRKAR1A PE=1 SV=1 | 43 | 5,35 | 19 | 59 | 2 | 2 | 2 | 2 | 2 | 6 |
| Q14978 | Nucleolar and coiled-body phosphoprotein 1 OS=Homo sapiens OX=9606 GN=NOLC1 PE=1 SV=2 | 73,6 | 9,47 | 679 | 59 | 2 | 2 | 2 | 2 | 2 | 3 |
| Q93100 | Phosphorylase b kinase regulatory subunit beta OS=Homo sapiens OX=9606 GN=PHKB PE=1 SV=3 | 124,8 | 6,95 | 265 | 59 | 2 | 2 | 2 | 2 | 2 | 2 |
| Q96S55 | ATPase WRNIP1 OS=Homo sapiens OX=9606 GN=WRNIP1 PE=1 SV=2 | 72,1 | 6,1 | 530 | 59 | 2 | 2 | 2 | 2 | 2 | 3 |
| Q9BU76 | Multiple myeloma tumor-associated protein 2 OS=Homo sapiens OX=9606 GN=MMTAG2 PE=1 SV=1 | 29,4 | 10,02 | 883 | 59 | 2 | 2 | 2 | 2 | 2 | 8 |
| O43684 | Mitotic checkpoint protein BUB3 OS=Homo sapiens OX=9606 GN=BUB3 PE=1 SV=1 | 37,1 | 6,84 | 99 | 58 | 2 | 2 | 2 | 2 | 2 | 8 |
| O95619 | YEATS domain-containing protein 4 OS=Homo sapiens OX=9606 GN=YEATS4 PE=1 SV=1 | 26,5 | 8,41 | 968 | 58 | 2 | 2 | 2 | 2 | 2 | 10 |
| O96008 | Mitochondrial import receptor subunit TOM40 homolog OS=Homo sapiens OX=9606 GN=TOMM40 PE=1 SV=1 | 37,9 | 7,25 | 1114 | 58 | 1 | 1 | 1 | 1 | 1 | 3 |
| P53677 | AP-3 complex subunit mu-2 OS=Homo sapiens OX=9606 GN=AP3M2 PE=1 SV=1 | 46,9 | 7,56 | 497 | 58 | 2 | 2 | 2 | 2 | 2 | 5 |
| P55265 | Double-stranded RNA-specific adenosine deaminase OS=Homo sapiens OX=9606 GN=ADAR PE=1 SV=4 | 136 | 8,65 | 851 | 58 | 2 | 2 | 2 | 2 | 2 | 2 |
| P61758 | Prefoldin subunit 3 OS=Homo sapiens OX=9606 GN=VBP1 PE=1 SV=4 | 22,6 | 7,11 | 20 | 58 | 2 | 2 | 2 | 2 | 2 | 11 |
| Q00059 | Transcription factor A, mitochondrial OS=Homo sapiens OX=9606 GN=TFAM PE=1 SV=1 | 29,1 | 9,72 | 666 | 58 | 1 | 1 | 1 | 1 | 1 | 4 |
| Q01650 | Large neutral amino acids transporter small subunit 1 OS=Homo sapiens OX=9606 GN=SLC7A5 PE=1 SV=2 | 55 | 7,72 | 395 | 58 | 1 | 1 | 1 | 1 | 1 | 3 |
| P33992 | DNA replication licensing factor MCM5 OS=Homo sapiens OX=9606 GN=MCM5 PE=1 SV=5 | 82,2 | 8,37 | 311 | 57 | 2 | 2 | 2 | 2 | 2 | 3 |
| Q8NE71 | ATP-binding cassette sub-family F member 1 OS=Homo sapiens OX=9606 GN=ABCF1 PE=1 SV=2 | 95,9 | 6,8 | 86 | 57 | 2 | 2 | 2 | 2 | 2 | 3 |
| Q96DV4 | 39S ribosomal protein L38, mitochondrial OS=Homo sapiens OX=9606 GN=MRPL38 PE=1 SV=2 | 44,6 | 7,53 | 364 | 57 | 2 | 2 | 2 | 2 | 2 | 5 |
| P15531 | Nucleoside diphosphate kinase A OS=Homo sapiens OX=9606 GN=NME1 PE=1 SV=1 | 17,1 | 6,19 | 46 | 56 | 1 | 1 | 1 | 1 | 1 | 11 |
| P20930 | Filaggrin OS=Homo sapiens OX=9606 GN=FLG PE=1 SV=3 | 434,9 | 9,25 | 775 | 56 | 1 | 1 | 1 | 1 | 1 | 0 |
| P22102 | Trifunctional purine biosynthetic protein adenosine-3 OS=Homo sapiens OX=9606 GN=GART PE=1 SV=1 | 107,7 | 6,7 | 388 | 56 | 2 | 2 | 2 | 2 | 2 | 4 |
| P49406 | 39S ribosomal protein L19, mitochondrial OS=Homo sapiens OX=9606 GN=MRPL19 PE=1 SV=2 | 33,5 | 9,5 | 879 | 56 | 2 | 2 | 2 | 2 | 2 | 6 |
| Q13642 | Four and a half LIM domains protein 1 OS=Homo sapiens OX=9606 GN=FHL1 PE=1 SV=4 | 36,2 | 8,97 | 433 | 56 | 1 | 1 | 1 | 1 | 1 | 4 |
| Q14574 | Desmocollin-3 OS=Homo sapiens OX=9606 GN=DSC3 PE=1 SV=3 | 99,9 | 6,1 | 818 | 56 | 1 | 1 | 1 | 1 | 1 | 1 |
| Q14966 | Zinc finger protein 638 OS=Homo sapiens OX=9606 GN=ZNF638 PE=1 SV=2 | 220,5 | 6,38 | 559 | 56 | 2 | 2 | 2 | 2 | 2 | 1 |
| Q1KMD3 | Heterogeneous nuclear ribonucleoprotein U-like protein 2 OS=Homo sapiens OX=9606 GN=HNRNPUL2 PE=1 SV=1 | 85,1 | 4,91 | 1056 | 56 | 2 | 2 | 2 | 2 | 2 | 3 |
| Q6UB35 | Monofunctional C1-tetrahydrofolate synthase, mitochondrial OS=Homo sapiens OX=9606 GN=MTHFD1L PE=1 SV=1 | 105,7 | 8,06 | 630 | 56 | 2 | 2 | 2 | 2 | 2 | 3 |
| Q96PV7 | Protein FAM193B OS=Homo sapiens OX=9606 GN=FAM193B PE=1 SV=3 | 96,5 | 7,9 | 187 | 56 | 1 | 1 | 1 | 1 | 1 | 1 |
| O14737 | Programmed cell death protein 5 OS=Homo sapiens OX=9606 GN=PDCD5 PE=1 SV=3 | 14,3 | 6,04 | 369 | 55 | 1 | 1 | 1 | 1 | 1 | 10 |
| P02545 | Prelamin-A/C OS=Homo sapiens OX=9606 GN=LMNA PE=1 SV=1 | 74,1 | 7,02 | 595 | 55 | 2 | 2 | 2 | 2 | 1 | 3 |
| P17812 | CTP synthase 1 OS=Homo sapiens OX=9606 GN=CTPS1 PE=1 SV=2 | 66,6 | 6,46 | 1077 | 55 | 1 | 1 | 1 | 1 | 1 | 2 |
| P35637 | RNA-binding protein FUS OS=Homo sapiens OX=9606 GN=FUS PE=1 SV=1 | 53,4 | 9,36 | 582 | 55 | 2 | 2 | 2 | 2 | 2 | 5 |
| Q8N684 | Cleavage and polyadenylation specificity factor subunit 7 OS=Homo sapiens OX=9606 GN=CPSF7 PE=1 SV=1 | 52 | 8 | 247 | 55 | 2 | 2 | 2 | 2 | 2 | 4 |
| Q9NSD9 | Phenylalanine--tRNA ligase beta subunit OS=Homo sapiens OX=9606 GN=FARSB PE=1 SV=3 | 66,1 | 6,84 | 172 | 55 | 1 | 1 | 1 | 1 | 1 | 2 |
| Q9P013 | Spliceosome-associated protein CWC15 homolog OS=Homo sapiens OX=9606 GN=CWC15 PE=1 SV=2 | 26,6 | 5,71 | 600 | 55 | 1 | 1 | 1 | 1 | 1 | 5 |
| Q9UNM6 | 26S proteasome non-ATPase regulatory subunit 13 OS=Homo sapiens OX=9606 GN=PSMD13 PE=1 SV=2 | 42,9 | 5,81 | 796 | 55 | 1 | 1 | 1 | 1 | 1 | 3 |
| Q9Y3A3 | MOB-like protein phocein OS=Homo sapiens OX=9606 GN=MOB4 PE=1 SV=1 | 26 | 5,78 | 169 | 55 | 2 | 2 | 2 | 2 | 2 | 10 |
| P11171 | Protein 4.1 OS=Homo sapiens OX=9606 GN=EPB41 PE=1 SV=4 | 97 | 5,58 | 826 | 54 | 1 | 1 | 1 | 1 | 1 | 2 |
| P49755 | Transmembrane emp24 domain-containing protein 10 OS=Homo sapiens OX=9606 GN=TMED10 PE=1 SV=2 | 25 | 7,44 | 344 | 54 | 1 | 1 | 1 | 1 | 1 | 5 |
| Q13243 | Serine/arginine-rich splicing factor 5 OS=Homo sapiens OX=9606 GN=SRSF5 PE=1 SV=1 | 31,2 | 11,59 | 1117 | 54 | 2 | 2 | 2 | 2 | 1 | 9 |
| Q8WWF6 | DnaJ homolog subfamily B member 3 OS=Homo sapiens OX=9606 GN=DNAJB3 PE=1 SV=1 | 16,5 | 4,94 | 77 | 54 | 1 | 1 | 1 | 1 | 1 | 9 |
| Q99496 | E3 ubiquitin-protein ligase RING2 OS=Homo sapiens OX=9606 GN=RNF2 PE=1 SV=1 | 37,6 | 6,84 | 245 | 54 | 2 | 2 | 2 | 2 | 2 | 9 |
| Q9UBQ5 | Eukaryotic translation initiation factor 3 subunit K OS=Homo sapiens OX=9606 GN=EIF3K PE=1 SV=1 | 25 | 4,93 | 331 | 54 | 2 | 2 | 2 | 2 | 2 | 11 |
| O60832 | H/ACA ribonucleoprotein complex subunit DKC1 OS=Homo sapiens OX=9606 GN=DKC1 PE=1 SV=3 | 57,6 | 9,42 | 481 | 53 | 2 | 2 | 2 | 2 | 2 | 5 |
| P61962 | DDB1- and CUL4-associated factor 7 OS=Homo sapiens OX=9606 GN=DCAF7 PE=1 SV=1 | 38,9 | 5,52 | 225 | 53 | 1 | 1 | 1 | 1 | 1 | 4 |
| Q5JSZ5 | Protein PRRC2B OS=Homo sapiens OX=9606 GN=PRRC2B PE=1 SV=2 | 242,8 | 8,34 | 426 | 53 | 1 | 1 | 1 | 1 | 1 | 1 |
| Q9BVK6 | Transmembrane emp24 domain-containing protein 9 OS=Homo sapiens OX=9606 GN=TMED9 PE=1 SV=2 | 27,3 | 8,02 | 620 | 53 | 2 | 2 | 2 | 2 | 2 | 9 |
| Q9NQ50 | 39S ribosomal protein L40, mitochondrial OS=Homo sapiens OX=9606 GN=MRPL40 PE=1 SV=1 | 24,5 | 9,63 | 853 | 53 | 2 | 2 | 2 | 2 | 2 | 13 |
| P14625 | Endoplasmin OS=Homo sapiens OX=9606 GN=HSP90B1 PE=1 SV=1 | 92,4 | 4,84 | 52 | 52 | 2 | 2 | 2 | 2 | 2 | 3 |
| Q06265 | Exosome complex component RRP45 OS=Homo sapiens OX=9606 GN=EXOSC9 PE=1 SV=3 | 48,9 | 5,29 | 244 | 52 | 1 | 1 | 1 | 1 | 1 | 3 |
| Q9NPE3 | H/ACA ribonucleoprotein complex subunit 3 OS=Homo sapiens OX=9606 GN=NOP10 PE=1 SV=1 | 7,7 | 9,99 | 78 | 52 | 1 | 1 | 1 | 1 | 1 | 13 |
| Q9Y676 | 28S ribosomal protein S18b, mitochondrial OS=Homo sapiens OX=9606 GN=MRPS18B PE=1 SV=1 | 29,4 | 9,38 | 1019 | 52 | 1 | 1 | 1 | 1 | 1 | 4 |
| P35658 | Nuclear pore complex protein Nup214 OS=Homo sapiens OX=9606 GN=NUP214 PE=1 SV=2 | 213,5 | 7,47 | 29 | 51 | 2 | 2 | 2 | 2 | 2 | 1 |
| P51648 | Aldehyde dehydrogenase family 3 member A2 OS=Homo sapiens OX=9606 GN=ALDH3A2 PE=1 SV=1 | 54,8 | 7,88 | 178 | 51 | 2 | 2 | 2 | 2 | 2 | 5 |
| P55060 | Exportin-2 OS=Homo sapiens OX=9606 GN=CSE1L PE=1 SV=3 | 110,3 | 5,77 | 449 | 51 | 2 | 2 | 2 | 2 | 2 | 3 |
| P61160 | Actin-related protein 2 OS=Homo sapiens OX=9606 GN=ACTR2 PE=1 SV=1 | 44,7 | 6,74 | 452 | 51 | 1 | 1 | 1 | 1 | 1 | 4 |
| P62995 | Transformer-2 protein homolog beta OS=Homo sapiens OX=9606 GN=TRA2B PE=1 SV=1 | 33,6 | 11,25 | 1111 | 51 | 1 | 1 | 1 | 1 | 1 | 6 |
| P82673 | 28S ribosomal protein S35, mitochondrial OS=Homo sapiens OX=9606 GN=MRPS35 PE=1 SV=1 | 36,8 | 8,24 | 411 | 51 | 2 | 2 | 2 | 2 | 2 | 7 |
| Q15424 | Scaffold attachment factor B1 OS=Homo sapiens OX=9606 GN=SAFB PE=1 SV=4 | 102,6 | 5,47 | 57 | 51 | 2 | 2 | 2 | 2 | 2 | 3 |
| Q9BYD3 | 39S ribosomal protein L4, mitochondrial OS=Homo sapiens OX=9606 GN=MRPL4 PE=1 SV=1 | 34,9 | 9,72 | 143 | 51 | 1 | 1 | 1 | 1 | 1 | 6 |
| Q13148 | TAR DNA-binding protein 43 OS=Homo sapiens OX=9606 GN=TARDBP PE=1 SV=1 | 44,7 | 6,19 | 1118 | 50 | 2 | 2 | 2 | 2 | 2 | 5 |
| Q96N67 | Dedicator of cytokinesis protein 7 OS=Homo sapiens OX=9606 GN=DOCK7 PE=1 SV=4 | 242,4 | 6,8 | 389 | 50 | 2 | 2 | 2 | 2 | 2 | 1 |
| Q9Y3Z3 | Deoxynucleoside triphosphate triphosphohydrolase SAMHD1 OS=Homo sapiens OX=9606 GN=SAMHD1 PE=1 SV=2 | 72,2 | 7,14 | 119 | 50 | 2 | 2 | 2 | 2 | 2 | 4 |
| P02663 | Alpha-S2-casein (Laboratory-Cont) OS=Bos taurus GN=CSN1S2 PE=1 SV=2 | 26 | 8,43 | 346 | 49 | 2 | 2 | 2 | 2 | 2 | 9 |
| P10599 | Thioredoxin OS=Homo sapiens OX=9606 GN=TXN PE=1 SV=3 | 11,7 | 4,92 | 953 | 49 | 1 | 1 | 1 | 1 | 1 | 12 |
| P28074 | Proteasome subunit beta type-5 OS=Homo sapiens OX=9606 GN=PSMB5 PE=1 SV=3 | 28,5 | 6,92 | 67 | 49 | 1 | 1 | 1 | 1 | 1 | 6 |
| P51570 | Galactokinase OS=Homo sapiens OX=9606 GN=GALK1 PE=1 SV=1 | 42,2 | 6,46 | 794 | 49 | 1 | 1 | 1 | 1 | 1 | 3 |
| Q14444 | Caprin-1 OS=Homo sapiens OX=9606 GN=CAPRIN1 PE=1 SV=2 | 78,3 | 5,25 | 243 | 49 | 1 | 1 | 1 | 1 | 1 | 2 |
| Q9BU61 | NADH dehydrogenase [ubiquinone] 1 alpha subcomplex assembly factor 3 OS=Homo sapiens OX=9606 GN=NDUFAF3 PE=1 SV=1 | 20,3 | 8,22 | 676 | 49 | 1 | 1 | 1 | 1 | 1 | 6 |
| Q9P0M9 | 39S ribosomal protein L27, mitochondrial OS=Homo sapiens OX=9606 GN=MRPL27 PE=1 SV=1 | 16,1 | 10,42 | 378 | 49 | 1 | 1 | 1 | 1 | 1 | 7 |
| P52298 | Nuclear cap-binding protein subunit 2 OS=Homo sapiens OX=9606 GN=NCBP2 PE=1 SV=1 | 18 | 8,21 | 88 | 48 | 1 | 1 | 1 | 1 | 1 | 7 |
| P84098 | 60S ribosomal protein L19 OS=Homo sapiens OX=9606 GN=RPL19 PE=1 SV=1 | 23,5 | 11,47 | 455 | 48 | 2 | 1 | 2 | 1 | 1 | 9 |
| Q13185 | Chromobox protein homolog 3 OS=Homo sapiens OX=9606 GN=CBX3 PE=1 SV=4 | 20,8 | 5,33 | 6 | 48 | 1 | 1 | 1 | 1 | 1 | 7 |
| Q13367 | AP-3 complex subunit beta-2 OS=Homo sapiens OX=9606 GN=AP3B2 PE=1 SV=2 | 119 | 5,59 | 478 | 48 | 1 | 1 | 1 | 1 | 1 | 1 |
| Q8N9N2 | Activating signal cointegrator 1 complex subunit 1 OS=Homo sapiens OX=9606 GN=ASCC1 PE=1 SV=1 | 45,5 | 5,54 | 48 | 48 | 1 | 1 | 1 | 1 | 1 | 2 |
| Q9H2W6 | 39S ribosomal protein L46, mitochondrial OS=Homo sapiens OX=9606 GN=MRPL46 PE=1 SV=1 | 31,7 | 7,05 | 1075 | 48 | 1 | 1 | 1 | 1 | 1 | 5 |
| Q9NR09 | Baculoviral IAP repeat-containing protein 6 OS=Homo sapiens OX=9606 GN=BIRC6 PE=1 SV=3 | 529,9 | 6,05 | 872 | 48 | 1 | 1 | 1 | 1 | 1 | 0 |
| P00338 | L-lactate dehydrogenase A chain OS=Homo sapiens OX=9606 GN=LDHA PE=1 SV=2 | 36,7 | 8,27 | 740 | 47 | 1 | 1 | 1 | 1 | 1 | 2 |
| P43243 | Matrin-3 OS=Homo sapiens OX=9606 GN=MATR3 PE=1 SV=2 | 94,6 | 6,25 | 634 | 47 | 1 | 1 | 1 | 1 | 1 | 1 |
| P62714 | Serine/threonine-protein phosphatase 2A catalytic subunit beta isoform OS=Homo sapiens OX=9606 GN=PPP2CB PE=1 SV=1 | 35,6 | 5,43 | 140 | 47 | 1 | 1 | 1 | 1 | 1 | 5 |
| Q01844 | RNA-binding protein EWS OS=Homo sapiens OX=9606 GN=EWSR1 PE=1 SV=1 | 68,4 | 9,33 | 70 | 47 | 1 | 1 | 1 | 1 | 1 | 4 |
| Q14318 | Peptidyl-prolyl cis-trans isomerase FKBP8 OS=Homo sapiens OX=9606 GN=FKBP8 PE=1 SV=2 | 44,5 | 4,84 | 301 | 47 | 1 | 1 | 1 | 1 | 1 | 3 |
| Q8NB16 | Mixed lineage kinase domain-like protein OS=Homo sapiens OX=9606 GN=MLKL PE=1 SV=1 | 54,4 | 8,82 | 568 | 47 | 1 | 1 | 1 | 1 | 1 | 1 |
| Q9BVG3 | E3 ubiquitin-protein ligase TRIM62 OS=Homo sapiens OX=9606 GN=TRIM62 PE=1 SV=1 | 54,2 | 6,62 | 807 | 47 | 1 | 1 | 1 | 1 | 1 | 1 |
| P00352 | Aldehyde dehydrogenase 1A1 OS=Homo sapiens OX=9606 GN=ALDH1A1 PE=1 SV=2 | 54,8 | 6,73 | 621 | 46 | 1 | 1 | 1 | 1 | 1 | 3 |
| P00492 | Hypoxanthine-guanine phosphoribosyltransferase OS=Homo sapiens OX=9606 GN=HPRT1 PE=1 SV=2 | 24,6 | 6,68 | 562 | 46 | 1 | 1 | 1 | 1 | 1 | 5 |
| P01859 | Immunoglobulin heavy constant gamma 2 OS=Homo sapiens OX=9606 GN=IGHG2 PE=1 SV=3 | 43,8 | 6,52 | 15 | 46 | 1 | 1 | 1 | 1 | 1 | 2 |
| P08621 | U1 small nuclear ribonucleoprotein 70 kDa OS=Homo sapiens OX=9606 GN=SNRNP70 PE=1 SV=2 | 51,5 | 9,94 | 45 | 46 | 1 | 1 | 1 | 1 | 1 | 4 |
| P31025 | Lipocalin-1 OS=Homo sapiens OX=9606 GN=LCN1 PE=1 SV=1 | 19,2 | 5,58 | 100 | 46 | 1 | 1 | 1 | 1 | 1 | 6 |
| P43490 | Nicotinamide phosphoribosyltransferase OS=Homo sapiens OX=9606 GN=NAMPT PE=1 SV=1 | 55,5 | 7,15 | 857 | 46 | 1 | 1 | 1 | 1 | 1 | 2 |
| Q12904 | Aminoacyl tRNA synthase complex-interacting multifunctional protein 1 OS=Homo sapiens OX=9606 GN=AIMP1 PE=1 SV=2 | 34,3 | 8,43 | 348 | 46 | 1 | 1 | 1 | 1 | 1 | 4 |
| Q75N03 | E3 ubiquitin-protein ligase Hakai OS=Homo sapiens OX=9606 GN=CBLL1 PE=1 SV=1 | 54,5 | 8,29 | 772 | 46 | 1 | 1 | 1 | 1 | 1 | 2 |
| Q969Q0 | 60S ribosomal protein L36a-like OS=Homo sapiens OX=9606 GN=RPL36AL PE=1 SV=3 | 12,5 | 10,65 | 920 | 46 | 1 | 1 | 1 | 1 | 1 | 8 |
| Q9BY89 | Uncharacterized protein KIAA1671 OS=Homo sapiens OX=9606 GN=KIAA1671 PE=1 SV=2 | 196,6 | 8,47 | 936 | 46 | 1 | 1 | 1 | 1 | 1 | 1 |
| Q9Y4W6 | AFG3-like protein 2 OS=Homo sapiens OX=9606 GN=AFG3L2 PE=1 SV=2 | 88,5 | 8,66 | 336 | 46 | 1 | 1 | 1 | 1 | 1 | 1 |
| Q9Y6G9 | Cytoplasmic dynein 1 light intermediate chain 1 OS=Homo sapiens OX=9606 GN=DYNC1LI1 PE=1 SV=3 | 56,5 | 6,42 | 1049 | 46 | 1 | 1 | 1 | 1 | 1 | 2 |
| P52434 | DNA-directed RNA polymerases I, II, and III subunit RPABC3 OS=Homo sapiens OX=9606 GN=POLR2H PE=1 SV=4 | 17,1 | 4,68 | 1061 | 45 | 1 | 1 | 1 | 1 | 1 | 4 |
| Q8WXF0 | Serine/arginine-rich splicing factor 12 OS=Homo sapiens OX=9606 GN=SRSF12 PE=2 SV=1 | 30,5 | 11,69 | 128 | 45 | 1 | 1 | 1 | 1 | 1 | 4 |
| Q92973 | Transportin-1 OS=Homo sapiens OX=9606 GN=TNPO1 PE=1 SV=2 | 102,3 | 4,98 | 462 | 45 | 2 | 2 | 2 | 2 | 2 | 3 |
| Q9BZX2 | Uridine-cytidine kinase 2 OS=Homo sapiens OX=9606 GN=UCK2 PE=1 SV=1 | 29,3 | 6,7 | 492 | 45 | 1 | 1 | 1 | 1 | 1 | 4 |
| Q9H3G5 | Probable serine carboxypeptidase CPVL OS=Homo sapiens OX=9606 GN=CPVL PE=1 SV=2 | 54,1 | 5,62 | 467 | 45 | 1 | 1 | 1 | 1 | 1 | 2 |
| Q9ULX3 | RNA-binding protein NOB1 OS=Homo sapiens OX=9606 GN=NOB1 PE=1 SV=1 | 46,6 | 7,18 | 858 | 45 | 1 | 1 | 1 | 1 | 1 | 2 |
| Q8NAV1 | Pre-mRNA-splicing factor 38A OS=Homo sapiens OX=9606 GN=PRPF38A PE=1 SV=1 | 37,5 | 9,96 | 547 | 44 | 1 | 1 | 1 | 1 | 1 | 3 |
| P01008 | Antithrombin-III OS=Homo sapiens OX=9606 GN=SERPINC1 PE=1 SV=1 | 52,6 | 6,71 | 473 | 43 | 1 | 1 | 1 | 1 | 1 | 2 |
| P08243 | Asparagine synthetase [glutamine-hydrolyzing] OS=Homo sapiens OX=9606 GN=ASNS PE=1 SV=4 | 64,3 | 6,86 | 706 | 43 | 1 | 1 | 1 | 1 | 1 | 2 |
| P27482 | Calmodulin-like protein 3 OS=Homo sapiens OX=9606 GN=CALML3 PE=1 SV=2 | 16,9 | 4,42 | 156 | 43 | 1 | 1 | 1 | 1 | 1 | 11 |
| P43307 | Translocon-associated protein subunit alpha OS=Homo sapiens OX=9606 GN=SSR1 PE=1 SV=3 | 32,2 | 4,49 | 578 | 43 | 1 | 1 | 1 | 1 | 1 | 3 |
| Q92769 | Histone deacetylase 2 OS=Homo sapiens OX=9606 GN=HDAC2 PE=1 SV=2 | 55,3 | 5,91 | 848 | 43 | 1 | 1 | 1 | 1 | 1 | 4 |
| Q9Y678 | Coatomer subunit gamma-1 OS=Homo sapiens OX=9606 GN=COPG1 PE=1 SV=1 | 97,7 | 5,47 | 396 | 43 | 1 | 1 | 1 | 1 | 1 | 1 |
| O00233 | 26S proteasome non-ATPase regulatory subunit 9 OS=Homo sapiens OX=9606 GN=PSMD9 PE=1 SV=3 | 24,7 | 6,95 | 270 | 42 | 1 | 1 | 1 | 1 | 1 | 4 |
| O95373 | Importin-7 OS=Homo sapiens OX=9606 GN=IPO7 PE=1 SV=1 | 119,4 | 4,82 | 880 | 42 | 1 | 1 | 1 | 1 | 1 | 1 |
| Q15363 | Transmembrane emp24 domain-containing protein 2 OS=Homo sapiens OX=9606 GN=TMED2 PE=1 SV=1 | 22,7 | 5,17 | 196 | 42 | 1 | 1 | 1 | 1 | 1 | 4 |
| Q5VTU8 | ATP synthase subunit epsilon-like protein, mitochondrial OS=Homo sapiens OX=9606 GN=ATP5F1EP2 PE=1 SV=1 | 5,8 | 10,14 | 686 | 42 | 1 | 1 | 1 | 1 | 1 | 16 |
| Q96A33 | PAT complex subunit CCDC47 OS=Homo sapiens OX=9606 GN=CCDC47 PE=1 SV=1 | 55,8 | 4,87 | 430 | 42 | 1 | 1 | 1 | 1 | 1 | 2 |
| O43823 | A-kinase anchor protein 8 OS=Homo sapiens OX=9606 GN=AKAP8 PE=1 SV=1 | 76,1 | 5,15 | 606 | 41 | 1 | 1 | 1 | 1 | 1 | 1 |
| O95232 | Luc7-like protein 3 OS=Homo sapiens OX=9606 GN=LUC7L3 PE=1 SV=2 | 51,4 | 9,79 | 771 | 41 | 1 | 1 | 1 | 1 | 1 | 2 |
| P12277 | Creatine kinase B-type OS=Homo sapiens OX=9606 GN=CKB PE=1 SV=1 | 42,6 | 5,59 | 682 | 41 | 1 | 1 | 1 | 1 | 1 | 4 |
| P13073 | Cytochrome c oxidase subunit 4 isoform 1, mitochondrial OS=Homo sapiens OX=9606 GN=COX4I1 PE=1 SV=1 | 19,6 | 9,51 | 693 | 41 | 1 | 1 | 1 | 1 | 1 | 6 |
| Q5SRE5 | Nucleoporin NUP188 OS=Homo sapiens OX=9606 GN=NUP188 PE=1 SV=1 | 195,9 | 6,73 | 414 | 41 | 1 | 1 | 1 | 1 | 1 | 1 |
| Q99871 | HAUS augmin-like complex subunit 7 OS=Homo sapiens OX=9606 GN=HAUS7 PE=1 SV=4 | 39,8 | 4,75 | 91 | 41 | 1 | 1 | 1 | 1 | 1 | 4 |
| P08579 | U2 small nuclear ribonucleoprotein B'' OS=Homo sapiens OX=9606 GN=SNRPB2 PE=1 SV=1 | 25,5 | 9,72 | 1095 | 40 | 1 | 1 | 1 | 1 | 1 | 8 |
| P11441 | Ubiquitin-like protein 4A OS=Homo sapiens OX=9606 GN=UBL4A PE=1 SV=1 | 17,8 | 8,66 | 495 | 40 | 1 | 1 | 1 | 1 | 1 | 5 |
| P46459 | Vesicle-fusing ATPase OS=Homo sapiens OX=9606 GN=NSF PE=1 SV=3 | 82,5 | 6,95 | 736 | 40 | 1 | 1 | 1 | 1 | 1 | 1 |
| P48729 | Casein kinase I isoform alpha OS=Homo sapiens OX=9606 GN=CSNK1A1 PE=1 SV=2 | 38,9 | 9,57 | 810 | 40 | 1 | 1 | 1 | 1 | 1 | 3 |
| P49674 | Casein kinase I isoform epsilon OS=Homo sapiens OX=9606 GN=CSNK1E PE=1 SV=1 | 47,3 | 9,66 | 687 | 40 | 1 | 1 | 1 | 1 | 1 | 3 |
| Q9BXW9 | Fanconi anemia group D2 protein OS=Homo sapiens OX=9606 GN=FANCD2 PE=1 SV=2 | 164 | 5,88 | 527 | 40 | 1 | 1 | 1 | 1 | 1 | 1 |
| Q9NZT1 | Calmodulin-like protein 5 OS=Homo sapiens OX=9606 GN=CALML5 PE=1 SV=2 | 15,9 | 4,44 | 709 | 40 | 1 | 1 | 1 | 1 | 1 | 10 |
| Q9Y2Q9 | 28S ribosomal protein S28, mitochondrial OS=Homo sapiens OX=9606 GN=MRPS28 PE=1 SV=1 | 20,8 | 9,1 | 635 | 40 | 1 | 1 | 1 | 1 | 1 | 6 |
| P43487 | Ran-specific GTPase-activating protein OS=Homo sapiens OX=9606 GN=RANBP1 PE=1 SV=1 | 23,3 | 5,29 | 110 | 39 | 1 | 1 | 1 | 1 | 1 | 4 |
| Q16637 | Survival motor neuron protein OS=Homo sapiens OX=9606 GN=SMN1 PE=1 SV=1 | 31,8 | 6,55 | 374 | 39 | 1 | 1 | 1 | 1 | 1 | 6 |
| Q6NUP7 | Serine/threonine-protein phosphatase 4 regulatory subunit 4 OS=Homo sapiens OX=9606 GN=PPP4R4 PE=1 SV=1 | 99,4 | 7,8 | 1035 | 39 | 1 | 1 | 1 | 1 | 1 | 1 |
| Q86XN8 | RNA-binding protein MEX3D OS=Homo sapiens OX=9606 GN=MEX3D PE=1 SV=3 | 64,8 | 5,35 | 16 | 39 | 1 | 1 | 1 | 1 | 1 | 1 |
| Q96J01 | THO complex subunit 3 OS=Homo sapiens OX=9606 GN=THOC3 PE=1 SV=1 | 38,7 | 6,09 | 1036 | 39 | 1 | 1 | 1 | 1 | 1 | 3 |
| Q9H3K6 | BolA-like protein 2 OS=Homo sapiens OX=9606 GN=BOLA2 PE=1 SV=1 | 10,1 | 6,52 | 176 | 39 | 1 | 1 | 1 | 1 | 1 | 19 |
| Q9Y2P8 | RNA 3'-terminal phosphate cyclase-like protein OS=Homo sapiens OX=9606 GN=RCL1 PE=1 SV=3 | 40,8 | 9,26 | 376 | 39 | 1 | 1 | 1 | 1 | 1 | 2 |
| O75828 | Carbonyl reductase [NADPH] 3 OS=Homo sapiens OX=9606 GN=CBR3 PE=1 SV=3 | 30,8 | 6,18 | 610 | 38 | 1 | 1 | 1 | 1 | 1 | 6 |
| O95299 | NADH dehydrogenase [ubiquinone] 1 alpha subcomplex subunit 10, mitochondrial OS=Homo sapiens OX=9606 GN=NDUFA10 PE=1 SV=1 | 40,7 | 8,48 | 450 | 38 | 1 | 1 | 1 | 1 | 1 | 3 |
| P01876 | Immunoglobulin heavy constant alpha 1 OS=Homo sapiens OX=9606 GN=IGHA1 PE=1 SV=3 | 42,8 | 5,64 | 1008 | 38 | 1 | 1 | 1 | 1 | 1 | 4 |
| P30566 | Adenylosuccinate lyase OS=Homo sapiens OX=9606 GN=ADSL PE=1 SV=2 | 54,9 | 7,11 | 933 | 38 | 1 | 1 | 1 | 1 | 1 | 3 |
| P49821 | NADH dehydrogenase [ubiquinone] flavoprotein 1, mitochondrial OS=Homo sapiens OX=9606 GN=NDUFV1 PE=1 SV=4 | 50,8 | 8,21 | 387 | 38 | 1 | 1 | 1 | 1 | 1 | 2 |
| P62826 | GTP-binding nuclear protein Ran OS=Homo sapiens OX=9606 GN=RAN PE=1 SV=3 | 24,4 | 7,49 | 980 | 38 | 1 | 1 | 1 | 1 | 1 | 5 |
| Q12789 | General transcription factor 3C polypeptide 1 OS=Homo sapiens OX=9606 GN=GTF3C1 PE=1 SV=4 | 238,7 | 7,3 | 650 | 38 | 1 | 1 | 1 | 1 | 1 | 1 |
| Q86VM9 | Zinc finger CCCH domain-containing protein 18 OS=Homo sapiens OX=9606 GN=ZC3H18 PE=1 SV=2 | 106,3 | 8,32 | 146 | 38 | 1 | 1 | 1 | 1 | 1 | 1 |
| Q8NI60 | Atypical kinase COQ8A, mitochondrial OS=Homo sapiens OX=9606 GN=COQ8A PE=1 SV=1 | 71,9 | 6,99 | 200 | 38 | 1 | 1 | 1 | 1 | 1 | 2 |
| Q8TDN6 | Ribosome biogenesis protein BRX1 homolog OS=Homo sapiens OX=9606 GN=BRIX1 PE=1 SV=2 | 41,4 | 9,92 | 377 | 38 | 1 | 1 | 1 | 1 | 1 | 2 |
| Q9H6T3 | RNA polymerase II-associated protein 3 OS=Homo sapiens OX=9606 GN=RPAP3 PE=1 SV=2 | 75,7 | 6,84 | 912 | 38 | 1 | 1 | 1 | 1 | 1 | 3 |
| Q9ULR0 | Pre-mRNA-splicing factor ISY1 homolog OS=Homo sapiens OX=9606 GN=ISY1 PE=1 SV=3 | 33 | 5,17 | 518 | 38 | 1 | 1 | 1 | 1 | 1 | 5 |
| Q9Y2J2 | Band 4.1-like protein 3 OS=Homo sapiens OX=9606 GN=EPB41L3 PE=1 SV=2 | 120,6 | 5,19 | 1043 | 38 | 1 | 1 | 1 | 1 | 1 | 1 |
| O15355 | Protein phosphatase 1G OS=Homo sapiens OX=9606 GN=PPM1G PE=1 SV=1 | 59,2 | 4,36 | 567 | 37 | 1 | 1 | 1 | 1 | 1 | 2 |
| O96000 | NADH dehydrogenase [ubiquinone] 1 beta subcomplex subunit 10 OS=Homo sapiens OX=9606 GN=NDUFB10 PE=1 SV=3 | 20,8 | 8,48 | 909 | 37 | 1 | 1 | 1 | 1 | 1 | 6 |
| Q15386 | Ubiquitin-protein ligase E3C OS=Homo sapiens OX=9606 GN=UBE3C PE=1 SV=3 | 123,8 | 6,71 | 302 | 37 | 1 | 1 | 1 | 1 | 1 | 1 |
| Q53H12 | Acylglycerol kinase, mitochondrial OS=Homo sapiens OX=9606 GN=AGK PE=1 SV=2 | 47,1 | 8,09 | 960 | 37 | 1 | 1 | 1 | 1 | 1 | 2 |
| Q9NSI2 | Ribosome biogenesis protein SLX9 homolog OS=Homo sapiens OX=9606 GN=SLX9 PE=1 SV=2 | 25,4 | 11,08 | 1100 | 37 | 1 | 1 | 1 | 1 | 1 | 5 |
| Q9Y2L1 | Exosome complex exonuclease RRP44 OS=Homo sapiens OX=9606 GN=DIS3 PE=1 SV=2 | 108,9 | 7,14 | 157 | 37 | 1 | 1 | 1 | 1 | 1 | 2 |
| Q9Y5B9 | FACT complex subunit SPT16 OS=Homo sapiens OX=9606 GN=SUPT16H PE=1 SV=1 | 119,8 | 5,66 | 1025 | 37 | 1 | 1 | 1 | 1 | 1 | 1 |
| E9PRG8 | Uncharacterized protein C11orf98 OS=Homo sapiens OX=9606 GN=C11orf98 PE=4 SV=2 | 14,2 | 11,53 | 223 | 36 | 1 | 1 | 1 | 1 | 1 | 11 |
| O43251 | RNA binding protein fox-1 homolog 2 OS=Homo sapiens OX=9606 GN=RBFOX2 PE=1 SV=3 | 41,3 | 7,27 | 227 | 36 | 1 | 1 | 1 | 1 | 1 | 4 |
| O60684 | Importin subunit alpha-7 OS=Homo sapiens OX=9606 GN=KPNA6 PE=1 SV=1 | 60 | 4,98 | 812 | 36 | 1 | 1 | 1 | 1 | 1 | 1 |
| P0C7P4 | Putative cytochrome b-c1 complex subunit Rieske-like protein 1 OS=Homo sapiens OX=9606 GN=UQCRFS1P1 PE=5 SV=1 | 30,8 | 8,87 | 365 | 36 | 1 | 1 | 1 | 1 | 1 | 3 |
| P13473 | Lysosome-associated membrane glycoprotein 2 OS=Homo sapiens OX=9606 GN=LAMP2 PE=1 SV=2 | 44,9 | 5,63 | 23 | 36 | 1 | 1 | 1 | 1 | 1 | 2 |
| P21964 | Catechol O-methyltransferase OS=Homo sapiens OX=9606 GN=COMT PE=1 SV=2 | 30 | 5,47 | 117 | 36 | 1 | 1 | 1 | 1 | 1 | 3 |
| P49321 | Nuclear autoantigenic sperm protein OS=Homo sapiens OX=9606 GN=NASP PE=1 SV=2 | 85,2 | 4,3 | 405 | 36 | 1 | 1 | 1 | 1 | 1 | 1 |
| P62942 | Peptidyl-prolyl cis-trans isomerase FKBP1A OS=Homo sapiens OX=9606 GN=FKBP1A PE=1 SV=2 | 11,9 | 8,16 | 1016 | 36 | 1 | 1 | 1 | 1 | 1 | 12 |
| Q13451 | Peptidyl-prolyl cis-trans isomerase FKBP5 OS=Homo sapiens OX=9606 GN=FKBP5 PE=1 SV=2 | 51,2 | 5,9 | 161 | 36 | 1 | 1 | 1 | 1 | 1 | 2 |
| Q15370 | Elongin-B OS=Homo sapiens OX=9606 GN=ELOB PE=1 SV=1 | 13,1 | 4,88 | 312 | 36 | 1 | 1 | 1 | 1 | 1 | 6 |
| Q6FI81 | Anamorsin OS=Homo sapiens OX=9606 GN=CIAPIN1 PE=1 SV=2 | 33,6 | 5,62 | 985 | 36 | 1 | 1 | 1 | 1 | 1 | 4 |
| Q92667 | A-kinase anchor protein 1, mitochondrial OS=Homo sapiens OX=9606 GN=AKAP1 PE=1 SV=1 | 97,3 | 4,94 | 792 | 36 | 1 | 1 | 1 | 1 | 1 | 2 |
| Q9BQ48 | 39S ribosomal protein L34, mitochondrial OS=Homo sapiens OX=9606 GN=MRPL34 PE=1 SV=1 | 10,2 | 12,25 | 162 | 36 | 1 | 1 | 1 | 1 | 1 | 13 |
| Q9UBK9 | Protein UXT OS=Homo sapiens OX=9606 GN=UXT PE=1 SV=1 | 18,2 | 7,59 | 1124 | 36 | 1 | 1 | 1 | 1 | 1 | 7 |
| Q9UKF6 | Cleavage and polyadenylation specificity factor subunit 3 OS=Homo sapiens OX=9606 GN=CPSF3 PE=1 SV=1 | 77,4 | 5,6 | 739 | 36 | 1 | 1 | 1 | 1 | 1 | 2 |
| O43379 | WD repeat-containing protein 62 OS=Homo sapiens OX=9606 GN=WDR62 PE=1 SV=4 | 165,8 | 5,91 | 744 | 35 | 1 | 1 | 1 | 1 | 1 | 1 |
| O43852 | Calumenin OS=Homo sapiens OX=9606 GN=CALU PE=1 SV=2 | 37,1 | 4,64 | 957 | 35 | 1 | 1 | 1 | 1 | 1 | 4 |
| P26583 | High mobility group protein B2 OS=Homo sapiens OX=9606 GN=HMGB2 PE=1 SV=2 | 24 | 7,81 | 118 | 35 | 1 | 1 | 1 | 1 | 1 | 4 |
| P35080 | Profilin-2 OS=Homo sapiens OX=9606 GN=PFN2 PE=1 SV=3 | 15 | 6,99 | 629 | 35 | 1 | 1 | 1 | 1 | 1 | 10 |
| Q14244 | Ensconsin OS=Homo sapiens OX=9606 GN=MAP7 PE=1 SV=1 | 84 | 9,61 | 113 | 35 | 1 | 1 | 1 | 1 | 1 | 1 |
| Q8N766 | ER membrane protein complex subunit 1 OS=Homo sapiens OX=9606 GN=EMC1 PE=1 SV=1 | 111,7 | 7,66 | 749 | 35 | 1 | 1 | 1 | 1 | 1 | 1 |
| Q8NFQ8 | Torsin-1A-interacting protein 2 OS=Homo sapiens OX=9606 GN=TOR1AIP2 PE=1 SV=1 | 51,2 | 4,96 | 607 | 35 | 1 | 1 | 1 | 1 | 1 | 2 |
| Q9BZE1 | 39S ribosomal protein L37, mitochondrial OS=Homo sapiens OX=9606 GN=MRPL37 PE=1 SV=2 | 48,1 | 8,59 | 1120 | 35 | 1 | 1 | 1 | 1 | 1 | 4 |
| Q9BZJ0 | Crooked neck-like protein 1 OS=Homo sapiens OX=9606 GN=CRNKL1 PE=1 SV=4 | 100,4 | 8 | 341 | 35 | 1 | 1 | 1 | 1 | 1 | 1 |
| O14773 | Tripeptidyl-peptidase 1 OS=Homo sapiens OX=9606 GN=TPP1 PE=1 SV=2 | 61,2 | 6,48 | 375 | 34 | 1 | 1 | 1 | 1 | 1 | 2 |
| O43837 | Isocitrate dehydrogenase [NAD] subunit beta, mitochondrial OS=Homo sapiens OX=9606 GN=IDH3B PE=1 SV=2 | 42,2 | 8,46 | 493 | 34 | 1 | 1 | 1 | 1 | 1 | 2 |
| O95433 | Activator of 90 kDa heat shock protein ATPase homolog 1 OS=Homo sapiens OX=9606 GN=AHSA1 PE=1 SV=1 | 38,3 | 5,53 | 711 | 34 | 1 | 1 | 1 | 1 | 1 | 4 |
| P07741 | Adenine phosphoribosyltransferase OS=Homo sapiens OX=9606 GN=APRT PE=1 SV=2 | 19,6 | 6,02 | 700 | 34 | 1 | 1 | 1 | 1 | 1 | 6 |
| P30044 | Peroxiredoxin-5, mitochondrial OS=Homo sapiens OX=9606 GN=PRDX5 PE=1 SV=4 | 22,1 | 8,7 | 423 | 34 | 1 | 1 | 1 | 1 | 1 | 4 |
| Q08AM6 | Protein VAC14 homolog OS=Homo sapiens OX=9606 GN=VAC14 PE=1 SV=1 | 87,9 | 6,13 | 13 | 34 | 1 | 1 | 1 | 1 | 1 | 2 |
| Q13614 | Myotubularin-related protein 2 OS=Homo sapiens OX=9606 GN=MTMR2 PE=1 SV=4 | 73,3 | 7,4 | 868 | 34 | 1 | 1 | 1 | 1 | 1 | 1 |
| Q13685 | Angio-associated migratory cell protein OS=Homo sapiens OX=9606 GN=AAMP PE=1 SV=2 | 46,7 | 4,42 | 580 | 34 | 1 | 1 | 1 | 1 | 1 | 3 |
| Q2NL82 | Pre-rRNA-processing protein TSR1 homolog OS=Homo sapiens OX=9606 GN=TSR1 PE=1 SV=1 | 91,8 | 7,42 | 192 | 34 | 1 | 1 | 1 | 1 | 1 | 1 |
| Q8TB72 | Pumilio homolog 2 OS=Homo sapiens OX=9606 GN=PUM2 PE=1 SV=2 | 114,1 | 7,08 | 352 | 34 | 1 | 1 | 1 | 1 | 1 | 1 |
| Q8WVV4 | Protein POF1B OS=Homo sapiens OX=9606 GN=POF1B PE=1 SV=3 | 68 | 6,32 | 990 | 34 | 1 | 1 | 1 | 1 | 1 | 2 |
| Q9NWT1 | p21-activated protein kinase-interacting protein 1 OS=Homo sapiens OX=9606 GN=PAK1IP1 PE=1 SV=2 | 43,9 | 8,91 | 923 | 34 | 1 | 1 | 1 | 1 | 1 | 3 |
| Q9UGM3 | Deleted in malignant brain tumors 1 protein OS=Homo sapiens OX=9606 GN=DMBT1 PE=1 SV=2 | 260,6 | 5,44 | 235 | 34 | 1 | 1 | 1 | 1 | 1 | 7 |
| O00148 | ATP-dependent RNA helicase DDX39A OS=Homo sapiens OX=9606 GN=DDX39A PE=1 SV=2 | 49,1 | 5,68 | 391 | 33 | 1 | 1 | 1 | 1 | 1 | 3 |
| O00410 | Importin-5 OS=Homo sapiens OX=9606 GN=IPO5 PE=1 SV=4 | 123,6 | 4,94 | 1128 | 33 | 1 | 1 | 1 | 1 | 1 | 1 |
| O60341 | Lysine-specific histone demethylase 1A OS=Homo sapiens OX=9606 GN=KDM1A PE=1 SV=2 | 92,8 | 6,52 | 732 | 33 | 1 | 1 | 1 | 1 | 1 | 1 |
| P00390 | Glutathione reductase, mitochondrial OS=Homo sapiens OX=9606 GN=GSR PE=1 SV=2 | 56,2 | 8,5 | 115 | 33 | 1 | 1 | 1 | 1 | 1 | 2 |
| P19404 | NADH dehydrogenase [ubiquinone] flavoprotein 2, mitochondrial OS=Homo sapiens OX=9606 GN=NDUFV2 PE=1 SV=2 | 27,4 | 8,06 | 1084 | 33 | 1 | 1 | 1 | 1 | 1 | 4 |
| P49006 | MARCKS-related protein OS=Homo sapiens OX=9606 GN=MARCKSL1 PE=1 SV=2 | 19,5 | 4,67 | 476 | 33 | 1 | 1 | 1 | 1 | 1 | 4 |
| P49720 | Proteasome subunit beta type-3 OS=Homo sapiens OX=9606 GN=PSMB3 PE=1 SV=2 | 22,9 | 6,55 | 155 | 33 | 1 | 1 | 1 | 1 | 1 | 4 |
| P53985 | Monocarboxylate transporter 1 OS=Homo sapiens OX=9606 GN=SLC16A1 PE=1 SV=3 | 53,9 | 8,66 | 1050 | 33 | 1 | 1 | 1 | 1 | 1 | 4 |
| P82664 | 28S ribosomal protein S10, mitochondrial OS=Homo sapiens OX=9606 GN=MRPS10 PE=1 SV=2 | 23 | 8 | 802 | 33 | 1 | 1 | 1 | 1 | 1 | 9 |
| Q13155 | Aminoacyl tRNA synthase complex-interacting multifunctional protein 2 OS=Homo sapiens OX=9606 GN=AIMP2 PE=1 SV=2 | 35,3 | 8,22 | 669 | 33 | 1 | 1 | 1 | 1 | 1 | 8 |
| Q15019 | Septin-2 OS=Homo sapiens OX=9606 GN=SEPTIN2 PE=1 SV=1 | 41,5 | 6,6 | 663 | 33 | 1 | 1 | 1 | 1 | 1 | 2 |
| Q16610 | Extracellular matrix protein 1 OS=Homo sapiens OX=9606 GN=ECM1 PE=1 SV=2 | 60,6 | 6,71 | 1106 | 33 | 1 | 1 | 1 | 1 | 1 | 2 |
| Q7Z7F7 | 39S ribosomal protein L55, mitochondrial OS=Homo sapiens OX=9606 GN=MRPL55 PE=1 SV=1 | 15,1 | 11,15 | 263 | 33 | 1 | 1 | 1 | 1 | 1 | 12 |
| Q8WU68 | Splicing factor U2AF 26 kDa subunit OS=Homo sapiens OX=9606 GN=U2AF1L4 PE=1 SV=2 | 25,7 | 7,15 | 616 | 33 | 1 | 1 | 1 | 1 | 1 | 5 |
| Q92600 | CCR4-NOT transcription complex subunit 9 OS=Homo sapiens OX=9606 GN=CNOT9 PE=1 SV=1 | 33,6 | 8,03 | 829 | 33 | 1 | 1 | 1 | 1 | 1 | 4 |
| Q969M3 | Protein YIPF5 OS=Homo sapiens OX=9606 GN=YIPF5 PE=1 SV=1 | 28 | 4,36 | 355 | 33 | 1 | 1 | 1 | 1 | 1 | 5 |
| Q9Y6C9 | Mitochondrial carrier homolog 2 OS=Homo sapiens OX=9606 GN=MTCH2 PE=1 SV=1 | 33,3 | 7,97 | 805 | 33 | 1 | 1 | 1 | 1 | 1 | 3 |
| A5YKK6 | CCR4-NOT transcription complex subunit 1 OS=Homo sapiens OX=9606 GN=CNOT1 PE=1 SV=2 | 266,8 | 7,11 | 362 | 32 | 1 | 1 | 1 | 1 | 1 | 0 |
| O43172 | U4/U6 small nuclear ribonucleoprotein Prp4 OS=Homo sapiens OX=9606 GN=PRPF4 PE=1 SV=2 | 58,4 | 7,42 | 535 | 32 | 1 | 1 | 1 | 1 | 1 | 3 |
| P23284 | Peptidyl-prolyl cis-trans isomerase B OS=Homo sapiens OX=9606 GN=PPIB PE=1 SV=2 | 23,7 | 9,41 | 961 | 32 | 1 | 1 | 1 | 1 | 1 | 6 |
| Q5W0B1 | ORC ubiquitin ligase 1 OS=Homo sapiens OX=9606 GN=OBI1 PE=1 SV=1 | 81,1 | 5,72 | 1113 | 32 | 1 | 1 | 1 | 1 | 1 | 2 |
| Q6P5R6 | 60S ribosomal protein L22-like 1 OS=Homo sapiens OX=9606 GN=RPL22L1 PE=1 SV=2 | 14,6 | 9,38 | 860 | 32 | 1 | 1 | 1 | 1 | 1 | 10 |
| Q7Z739 | YTH domain-containing family protein 3 OS=Homo sapiens OX=9606 GN=YTHDF3 PE=1 SV=1 | 63,8 | 9,04 | 525 | 32 | 1 | 1 | 1 | 1 | 1 | 2 |
| Q8TF09 | Dynein light chain roadblock-type 2 OS=Homo sapiens OX=9606 GN=DYNLRB2 PE=1 SV=1 | 10,8 | 7,5 | 548 | 32 | 1 | 1 | 1 | 1 | 1 | 13 |
| Q96GA3 | Protein LTV1 homolog OS=Homo sapiens OX=9606 GN=LTV1 PE=1 SV=1 | 54,8 | 4,91 | 84 | 32 | 1 | 1 | 1 | 1 | 1 | 2 |
| Q9GZT3 | SRA stem-loop-interacting RNA-binding protein, mitochondrial OS=Homo sapiens OX=9606 GN=SLIRP PE=1 SV=1 | 12,3 | 10,24 | 905 | 32 | 1 | 1 | 1 | 1 | 1 | 9 |
| Q9H0U3 | Magnesium transporter protein 1 OS=Homo sapiens OX=9606 GN=MAGT1 PE=1 SV=1 | 38 | 9,63 | 101 | 32 | 1 | 1 | 1 | 1 | 1 | 3 |
| Q9NP92 | 39S ribosomal protein S30, mitochondrial OS=Homo sapiens OX=9606 GN=MRPS30 PE=1 SV=2 | 50,3 | 7,97 | 738 | 32 | 1 | 1 | 1 | 1 | 1 | 2 |
| Q9NUD5 | Zinc finger CCHC domain-containing protein 3 OS=Homo sapiens OX=9606 GN=ZCCHC3 PE=1 SV=2 | 43,5 | 8,53 | 660 | 32 | 1 | 1 | 1 | 1 | 1 | 3 |
| Q9UI42 | Carboxypeptidase A4 OS=Homo sapiens OX=9606 GN=CPA4 PE=1 SV=2 | 47,3 | 6,7 | 28 | 32 | 1 | 1 | 1 | 1 | 1 | 2 |
| Q9Y421 | Protein FAM32A OS=Homo sapiens OX=9606 GN=FAM32A PE=1 SV=2 | 13,2 | 9,99 | 425 | 32 | 1 | 1 | 1 | 1 | 1 | 8 |
| Q9Y4W2 | Ribosomal biogenesis protein LAS1L OS=Homo sapiens OX=9606 GN=LAS1L PE=1 SV=2 | 83 | 4,73 | 1089 | 32 | 1 | 1 | 1 | 1 | 1 | 2 |
| O60884 | DnaJ homolog subfamily A member 2 OS=Homo sapiens OX=9606 GN=DNAJA2 PE=1 SV=1 | 45,7 | 6,48 | 921 | 31 | 1 | 1 | 1 | 1 | 1 | 2 |
| P18583 | Protein SON OS=Homo sapiens OX=9606 GN=SON PE=1 SV=4 | 263,7 | 5,64 | 30 | 31 | 1 | 1 | 1 | 1 | 1 | 1 |
| P28340 | DNA polymerase delta catalytic subunit OS=Homo sapiens OX=9606 GN=POLD1 PE=1 SV=2 | 123,6 | 7,03 | 1119 | 31 | 1 | 1 | 1 | 1 | 1 | 1 |
| P46060 | Ran GTPase-activating protein 1 OS=Homo sapiens OX=9606 GN=RANGAP1 PE=1 SV=1 | 63,5 | 4,68 | 461 | 31 | 1 | 1 | 1 | 1 | 1 | 2 |
| Q09666 | Neuroblast differentiation-associated protein AHNAK OS=Homo sapiens OX=9606 GN=AHNAK PE=1 SV=2 | 628,7 | 6,15 | 514 | 31 | 1 | 1 | 1 | 1 | 1 | 2 |
| Q15003 | Condensin complex subunit 2 OS=Homo sapiens OX=9606 GN=NCAPH PE=1 SV=3 | 82,5 | 5,06 | 998 | 31 | 1 | 1 | 1 | 1 | 1 | 1 |
| Q8TEX9 | Importin-4 OS=Homo sapiens OX=9606 GN=IPO4 PE=1 SV=2 | 118,6 | 4,96 | 899 | 31 | 1 | 1 | 1 | 1 | 1 | 1 |
| Q9P0J0 | NADH dehydrogenase [ubiquinone] 1 alpha subcomplex subunit 13 OS=Homo sapiens OX=9606 GN=NDUFA13 PE=1 SV=3 | 16,7 | 8,43 | 727 | 31 | 1 | 1 | 1 | 1 | 1 | 6 |
| Q9Y239 | Nucleotide-binding oligomerization domain-containing protein 1 OS=Homo sapiens OX=9606 GN=NOD1 PE=1 SV=1 | 107,6 | 7,11 | 1071 | 31 | 1 | 1 | 1 | 1 | 1 | 1 |
| P62910 | 60S ribosomal protein L32 OS=Homo sapiens OX=9606 GN=RPL32 PE=1 SV=2 | 15,9 | 11,33 | 705 | 31 | 1 | 1 | 1 | 1 | 1 | 10 |
| O60783 | 28S ribosomal protein S14, mitochondrial OS=Homo sapiens OX=9606 GN=MRPS14 PE=1 SV=1 | 15,1 | 11,41 | 491 | 30 | 1 | 1 | 1 | 1 | 1 | 7 |
| O94900 | Thymocyte selection-associated high mobility group box protein TOX OS=Homo sapiens OX=9606 GN=TOX PE=1 SV=3 | 57,5 | 7,33 | 43 | 30 | 1 | 1 | 1 | 1 | 1 | 2 |
| P11166 | Solute carrier family 2, facilitated glucose transporter member 1 OS=Homo sapiens OX=9606 GN=SLC2A1 PE=1 SV=2 | 54 | 8,72 | 894 | 30 | 1 | 1 | 1 | 1 | 1 | 2 |
| P16435 | NADPH--cytochrome P450 reductase OS=Homo sapiens OX=9606 GN=POR PE=1 SV=2 | 76,6 | 5,58 | 337 | 30 | 1 | 1 | 1 | 1 | 1 | 1 |
| P27797 | Calreticulin OS=Homo sapiens OX=9606 GN=CALR PE=1 SV=1 | 48,1 | 4,44 | 1097 | 30 | 1 | 1 | 1 | 1 | 1 | 3 |
| P35237 | Serpin B6 OS=Homo sapiens OX=9606 GN=SERPINB6 PE=1 SV=3 | 42,6 | 5,27 | 466 | 30 | 1 | 1 | 1 | 1 | 1 | 4 |
| P46020 | Phosphorylase b kinase regulatory subunit alpha, skeletal muscle isoform OS=Homo sapiens OX=9606 GN=PHKA1 PE=1 SV=2 | 137,2 | 6,19 | 884 | 30 | 1 | 1 | 1 | 1 | 1 | 1 |
| P61221 | ATP-binding cassette sub-family E member 1 OS=Homo sapiens OX=9606 GN=ABCE1 PE=1 SV=1 | 67,3 | 8,34 | 594 | 30 | 1 | 1 | 1 | 1 | 1 | 3 |
| Q13257 | Mitotic spindle assembly checkpoint protein MAD2A OS=Homo sapiens OX=9606 GN=MAD2L1 PE=1 SV=1 | 23,5 | 5,08 | 74 | 30 | 1 | 1 | 1 | 1 | 1 | 8 |
| Q15006 | ER membrane protein complex subunit 2 OS=Homo sapiens OX=9606 GN=EMC2 PE=1 SV=1 | 34,8 | 6,57 | 967 | 30 | 1 | 1 | 1 | 1 | 1 | 4 |
| Q53GS9 | U4/U6.U5 tri-snRNP-associated protein 2 OS=Homo sapiens OX=9606 GN=USP39 PE=1 SV=2 | 65,3 | 8,91 | 87 | 30 | 1 | 1 | 1 | 1 | 1 | 3 |
| Q6N021 | Methylcytosine dioxygenase TET2 OS=Homo sapiens OX=9606 GN=TET2 PE=1 SV=3 | 223,7 | 7,99 | 1027 | 30 | 1 | 1 | 1 | 1 | 1 | 1 |
| Q8TBB5 | Kelch domain-containing protein 4 OS=Homo sapiens OX=9606 GN=KLHDC4 PE=1 SV=1 | 57,9 | 5,72 | 898 | 30 | 1 | 1 | 1 | 1 | 1 | 2 |
| Q9UPV9 | Trafficking kinesin-binding protein 1 OS=Homo sapiens OX=9606 GN=TRAK1 PE=1 SV=1 | 106 | 5,85 | 126 | 30 | 1 | 1 | 1 | 1 | 1 | 1 |
| O00139 | Kinesin-like protein KIF2A OS=Homo sapiens OX=9606 GN=KIF2A PE=1 SV=3 | 79,9 | 6,68 | 528 | 29 | 1 | 1 | 1 | 1 | 1 | 2 |
| O15397 | Importin-8 OS=Homo sapiens OX=9606 GN=IPO8 PE=1 SV=2 | 119,9 | 5,16 | 721 | 29 | 1 | 1 | 1 | 1 | 1 | 1 |
| O75340 | Programmed cell death protein 6 OS=Homo sapiens OX=9606 GN=PDCD6 PE=1 SV=1 | 21,9 | 5,4 | 917 | 29 | 1 | 1 | 1 | 1 | 1 | 6 |
| O76003 | Glutaredoxin-3 OS=Homo sapiens OX=9606 GN=GLRX3 PE=1 SV=2 | 37,4 | 5,39 | 564 | 29 | 1 | 1 | 1 | 1 | 1 | 4 |
| P06493 | Cyclin-dependent kinase 1 OS=Homo sapiens OX=9606 GN=CDK1 PE=1 SV=3 | 34,1 | 8,4 | 278 | 29 | 1 | 1 | 1 | 1 | 1 | 5 |
| P07384 | Calpain-1 catalytic subunit OS=Homo sapiens OX=9606 GN=CAPN1 PE=1 SV=1 | 81,8 | 5,67 | 1037 | 29 | 1 | 1 | 1 | 1 | 1 | 3 |
| P23490 | Loricrin OS=Homo sapiens OX=9606 GN=LORICRIN PE=1 SV=2 | 25,7 | 8,09 | 216 | 29 | 1 | 1 | 1 | 1 | 1 | 3 |
| P33947 | ER lumen protein-retaining receptor 2 OS=Homo sapiens OX=9606 GN=KDELR2 PE=1 SV=1 | 24,4 | 8,72 | 36 | 29 | 1 | 1 | 1 | 1 | 1 | 5 |
| P60981 | Destrin OS=Homo sapiens OX=9606 GN=DSTN PE=1 SV=3 | 18,5 | 7,85 | 1072 | 29 | 1 | 1 | 1 | 1 | 1 | 7 |
| P61009 | Signal peptidase complex subunit 3 OS=Homo sapiens OX=9606 GN=SPCS3 PE=1 SV=1 | 20,3 | 8,62 | 353 | 29 | 1 | 1 | 1 | 1 | 1 | 7 |
| P84103 | Serine/arginine-rich splicing factor 3 OS=Homo sapiens OX=9606 GN=SRSF3 PE=1 SV=1 | 19,3 | 11,65 | 896 | 29 | 1 | 1 | 1 | 1 | 1 | 5 |
| Q5XKP0 | MICOS complex subunit MIC13 OS=Homo sapiens OX=9606 GN=MICOS13 PE=1 SV=1 | 13,1 | 9,42 | 1011 | 29 | 1 | 1 | 1 | 1 | 1 | 6 |
| Q6F5E8 | Capping protein, Arp2/3 and myosin-I linker protein 2 OS=Homo sapiens OX=9606 GN=CARMIL2 PE=1 SV=2 | 154,6 | 6,76 | 4 | 29 | 1 | 1 | 1 | 1 | 1 | 1 |
| Q8IW35 | Centrosomal protein of 97 kDa OS=Homo sapiens OX=9606 GN=CEP97 PE=1 SV=1 | 96,9 | 5,02 | 254 | 29 | 1 | 1 | 1 | 1 | 1 | 1 |
| Q8WWK9 | Cytoskeleton-associated protein 2 OS=Homo sapiens OX=9606 GN=CKAP2 PE=1 SV=1 | 76,9 | 9,41 | 993 | 29 | 1 | 1 | 1 | 1 | 1 | 2 |
| Q99698 | Lysosomal-trafficking regulator OS=Homo sapiens OX=9606 GN=LYST PE=1 SV=3 | 428,9 | 6,61 | 780 | 29 | 1 | 1 | 1 | 1 | 1 | 0 |
| Q9BZM4 | UL16-binding protein 3 OS=Homo sapiens OX=9606 GN=ULBP3 PE=1 SV=1 | 27,9 | 8,02 | 833 | 29 | 1 | 1 | 1 | 1 | 1 | 5 |
| Q9NXW2 | DnaJ homolog subfamily B member 12 OS=Homo sapiens OX=9606 GN=DNAJB12 PE=1 SV=5 | 41,8 | 8,69 | 183 | 29 | 1 | 1 | 1 | 1 | 1 | 3 |
| Q9Y383 | Putative RNA-binding protein Luc7-like 2 OS=Homo sapiens OX=9606 GN=LUC7L2 PE=1 SV=2 | 46,5 | 10,01 | 219 | 29 | 1 | 1 | 1 | 1 | 1 | 3 |
| O00330 | Pyruvate dehydrogenase protein X component, mitochondrial OS=Homo sapiens OX=9606 GN=PDHX PE=1 SV=3 | 54,1 | 8,66 | 2 | 28 | 1 | 1 | 1 | 1 | 1 | 3 |
| O14929 | Histone acetyltransferase type B catalytic subunit OS=Homo sapiens OX=9606 GN=HAT1 PE=1 SV=2 | 49,5 | 5,69 | 284 | 28 | 1 | 1 | 1 | 1 | 1 | 3 |
| O43395 | U4/U6 small nuclear ribonucleoprotein Prp3 OS=Homo sapiens OX=9606 GN=PRPF3 PE=1 SV=2 | 77,5 | 9,5 | 814 | 28 | 1 | 1 | 1 | 1 | 1 | 1 |
| O43670 | BUB3-interacting and GLEBS motif-containing protein ZNF207 OS=Homo sapiens OX=9606 GN=ZNF207 PE=1 SV=1 | 50,7 | 9,1 | 540 | 28 | 1 | 1 | 1 | 1 | 1 | 3 |
| O75607 | Nucleoplasmin-3 OS=Homo sapiens OX=9606 GN=NPM3 PE=1 SV=3 | 19,3 | 4,63 | 966 | 28 | 1 | 1 | 1 | 1 | 1 | 9 |
| O95573 | Fatty acid CoA ligase Acsl3 OS=Homo sapiens OX=9606 GN=ACSL3 PE=1 SV=3 | 80,4 | 8,38 | 419 | 28 | 1 | 1 | 1 | 1 | 1 | 1 |
| P00747 | Plasminogen OS=Homo sapiens OX=9606 GN=PLG PE=1 SV=2 | 90,5 | 7,24 | 643 | 28 | 1 | 1 | 1 | 1 | 1 | 1 |
| P07195 | L-lactate dehydrogenase B chain OS=Homo sapiens OX=9606 GN=LDHB PE=1 SV=2 | 36,6 | 6,05 | 553 | 28 | 1 | 1 | 1 | 1 | 1 | 4 |
| P14406 | Cytochrome c oxidase subunit 7A2, mitochondrial OS=Homo sapiens OX=9606 GN=COX7A2 PE=1 SV=1 | 9,4 | 9,76 | 83 | 28 | 1 | 1 | 1 | 1 | 1 | 12 |
| P18669 | Phosphoglycerate mutase 1 OS=Homo sapiens OX=9606 GN=PGAM1 PE=1 SV=2 | 28,8 | 7,18 | 102 | 28 | 1 | 1 | 1 | 1 | 1 | 4 |
| P28070 | Proteasome subunit beta type-4 OS=Homo sapiens OX=9606 GN=PSMB4 PE=1 SV=4 | 29,2 | 5,97 | 689 | 28 | 1 | 1 | 1 | 1 | 1 | 3 |
| P49915 | GMP synthase [glutamine-hydrolyzing] OS=Homo sapiens OX=9606 GN=GMPS PE=1 SV=1 | 76,7 | 6,87 | 273 | 28 | 1 | 1 | 1 | 1 | 1 | 1 |
| P50454 | Serpin H1 OS=Homo sapiens OX=9606 GN=SERPINH1 PE=1 SV=2 | 46,4 | 8,69 | 984 | 28 | 1 | 1 | 1 | 1 | 1 | 4 |
| Q86Y56 | Dynein axonemal assembly factor 5 OS=Homo sapiens OX=9606 GN=DNAAF5 PE=1 SV=4 | 93,5 | 6,42 | 849 | 28 | 1 | 1 | 1 | 1 | 1 | 1 |
| Q8IWZ3 | Ankyrin repeat and KH domain-containing protein 1 OS=Homo sapiens OX=9606 GN=ANKHD1 PE=1 SV=1 | 269,3 | 5,73 | 65 | 28 | 1 | 1 | 1 | 1 | 1 | 0 |
| Q9NQ55 | Suppressor of SWI4 1 homolog OS=Homo sapiens OX=9606 GN=PPAN PE=2 SV=1 | 53,2 | 10,13 | 819 | 28 | 1 | 1 | 1 | 1 | 1 | 3 |
| Q9NVS2 | 39S ribosomal protein S18a, mitochondrial OS=Homo sapiens OX=9606 GN=MRPS18A PE=1 SV=1 | 22,2 | 10,33 | 992 | 28 | 1 | 1 | 1 | 1 | 1 | 5 |
| Q9P270 | SLAIN motif-containing protein 2 OS=Homo sapiens OX=9606 GN=SLAIN2 PE=1 SV=2 | 62,5 | 9,45 | 371 | 28 | 1 | 1 | 1 | 1 | 1 | 3 |
| Q9Y4E8 | Ubiquitin carboxyl-terminal hydrolase 15 OS=Homo sapiens OX=9606 GN=USP15 PE=1 SV=3 | 112,3 | 5,22 | 647 | 28 | 1 | 1 | 1 | 1 | 1 | 1 |
| O00743 | Serine/threonine-protein phosphatase 6 catalytic subunit OS=Homo sapiens OX=9606 GN=PPP6C PE=1 SV=1 | 35,1 | 5,69 | 719 | 27 | 1 | 1 | 1 | 1 | 1 | 3 |
| O14545 | TRAF-type zinc finger domain-containing protein 1 OS=Homo sapiens OX=9606 GN=TRAFD1 PE=1 SV=1 | 64,8 | 5,29 | 299 | 27 | 1 | 1 | 1 | 1 | 1 | 2 |
| O75390 | Citrate synthase, mitochondrial OS=Homo sapiens OX=9606 GN=CS PE=1 SV=2 | 51,7 | 8,32 | 228 | 27 | 1 | 1 | 1 | 1 | 1 | 2 |
| P00739 | Haptoglobin-related protein OS=Homo sapiens OX=9606 GN=HPR PE=2 SV=2 | 39 | 7,09 | 259 | 27 | 1 | 1 | 1 | 1 | 1 | 3 |
| P06280 | Alpha-galactosidase A OS=Homo sapiens OX=9606 GN=GLA PE=1 SV=1 | 48,7 | 5,6 | 351 | 27 | 1 | 1 | 1 | 1 | 1 | 2 |
| P10155 | RNA-binding protein RO60 OS=Homo sapiens OX=9606 GN=RO60 PE=1 SV=2 | 60,6 | 8,03 | 149 | 27 | 2 | 1 | 2 | 1 | 1 | 2 |
| P22061 | Protein-L-isoaspartate(D-aspartate) O-methyltransferase OS=Homo sapiens OX=9606 GN=PCMT1 PE=1 SV=4 | 24,6 | 7,21 | 215 | 27 | 1 | 1 | 1 | 1 | 1 | 6 |
| P22735 | Protein-glutamine gamma-glutamyltransferase K OS=Homo sapiens OX=9606 GN=TGM1 PE=1 SV=4 | 89,7 | 6,04 | 98 | 27 | 1 | 1 | 1 | 1 | 1 | 2 |
| P52565 | Rho GDP-dissociation inhibitor 1 OS=Homo sapiens OX=9606 GN=ARHGDIA PE=1 SV=3 | 23,2 | 5,11 | 144 | 27 | 1 | 1 | 1 | 1 | 1 | 7 |
| P62304 | Small nuclear ribonucleoprotein E OS=Homo sapiens OX=9606 GN=SNRPE PE=1 SV=1 | 10,8 | 9,44 | 310 | 27 | 1 | 1 | 1 | 1 | 1 | 13 |
| P83369 | U7 snRNA-associated Sm-like protein LSm11 OS=Homo sapiens OX=9606 GN=LSM11 PE=1 SV=2 | 39,5 | 10,99 | 322 | 27 | 1 | 1 | 1 | 1 | 1 | 3 |
| Q14157 | Ubiquitin-associated protein 2-like OS=Homo sapiens OX=9606 GN=UBAP2L PE=1 SV=2 | 114,5 | 7,11 | 981 | 27 | 1 | 1 | 1 | 1 | 1 | 1 |
| Q14165 | Malectin OS=Homo sapiens OX=9606 GN=MLEC PE=1 SV=1 | 32,2 | 5,41 | 148 | 27 | 1 | 1 | 1 | 1 | 1 | 4 |
| Q14687 | Genetic suppressor element 1 OS=Homo sapiens OX=9606 GN=GSE1 PE=1 SV=3 | 136,1 | 7,74 | 565 | 27 | 1 | 1 | 1 | 1 | 1 | 1 |
| Q15020 | Squamous cell carcinoma antigen recognized by T-cells 3 OS=Homo sapiens OX=9606 GN=SART3 PE=1 SV=1 | 109,9 | 5,57 | 298 | 27 | 1 | 1 | 1 | 1 | 1 | 1 |
| Q3MHD2 | Protein LSM12 OS=Homo sapiens OX=9606 GN=LSM12 PE=1 SV=2 | 21,7 | 7,74 | 549 | 27 | 1 | 1 | 1 | 1 | 1 | 7 |
| Q7Z2T5 | TRMT1-like protein OS=Homo sapiens OX=9606 GN=TRMT1L PE=1 SV=2 | 81,7 | 7,88 | 111 | 27 | 1 | 1 | 1 | 1 | 1 | 1 |
| Q92615 | La-related protein 4B OS=Homo sapiens OX=9606 GN=LARP4B PE=1 SV=3 | 80,5 | 6,92 | 977 | 27 | 1 | 1 | 1 | 1 | 1 | 1 |
| Q96EY7 | Pentatricopeptide repeat domain-containing protein 3, mitochondrial OS=Homo sapiens OX=9606 GN=PTCD3 PE=1 SV=3 | 78,5 | 6,42 | 644 | 27 | 1 | 1 | 1 | 1 | 1 | 1 |
| Q9P015 | 39S ribosomal protein L15, mitochondrial OS=Homo sapiens OX=9606 GN=MRPL15 PE=1 SV=1 | 33,4 | 10,01 | 699 | 27 | 1 | 1 | 1 | 1 | 1 | 3 |
| Q9UJZ1 | Stomatin-like protein 2, mitochondrial OS=Homo sapiens OX=9606 GN=STOML2 PE=1 SV=1 | 38,5 | 7,39 | 1110 | 27 | 1 | 1 | 1 | 1 | 1 | 3 |
| Q9UPQ9 | Trinucleotide repeat-containing gene 6B protein OS=Homo sapiens OX=9606 GN=TNRC6B PE=1 SV=4 | 193,9 | 6,76 | 204 | 27 | 1 | 1 | 1 | 1 | 1 | 1 |
| Q9Y5Q8 | General transcription factor 3C polypeptide 5 OS=Homo sapiens OX=9606 GN=GTF3C5 PE=1 SV=2 | 59,5 | 6,9 | 1125 | 27 | 1 | 1 | 1 | 1 | 1 | 2 |
| O43795 | Unconventional myosin-Ib OS=Homo sapiens OX=9606 GN=MYO1B PE=1 SV=3 | 131,9 | 9,38 | 906 | 26 | 1 | 1 | 1 | 1 | 1 | 1 |
| O60264 | SWI/SNF-related matrix-associated actin-dependent regulator of chromatin subfamily A member 5 OS=Homo sapiens OX=9606 GN=SMARCA5 PE=1 SV=1 | 121,8 | 8,09 | 97 | 26 | 1 | 1 | 1 | 1 | 1 | 1 |
| P06730 | Eukaryotic translation initiation factor 4E OS=Homo sapiens OX=9606 GN=EIF4E PE=1 SV=2 | 25,1 | 6,15 | 685 | 26 | 1 | 1 | 1 | 1 | 1 | 6 |
| P09234 | U1 small nuclear ribonucleoprotein C OS=Homo sapiens OX=9606 GN=SNRPC PE=1 SV=1 | 17,4 | 9,67 | 379 | 26 | 1 | 1 | 1 | 1 | 1 | 8 |
| P10145 | Interleukin-8 OS=Homo sapiens OX=9606 GN=CXCL8 PE=1 SV=1 | 11,1 | 8,84 | 835 | 26 | 1 | 1 | 1 | 1 | 1 | 6 |
| P35659 | Protein DEK OS=Homo sapiens OX=9606 GN=DEK PE=1 SV=1 | 42,6 | 8,56 | 979 | 26 | 1 | 1 | 1 | 1 | 1 | 3 |
| P42704 | Leucine-rich PPR motif-containing protein, mitochondrial OS=Homo sapiens OX=9606 GN=LRPPRC PE=1 SV=3 | 157,8 | 6,13 | 501 | 26 | 1 | 1 | 1 | 1 | 1 | 1 |
| Q13501 | Sequestosome-1 OS=Homo sapiens OX=9606 GN=SQSTM1 PE=1 SV=1 | 47,7 | 5,22 | 703 | 26 | 1 | 1 | 1 | 1 | 1 | 3 |
| Q15070 | Mitochondrial inner membrane protein OXA1L OS=Homo sapiens OX=9606 GN=OXA1L PE=1 SV=3 | 48,5 | 9,45 | 793 | 26 | 1 | 1 | 1 | 1 | 1 | 2 |
| Q16643 | Drebrin OS=Homo sapiens OX=9606 GN=DBN1 PE=1 SV=4 | 71,4 | 4,45 | 614 | 26 | 1 | 1 | 1 | 1 | 1 | 2 |
| Q7Z2W9 | 39S ribosomal protein L21, mitochondrial OS=Homo sapiens OX=9606 GN=MRPL21 PE=1 SV=2 | 22,8 | 9,89 | 22 | 26 | 1 | 1 | 1 | 1 | 1 | 5 |
| Q9UBC9 | Small proline-rich protein 3 OS=Homo sapiens OX=9606 GN=SPRR3 PE=1 SV=2 | 18,1 | 8,57 | 460 | 26 | 1 | 1 | 1 | 1 | 1 | 24 |
| Q9UII4 | E3 ISG15--protein ligase HERC5 OS=Homo sapiens OX=9606 GN=HERC5 PE=1 SV=2 | 116,8 | 7,65 | 295 | 26 | 1 | 1 | 1 | 1 | 1 | 2 |
| O14617 | AP-3 complex subunit delta-1 OS=Homo sapiens OX=9606 GN=AP3D1 PE=1 SV=1 | 130,1 | 8,48 | 220 | 25 | 1 | 1 | 1 | 1 | 1 | 1 |
| O43164 | E3 ubiquitin-protein ligase Praja-2 OS=Homo sapiens OX=9606 GN=PJA2 PE=1 SV=4 | 78,2 | 4,39 | 759 | 25 | 1 | 1 | 1 | 1 | 1 | 3 |
| O94826 | Mitochondrial import receptor subunit TOM70 OS=Homo sapiens OX=9606 GN=TOMM70 PE=1 SV=1 | 67,4 | 7,12 | 651 | 25 | 1 | 1 | 1 | 1 | 1 | 2 |
| P02662 | Alpha-S1-casein (Laboratory-Cont) OS=Bos taurus GN=CSN1S1 PE=1 SV=2 | 24,5 | 5,02 | 94 | 25 | 1 | 1 | 1 | 1 | 1 | 7 |
| P05386 | 60S acidic ribosomal protein P1 OS=Homo sapiens OX=9606 GN=RPLP1 PE=1 SV=1 | 11,5 | 4,32 | 328 | 25 | 1 | 1 | 1 | 1 | 1 | 14 |
| P0DKX4 | Small integral membrane protein 18 OS=Homo sapiens OX=9606 GN=SMIM18 PE=4 SV=1 | 11,1 | 7,17 | 954 | 25 | 1 | 1 | 1 | 1 | 1 | 7 |
| P26639 | Threonine--tRNA ligase 1, cytoplasmic OS=Homo sapiens OX=9606 GN=TARS1 PE=1 SV=3 | 83,4 | 6,67 | 605 | 25 | 1 | 1 | 1 | 1 | 1 | 2 |
| P35241 | Radixin OS=Homo sapiens OX=9606 GN=RDX PE=1 SV=1 | 68,5 | 6,37 | 471 | 25 | 1 | 1 | 1 | 1 | 1 | 2 |
| P51610 | Host cell factor 1 OS=Homo sapiens OX=9606 GN=HCFC1 PE=1 SV=2 | 208,6 | 7,46 | 173 | 25 | 1 | 1 | 1 | 1 | 1 | 1 |
| Q01780 | Exosome component 10 OS=Homo sapiens OX=9606 GN=EXOSC10 PE=1 SV=2 | 100,8 | 8,46 | 1006 | 25 | 1 | 1 | 1 | 1 | 1 | 1 |
| Q13901 | Nuclear nucleic acid-binding protein C1D OS=Homo sapiens OX=9606 GN=C1D PE=1 SV=1 | 16 | 9,03 | 222 | 25 | 1 | 1 | 1 | 1 | 1 | 6 |
| Q14566 | DNA replication licensing factor MCM6 OS=Homo sapiens OX=9606 GN=MCM6 PE=1 SV=1 | 92,8 | 5,41 | 538 | 25 | 1 | 1 | 1 | 1 | 1 | 2 |
| Q5HYJ3 | Protein FAM76B OS=Homo sapiens OX=9606 GN=FAM76B PE=1 SV=3 | 38,7 | 9,25 | 384 | 25 | 1 | 1 | 1 | 1 | 1 | 3 |
| Q9BV38 | WD repeat-containing protein 18 OS=Homo sapiens OX=9606 GN=WDR18 PE=1 SV=2 | 47,4 | 6,7 | 673 | 25 | 1 | 1 | 1 | 1 | 1 | 3 |
| Q9BZE4 | GTP-binding protein 4 OS=Homo sapiens OX=9606 GN=GTPBP4 PE=1 SV=3 | 73,9 | 9,5 | 105 | 25 | 1 | 1 | 1 | 1 | 1 | 2 |
| P00711 | Alpha-lactalbumin (Laboratory-Cont) OS=Bos taurus GN=LALBA PE=1 SV=2 | 16,2 | 5,14 | 249 | 24 | 1 | 1 | 1 | 1 | 1 | 12 |
| P11717 | Cation-independent mannose-6-phosphate receptor OS=Homo sapiens OX=9606 GN=IGF2R PE=1 SV=3 | 274,2 | 5,94 | 597 | 24 | 1 | 1 | 1 | 1 | 1 | 0 |
| P40937 | Replication factor C subunit 5 OS=Homo sapiens OX=9606 GN=RFC5 PE=1 SV=1 | 38,5 | 7,2 | 1004 | 24 | 1 | 1 | 1 | 1 | 1 | 3 |
| P51572 | B-cell receptor-associated protein 31 OS=Homo sapiens OX=9606 GN=BCAP31 PE=1 SV=3 | 28 | 8,44 | 723 | 24 | 1 | 1 | 1 | 1 | 1 | 6 |
| P52701 | DNA mismatch repair protein Msh6 OS=Homo sapiens OX=9606 GN=MSH6 PE=1 SV=2 | 152,7 | 6,9 | 803 | 24 | 1 | 1 | 1 | 1 | 1 | 1 |
| P82914 | 28S ribosomal protein S15, mitochondrial OS=Homo sapiens OX=9606 GN=MRPS15 PE=1 SV=1 | 29,8 | 10,48 | 96 | 24 | 1 | 1 | 1 | 1 | 1 | 4 |
| Q15517 | Corneodesmosin OS=Homo sapiens OX=9606 GN=CDSN PE=1 SV=3 | 51,5 | 8,35 | 1053 | 24 | 1 | 1 | 1 | 1 | 1 | 3 |
| Q16659 | Mitogen-activated protein kinase 6 OS=Homo sapiens OX=9606 GN=MAPK6 PE=1 SV=1 | 82,6 | 5,03 | 191 | 24 | 1 | 1 | 1 | 1 | 1 | 1 |
| Q6PK04 | Coiled-coil domain-containing protein 137 OS=Homo sapiens OX=9606 GN=CCDC137 PE=1 SV=1 | 33,2 | 10,93 | 1091 | 24 | 1 | 1 | 1 | 1 | 1 | 6 |
| Q7L2J0 | 7SK snRNA methylphosphate capping enzyme OS=Homo sapiens OX=9606 GN=MEPCE PE=1 SV=1 | 74,3 | 9,57 | 608 | 24 | 1 | 1 | 1 | 1 | 1 | 3 |
| Q92878 | DNA repair protein RAD50 OS=Homo sapiens OX=9606 GN=RAD50 PE=1 SV=1 | 153,8 | 6,89 | 305 | 24 | 1 | 1 | 1 | 1 | 1 | 1 |
| Q96C01 | Protein FAM136A OS=Homo sapiens OX=9606 GN=FAM136A PE=1 SV=1 | 15,6 | 7,61 | 646 | 24 | 1 | 1 | 1 | 1 | 1 | 5 |
| Q96IJ6 | Mannose-1-phosphate guanyltransferase alpha OS=Homo sapiens OX=9606 GN=GMPPA PE=1 SV=1 | 46,3 | 7,21 | 942 | 24 | 1 | 1 | 1 | 1 | 1 | 3 |
| Q9BUN8 | Derlin-1 OS=Homo sapiens OX=9606 GN=DERL1 PE=1 SV=1 | 28,8 | 9,51 | 760 | 24 | 1 | 1 | 1 | 1 | 1 | 4 |
| Q9NWU2 | Glucose-induced degradation protein 8 homolog OS=Homo sapiens OX=9606 GN=GID8 PE=1 SV=1 | 26,7 | 4,97 | 386 | 24 | 1 | 1 | 1 | 1 | 1 | 4 |
| Q9UBU9 | Nuclear RNA export factor 1 OS=Homo sapiens OX=9606 GN=NXF1 PE=1 SV=1 | 70,1 | 8,51 | 62 | 24 | 1 | 1 | 1 | 1 | 1 | 2 |
| Q9UGP4 | LIM domain-containing protein 1 OS=Homo sapiens OX=9606 GN=LIMD1 PE=1 SV=1 | 72,1 | 6,65 | 889 | 24 | 1 | 1 | 1 | 1 | 1 | 2 |
| Q9Y679 | Lipid droplet-regulating VLDL assembly factor AUP1 OS=Homo sapiens OX=9606 GN=AUP1 PE=1 SV=2 | 45,8 | 8,65 | 584 | 24 | 1 | 1 | 1 | 1 | 1 | 3 |
| O15372 | Eukaryotic translation initiation factor 3 subunit H OS=Homo sapiens OX=9606 GN=EIF3H PE=1 SV=1 | 39,9 | 6,54 | 667 | 23 | 1 | 1 | 1 | 1 | 1 | 3 |
| O75683 | Surfeit locus protein 6 OS=Homo sapiens OX=9606 GN=SURF6 PE=1 SV=3 | 41,4 | 10,64 | 612 | 23 | 1 | 1 | 1 | 1 | 1 | 7 |
| P12273 | Prolactin-inducible protein OS=Homo sapiens OX=9606 GN=PIP PE=1 SV=1 | 16,6 | 8,05 | 441 | 23 | 1 | 1 | 1 | 1 | 1 | 11 |
| P49257 | Protein ERGIC-53 OS=Homo sapiens OX=9606 GN=LMAN1 PE=1 SV=2 | 57,5 | 6,77 | 76 | 23 | 1 | 1 | 1 | 1 | 1 | 2 |
| P55081 | Microfibrillar-associated protein 1 OS=Homo sapiens OX=9606 GN=MFAP1 PE=1 SV=2 | 51,9 | 4,98 | 918 | 23 | 1 | 1 | 1 | 1 | 1 | 3 |
| Q15125 | 3-beta-hydroxysteroid-Delta(8),Delta(7)-isomerase OS=Homo sapiens OX=9606 GN=EBP PE=1 SV=3 | 26,3 | 7,9 | 963 | 23 | 1 | 1 | 1 | 1 | 1 | 4 |
| Q8TF71 | Monocarboxylate transporter 10 OS=Homo sapiens OX=9606 GN=SLC16A10 PE=1 SV=1 | 55,5 | 7,69 | 168 | 23 | 1 | 1 | 1 | 1 | 1 | 2 |
| Q96KR1 | Zinc finger RNA-binding protein OS=Homo sapiens OX=9606 GN=ZFR PE=1 SV=2 | 116,9 | 9,04 | 945 | 23 | 1 | 1 | 1 | 1 | 1 | 1 |
| Q99442 | Translocation protein SEC62 OS=Homo sapiens OX=9606 GN=SEC62 PE=1 SV=1 | 45,8 | 7,12 | 496 | 23 | 1 | 1 | 1 | 1 | 1 | 2 |
| Q9GZP4 | PITH domain-containing protein 1 OS=Homo sapiens OX=9606 GN=PITHD1 PE=1 SV=1 | 24,2 | 5,74 | 303 | 23 | 1 | 1 | 1 | 1 | 1 | 3 |
| Q9P2I0 | Cleavage and polyadenylation specificity factor subunit 2 OS=Homo sapiens OX=9606 GN=CPSF2 PE=1 SV=2 | 88,4 | 5,11 | 272 | 23 | 1 | 1 | 1 | 1 | 1 | 2 |
| Q9UJA5 | tRNA (adenine(58)-N(1))-methyltransferase non-catalytic subunit TRM6 OS=Homo sapiens OX=9606 GN=TRMT6 PE=1 SV=1 | 55,8 | 7,55 | 707 | 23 | 1 | 1 | 1 | 1 | 1 | 3 |
| Q9Y6G3 | 39S ribosomal protein L42, mitochondrial OS=Homo sapiens OX=9606 GN=MRPL42 PE=1 SV=1 | 16,7 | 8,35 | 217 | 23 | 1 | 1 | 1 | 1 | 1 | 4 |
| O75251 | NADH dehydrogenase [ubiquinone] iron-sulfur protein 7, mitochondrial OS=Homo sapiens OX=9606 GN=NDUFS7 PE=1 SV=3 | 23,5 | 9,99 | 932 | 22 | 1 | 1 | 1 | 1 | 1 | 4 |
| P03496 | Non-structural protein 1 OS=Influenza A virus (strain A/Puerto Rico/8/1934 H1N1) OX=211044 GN=NS PE=1 SV=1 | 25,9 | 6,61 | 623 | 22 | 1 | 1 | 1 | 1 | 1 | 3 |
| P04083 | Annexin A1 OS=Homo sapiens OX=9606 GN=ANXA1 PE=1 SV=2 | 38,7 | 7,02 | 859 | 22 | 1 | 1 | 1 | 1 | 1 | 3 |
| P07476 | Involucrin OS=Homo sapiens OX=9606 GN=IVL PE=1 SV=2 | 68,4 | 4,61 | 1048 | 22 | 1 | 1 | 1 | 1 | 1 | 2 |
| P12814 | Alpha-actinin-1 OS=Homo sapiens OX=9606 GN=ACTN1 PE=1 SV=2 | 103 | 5,41 | 479 | 22 | 1 | 1 | 1 | 1 | 1 | 1 |
| P46109 | Crk-like protein OS=Homo sapiens OX=9606 GN=CRKL PE=1 SV=1 | 33,8 | 6,74 | 210 | 22 | 1 | 1 | 1 | 1 | 1 | 6 |
| P51617 | Interleukin-1 receptor-associated kinase 1 OS=Homo sapiens OX=9606 GN=IRAK1 PE=1 SV=2 | 76,5 | 6,62 | 927 | 22 | 1 | 1 | 1 | 1 | 1 | 2 |
| Q00341 | Vigilin OS=Homo sapiens OX=9606 GN=HDLBP PE=1 SV=2 | 141,4 | 6,87 | 21 | 22 | 1 | 1 | 1 | 1 | 1 | 1 |
| Q08J23 | RNA cytosine C(5)-methyltransferase NSUN2 OS=Homo sapiens OX=9606 GN=NSUN2 PE=1 SV=2 | 86,4 | 6,77 | 240 | 22 | 1 | 1 | 1 | 1 | 1 | 2 |
| Q15274 | Nicotinate-nucleotide pyrophosphorylase [carboxylating] OS=Homo sapiens OX=9606 GN=QPRT PE=1 SV=3 | 30,8 | 6,21 | 881 | 22 | 1 | 1 | 1 | 1 | 1 | 5 |
| Q5SWX8 | Protein odr-4 homolog OS=Homo sapiens OX=9606 GN=ODR4 PE=1 SV=1 | 51,1 | 5,92 | 349 | 22 | 1 | 1 | 1 | 1 | 1 | 2 |
| Q5UIP0 | Telomere-associated protein RIF1 OS=Homo sapiens OX=9606 GN=RIF1 PE=1 SV=2 | 274,3 | 5,52 | 575 | 22 | 1 | 1 | 1 | 1 | 1 | 1 |
| Q6PI48 | Aspartate--tRNA ligase, mitochondrial OS=Homo sapiens OX=9606 GN=DARS2 PE=1 SV=1 | 73,5 | 8,02 | 370 | 22 | 1 | 1 | 1 | 1 | 1 | 1 |
| Q7L8L6 | FAST kinase domain-containing protein 5, mitochondrial OS=Homo sapiens OX=9606 GN=FASTKD5 PE=1 SV=1 | 86,5 | 8,13 | 107 | 22 | 1 | 1 | 1 | 1 | 1 | 1 |
| Q96JP5 | E3 ubiquitin-protein ligase ZFP91 OS=Homo sapiens OX=9606 GN=ZFP91 PE=1 SV=1 | 63,4 | 7,36 | 1066 | 22 | 1 | 1 | 1 | 1 | 1 | 2 |
| Q96MX6 | Dynein axonemal assembly factor 10 OS=Homo sapiens OX=9606 GN=DNAAF10 PE=1 SV=1 | 39,7 | 8,09 | 464 | 22 | 1 | 1 | 1 | 1 | 1 | 4 |
| Q9BYE7 | Polycomb group RING finger protein 6 OS=Homo sapiens OX=9606 GN=PCGF6 PE=1 SV=2 | 39 | 4,97 | 458 | 22 | 1 | 1 | 1 | 1 | 1 | 3 |
| Q9BZI7 | Regulator of nonsense transcripts 3B OS=Homo sapiens OX=9606 GN=UPF3B PE=1 SV=1 | 57,7 | 9,48 | 319 | 22 | 1 | 1 | 1 | 1 | 1 | 2 |
| Q9H3U1 | Protein unc-45 homolog A OS=Homo sapiens OX=9606 GN=UNC45A PE=1 SV=1 | 103 | 6,07 | 58 | 22 | 1 | 1 | 1 | 1 | 1 | 2 |
| P13861 | cAMP-dependent protein kinase type II-alpha regulatory subunit OS=Homo sapiens OX=9606 GN=PRKAR2A PE=1 SV=2 | 45,5 | 5,07 | 741 | 21 | 1 | 1 | 1 | 1 | 1 | 3 |
| P29558 | RNA-binding motif, single-stranded-interacting protein 1 OS=Homo sapiens OX=9606 GN=RBMS1 PE=1 SV=3 | 44,5 | 8,79 | 431 | 21 | 1 | 1 | 1 | 1 | 1 | 4 |
| Q15014 | Mortality factor 4-like protein 2 OS=Homo sapiens OX=9606 GN=MORF4L2 PE=1 SV=1 | 32,3 | 9,72 | 427 | 21 | 1 | 1 | 1 | 1 | 1 | 4 |
| Q15050 | Ribosome biogenesis regulatory protein homolog OS=Homo sapiens OX=9606 GN=RRS1 PE=1 SV=2 | 41,2 | 10,7 | 208 | 21 | 1 | 1 | 1 | 1 | 1 | 2 |
| Q6W2J9 | BCL-6 corepressor OS=Homo sapiens OX=9606 GN=BCOR PE=1 SV=1 | 192,1 | 6,48 | 417 | 21 | 1 | 1 | 1 | 1 | 1 | 1 |
| Q8IXM3 | 39S ribosomal protein L41, mitochondrial OS=Homo sapiens OX=9606 GN=MRPL41 PE=1 SV=1 | 15,4 | 9,57 | 904 | 21 | 1 | 1 | 1 | 1 | 1 | 7 |
| Q8WVX9 | Fatty acyl-CoA reductase 1 OS=Homo sapiens OX=9606 GN=FAR1 PE=1 SV=1 | 59,3 | 9,17 | 649 | 21 | 1 | 1 | 1 | 1 | 1 | 2 |
| Q9ULW0 | Targeting protein for Xklp2 OS=Homo sapiens OX=9606 GN=TPX2 PE=1 SV=2 | 85,6 | 9,23 | 448 | 21 | 1 | 1 | 1 | 1 | 1 | 1 |
| Q9UMS4 | Pre-mRNA-processing factor 19 OS=Homo sapiens OX=9606 GN=PRPF19 PE=1 SV=1 | 55,1 | 6,61 | 435 | 21 | 1 | 1 | 1 | 1 | 1 | 6 |
| P49756 | RNA-binding protein 25 OS=Homo sapiens OX=9606 GN=RBM25 PE=1 SV=3 | 100,1 | 6,32 | 587 | 20 | 1 | 1 | 1 | 1 | 1 | 1 |

**Table B. Qualitative proteomic analysis of proteins detected exclusively in TRIM34-FLAG–overexpressing cells.** Proteins were identified by mass spectrometry following FLAG pull-down, and only those uniquely present in TRIM34-FLAG–overexpressing cells compared with control cells (transfected with the empty plasmid) are shown. Protein accession, description, molecular weight, isoelectric point, group ID, Mascot score, PSMs, peptides, unique peptides, and sequence coverage are shown, ordered by Mascot score.

| **MOCK** | | | | | |  |
| --- | --- | --- | --- | --- | --- | --- |
| **ENRICHED GO BIOLOGICAL PROCESSES** | **FDR** | **(-log10 FDR)** | **FOLD ENRICHMENT** | **GENES** | **COUNT** | |
| GO:0032543~mitochondrial translation | 3,60E-22 | 21,4439 | 12,5365 | Q96A35, Q96DV4, P54136, Q6P1L8, Q8IXM3, Q9BYC9, Q9P0J6, O60783, Q14197, Q9HD33, P82663, P82664, Q13084, Q9H9J2, Q9NYK5, Q96GC5, P82921, P82912, Q96EY7, P51398, Q9BYD6, Q9Y3B7, Q9BYN8, Q9P0M9, Q9Y3D3, Q9BYD3, P82650, Q92665, P82673, Q7Z7F7, P82932, Q16540 | 32 | |
| GO:0006412~translation | 1,57E-12 | 11,8036 | 6,0821 | Q96A35, Q71RC2, Q12904, Q8IXM3, Q9BYC9, Q04637, Q9P0J6, P63220, O60783, P47897, P15170, Q13084, P82921, P82912, Q9H7H0, Q8NE71, Q9BYD6, Q8N5C6, Q9Y3B7, P62861, Q9P0M9, Q9Y3D3, P63173, Q9BYD3, Q13155, P05386, P46777, P60866, Q7Z7F7, P82932, Q9P2E9, Q16540 | 32 | |
| GO:0006364~rRNA processing | 1,34E-10 | 9,8718 | 7,2554 | P38919, P78346, Q13610, Q9Y2L1, O43159, Q14978, Q13601, O00541, Q8TDN6, P57678, Q8TEX9, Q9UKD2, O60832, Q9BUL9, Q8IZL8, Q92841, Q9Y2W2, Q9Y2X3, Q92499, Q9BV38, Q9Y4W2, P46777, Q14684, Q9UQ80 | 24 | |
| GO:0000398~mRNA splicing, via spliceosome | 2,52E-10 | 9,5991 | 5,5119 | P52597, P52298, Q9UQ35, O75643, Q9H307, Q9Y5S9, O00148, Q8IYB3, Q15020, P09012, P09651, P62995, P62314, O94906, P08621, P38919, O43660, Q9UMS4, O75533, Q15427, Q9UKM9, P38159, Q8WXD5, Q9BTD8, Q07955, Q15393, P26368, Q13595, P62304 | 29 | |
| **GO:0006913~nucleocytoplasmic transport** | 4,73E-09 | 8,3252 | 11,9978 | O14980, Q8TEM1, Q12769, Q8N1F7, P35658, P78406, P55735, P37198, Q99567, Q8WYP5, Q7Z3B4, Q92621, Q5SRE5, Q8WUM0 | 15 | |
| GO:0000387~spliceosomal snRNP assembly | 3,92E-08 | 7,4070 | 15,3572 | Q16637, P38432, Q9Y3F4, O14893, Q15020, Q9UHI6, P57678, P62314, Q9NWZ8, Q8WXD5, P62304 | 12 | |
| GO:0000245~spliceosomal complex assembly | 3,25E-07 | 6,4884 | 15,0830 | Q16637, Q9UMS4, O75533, Q92499, O14893, P26368, P62314, O94906, Q8WXD5, P62304 | 11 | |
| GO:0043161~proteasome-mediated ubiquitin-dependent protein catabolic process | 2,26E-06 | 5,6455 | 4,0089 | O00487, P48729, P62714, P25686, P25787, Q96CS3, O43242, P09936, O43164, P62191, Q9UNM6, P28074, P54727, P17980, O00232, P28072, P28070, Q9UNE7, Q99436, O95071, P48556, P46379, P49721, P49720, P60900, P43686 | 26 | |
| GO:0008380~RNA splicing | 3,29E-06 | 5,4823 | 4,3932 | Q16637, P38919, P52298, Q4G0J3, O75533, Q9H307, Q15427, O95232, Q9Y5S9, Q9NW13, Q8N163, Q07955, Q8IYB3, O14893, Q9Y2W2, Q15393, P26368, Q14498, Q14684, P62314, Q9Y2W1, O94906 | 23 | |
| GO:0051028~mRNA transport | 3,02E-05 | 4,5205 | 6,6358 | Q8TEM1, Q9Y6M1, P51114, P55735, Q9Y5S9, P37198, Q9BZI7, Q07955, Q8WYP5, Q7Z3B4, Q92621, Q5SRE5, P09651, Q9UN86 | 14 | |
| GO:0009060~aerobic respiration | 3,02E-05 | 4,5205 | 7,3398 | P30566, P56556, P28331, O75489, O95140, O95299, Q9P0J0, P21912, O75380, P19404, Q16795, P49821, P22695 | 13 | |
| **GO:0006406~mRNA export from nucleus** | 8,59E-05 | 4,0660 | 7,4309 | P38919, P52298, Q99567, O14980, Q12769, P35658, Q92900, P78406, Q8WUM0, Q9Y5S9, O00148 | 12 | |
| GO:0021762~substantia nigra development | 5,40E-04 | 3,2676 | 7,9985 | P09936, P63167, P24539, O75489, O15027, P09543, Q6S8J3, P12277, P62258, P60953 | 10 | |
| **GO:0006606~protein import into nucleus** | 6,06E-04 | 3,2177 | 5,0234 | Q13501, Q8N1F7, P35658, Q8TEX9, Q96P70, P55735, O95373, P37198, Q99567, O60684, Q92973, P52292, Q5SRE5, Q8WUM0 | 14 | |
| GO:0006397~mRNA processing | 7,34E-04 | 3,1342 | 3,3454 | Q16637, Q4G0J3, Q9UQ35, Q08J23, O75643, Q7L0Y3, Q9H307, Q15427, Q9Y5S9, Q9NW13, Q8N163, Q07955, Q8IYB3, O14893, Q9Y2W2, Q92499, P26368, Q14498, Q14684, Q9Y2W1 | 21 | |
| GO:0042776~proton motive force-driven mitochondrial ATP synthesis | 0,0011 | 2,9659 | 6,3033 | P21912, O75380, P19404, P56556, P24539, P28331, O75489, Q16795, P49821, O95299, Q9P0J0 | 11 | |
| GO:0006446~regulation of translational initiation | 0,0014 | 2,8493 | 9,9079 | Q04637, O15372, P52298, P55884, P23588, Q92499, Q9UBQ5, P60228 | 8 | |
| **GO:0006405~RNA export from nucleus** | 0,0018 | 2,7497 | 12,2160 | P37198, Q5SRE5, P09651, P35658, P78406, O00148 | 7 | |
| GO:0000375~RNA splicing, via transesterification reactions | 0,0037 | 2,4265 | 10,7500 | O75533, Q15427, Q8IYB3, O14893, Q15393, P62995, O94906 | 7 | |
| GO:0006417~regulation of translation | 0,0045 | 2,3516 | 4,7496 | Q96EY7, Q96DH6, Q92616, Q9H0A0, P63173, Q14978, Q9BRX2, O43347, Q9Y6M1, Q7L576, Q9Y5S9, Q9UQ80 | 12 | |
| GO:0000184~nuclear-transcribed mRNA catabolic process, nonsense-mediated decay | 0,0075 | 2,1269 | 7,4913 | P38919, P52298, Q9BZI7, P15170, Q9BRP8, Q92900, P60228, Q9Y5S9 | 8 | |
| GO:0032435~negative regulation of proteasomal ubiquitin-dependent protein catabolic process | 0,0075 | 2,1269 | 7,4913 | Q9UL15, O15372, P68400, P67870, Q8N163, P46379, Q93008 | 8 | |
| GO:0006120~mitochondrial electron transport, NADH to ubiquinone | 0,0152 | 1,8182 | 6,6770 | O75380, P19404, P56556, P28331, O75489, Q16795, P49821, O95299 | 8 | |
| GO:0061014~positive regulation of mRNA catabolic process | 0,0161 | 1,7931 | 15,9971 | Q9HCE1, Q7Z2W4, O00178, Q92900, A5YKK6 | 5 | |
| GO:0045727~positive regulation of translation | 0,0205 | 1,6890 | 4,7399 | P38919, Q71RC2, Q9H0A0, Q9BZI7, Q8NE71, Q9BRP8, P46777, P51114, P60228, P05455 | 10 | |
| GO:0018279~protein N-linked glycosylation via asparagine | 0,0258 | 1,5883 | 9,5982 | Q9H0U3, P04844, O60762, P39656, Q9NRP0, P46977 | 6 | |
| GO:0006511~ubiquitin-dependent protein catabolic process | 0,0344 | 1,4635 | 2,7774 | Q9H0E2, Q13501, O43242, P09936, Q7L5D6, Q9UNM6, Q86YT6, P11441, O00487, Q99942, Q9UNE7, Q96T88, Q7Z6Z7, P46379, P60900, P25787, Q15386 | 17 | |
| GO:0048026~positive regulation of mRNA splicing, via spliceosome | 0,0344 | 1,4635 | 6,8911 | Q9UMS4, Q15427, P38159, P62995, Q13595, Q9Y2W1, P08621 | 7 | |
| GO:0032922~circadian regulation of gene expression | 0,0346 | 1,4612 | 4,8667 | Q92769, P43490, Q9Y5V3, Q13547, Q7Z6Z7, Q9BQG0, P36873, P48730, O75592 | 9 | |
| GO:0051301~cell division | 0,0346 | 1,4612 | 2,2936 | O95347, Q14204, Q9UBB4, Q08J23, O43684, Q9BRX2, Q8IZT6, P36873, Q9NRZ9, P78406, P48729, Q9NR09, P52926, Q9NTJ3, Q8WYP5, Q15003, Q93008, Q14683, Q9BW19, Q9BQE3 | 23 | |
| GO:0006413~translational initiation | 0,0346 | 1,4604 | 5,5844 | Q04637, O15372, P55884, P23588, Q9Y262, Q8NE71, Q9UBQ5, P60228 | 8 | |
| GO:0045333~cellular respiration | 0,0346 | 1,4604 | 6,7188 | Q9UDW1, P28331, P08574, P00403, Q9UJS0, P22695 | 7 | |
| GO:0036503~ERAD pathway | 0,0422 | 1,3750 | 4,1283 | Q99942, Q9UNE7, Q9HCN8, Q96CS3, Q9Y679, Q7L5D6, P46379, P25686, O94905, P60468 | 10 | |
| GO:0032508~DNA duplex unwinding | 0,0453 | 1,3441 | 5,2058 | P35250, P40938, P04083, Q7L2E3, Q92499, Q92900, P53999, P35249 | 8 | |
| GO:0006986~response to unfolded protein | 0,0453 | 1,3441 | 5,2058 | P31689, Q96CS3, Q9UDY4, P07900, O95140, Q15629, P25686, P50454 | 8 | |
| GO:0001732~formation of cytoplasmic translation initiation complex | 0,0453 | 1,3441 | 11,2921 | O15372, P55884, Q9Y262, Q9UBQ5, P60228 | 5 | |
| GO:0007049~cell cycle | 0,0474 | 1,3247 | 2,3699 | O95347, Q14676, Q9BRX2, Q9UPN4, P36873, Q09028, Q9NRZ9, Q8IXM3, O75351, P78406, P48729, Q96T88, P68400, Q9NR09, Q8N163, P52926, Q9NVI1, Q8WYP5, P61289 | 20 | |
| **IAV** | | | | | |  |
| **ENRICHED GO BIOLOGICAL PROCESSES** | **FDR** | **(-log10 FDR)** | **FOLD ENRICHMENT** | **GENES** | **COUNT** | |
| GO:0000398~mRNA splicing, via spliceosome | 2,28E-27 | 26,6418 | 6,6040 | Q15459, P52597, O43395, Q16629, Q9UJV9, Q53GS9, Q9UQ35, P52272, Q9P013, O00148, Q96DI7, P84103, Q15020, P09012, Q96A72, Q13243, P62995, O94906, Q13247, Q12874, Q15029, Q8WXF0, Q9UKM9, O43447, Q6P2Q9, P09661, Q13573, O43172, P08579, P55081, P52298, O75643, Q9H307, O60506, Q9Y5S9, Q9Y3C6, Q8WU68, P17844, Q96PK6, P09234, P61326, Q15287, P08621, O43143, O43660, Q9UMS4, P31943, Q8NAV1, Q8IW35, Q8WXD5, Q99459, Q07955, P26368, Q9BZJ0, Q9ULR0, P62304 | 58 | |
| GO:0032543~mitochondrial translation | 4,63E-27 | 26,3346 | 9,8571 | Q96DV4, P54136, Q7Z2W9, Q8IXM3, Q9H0U6, Q9Y2Q9, Q9H2W6, Q9P015, Q9NVS2, Q9NWU5, Q9Y6G3, Q9BZE1, O60783, P82664, Q92552, Q9H9J2, Q9NYK5, Q96GC5, P82921, Q8N983, Q96EY7, Q9BQ48, Q9Y676, P82914, Q9NP92, P51398, Q9NRX2, Q9NQ50, Q9Y399, Q9Y2R9, Q9Y3D9, Q9BYN8, Q9P0M9, Q9Y3D3, Q9BYD3, P82650, Q92665, P82673, P09001, Q7Z7F7, P49406, P82933 | 42 | |
| GO:0006412~translation | 6,18E-26 | 25,2092 | 6,3762 | O00425, Q9NSD9, Q12904, Q9H0U6, Q9P015, Q8IXM3, Q9NVS2, Q04637, Q9NWU5, Q9Y6G3, P62273, Q9BZE1, P62910, P82921, Q8N983, Q07020, Q9Y676, P82914, O43324, Q9NRX2, Q9Y399, P46781, P36578, Q8NE71, P62244, P84098, P61313, P05386, P46777, P27635, P62841, P49406, P32969, O60783, P63220, P47897, P62854, Q9BQ48, Q9NP92, P08708, P62829, P14868, Q9Y2R9, P42677, Q9Y3D9, Q9P0M9, Q9Y3D3, Q9BYD3, P09001, Q13155, Q7Z7F7, Q13310, Q9P2E9, Q969Q0, P82933 | 56 | |
| GO:0008380~RNA splicing | 3,18E-13 | 12,4974 | 4,6915 | P52298, O43395, P35637, Q16629, O43251, Q53GS9, Q14966, Q9H307, P18583, Q9H0S4, O95232, O60506, Q9Y5S9, Q96DI7, P49756, Q96A72, P26599, Q14498, P61326, O94906, Q15287, Q13148, Q16637, O43143, Q96SB4, Q92945, Q5BKZ1, Q9GZT3, Q69YN4, Q96J01, Q99873, Q07955, Q9UKV3, Q9Y6Y0, Q6P2Q9, O14893, P09661, P26368, O43172, Q9ULX6 | 41 | |
| GO:0045653~negative regulation of megakaryocyte differentiation | 1,08E-12 | 11,9647 | 17,2500 | P62805, Q99873 | 15 | |
| GO:0006364~rRNA processing | 1,08E-12 | 11,9647 | 5,7953 | Q9H0S4, Q06265, O00567, Q9UKD2, Q9Y2X3, Q92499, Q15024, O75607, P78316, Q9Y2L1, P46087, Q13868, Q14978, P08708, Q13601, Q01780, Q9NR30, Q8TDN6, Q9NQT4, Q9NQ55, P57678, Q8TEX9, O60832, P42677, Q9Y3B2, Q92841, Q5JTH9, Q9ULX3, Q9BV38, Q9Y4W2, P46777, P62841 | 32 | |
| GO:0043161~proteasome-mediated ubiquitin-dependent protein catabolic process | 2,78E-12 | 11,5559 | 4,0643 | P28066, P51665, P35998, O00487, Q9NWU2, P48729, P62333, P62195, P20618, Q9H7D7, P63208, Q16531, P25788, P62714, P55072, Q13200, Q15366, P25787, P25786, Q15008, O43242, O43164, P55036, Q9BV68, O00231, P62191, Q9UNM6, P28074, P54727, P17980, Q99460, O00232, P28070, Q9UNE7, Q99436, O14818, O95071, P48556, P46379, P49720, P60900, P43686, Q16186, Q9BUN8 | 44 | |
| GO:0006457~protein folding | 2,90E-12 | 11,5378 | 4,8994 | O95433, P62937, O95757, Q96A33, Q99471, O60925, P30101, Q9Y3C6, Q15084, Q92598, O95816, O15212, Q9Y230, P27824, P07237, P31689, P23284, Q92945, P49257, Q14318, P14625, O43447, Q9Y4L1, P34932, Q8NBS9, Q96EY1, P49368, P61758, Q9UHV9, P08238, P10809, P27797, Q13451, Q99615, P62942 | 36 | |
| GO:0061644~protein localization to CENP-A containing chromatin | 3,75E-12 | 11,4265 | 17,8889 | P62805 | 14 | |
| GO:0006397~mRNA processing | 6,63E-11 | 10,1787 | 3,9129 | Q15459, O43395, Q16629, O43251, Q53GS9, Q9UQ35, Q7L0Y3, P55265, O75643, O75940, Q9H307, P18583, Q9H0S4, Q9Y5S9, P49756, Q92499, Q75N03, Q6UN15, P26599, Q13243, Q14498, Q10570, Q13148, Q16637, O43143, Q12874, Q92945, Q08J23, Q5BKZ1, Q69YN4, Q96J01, P14866, Q9UJA5, Q07955, Q9UKV3, Q6P2Q9, O14893, P26368, Q7L8L6, Q9ULX6 | 41 | |
| GO:0042776~proton motive force-driven mitochondrial ATP synthesis | 9,37E-10 | 9,0281 | 7,2090 | O75947, O75306, P28331, P48047, P56134, O75489, O43920, P36542, O95299, Q9P0J0, Q5VTU8, O96000, O75251, P21912, O75380, P19404, P24539, P31040, Q9UJZ1, P56385, P49821 | 21 | |
| GO:0032200~telomere organization | 4,56E-09 | 8,3411 | 11,9259 | P62805 | 14 | |
| GO:0002181~cytoplasmic translation | 1,75E-07 | 6,7565 | 5,2165 | Q07020, P32969, P08708, P62829, P46781, P36578, O00178, P42677, P62244, P84098, P62273, P63220, P62910, Q6P5R6, P61313, Q9Y295, P05386, P46777, P62841, P27635, P62854 | 22 | |
| GO:0006413~translational initiation | 1,47E-06 | 5,8341 | 6,6909 | O15371, O15372, Q13347, P55884, O75821, P08708, O00571, Q8NE71, P41091, Q04637, P61221, Q9Y262, Q9UBQ5, P60228, P60842, P06730 | 16 | |
| GO:0000381~regulation of alternative mRNA splicing, via spliceosome | 1,72E-06 | 5,7643 | 6,1094 | Q15459, Q13247, O43251, Q92945, Q8WXF0, O75940, P14866, P51114, Q9Y5S9, P17844, P49756, Q92841, P26599, P62995, Q15287, Q96T37, P61326 | 17 | |
| GO:0000245~spliceosomal complex assembly | 1,78E-06 | 5,7500 | 9,8571 | Q16637, Q9UMS4, Q96SB4, Q53GS9, Q92499, O14893, P26368, Q9BZJ0, O94906, Q8WXD5, P62304 | 12 | |
| GO:0050821~protein stabilization | 8,49E-06 | 5,0712 | 3,0795 | O43670, P04637, Q96MX6, P13473, Q9BZE4, Q02413, O95816, O15212, Q9Y230, Q93009, P35080, Q14574, Q93008, Q9UBK9, P23284, P62829, Q9H6T3, P19388, P51610, Q9UNE7, P68400, Q99497, O15027, Q96EY1, P46379, P49368, P46777, Q9UHV9, P08238, P10809, P27797 | 32 | |
| GO:0006334~nucleosome assembly | 1,88E-05 | 4,7265 | 3,5714 | Q99733, P26583, Q9BQ67, P62805, P49321, O14929, O60264, Q15020, Q09028, P55209, Q9Y5B9, P16402 | 25 | |
| **GO:0006606~protein import into nucleus** | 2,24E-05 | 4,6507 | 4,2991 | Q13501, Q14974, P62826, O15397, P55265, P04637, Q8N1F7, P35658, O00410, Q8TEX9, Q96P70, O95373, P37198, Q99567, O60684, Q92973, P52292, Q5SRE5, P02545, P55060 | 20 | |
| GO:0009060~aerobic respiration | 2,26E-05 | 4,6467 | 5,4118 | P30566, O75306, P28331, P14927, O75489, O43920, O95299, Q9P0J0, O96000, O75251, P21912, O75380, P19404, Q15070, P49821, P22695 | 16 | |
| GO:0034975~protein folding in endoplasmic reticulum | 2,69E-05 | 4,5710 | 12,1765 | Q8N766, P11021, Q15006, Q8IXB1, P14625, P27824, P30101, P07237, P27797 | 9 | |
| GO:0061077~chaperone-mediated protein folding | 3,89E-05 | 4,4098 | 6,6444 | P23284, Q8WWF6, Q99471, O60925, Q9H3U1, P68400, O15212, P49368, P61758, P04792, Q9UHV9, P08238, Q13451 | 13 | |
| GO:0000380~alternative mRNA splicing, via spliceosome | 6,43E-05 | 4,1919 | 9,2000 | Q13247, P52298, P17844, Q9Y3F4, P52272, Q07955, Q92841, Q9BUJ2, Q96I25, Q1KMD3 | 10 | |
| GO:0000375~RNA splicing, via transesterification reactions | 6,43E-05 | 4,1919 | 9,2000 | Q12874, Q96DI7, O43395, Q92945, O75940, Q6P2Q9, O14893, P62995, O43172, O94906 | 10 | |
| GO:0042274~ribosomal small subunit biogenesis | 8,35E-05 | 4,0781 | 4,8421 | Q9UNQ2, P78316, P08708, P46781, Q13601, Q01780, O75683, Q9Y2P8, Q9H583, Q8TEX9, O00567, P42677, P62244, Q9Y2X3, P62841, Q96GA3 | 16 | |
| **GO:0006406~mRNA export from nucleus** | 2,13E-04 | 3,6725 | 5,1935 | P52298, O14980, Q96J01, P35658, P78406, O00148, Q9Y5S9, Q99567, P84103, Q9UBU9, Q96A72, P61326, P06730 | 14 | |
| GO:0036503~ERAD pathway | 2,29E-04 | 3,6398 | 4,2043 | Q9Y679, Q7L5D6, Q96A33, P14625, Q8TCJ2, Q9NXW2, Q99942, Q9UNE7, P11021, P62333, Q8IXB1, P46379, P27824, P55072, Q9BUN8, P27797, P60468 | 17 | |
| GO:0000387~spliceosomal snRNP assembly | 3,17E-04 | 3,4986 | 7,6667 | Q16637, Q9Y3F4, P09234, O14893, Q15020, Q9UHI6, P57678, Q8WXD5, P62304 | 10 | |
| GO:0001732~formation of cytoplasmic translation initiation complex | 3,49E-04 | 3,4567 | 10,8235 | O15371, O15372, Q13347, P55884, O75821, Q9Y262, Q9UBQ5, P60228 | 8 | |
| GO:0006401~RNA catabolic process | 5,37E-04 | 3,2702 | 7,1875 | Q14103, Q9Y2L1, Q13868, Q9Y3B2, Q01780, Q9NQT4, Q06265, Q13310, Q96B26, Q15024 | 10 | |
| GO:0006325~chromatin organization | 5,95E-04 | 3,2254 | 2,7944 | O15294, P26583, Q15424, P35659, P62805, O94900, O94776, O14929, Q13185, O60264, P46379, Q15014, Q13263 | 26 | |
| GO:0032508~DNA duplex unwinding | 5,95E-04 | 3,2254 | 5,0678 | Q8N9N2, P12956, P40937, P33993, O00571, P35249, Q96S55, P35250, P04083, Q13283, Q92499, Q9Y230, Q92878 | 13 | |
| GO:0032435~negative regulation of proteasomal ubiquitin-dependent protein catabolic process | 6,06E-04 | 3,2176 | 6,1707 | O15294, O15372, P68400, P67870, Q99497, P46379, Q9Y5K5, Q93009, P08238, Q93008 | 11 | |
| GO:0045727~positive regulation of translation | 7,32E-04 | 3,1356 | 4,2593 | Q7Z739, O15371, O15294, Q14103, Q92615, P13639, Q04695, O00571, Q8NE71, P51114, P42677, Q99873, Q9BZI7, P46777, P60228 | 15 | |
| GO:0034976~response to endoplasmic reticulum stress | 0,0012 | 2,9121 | 3,8333 | Q13148, P51572, Q9Y239, P14625, P16615, P30101, Q9Y4L1, P10145, P11021, Q15084, Q8IXB1, P57088, O15027, P62714, P29966, P07237 | 16 | |
| GO:0034063~stress granule assembly | 0,0013 | 2,8835 | 7,3929 | Q7Z739, Q14204, Q8TB72, P26196, Q9Y520, Q13283, O00571, Q8ND56, Q14157 | 9 | |
| GO:1990000~amyloid fibril formation | 0,0022 | 2,6656 | 6,9000 | Q13148, P35637, P04080, Q15517, P06396, Q93008, P62942 | 9 | |
| GO:0010498~proteasomal protein catabolic process | 0,0022 | 2,6656 | 6,9000 | Q9UMS4, O14818, P25788, Q16531, P46379, P55072, P60900, P25787, P25786 | 9 | |
| **GO:0006913~nucleocytoplasmic transport** | 0,0023 | 2,6438 | 5,2708 | P37198, P46060, Q99567, O14980, Q92621, Q5SRE5, Q8N1F7, P35658, P43487, P78406 | 11 | |
| GO:0006986~response to unfolded protein | 0,0027 | 2,5682 | 4,6780 | P31689, O95757, P17066, Q92598, Q8TCJ2, P04792, P50454, P25685, P34932, P08238, P10809, Q9BUN8 | 12 | |
| GO:0006888~endoplasmic reticulum to Golgi vesicle-mediated transport | 0,0035 | 2,4574 | 3,1603 | P53621, P51572, Q9Y678, Q969M3, P49257, P33947, P18085, P53618, O75340, O75396, Q9Y4L1, Q15363, O15027, P49755, Q9BVK6, P55072, P62820, P07237 | 18 | |
| GO:0000027~ribosomal large subunit assembly | 0,0050 | 2,3017 | 8,9444 | P46087, Q15050, Q8TDN6, Q9NQ55, P46777, P27635 | 7 | |
| GO:0006260~DNA replication | 0,0052 | 2,2814 | 3,5204 | Q14566, Q9BQ67, P40937, P49321, P28340, P33992, P33993, Q09028, P55209, P11387, P06493, Q16576, Q9Y3Z3, Q9Y5B9, P29558 | 15 | |
| GO:0000956~nuclear-transcribed mRNA catabolic process | 0,0055 | 2,2575 | 7,0769 | P17844, Q13868, Q01780, Q9NQT4, Q06265, Q96B26, Q15024, Q9UKD2 | 8 | |
| GO:0006511~ubiquitin-dependent protein catabolic process | 0,0057 | 2,2472 | 2,4468 | Q7L5D6, Q9Y5K5, P11441, P35998, O00487, Q99942, Q7Z6Z7, P63208, Q16531, P25788, P55072, P25787, Q15386, P25786, Q13501, O43242, Q9UII4, Q9BV68, O00231, Q9UNM6, O00233, Q9UNE7, O14818, P46379, P60900 | 25 | |
| GO:0071038~TRAMP-dependent tRNA surveillance pathway | 0,0064 | 2,1966 | 16,4286 | Q13868, Q01780, Q06265, Q96B26, Q15024 | 5 | |
| GO:0071035~nuclear polyadenylation-dependent rRNA catabolic process | 0,0064 | 2,1966 | 16,4286 | Q13868, Q01780, Q06265, Q96B26, Q15024 | 5 | |
| GO:0043484~regulation of RNA splicing | 0,0064 | 2,1965 | 3,8831 | P52597, P31943, P35637, P55795, Q14966, Q5BKZ1, P18583, Q09666, P14866, Q07955, P26599, P62995, P08621 | 13 | |
| GO:0006120~mitochondrial electron transport, NADH to ubiquinone | 0,0075 | 2,1262 | 5,0000 | O75380, P19404, O75306, P28331, O75489, P49821, O43920, O95299, O75251, O96000 | 10 | |
| GO:0061621~canonical glycolysis | 0,0080 | 2,0987 | 8,0500 | P06733, P18669, P14618, P17858, P08237, Q01813, P52789 | 7 | |
| GO:1901800~positive regulation of proteasomal protein catabolic process | 0,0080 | 2,0987 | 8,0500 | P62333, P62195, P62191, O95816, P17980, P35998, P43686 | 7 | |
| GO:0051301~cell division | 0,0086 | 2,0646 | 2,0312 | Q14204, O43670, Q14203, Q13257, Q53GS9, P33992, P78406, Q9P258, P48729, Q9Y6G9, Q9NR09, P11171, P09496, Q9ULW0, Q15003, Q93008, Q9BQE3, O00139, O95347, P62826, O95229, Q08J23, Q7Z4H7, O43684, Q99459, P62140, Q5T9A4, P27816, Q9NTJ3, Q99871, P06493 | 34 | |
| GO:0051028~mRNA transport | 0,0092 | 2,0364 | 3,6914 | O00425, Q16629, Q9NZI8, Q92945, P51114, Q9Y5S9, P37198, P42704, Q9BZI7, Q07955, Q92621, Q5SRE5, P06730 | 13 | |
| GO:0006099~tricarboxylic acid cycle | 0,0092 | 2,0364 | 5,4474 | P21912, O75390, P08559, P36957, P10515, P31040, P11177, O43837, Q9P2R7 | 9 | |
| GO:0045070~positive regulation of viral genome replication | 0,0092 | 2,0364 | 6,3448 | Q96SB4, P62937, P55265, O00571, Q16531, O43447, Q9P035, O95793 | 8 | |
| GO:0042026~protein refolding | 0,0092 | 2,0364 | 6,3448 | P31689, P11021, O60884, P17066, P04792, P10809, P62942 | 8 | |
| GO:0010466~negative regulation of peptidase activity | 0,0101 | 1,9945 | 14,3750 | P01040, P04080, P48594, P29508, Q16610 | 5 | |
| GO:0006164~purine nucleotide biosynthetic process | 0,0102 | 1,9915 | 9,8571 | P30566, P00492, Q14558, P22102, P11908, P11586 | 6 | |
| GO:0006281~DNA repair | 0,0121 | 1,9172 | 2,2397 | P28340, Q9Y5K5, O60264, Q16531, Q9Y230, P55072, Q92878, Q9Y3Z3, Q13263, Q9UMS4, P49674, P40937, O96019, Q01780, P52701, P35249, P35250, Q9UNE7, Q99459, Q5UIP0, O95071, Q99497, P06493, Q15014, Q9Y5B9 | 26 | |
| GO:0048025~negative regulation of mRNA splicing, via spliceosome | 0,0121 | 1,9172 | 7,3182 | Q13247, Q16629, Q8WXF0, Q9UKV3, P26368, P26599, Q15287 | 7 | |
| GO:0006446~regulation of translational initiation | 0,0132 | 1,8799 | 5,9355 | Q04637, O15372, P52298, P55884, Q92499, Q9UBQ5, P04792, P60228 | 8 | |
| GO:1902600~proton transmembrane transport | 0,0155 | 1,8108 | 2,8129 | O75947, P48047, P56134, Q9H1K4, P36542, P12235, P53985, Q5VTU8, P13073, P24539, Q00325, P56385, P00403, Q9UJS0, P05023, P0C7P4 | 17 | |
| GO:0000467~exonucleolytic trimming to generate mature 3'-end of 5.8S rRNA from tricistronic rRNA transcript (SSU-rRNA, 5.8S rRNA, LSU-rRNA) | 0,0157 | 1,8048 | 12,7778 | Q13868, Q01780, Q06265, Q96B26, Q15024 | 5 | |
| GO:0000056~ribosomal small subunit export from nucleus | 0,0157 | 1,8048 | 12,7778 | Q99567, P62826, O14980, P62841, Q96GA3 | 5 | |
| GO:0018279~protein N-linked glycosylation via asparagine | 0,0187 | 1,7280 | 6,7083 | P04843, Q9H0U3, P04844, O60762, Q8TCJ2, P39656, P46977 | 7 | |
| GO:0000413~protein peptidyl-prolyl isomerization | 0,0234 | 1,6315 | 6,4400 | Q9Y3C6, P23284, P62937, O43447, Q13451, P62942 | 7 | |
| GO:0048511~rhythmic process | 0,0235 | 1,6296 | 3,4937 | Q13148, P17844, P68400, Q8WXF1, P04637, P06493, Q13185, Q16531, Q93009, Q93008, P11387 | 12 | |
| GO:0032981~mitochondrial respiratory chain complex I assembly | 0,0235 | 1,6296 | 3,7761 | O95831, O75306, P28331, O75489, Q15070, O43920, O95299, Q9BU61, Q9P0J0, O75251, O96000 | 11 | |
| GO:0015986~proton motive force-driven ATP synthesis | 0,0280 | 1,5530 | 6,1923 | O75947, P24539, P48047, P56134, P56385, P36542, Q5VTU8 | 7 | |
| GO:0051664~nuclear pore localization | 0,0289 | 1,5389 | 18,4000 | P20700, P02545, P51114, Q03252 | 4 | |
| GO:0075525~viral translational termination-reinitiation | 0,0289 | 1,5389 | 18,4000 | O15371, P55884, O75821, Q9Y262 | 4 | |
| GO:0000244~spliceosomal tri-snRNP complex assembly | 0,0327 | 1,4858 | 5,9630 | O43395, Q9UMS4, Q8WXF0, Q6P2Q9, Q15020, Q9UHI6, O94906 | 7 | |
| GO:0018149~peptide cross-linking | 0,0327 | 1,4858 | 5,9630 | P20930, P22735, P01040, P23490, P04083, P07476, Q08188 | 7 | |
| GO:0071028~nuclear mRNA surveillance | 0,0327 | 1,4858 | 10,4545 | Q01780, Q9NQT4, Q06265, Q96B26, Q15024 | 5 | |
| GO:0034475~U4 snRNA 3'-end processing | 0,0327 | 1,4858 | 10,4545 | Q13868, Q9NQT4, Q06265, Q96B26, Q15024 | 5 | |
| GO:0021762~substantia nigra development | 0,0344 | 1,4633 | 4,3125 | P11021, P24539, O75489, P00338, O15027, P09543, P00367, P12277, P62258 | 9 | |
| GO:0006417~regulation of translation | 0,0344 | 1,4633 | 3,0825 | Q96EY7, Q92616, Q13428, Q8TB72, Q14978, P61221, P46781, Q9UPQ9, P27635, Q9Y5S9, P61326, P06730 | 13 | |
| GO:0034605~cellular response to heat | 0,0354 | 1,4512 | 3,8333 | Q9UNE7, Q92769, P51617, P17066, P29692, O75340, P55072, P25685, P62258, P08238 | 10 | |
| GO:0006414~translational elongation | 0,0354 | 1,4512 | 7,2632 | P13639, P49411, P29692, P24534, O00178, P05386 | 6 | |
| GO:0007076~mitotic chromosome condensation | 0,0354 | 1,4512 | 7,2632 | O95347, Q9NTJ3, O43823, Q15003, Q9ULX6 | 6 | |
| GO:0006096~glycolytic process | 0,0372 | 1,4296 | 4,2245 | P06733, P18669, P14618, P17858, P00338, P09972, O60825, P08237, P52789 | 9 | |
| GO:0042273~ribosomal large subunit biogenesis | 0,0418 | 1,3792 | 4,7179 | P46087, Q9BZE4, Q9Y3C1, Q15050, O75683, P46777, Q9NWT1, Q9UKD2 | 8 | |
| GO:0017148~negative regulation of translation | 0,0418 | 1,3792 | 2,9900 | O00425, Q14444, Q9NZI8, P26196, O00571, Q8ND56, O60506, P51114, A5YKK6, P68400, P27797, Q92600, P06730 | 13 | |
| GO:0098761~cellular response to interleukin-7 | 0,0418 | 1,3792 | 9,5833 | P46109, P54727, P30101, P10809, P07237 | 5 | |
| GO:0045333~cellular respiration | 0,0472 | 1,3259 | 4,6000 | P13073, P14927, P28331, P00403, P14406, Q9UJS0, P22695 | 8 | |

**Table C. Biological process organization of mass spectrometry data.** Gene Ontology (GO) enrichment analysis of TRIM34 interactome proteins using DAVID. Shown are enriched GO biological processes in mock and IAV samples with FDR < 0.05, including FDR, -log10(FDR), fold enrichment, genes, and counts, ordered by increasing FDR (most to least significant).

| **ACCESSION** | **DESCRIPTION** | **MW [KDa]** | **calc. pI** | **SAMPLE** | **Protein Group IDs** | **Score Mascot: Mascot** | **# PSMs** | **# Peptides** | **# Unique Peptides** | **Coverage [%]** |
| --- | --- | --- | --- | --- | --- | --- | --- | --- | --- | --- |
| P32455 | **Guanylate-binding protein 1 OS=Homo sapiens OX=9606 GN=GBP1 PE=1 SV=2** | 67,9 | 6,32 | MOCK-infected cells | 772 | 3200 | 437 | 53 | 38 | 78 |
|  |  |  |  | IAV-infected cells | 637 | 4275 | 475 | 69 | 52 | 87 |

**Table D. Mass spectrometry identification of GBP1 protein.** Human 293T cells were transfected with the pCAGGS plasmid encoding GBP1-FLAG or the empty plasmid, as control. At 24hpt, cells were mock-infected o infected with IAV (MOI 1). Mass spectrometry identification of protein GBP1 after FLAG pull-down. Protein accession, description, molecular weight, isoelectric point, sample, group ID, Mascot score, PSMs, peptides, unique peptides, and sequence coverage are shown.

| **MOCK** | | | | | | | | | | | | | | | | | | | | | | | | | |  |  |  |
| --- | --- | --- | --- | --- | --- | --- | --- | --- | --- | --- | --- | --- | --- | --- | --- | --- | --- | --- | --- | --- | --- | --- | --- | --- | --- | --- | --- | --- |
| **ACCESSION** | | **DESCRIPTION** | | **MW [kDa]** | **calc. pI** | | **Protein Group IDs** | | | **Score Mascot: Mascot** | | | **#PSMs (by Search Engine): Mascot** | | | **#Peptides (by Search Engine): Mascot** | | | **#PSMs** | | | | | **#Peptides** | |  |  |  |
| P32455 | | **Guanylate-binding protein 1** OS=Homo sapiens OX=9606 GN=GBP1 PE=1 SV=2 | | 67,9 | 6,32 | | 772 | | | 3200 | | | 437 | | | 53 | | | 38 | | | | | 78 | |  |  |  |
| P08238 | | Heat shock protein HSP 90-beta OS=Homo sapiens OX=9606 GN=HSP90AB1 PE=1 SV=4 | | 83,2 | 5,03 | | 295 | | | 1272 | | | 30 | | | 23 | | | 11 | | | | | 38 | |  |  |  |
| P68363 | | Tubulin alpha-1B chain OS=Homo sapiens OX=9606 GN=TUBA1B PE=1 SV=1 | | 50,1 | 5,06 | | 612 | | | 998 | | | 26 | | | 16 | | | 16 | | | | | 48 | |  |  |  |
| Q92841 | | Probable ATP-dependent RNA helicase DDX17 OS=Homo sapiens OX=9606 GN=DDX17 PE=1 SV=2 | | 80,2 | 8,27 | | 503 | | | 751 | | | 18 | | | 14 | | | 11 | | | | | 25 | |  |  |  |
| P07437 | | Tubulin beta chain OS=Homo sapiens OX=9606 GN=TUBB PE=1 SV=2 | | 49,6 | 4,89 | | 709 | | | 738 | | | 24 | | | 14 | | | 3 | | | | | 39 | |  |  |  |
| P68371 | | Tubulin beta-4B chain OS=Homo sapiens OX=9606 GN=TUBB4B PE=1 SV=1 | | 49,8 | 4,89 | | 720 | | | 696 | | | 22 | | | 14 | | | 3 | | | | | 39 | |  |  |  |
| Q9H0R5 | | Guanylate-binding protein 3 OS=Homo sapiens OX=9606 GN=GBP3 PE=1 SV=3 | | 68,1 | 6,51 | | 481 | | | 691 | | | 68 | | | 14 | | | 1 | | | | | 22 | |  |  |  |
| P50990 | | T-complex protein 1 subunit theta OS=Homo sapiens OX=9606 GN=CCT8 PE=1 SV=4 | | 59,6 | 5,6 | | 356 | | | 629 | | | 13 | | | 12 | | | 12 | | | | | 25 | |  |  |  |
| P13797 | | Plastin-3 OS=Homo sapiens OX=9606 GN=PLS3 PE=1 SV=4 | | 70,8 | 5,6 | | 666 | | | 589 | | | 16 | | | 12 | | | 12 | | | | | 21 | |  |  |  |
| P13646 | | Keratin, type I cytoskeletal 13 OS=Homo sapiens OX=9606 GN=KRT13 PE=1 SV=4 | | 49,6 | 4,96 | | 661 | | | 587 | | | 23 | | | 12 | | | 5 | | | | | 22 | |  |  |  |
| P05023 | | Sodium/potassium-transporting ATPase subunit alpha-1 OS=Homo sapiens OX=9606 GN=ATP1A1 PE=1 SV=1 | | 112,8 | 5,49 | | 302 | | | 535 | | | 12 | | | 11 | | | 11 | | | | | 14 | |  |  |  |
| P63267 | | Actin, gamma-enteric smooth muscle OS=Homo sapiens OX=9606 GN=ACTG2 PE=1 SV=1 | | 41,9 | 5,48 | | 124 | | | 525 | | | 18 | | | 10 | | | 1 | | | | | 27 | |  |  |  |
| P25705 | | ATP synthase F(1) complex subunit alpha, mitochondrial OS=Homo sapiens OX=9606 GN=ATP5F1A PE=1 SV=1 | | 59,7 | 9,13 | | 520 | | | 516 | | | 11 | | | 10 | | | 10 | | | | | 20 | |  |  |  |
| P49327 | | Fatty acid synthase OS=Homo sapiens OX=9606 GN=FASN PE=1 SV=3 | | 273,3 | 6,44 | | 135 | | | 482 | | | 12 | | | 12 | | | 12 | | | | | 6 | |  |  |  |
| P06576 | | ATP synthase F(1) complex subunit beta, mitochondrial OS=Homo sapiens OX=9606 GN=ATP5F1B PE=1 SV=3 | | 56,5 | 5,4 | | 521 | | | 445 | | | 11 | | | 10 | | | 10 | | | | | 25 | |  |  |  |
| P78371 | | T-complex protein 1 subunit beta OS=Homo sapiens OX=9606 GN=CCT2 PE=1 SV=4 | | 57,5 | 6,46 | | 547 | | | 426 | | | 11 | | | 9 | | | 9 | | | | | 21 | |  |  |  |
| P40227 | | T-complex protein 1 subunit zeta OS=Homo sapiens OX=9606 GN=CCT6A PE=1 SV=3 | | 58 | 6,68 | | 560 | | | 419 | | | 10 | | | 9 | | | 9 | | | | | 15 | |  |  |  |
| O60506 | | Heterogeneous nuclear ribonucleoprotein Q OS=Homo sapiens OX=9606 GN=SYNCRIP PE=1 SV=2 | | 69,6 | 8,59 | | 385 | | | 410 | | | 13 | | | 11 | | | 8 | | | | | 21 | |  |  |  |
| P10412 | | Histone H1.4 OS=Homo sapiens OX=9606 GN=H1-4 PE=1 SV=2 | | 21,9 | 11,03 | | 544 | | | 389 | | | 8 | | | 7 | | | 2 | | | | | 27 | |  |  |  |
| O43390 | | Heterogeneous nuclear ribonucleoprotein R OS=Homo sapiens OX=9606 GN=HNRNPR PE=1 SV=1 | | 70,9 | 8,13 | | 192 | | | 356 | | | 10 | | | 10 | | | 7 | | | | | 19 | |  |  |  |
| P11940 | | Polyadenylate-binding protein 1 OS=Homo sapiens OX=9606 GN=PABPC1 PE=1 SV=2 | | 70,6 | 9,5 | | 734 | | | 341 | | | 11 | | | 10 | | | 8 | | | | | 17 | |  |  |  |
| P23526 | | Adenosylhomocysteinase OS=Homo sapiens OX=9606 GN=AHCY PE=1 SV=4 | | 47,7 | 6,34 | | 618 | | | 333 | | | 7 | | | 7 | | | 7 | | | | | 18 | |  |  |  |
| P12235 | | ADP/ATP translocase 1 OS=Homo sapiens OX=9606 GN=SLC25A4 PE=1 SV=4 | | 33 | 9,76 | | 415 | | | 328 | | | 8 | | | 7 | | | 2 | | | | | 22 | |  |  |  |
| Q02880 | | DNA topoisomerase 2-beta OS=Homo sapiens OX=9606 GN=TOP2B PE=1 SV=3 | | 183,2 | 8 | | 109 | | | 326 | | | 10 | | | 10 | | | 3 | | | | | 7 | |  |  |  |
| P42167 | | Lamina-associated polypeptide 2, isoforms beta/gamma OS=Homo sapiens OX=9606 GN=TMPO PE=1 SV=2 | | 50,6 | 9,38 | | 429 | | | 324 | | | 5 | | | 5 | | | 5 | | | | | 16 | |  |  |  |
| P27708 | | Multifunctional protein CAD OS=Homo sapiens OX=9606 GN=CAD PE=1 SV=3 | | 242,8 | 6,46 | | 154 | | | 322 | | | 9 | | | 9 | | | 9 | | | | | 4 | |  |  |  |
| P22314 | | Ubiquitin-like modifier-activating enzyme 1 OS=Homo sapiens OX=9606 GN=UBA1 PE=1 SV=3 | | 117,8 | 5,76 | | 523 | | | 312 | | | 6 | | | 6 | | | 6 | | | | | 7 | |  |  |  |
| P32456 | | Guanylate-binding protein 2 OS=Homo sapiens OX=9606 GN=GBP2 PE=1 SV=3 | | 67,2 | 5,71 | | 749 | | | 311 | | | 34 | | | 6 | | | 1 | | | | | 10 | |  |  |  |
| P17987 | | T-complex protein 1 subunit alpha OS=Homo sapiens OX=9606 GN=TCP1 PE=1 SV=1 | | 60,3 | 6,11 | | 240 | | | 307 | | | 10 | | | 9 | | | 9 | | | | | 19 | |  |  |  |
| P16402 | | Histone H1.3 OS=Homo sapiens OX=9606 GN=H1-3 PE=1 SV=2 | | 22,3 | 11,02 | | 525 | | | 299 | | | 7 | | | 6 | | | 1 | | | | | 26 | |  |  |  |
| P30050 | | Large ribosomal subunit protein uL11 OS=Homo sapiens OX=9606 GN=RPL12 PE=1 SV=1 | | 17,8 | 9,42 | | 50 | | | 296 | | | 12 | | | 5 | | | 5 | | | | | 41 | |  |  |  |
| Q58FF6 | | Putative heat shock protein HSP 90-beta 4 OS=Homo sapiens OX=9606 GN=HSP90AB4P PE=5 SV=1 | | 58,2 | 4,73 | | 408 | | | 289 | | | 6 | | | 5 | | | 1 | | | | | 9 | |  |  |  |
| P13010 | | X-ray repair cross-complementing protein 5 OS=Homo sapiens OX=9606 GN=XRCC5 PE=1 SV=3 | | 82,7 | 5,81 | | 38 | | | 287 | | | 7 | | | 7 | | | 7 | | | | | 10 | |  |  |  |
| P60842 | | Eukaryotic initiation factor 4A-I OS=Homo sapiens OX=9606 GN=EIF4A1 PE=1 SV=1 | | 46,1 | 5,48 | | 331 | | | 278 | | | 7 | | | 7 | | | 6 | | | | | 21 | |  |  |  |
| Q04637 | | Eukaryotic translation initiation factor 4 gamma 1 OS=Homo sapiens OX=9606 GN=EIF4G1 PE=1 SV=4 | | 175,4 | 5,33 | | 166 | | | 269 | | | 8 | | | 8 | | | 8 | | | | | 7 | |  |  |  |
| Q92598 | | Heat shock protein 105 kDa OS=Homo sapiens OX=9606 GN=HSPH1 PE=1 SV=1 | | 96,8 | 5,39 | | 690 | | | 264 | | | 6 | | | 6 | | | 6 | | | | | 8 | |  |  |  |
| Q16576 | | Histone-binding protein RBBP7 OS=Homo sapiens OX=9606 GN=RBBP7 PE=1 SV=1 | | 47,8 | 5,05 | | 60 | | | 259 | | | 5 | | | 5 | | | 5 | | | | | 11 | |  |  |  |
| P32969 | | Large ribosomal subunit protein uL6 OS=Homo sapiens OX=9606 GN=RPL9 PE=1 SV=1 | | 21,9 | 9,95 | | 379 | | | 258 | | | 9 | | | 5 | | | 5 | | | | | 37 | |  |  |  |
| P35637 | | RNA-binding protein FUS OS=Homo sapiens OX=9606 GN=FUS PE=1 SV=1 | | 53,4 | 9,36 | | 416 | | | 257 | | | 5 | | | 5 | | | 5 | | | | | 12 | |  |  |  |
| Q5QNW6 | | Histone H2B type 2-F OS=Homo sapiens OX=9606 GN=H2BC18 PE=1 SV=3 | | 13,9 | 10,32 | | 139 | | | 255 | | | 5 | | | 5 | | | 1 | | | | | 41 | |  |  |  |
| Q16778 | | Histone H2B type 2-E OS=Homo sapiens OX=9606 GN=H2BC21 PE=1 SV=3 | | 13,9 | 10,32 | | 83 | | | 246 | | | 5 | | | 5 | | | 1 | | | | | 41 | |  |  |  |
| P36578 | | Large ribosomal subunit protein uL4 OS=Homo sapiens OX=9606 GN=RPL4 PE=1 SV=5 | | 47,7 | 11,06 | | 239 | | | 244 | | | 7 | | | 7 | | | 7 | | | | | 19 | |  |  |  |
| P55795 | | Heterogeneous nuclear ribonucleoprotein H2 OS=Homo sapiens OX=9606 GN=HNRNPH2 PE=1 SV=1 | | 49,2 | 6,3 | | 739 | | | 243 | | | 5 | | | 5 | | | 1 | | | | | 13 | |  |  |  |
| Q86VP6 | | Cullin-associated NEDD8-dissociated protein 1 OS=Homo sapiens OX=9606 GN=CAND1 PE=1 SV=2 | | 136,3 | 5,78 | | 35 | | | 241 | | | 7 | | | 7 | | | 7 | | | | | 6 | |  |  |  |
| P49368 | | T-complex protein 1 subunit gamma OS=Homo sapiens OX=9606 GN=CCT3 PE=1 SV=4 | | 60,5 | 6,49 | | 730 | | | 240 | | | 7 | | | 7 | | | 7 | | | | | 14 | |  |  |  |
| O43809 | | Cleavage and polyadenylation specificity factor subunit 5 OS=Homo sapiens OX=9606 GN=NUDT21 PE=1 SV=1 | | 26,2 | 8,82 | | 668 | | | 235 | | | 4 | | | 4 | | | 4 | | | | | 15 | |  |  |  |
| Q92945 | | Far upstream element-binding protein 2 OS=Homo sapiens OX=9606 GN=KHSRP PE=1 SV=4 | | 73,1 | 7,3 | | 426 | | | 228 | | | 8 | | | 8 | | | 8 | | | | | 14 | |  |  |  |
| P30041 | | Peroxiredoxin-6 OS=Homo sapiens OX=9606 GN=PRDX6 PE=1 SV=3 | | 25 | 6,38 | | 264 | | | 219 | | | 6 | | | 5 | | | 5 | | | | | 18 | |  |  |  |
| Q15366 | | Poly(rC)-binding protein 2 OS=Homo sapiens OX=9606 GN=PCBP2 PE=1 SV=1 | | 38,6 | 6,79 | | 370 | | | 219 | | | 6 | | | 5 | | | 2 | | | | | 16 | |  |  |  |
| Q15084 | | Protein disulfide-isomerase A6 OS=Homo sapiens OX=9606 GN=PDIA6 PE=1 SV=1 | | 48,1 | 5,08 | | 686 | | | 217 | | | 3 | | | 3 | | | 3 | | | | | 9 | |  |  |  |
| Q14008 | | Cytoskeleton-associated protein 5 OS=Homo sapiens OX=9606 GN=CKAP5 PE=1 SV=3 | | 225,4 | 7,8 | | 404 | | | 217 | | | 5 | | | 5 | | | 5 | | | | | 3 | |  |  |  |
| Q8WXF1 | | Paraspeckle component 1 OS=Homo sapiens OX=9606 GN=PSPC1 PE=1 SV=1 | | 58,7 | 6,67 | | 246 | | | 215 | | | 5 | | | 5 | | | 4 | | | | | 11 | |  |  |  |
| P41252 | | Isoleucine--tRNA ligase, cytoplasmic OS=Homo sapiens OX=9606 GN=IARS1 PE=1 SV=2 | | 144,4 | 6,15 | | 632 | | | 209 | | | 6 | | | 6 | | | 6 | | | | | 6 | |  |  |  |
| P62805 | | Histone H4 OS=Homo sapiens OX=9606 GN=H4C1 PE=1 SV=2 | | 11,4 | 11,36 | | 477 | | | 208 | | | 7 | | | 4 | | | 4 | | | | | 41 | |  |  |  |
| P46977 | | Dolichyl-diphosphooligosaccharide--protein glycosyltransferase subunit STT3A OS=Homo sapiens OX=9606 GN=STT3A PE=1 SV=2 | | 80,5 | 8,07 | | 16 | | | 208 | | | 5 | | | 5 | | | 5 | | | | | 6 | |  |  |  |
| P15880 | | Small ribosomal subunit protein uS5 OS=Homo sapiens OX=9606 GN=RPS2 PE=1 SV=2 | | 31,3 | 10,24 | | 588 | | | 206 | | | 6 | | | 5 | | | 5 | | | | | 17 | |  |  |  |
| Q9NR30 | | Nucleolar RNA helicase 2 OS=Homo sapiens OX=9606 GN=DDX21 PE=1 SV=5 | | 87,3 | 9,28 | | 70 | | | 200 | | | 6 | | | 6 | | | 6 | | | | | 10 | |  |  |  |
| Q15365 | | Poly(rC)-binding protein 1 OS=Homo sapiens OX=9606 GN=PCBP1 PE=1 SV=2 | | 37,5 | 7,09 | | 423 | | | 199 | | | 6 | | | 5 | | | 2 | | | | | 17 | |  |  |  |
| Q9BUF5 | | Tubulin beta-6 chain OS=Homo sapiens OX=9606 GN=TUBB6 PE=1 SV=1 | | 49,8 | 4,88 | | 338 | | | 195 | | | 6 | | | 5 | | | 1 | | | | | 13 | |  |  |  |
| P48594 | | Serpin B4 OS=Homo sapiens OX=9606 GN=SERPINB4 PE=1 SV=2 | | 44,8 | 6,21 | | 47 | | | 188 | | | 4 | | | 4 | | | 4 | | | | | 9 | |  |  |  |
| P14678 | | Small nuclear ribonucleoprotein-associated proteins B and B' OS=Homo sapiens OX=9606 GN=SNRPB PE=1 SV=2 | | 24,6 | 11,19 | | 489 | | | 188 | | | 3 | | | 3 | | | 3 | | | | | 13 | |  |  |  |
| Q92522 | | Histone H1.10 OS=Homo sapiens OX=9606 GN=H1-10 PE=1 SV=1 | | 22,5 | 10,76 | | 146 | | | 187 | | | 5 | | | 5 | | | 5 | | | | | 26 | |  |  |  |
| P62937 | | Peptidyl-prolyl cis-trans isomerase A OS=Homo sapiens OX=9606 GN=PPIA PE=1 SV=2 | | 18 | 7,81 | | 515 | | | 186 | | | 5 | | | 4 | | | 4 | | | | | 26 | |  |  |  |
| Q00325 | | Solute carrier family 25 member 3 OS=Homo sapiens OX=9606 GN=SLC25A3 PE=1 SV=2 | | 40,1 | 9,38 | | 799 | | | 185 | | | 6 | | | 5 | | | 5 | | | | | 14 | |  |  |  |
| P48047 | | ATP synthase peripheral stalk subunit OSCP, mitochondrial OS=Homo sapiens OX=9606 GN=ATP5PO PE=1 SV=1 | | 23,3 | 9,96 | | 626 | | | 184 | | | 4 | | | 3 | | | 3 | | | | | 16 | |  |  |  |
| P04181 | | Ornithine aminotransferase, mitochondrial OS=Homo sapiens OX=9606 GN=OAT PE=1 SV=1 | | 48,5 | 7,03 | | 798 | | | 184 | | | 3 | | | 3 | | | 3 | | | | | 7 | |  |  |  |
| P24534 | | Elongation factor 1-beta OS=Homo sapiens OX=9606 GN=EEF1B2 PE=1 SV=3 | | 24,7 | 4,67 | | 480 | | | 177 | | | 4 | | | 3 | | | 3 | | | | | 16 | |  |  |  |
| Q8NCA5 | | Protein FAM98A OS=Homo sapiens OX=9606 GN=FAM98A PE=1 SV=2 | | 55,2 | 8,95 | | 808 | | | 176 | | | 4 | | | 3 | | | 3 | | | | | 11 | |  |  |  |
| Q9UJS0 | | Electrogenic aspartate/glutamate antiporter SLC25A13, mitochondrial OS=Homo sapiens OX=9606 GN=SLC25A13 PE=1 SV=2 | | 74,1 | 8,62 | | 555 | | | 175 | | | 4 | | | 4 | | | 4 | | | | | 7 | |  |  |  |
| Q14204 | | Cytoplasmic dynein 1 heavy chain 1 OS=Homo sapiens OX=9606 GN=DYNC1H1 PE=1 SV=5 | | 532,1 | 6,4 | | 291 | | | 171 | | | 6 | | | 6 | | | 6 | | | | | 2 | |  |  |  |
| O00303 | | Eukaryotic translation initiation factor 3 subunit F OS=Homo sapiens OX=9606 GN=EIF3F PE=1 SV=1 | | 37,5 | 5,45 | | 748 | | | 170 | | | 4 | | | 4 | | | 4 | | | | | 15 | |  |  |  |
| Q13162 | | Peroxiredoxin-4 OS=Homo sapiens OX=9606 GN=PRDX4 PE=1 SV=1 | | 30,5 | 6,29 | | 508 | | | 170 | | | 5 | | | 4 | | | 2 | | | | | 15 | |  |  |  |
| Q96C36 | | Pyrroline-5-carboxylate reductase 2 OS=Homo sapiens OX=9606 GN=PYCR2 PE=1 SV=1 | | 33,6 | 7,77 | | 54 | | | 165 | | | 4 | | | 4 | | | 4 | | | | | 15 | |  |  |  |
| P11387 | | DNA topoisomerase 1 OS=Homo sapiens OX=9606 GN=TOP1 PE=1 SV=2 | | 90,7 | 9,31 | | 820 | | | 165 | | | 6 | | | 6 | | | 6 | | | | | 8 | |  |  |  |
| P36957 | | Dihydrolipoyllysine-residue succinyltransferase component of 2-oxoglutarate dehydrogenase complex, mitochondrial OS=Homo sapiens OX=9606 GN=DLST PE=1 SV=4 | | 48,7 | 8,95 | | 161 | | | 164 | | | 4 | | | 4 | | | 4 | | | | | 13 | |  |  |  |
| P52597 | | Heterogeneous nuclear ribonucleoprotein F OS=Homo sapiens OX=9606 GN=HNRNPF PE=1 SV=3 | | 45,6 | 5,58 | | 514 | | | 164 | | | 5 | | | 5 | | | 3 | | | | | 13 | |  |  |  |
| P04844 | | Dolichyl-diphosphooligosaccharide--protein glycosyltransferase subunit 2 OS=Homo sapiens OX=9606 GN=RPN2 PE=1 SV=3 | | 69,2 | 5,69 | | 277 | | | 164 | | | 5 | | | 5 | | | 5 | | | | | 11 | |  |  |  |
| P62241 | | Small ribosomal subunit protein eS8 OS=Homo sapiens OX=9606 GN=RPS8 PE=1 SV=2 | | 24,2 | 10,32 | | 220 | | | 162 | | | 4 | | | 3 | | | 3 | | | | | 17 | |  |  |  |
| P22392 | | Nucleoside diphosphate kinase B OS=Homo sapiens OX=9606 GN=NME2 PE=1 SV=1 | | 17,3 | 8,41 | | 735 | | | 162 | | | 4 | | | 4 | | | 4 | | | | | 31 | |  |  |  |
| P12277 | | Creatine kinase B-type OS=Homo sapiens OX=9606 GN=CKB PE=1 SV=1 | | 42,6 | 5,59 | | 487 | | | 160 | | | 3 | | | 3 | | | 3 | | | | | 9 | |  |  |  |
| P62993 | | Growth factor receptor-bound protein 2 OS=Homo sapiens OX=9606 GN=GRB2 PE=1 SV=1 | | 25,2 | 6,32 | | 581 | | | 157 | | | 4 | | | 4 | | | 4 | | | | | 20 | |  |  |  |
| P22087 | | rRNA 2'-O-methyltransferase fibrillarin OS=Homo sapiens OX=9606 GN=FBL PE=1 SV=2 | | 33,8 | 10,18 | | 795 | | | 157 | | | 4 | | | 4 | | | 4 | | | | | 12 | |  |  |  |
| P62829 | | Large ribosomal subunit protein uL14 OS=Homo sapiens OX=9606 GN=RPL23 PE=1 SV=1 | | 14,9 | 10,51 | | 273 | | | 156 | | | 7 | | | 3 | | | 3 | | | | | 24 | |  |  |  |
| Q15717 | | ELAV-like protein 1 OS=Homo sapiens OX=9606 GN=ELAVL1 PE=1 SV=2 | | 36,1 | 9,17 | | 803 | | | 148 | | | 5 | | | 5 | | | 5 | | | | | 15 | |  |  |  |
| Q16777 | | Histone H2A type 2-C OS=Homo sapiens OX=9606 GN=H2AC20 PE=1 SV=4 | | 14 | 10,9 | | 10 | | | 147 | | | 5 | | | 4 | | | 2 | | | | | 33 | |  |  |  |
| Q04760 | | Lactoylglutathione lyase OS=Homo sapiens OX=9606 GN=GLO1 PE=1 SV=4 | | 20,8 | 5,31 | | 195 | | | 147 | | | 5 | | | 5 | | | 5 | | | | | 22 | |  |  |  |
| Q07021 | | Complement component 1 Q subcomponent-binding protein, mitochondrial OS=Homo sapiens OX=9606 GN=C1QBP PE=1 SV=1 | | 31,3 | 4,84 | | 494 | | | 146 | | | 3 | | | 3 | | | 3 | | | | | 17 | |  |  |  |
| P12532 | | Creatine kinase U-type, mitochondrial OS=Homo sapiens OX=9606 GN=CKMT1A PE=1 SV=1 | | 47 | 8,34 | | 640 | | | 146 | | | 3 | | | 2 | | | 2 | | | | | 7 | |  |  |  |
| Q9UMS4 | | Pre-mRNA-processing factor 19 OS=Homo sapiens OX=9606 GN=PRPF19 PE=1 SV=1 | | 55,1 | 6,61 | | 299 | | | 144 | | | 4 | | | 3 | | | 3 | | | | | 7 | |  |  |  |
| P62266 | | Small ribosomal subunit protein uS12 OS=Homo sapiens OX=9606 GN=RPS23 PE=1 SV=3 | | 15,8 | 10,49 | | 448 | | | 143 | | | 3 | | | 2 | | | 2 | | | | | 15 | |  |  |  |
| P11586 | | C-1-tetrahydrofolate synthase, cytoplasmic OS=Homo sapiens OX=9606 GN=MTHFD1 PE=1 SV=4 | | 101,5 | 7,3 | | 590 | | | 142 | | | 4 | | | 4 | | | 4 | | | | | 6 | |  |  |  |
| P25398 | | Small ribosomal subunit protein eS12 OS=Homo sapiens OX=9606 GN=RPS12 PE=1 SV=3 | | 14,5 | 7,21 | | 689 | | | 140 | | | 3 | | | 3 | | | 3 | | | | | 31 | |  |  |  |
| P18085 | | ADP-ribosylation factor 4 OS=Homo sapiens OX=9606 GN=ARF4 PE=1 SV=3 | | 20,5 | 7,14 | | 211 | | | 137 | | | 3 | | | 3 | | | 2 | | | | | 19 | |  |  |  |
| Q9P035 | | Very-long-chain (3R)-3-hydroxyacyl-CoA dehydratase 3 OS=Homo sapiens OX=9606 GN=HACD3 PE=1 SV=2 | | 43,1 | 8,94 | | 52 | | | 135 | | | 2 | | | 2 | | | 2 | | | | | 7 | |  |  |  |
| Q49A26 | | Cytokine-like nuclear factor N-PAC OS=Homo sapiens OX=9606 GN=GLYR1 PE=1 SV=4 | | 60,5 | 9,17 | | 662 | | | 131 | | | 2 | | | 2 | | | 2 | | | | | 5 | |  |  |  |
| P46940 | | Ras GTPase-activating-like protein IQGAP1 OS=Homo sapiens OX=9606 GN=IQGAP1 PE=1 SV=1 | | 189,1 | 6,48 | | 805 | | | 129 | | | 3 | | | 3 | | | 3 | | | | | 2 | |  |  |  |
| P41091 | | Eukaryotic translation initiation factor 2 subunit 3 OS=Homo sapiens OX=9606 GN=EIF2S3 PE=1 SV=3 | | 51,1 | 8,4 | | 454 | | | 129 | | | 4 | | | 4 | | | 4 | | | | | 10 | |  |  |  |
| P53999 | | Activated RNA polymerase II transcriptional coactivator p15 OS=Homo sapiens OX=9606 GN=SUB1 PE=1 SV=3 | | 14,4 | 9,6 | | 399 | | | 128 | | | 3 | | | 3 | | | 3 | | | | | 27 | |  |  |  |
| Q9P2J5 | | Leucine--tRNA ligase, cytoplasmic OS=Homo sapiens OX=9606 GN=LARS1 PE=1 SV=2 | | 134,4 | 7,3 | | 276 | | | 127 | | | 4 | | | 4 | | | 4 | | | | | 5 | |  |  |  |
| P08708 | | Small ribosomal subunit protein eS17 OS=Homo sapiens OX=9606 GN=RPS17 PE=1 SV=2 | | 15,5 | 9,85 | | 758 | | | 125 | | | 7 | | | 4 | | | 4 | | | | | 48 | |  |  |  |
| P00492 | | Hypoxanthine-guanine phosphoribosyltransferase OS=Homo sapiens OX=9606 GN=HPRT1 PE=1 SV=2 | | 24,6 | 6,68 | | 401 | | | 125 | | | 3 | | | 2 | | | 2 | | | | | 11 | |  |  |  |
| Q14498 | | RNA-binding protein 39 OS=Homo sapiens OX=9606 GN=RBM39 PE=1 SV=2 | | 59,3 | 10,1 | | 562 | | | 124 | | | 4 | | | 3 | | | 3 | | | | | 8 | |  |  |  |
| Q13310 | | Polyadenylate-binding protein 4 OS=Homo sapiens OX=9606 GN=PABPC4 PE=1 SV=1 | | 70,7 | 9,26 | | 301 | | | 122 | | | 4 | | | 4 | | | 2 | | | | | 7 | |  |  |  |
| P55084 | | Trifunctional enzyme subunit beta, mitochondrial OS=Homo sapiens OX=9606 GN=HADHB PE=1 SV=3 | | 51,3 | 9,41 | | 601 | | | 122 | | | 4 | | | 4 | | | 4 | | | | | 8 | |  |  |  |
| P55265 | | Double-stranded RNA-specific adenosine deaminase OS=Homo sapiens OX=9606 GN=ADAR PE=1 SV=4 | | 136 | 8,65 | | 609 | | | 121 | | | 4 | | | 4 | | | 4 | | | | | 4 | |  |  |  |
| P29692 | | Elongation factor 1-delta OS=Homo sapiens OX=9606 GN=EEF1D PE=1 SV=5 | | 31,1 | 5,01 | | 21 | | | 119 | | | 3 | | | 3 | | | 3 | | | | | 12 | |  |  |  |
| Q8N684 | | Cleavage and polyadenylation specificity factor subunit 7 OS=Homo sapiens OX=9606 GN=CPSF7 PE=1 SV=1 | | 52 | 8 | | 184 | | | 119 | | | 3 | | | 3 | | | 3 | | | | | 8 | |  |  |  |
| P26196 | | Probable ATP-dependent RNA helicase DDX6 OS=Homo sapiens OX=9606 GN=DDX6 PE=1 SV=2 | | 54,4 | 8,66 | | 39 | | | 119 | | | 5 | | | 3 | | | 3 | | | | | 10 | |  |  |  |
| Q13185 | | Chromobox protein homolog 3 OS=Homo sapiens OX=9606 GN=CBX3 PE=1 SV=4 | | 20,8 | 5,33 | | 2 | | | 119 | | | 4 | | | 3 | | | 3 | | | | | 17 | |  |  |  |
| Q9NY12 | | H/ACA ribonucleoprotein complex subunit 1 OS=Homo sapiens OX=9606 GN=GAR1 PE=1 SV=1 | | 22,3 | 10,92 | | 23 | | | 118 | | | 3 | | | 3 | | | 3 | | | | | 12 | |  |  |  |
| Q99497 | | Parkinson disease protein 7 OS=Homo sapiens OX=9606 GN=PARK7 PE=1 SV=2 | | 19,9 | 6,79 | | 797 | | | 118 | | | 2 | | | 2 | | | 2 | | | | | 12 | |  |  |  |
| P46776 | | Large ribosomal subunit protein uL15 OS=Homo sapiens OX=9606 GN=RPL27A PE=1 SV=2 | | 16,6 | 11 | | 809 | | | 116 | | | 2 | | | 2 | | | 2 | | | | | 14 | |  |  |  |
| Q16531 | | DNA damage-binding protein 1 OS=Homo sapiens OX=9606 GN=DDB1 PE=1 SV=1 | | 126,9 | 5,26 | | 104 | | | 115 | | | 4 | | | 4 | | | 4 | | | | | 5 | |  |  |  |
| Q9Y2X3 | | Nucleolar protein 58 OS=Homo sapiens OX=9606 GN=NOP58 PE=1 SV=1 | | 59,5 | 8,92 | | 112 | | | 115 | | | 1 | | | 1 | | | 1 | | | | | 3 | |  |  |  |
| Q71UI9 | | Histone H2A.V OS=Homo sapiens OX=9606 GN=H2AZ2 PE=1 SV=3 | | 13,5 | 10,58 | | 24 | | | 114 | | | 3 | | | 3 | | | 1 | | | | | 20 | |  |  |  |
| P43487 | | Ran-specific GTPase-activating protein OS=Homo sapiens OX=9606 GN=RANBP1 PE=1 SV=1 | | 23,3 | 5,29 | | 87 | | | 114 | | | 3 | | | 3 | | | 3 | | | | | 27 | |  |  |  |
| Q15029 | | 116 kDa U5 small nuclear ribonucleoprotein component OS=Homo sapiens OX=9606 GN=EFTUD2 PE=1 SV=1 | | 109,4 | 5 | | 793 | | | 114 | | | 4 | | | 4 | | | 3 | | | | | 6 | |  |  |  |
| P61513 | | Large ribosomal subunit protein eL43 OS=Homo sapiens OX=9606 GN=RPL37A PE=1 SV=2 | | 10,3 | 10,43 | | 145 | | | 113 | | | 2 | | | 1 | | | 1 | | | | | 20 | |  |  |  |
| O00469 | | Procollagen-lysine,2-oxoglutarate 5-dioxygenase 2 OS=Homo sapiens OX=9606 GN=PLOD2 PE=1 SV=2 | | 84,6 | 6,71 | | 538 | | | 113 | | | 3 | | | 3 | | | 3 | | | | | 5 | |  |  |  |
| P35232 | | Prohibitin 1 OS=Homo sapiens OX=9606 GN=PHB1 PE=1 SV=1 | | 29,8 | 5,76 | | 407 | | | 112 | | | 3 | | | 3 | | | 3 | | | | | 12 | |  |  |  |
| P09972 | | Fructose-bisphosphate aldolase C OS=Homo sapiens OX=9606 GN=ALDOC PE=1 SV=2 | | 39,4 | 6,87 | | 185 | | | 111 | | | 3 | | | 2 | | | 1 | | | | | 8 | |  |  |  |
| O60832 | | H/ACA ribonucleoprotein complex subunit DKC1 OS=Homo sapiens OX=9606 GN=DKC1 PE=1 SV=3 | | 57,6 | 9,42 | | 337 | | | 111 | | | 4 | | | 3 | | | 3 | | | | | 5 | |  |  |  |
| Q9H078 | | Mitochondrial disaggregase OS=Homo sapiens OX=9606 GN=CLPB PE=1 SV=1 | | 78,7 | 9,01 | | 148 | | | 111 | | | 2 | | | 2 | | | 2 | | | | | 5 | |  |  |  |
| Q9Y295 | | Developmentally-regulated GTP-binding protein 1 OS=Homo sapiens OX=9606 GN=DRG1 PE=1 SV=1 | | 40,5 | 8,9 | | 278 | | | 111 | | | 2 | | | 2 | | | 2 | | | | | 6 | |  |  |  |
| Q9NXS2 | | Glutaminyl-peptide cyclotransferase-like protein OS=Homo sapiens OX=9606 GN=QPCTL PE=1 SV=2 | | 42,9 | 9,82 | | 28 | | | 110 | | | 2 | | | 2 | | | 2 | | | | | 6 | |  |  |  |
| P56192 | | Methionine--tRNA ligase, cytoplasmic OS=Homo sapiens OX=9606 GN=MARS1 PE=1 SV=2 | | 101,1 | 6,16 | | 106 | | | 110 | | | 2 | | | 2 | | | 2 | | | | | 2 | |  |  |  |
| P25205 | | DNA replication licensing factor MCM3 OS=Homo sapiens OX=9606 GN=MCM3 PE=1 SV=3 | | 90,9 | 5,77 | | 226 | | | 109 | | | 2 | | | 2 | | | 2 | | | | | 3 | |  |  |  |
| Q9NTK5 | | Obg-like ATPase 1 OS=Homo sapiens OX=9606 GN=OLA1 PE=1 SV=2 | | 44,7 | 7,81 | | 122 | | | 108 | | | 2 | | | 2 | | | 2 | | | | | 6 | |  |  |  |
| Q01130 | | Serine/arginine-rich splicing factor 2 OS=Homo sapiens OX=9606 GN=SRSF2 PE=1 SV=4 | | 25,5 | 11,85 | | 814 | | | 108 | | | 3 | | | 3 | | | 3 | | | | | 19 | |  |  |  |
| P06737 | | Glycogen phosphorylase, liver form OS=Homo sapiens OX=9606 GN=PYGL PE=1 SV=4 | | 97,1 | 7,17 | | 660 | | | 108 | | | 4 | | | 4 | | | 4 | | | | | 8 | |  |  |  |
| Q01813 | | ATP-dependent 6-phosphofructokinase, platelet type OS=Homo sapiens OX=9606 GN=PFKP PE=1 SV=2 | | 85,5 | 7,55 | | 304 | | | 104 | | | 4 | | | 4 | | | 2 | | | | | 7 | |  |  |  |
| P62140 | | Serine/threonine-protein phosphatase PP1-beta catalytic subunit OS=Homo sapiens OX=9606 GN=PPP1CB PE=1 SV=3 | | 37,2 | 6,19 | | 625 | | | 102 | | | 3 | | | 2 | | | 1 | | | | | 8 | |  |  |  |
| P35659 | | Protein DEK OS=Homo sapiens OX=9606 GN=DEK PE=1 SV=1 | | 42,6 | 8,56 | | 716 | | | 102 | | | 1 | | | 1 | | | 1 | | | | | 3 | |  |  |  |
| Q92769 | | Histone deacetylase 2 OS=Homo sapiens OX=9606 GN=HDAC2 PE=1 SV=2 | | 55,3 | 5,91 | | 608 | | | 102 | | | 3 | | | 3 | | | 1 | | | | | 8 | |  |  |  |
| Q15517 | | Corneodesmosin OS=Homo sapiens OX=9606 GN=CDSN PE=1 SV=4 | | 51,6 | 8,44 | | 322 | | | 101 | | | 3 | | | 3 | | | 2 | | | | | 6 | |  |  |  |
| P84103 | | Serine/arginine-rich splicing factor 3 OS=Homo sapiens OX=9606 GN=SRSF3 PE=1 SV=1 | | 19,3 | 11,65 | | 646 | | | 100 | | | 4 | | | 3 | | | 3 | | | | | 19 | |  |  |  |
| Q14152 | | Eukaryotic translation initiation factor 3 subunit A OS=Homo sapiens OX=9606 GN=EIF3A PE=1 SV=1 | | 166,5 | 6,79 | | 697 | | | 100 | | | 3 | | | 3 | | | 3 | | | | | 2 | |  |  |  |
| Q12931 | | Heat shock protein 75 kDa, mitochondrial OS=Homo sapiens OX=9606 GN=TRAP1 PE=1 SV=3 | | 80,1 | 8,21 | | 335 | | | 98 | | | 3 | | | 2 | | | 1 | | | | | 4 | |  |  |  |
| P17858 | | ATP-dependent 6-phosphofructokinase, liver type OS=Homo sapiens OX=9606 GN=PFKL PE=1 SV=6 | | 85 | 7,5 | | 492 | | | 95 | | | 3 | | | 3 | | | 1 | | | | | 5 | |  |  |  |
| P25789 | | Proteasome subunit alpha type-4 OS=Homo sapiens OX=9606 GN=PSMA4 PE=1 SV=1 | | 29,5 | 7,72 | | 822 | | | 95 | | | 2 | | | 2 | | | 2 | | | | | 7 | |  |  |  |
| Q53GQ0 | | Very-long-chain 3-oxoacyl-CoA reductase OS=Homo sapiens OX=9606 GN=HSD17B12 PE=1 SV=2 | | 34,3 | 9,32 | | 268 | | | 95 | | | 2 | | | 2 | | | 2 | | | | | 8 | |  |  |  |
| Q6PKG0 | | La-related protein 1 OS=Homo sapiens OX=9606 GN=LARP1 PE=1 SV=2 | | 123,4 | 8,82 | | 420 | | | 94 | | | 4 | | | 3 | | | 3 | | | | | 3 | |  |  |  |
| P43243 | | Matrin-3 OS=Homo sapiens OX=9606 GN=MATR3 PE=1 SV=2 | | 94,6 | 6,25 | | 449 | | | 93 | | | 2 | | | 2 | | | 2 | | | | | 3 | |  |  |  |
| P39656 | | Dolichyl-diphosphooligosaccharide--protein glycosyltransferase 48 kDa subunit OS=Homo sapiens OX=9606 GN=DDOST PE=1 SV=4 | | 50,8 | 6,55 | | 133 | | | 93 | | | 3 | | | 3 | | | 3 | | | | | 7 | |  |  |  |
| Q9P258 | | Protein RCC2 OS=Homo sapiens OX=9606 GN=RCC2 PE=1 SV=2 | | 56 | 8,78 | | 469 | | | 92 | | | 3 | | | 3 | | | 3 | | | | | 5 | |  |  |  |
| P24666 | | Low molecular weight phosphotyrosine protein phosphatase OS=Homo sapiens OX=9606 GN=ACP1 PE=1 SV=3 | | 18 | 6,74 | | 622 | | | 90 | | | 3 | | | 3 | | | 3 | | | | | 20 | |  |  |  |
| O14980 | | Exportin-1 OS=Homo sapiens OX=9606 GN=XPO1 PE=1 SV=1 | | 123,3 | 6,06 | | 364 | | | 90 | | | 3 | | | 3 | | | 3 | | | | | 3 | |  |  |  |
| P46777 | | Large ribosomal subunit protein uL18 OS=Homo sapiens OX=9606 GN=RPL5 PE=1 SV=3 | | 34,3 | 9,72 | | 549 | | | 90 | | | 3 | | | 3 | | | 3 | | | | | 12 | |  |  |  |
| Q3MHD2 | | Protein LSM12 OS=Homo sapiens OX=9606 GN=LSM12 PE=1 SV=2 | | 21,7 | 7,74 | | 392 | | | 89 | | | 2 | | | 2 | | | 2 | | | | | 14 | |  |  |  |
| P84243 | | Histone H3.3 OS=Homo sapiens OX=9606 GN=H3-3A PE=1 SV=2 | | 15,3 | 11,27 | | 32 | | | 89 | | | 2 | | | 2 | | | 2 | | | | | 10 | |  |  |  |
| P16615 | | Sarcoplasmic/endoplasmic reticulum calcium ATPase 2 OS=Homo sapiens OX=9606 GN=ATP2A2 PE=1 SV=1 | | 114,7 | 5,34 | | 466 | | | 89 | | | 4 | | | 4 | | | 4 | | | | | 4 | |  |  |  |
| P22695 | | Cytochrome b-c1 complex subunit 2, mitochondrial OS=Homo sapiens OX=9606 GN=UQCRC2 PE=1 SV=3 | | 48,4 | 8,63 | | 438 | | | 88 | | | 3 | | | 2 | | | 2 | | | | | 6 | |  |  |  |
| Q96AE4 | | Far upstream element-binding protein 1 OS=Homo sapiens OX=9606 GN=FUBP1 PE=1 SV=3 | | 67,5 | 7,61 | | 46 | | | 88 | | | 3 | | | 3 | | | 3 | | | | | 6 | |  |  |  |
| Q9NPD3 | | Exosome complex component RRP41 OS=Homo sapiens OX=9606 GN=EXOSC4 PE=1 SV=3 | | 26,4 | 6,52 | | 199 | | | 87 | | | 2 | | | 2 | | | 2 | | | | | 9 | |  |  |  |
| O14979 | | Heterogeneous nuclear ribonucleoprotein D-like OS=Homo sapiens OX=9606 GN=HNRNPDL PE=1 SV=3 | | 46,4 | 9,57 | | 606 | | | 87 | | | 2 | | | 2 | | | 2 | | | | | 6 | |  |  |  |
| P49458 | | Signal recognition particle 9 kDa protein OS=Homo sapiens OX=9606 GN=SRP9 PE=1 SV=2 | | 10,1 | 7,97 | | 259 | | | 87 | | | 2 | | | 2 | | | 2 | | | | | 22 | |  |  |  |
| Q9Y5M8 | | Signal recognition particle receptor subunit beta OS=Homo sapiens OX=9606 GN=SRPRB PE=1 SV=3 | | 29,7 | 9,04 | | 129 | | | 86 | | | 1 | | | 1 | | | 1 | | | | | 7 | |  |  |  |
| P62854 | | Small ribosomal subunit protein eS26 OS=Homo sapiens OX=9606 GN=RPS26 PE=1 SV=3 | | 13 | 11 | | 580 | | | 85 | | | 2 | | | 2 | | | 2 | | | | | 21 | |  |  |  |
| P00338 | | L-lactate dehydrogenase A chain OS=Homo sapiens OX=9606 GN=LDHA PE=1 SV=2 | | 36,7 | 8,27 | | 534 | | | 85 | | | 3 | | | 3 | | | 3 | | | | | 9 | |  |  |  |
| P35573 | | Glycogen debranching enzyme OS=Homo sapiens OX=9606 GN=AGL PE=1 SV=3 | | 174,7 | 6,76 | | 495 | | | 85 | | | 3 | | | 3 | | | 3 | | | | | 2 | |  |  |  |
| P42677 | | Small ribosomal subunit protein eS27 OS=Homo sapiens OX=9606 GN=RPS27 PE=1 SV=3 | | 9,5 | 9,45 | | 339 | | | 84 | | | 3 | | | 3 | | | 2 | | | | | 30 | |  |  |  |
| Q13642 | | Four and a half LIM domains protein 1 OS=Homo sapiens OX=9606 GN=FHL1 PE=1 SV=4 | | 36,2 | 8,97 | | 297 | | | 83 | | | 3 | | | 2 | | | 2 | | | | | 7 | |  |  |  |
| P53007 | | Tricarboxylate transport protein, mitochondrial OS=Homo sapiens OX=9606 GN=SLC25A1 PE=1 SV=2 | | 34 | 9,89 | | 410 | | | 83 | | | 3 | | | 3 | | | 3 | | | | | 10 | |  |  |  |
| P31153 | | S-adenosylmethionine synthase isoform type-2 OS=Homo sapiens OX=9606 GN=MAT2A PE=1 SV=1 | | 43,6 | 6,48 | | 318 | | | 82 | | | 2 | | | 2 | | | 2 | | | | | 8 | |  |  |  |
| Q96CT7 | | Coiled-coil domain-containing protein 124 OS=Homo sapiens OX=9606 GN=CCDC124 PE=1 SV=1 | | 25,8 | 9,54 | | 417 | | | 82 | | | 4 | | | 4 | | | 4 | | | | | 17 | |  |  |  |
| A0A075B6R9 | | Probable non-functional immunoglobulin kappa variable 2D-24 OS=Homo sapiens OX=9606 GN=IGKV2D-24 PE=1 SV=1 | | 13,1 | 8,87 | | 190 | | | 82 | | | 4 | | | 2 | | | 1 | | | | | 13 | |  |  |  |
| P35241 | | Radixin OS=Homo sapiens OX=9606 GN=RDX PE=1 SV=1 | | 68,5 | 6,37 | | 329 | | | 82 | | | 2 | | | 2 | | | 2 | | | | | 3 | |  |  |  |
| P06493 | | Cyclin-dependent kinase 1 OS=Homo sapiens OX=9606 GN=CDK1 PE=1 SV=3 | | 34,1 | 8,4 | | 206 | | | 80 | | | 3 | | | 2 | | | 2 | | | | | 7 | |  |  |  |
| Q16527 | | Cysteine and glycine-rich protein 2 OS=Homo sapiens OX=9606 GN=CSRP2 PE=1 SV=3 | | 20,9 | 8,62 | | 821 | | | 80 | | | 3 | | | 3 | | | 3 | | | | | 21 | |  |  |  |
| O14880 | | Glutathione S-transferase 3, mitochondrial OS=Homo sapiens OX=9606 GN=MGST3 PE=1 SV=1 | | 16,5 | 9,38 | | 432 | | | 79 | | | 1 | | | 1 | | | 1 | | | | | 9 | |  |  |  |
| P35237 | | Serpin B6 OS=Homo sapiens OX=9606 GN=SERPINB6 PE=1 SV=3 | | 42,6 | 5,27 | | 326 | | | 79 | | | 2 | | | 2 | | | 2 | | | | | 7 | |  |  |  |
| Q71UM5 | | Ribosomal protein eS27-like OS=Homo sapiens OX=9606 GN=RPS27L PE=1 SV=3 | | 9,5 | 9,45 | | 79 | | | 79 | | | 3 | | | 2 | | | 1 | | | | | 29 | |  |  |  |
| Q00059 | | Transcription factor A, mitochondrial OS=Homo sapiens OX=9606 GN=TFAM PE=1 SV=1 | | 29,1 | 9,72 | | 474 | | | 78 | | | 3 | | | 3 | | | 3 | | | | | 13 | |  |  |  |
| Q13547 | | Histone deacetylase 1 OS=Homo sapiens OX=9606 GN=HDAC1 PE=1 SV=1 | | 55,1 | 5,48 | | 285 | | | 77 | | | 3 | | | 3 | | | 1 | | | | | 8 | |  |  |  |
| P83731 | | Large ribosomal subunit protein eL24 OS=Homo sapiens OX=9606 GN=RPL24 PE=1 SV=1 | | 17,8 | 11,25 | | 564 | | | 77 | | | 1 | | | 1 | | | 1 | | | | | 8 | |  |  |  |
| O76003 | | Glutaredoxin-3 OS=Homo sapiens OX=9606 GN=GLRX3 PE=1 SV=2 | | 37,4 | 5,39 | | 402 | | | 77 | | | 5 | | | 5 | | | 5 | | | | | 14 | |  |  |  |
| Q9H9B4 | | Sideroflexin-1 OS=Homo sapiens OX=9606 GN=SFXN1 PE=1 SV=4 | | 35,6 | 9,07 | | 561 | | | 77 | | | 4 | | | 3 | | | 3 | | | | | 10 | |  |  |  |
| Q96IX5 | | ATP synthase F(0) complex subunit k, mitochondrial OS=Homo sapiens OX=9606 GN=ATP5MK PE=1 SV=1 | | 6,5 | 9,76 | | 649 | | | 76 | | | 1 | | | 1 | | | 1 | | | | | 26 | |  |  |  |
| P61221 | | ATP-binding cassette sub-family E member 1 OS=Homo sapiens OX=9606 GN=ABCE1 PE=1 SV=1 | | 67,3 | 8,34 | | 424 | | | 76 | | | 2 | | | 2 | | | 2 | | | | | 5 | |  |  |  |
| Q02878 | | Large ribosomal subunit protein eL6 OS=Homo sapiens OX=9606 GN=RPL6 PE=1 SV=3 | | 32,7 | 10,58 | | 159 | | | 75 | | | 4 | | | 3 | | | 3 | | | | | 11 | |  |  |  |
| P67812 | | Signal peptidase complex catalytic subunit SEC11A OS=Homo sapiens OX=9606 GN=SEC11A PE=1 SV=1 | | 20,6 | 9,48 | | 802 | | | 74 | | | 2 | | | 2 | | | 2 | | | | | 9 | |  |  |  |
| P50914 | | Large ribosomal subunit protein eL14 OS=Homo sapiens OX=9606 GN=RPL14 PE=1 SV=4 | | 23,4 | 10,93 | | 100 | | | 74 | | | 1 | | | 1 | | | 1 | | | | | 6 | |  |  |  |
| O75323 | | Protein NipSnap homolog 2 OS=Homo sapiens OX=9606 GN=NIPSNAP2 PE=1 SV=1 | | 33,7 | 9,36 | | 257 | | | 74 | | | 2 | | | 2 | | | 2 | | | | | 7 | |  |  |  |
| P62910 | | Large ribosomal subunit protein eL32 OS=Homo sapiens OX=9606 GN=RPL32 PE=1 SV=2 | | 15,9 | 11,33 | | 507 | | | 72 | | | 1 | | | 1 | | | 1 | | | | | 10 | |  |  |  |
| P61204 | | ADP-ribosylation factor 3 OS=Homo sapiens OX=9606 GN=ARF3 PE=1 SV=2 | | 20,6 | 7,43 | | 84 | | | 72 | | | 2 | | | 2 | | | 1 | | | | | 14 | |  |  |  |
| P53985 | | Monocarboxylate transporter 1 OS=Homo sapiens OX=9606 GN=SLC16A1 PE=1 SV=3 | | 53,9 | 8,66 | | 773 | | | 72 | | | 2 | | | 2 | | | 2 | | | | | 5 | |  |  |  |
| Q08945 | | FACT complex subunit SSRP1 OS=Homo sapiens OX=9606 GN=SSRP1 PE=1 SV=1 | | 81 | 6,87 | | 405 | | | 72 | | | 2 | | | 2 | | | 2 | | | | | 3 | |  |  |  |
| Q9H2W6 | | Large ribosomal subunit protein mL46 OS=Homo sapiens OX=9606 GN=MRPL46 PE=1 SV=1 | | 31,7 | 7,05 | | 785 | | | 72 | | | 2 | | | 1 | | | 1 | | | | | 5 | |  |  |  |
| P31948 | | Stress-induced-phosphoprotein 1 OS=Homo sapiens OX=9606 GN=STIP1 PE=1 SV=1 | | 62,6 | 6,8 | | 591 | | | 72 | | | 3 | | | 3 | | | 3 | | | | | 6 | |  |  |  |
| A8MTJ3 | | Guanine nucleotide-binding protein G(t) subunit alpha-3 OS=Homo sapiens OX=9606 GN=GNAT3 PE=1 SV=2 | | 40,3 | 6,01 | | 44 | | | 71 | | | 1 | | | 1 | | | 1 | | | | | 3 | |  |  |  |
| O95793 | | Double-stranded RNA-binding protein Staufen homolog 1 OS=Homo sapiens OX=9606 GN=STAU1 PE=1 SV=2 | | 63,1 | 9,44 | | 99 | | | 70 | | | 3 | | | 3 | | | 3 | | | | | 7 | |  |  |  |
| Q9Y3F4 | | Serine-threonine kinase receptor-associated protein OS=Homo sapiens OX=9606 GN=STRAP PE=1 SV=1 | | 38,4 | 5,12 | | 607 | | | 69 | | | 1 | | | 1 | | | 1 | | | | | 5 | |  |  |  |
| P62891 | | Large ribosomal subunit protein eL39 OS=Homo sapiens OX=9606 GN=RPL39 PE=1 SV=2 | | 6,4 | 12,56 | | 88 | | | 69 | | | 4 | | | 2 | | | 2 | | | | | 20 | |  |  |  |
| P28288 | | ATP-binding cassette sub-family D member 3 OS=Homo sapiens OX=9606 GN=ABCD3 PE=1 SV=1 | | 75,4 | 9,36 | | 153 | | | 68 | | | 2 | | | 2 | | | 2 | | | | | 3 | |  |  |  |
| P30419 | | Glycylpeptide N-tetradecanoyltransferase 1 OS=Homo sapiens OX=9606 GN=NMT1 PE=1 SV=2 | | 56,8 | 7,8 | | 708 | | | 67 | | | 2 | | | 2 | | | 2 | | | | | 5 | |  |  |  |
| Q96AG4 | | Leucine-rich repeat-containing protein 59 OS=Homo sapiens OX=9606 GN=LRRC59 PE=1 SV=1 | | 34,9 | 9,57 | | 252 | | | 67 | | | 3 | | | 3 | | | 3 | | | | | 10 | |  |  |  |
| P13489 | | Ribonuclease inhibitor OS=Homo sapiens OX=9606 GN=RNH1 PE=1 SV=2 | | 49,9 | 4,82 | | 300 | | | 66 | | | 1 | | | 1 | | | 1 | | | | | 3 | |  |  |  |
| P17812 | | CTP synthase 1 OS=Homo sapiens OX=9606 GN=CTPS1 PE=1 SV=2 | | 66,6 | 6,46 | | 788 | | | 66 | | | 2 | | | 2 | | | 2 | | | | | 3 | |  |  |  |
| P51570 | | Galactokinase OS=Homo sapiens OX=9606 GN=GALK1 PE=1 SV=1 | | 42,2 | 6,46 | | 572 | | | 66 | | | 2 | | | 2 | | | 2 | | | | | 6 | |  |  |  |
| P07195 | | L-lactate dehydrogenase B chain OS=Homo sapiens OX=9606 GN=LDHB PE=1 SV=2 | | 36,6 | 6,05 | | 395 | | | 66 | | | 1 | | | 1 | | | 1 | | | | | 5 | |  |  |  |
| P31689 | | DnaJ homolog subfamily A member 1 OS=Homo sapiens OX=9606 GN=DNAJA1 PE=1 SV=2 | | 44,8 | 7,08 | | 545 | | | 66 | | | 2 | | | 2 | | | 2 | | | | | 9 | |  |  |  |
| P62861 | | Ubiquitin-like FUBI-ribosomal protein eS30 fusion protein OS=Homo sapiens OX=9606 GN=FAU PE=1 SV=2 | | 14,4 | 10,17 | | 482 | | | 65 | | | 1 | | | 1 | | | 1 | | | | | 8 | |  |  |  |
| P10515 | | Dihydrolipoyllysine-residue acetyltransferase component of pyruvate dehydrogenase complex, mitochondrial OS=Homo sapiens OX=9606 GN=DLAT PE=1 SV=3 | | 69 | 7,84 | | 568 | | | 65 | | | 1 | | | 1 | | | 1 | | | | | 2 | |  |  |  |
| Q06203 | | Amidophosphoribosyltransferase OS=Homo sapiens OX=9606 GN=PPAT PE=1 SV=1 | | 57,4 | 6,76 | | 816 | | | 65 | | | 2 | | | 2 | | | 2 | | | | | 4 | |  |  |  |
| Q13155 | | Aminoacyl tRNA synthase complex-interacting multifunctional protein 2 OS=Homo sapiens OX=9606 GN=AIMP2 PE=1 SV=2 | | 35,3 | 8,22 | | 478 | | | 65 | | | 1 | | | 1 | | | 1 | | | | | 8 | |  |  |  |
| Q53GS9 | | Ubiquitin carboxyl-terminal hydrolase 39 OS=Homo sapiens OX=9606 GN=USP39 PE=1 SV=2 | | 65,3 | 8,91 | | 66 | | | 64 | | | 3 | | | 3 | | | 3 | | | | | 6 | |  |  |  |
| Q99714 | | 3-hydroxyacyl-CoA dehydrogenase type-2 OS=Homo sapiens OX=9606 GN=HSD17B10 PE=1 SV=3 | | 26,9 | 7,78 | | 543 | | | 64 | | | 2 | | | 2 | | | 2 | | | | | 7 | |  |  |  |
| P09211 | | Glutathione S-transferase P OS=Homo sapiens OX=9606 GN=GSTP1 PE=1 SV=2 | | 23,3 | 5,64 | | 655 | | | 63 | | | 1 | | | 1 | | | 1 | | | | | 5 | |  |  |  |
| Q8N1G4 | | Leucine-rich repeat-containing protein 47 OS=Homo sapiens OX=9606 GN=LRRC47 PE=1 SV=1 | | 63,4 | 8,28 | | 695 | | | 62 | | | 3 | | | 3 | | | 3 | | | | | 6 | |  |  |  |
| O60841 | | Eukaryotic translation initiation factor 5B OS=Homo sapiens OX=9606 GN=EIF5B PE=1 SV=4 | | 138,7 | 5,49 | | 435 | | | 61 | | | 1 | | | 1 | | | 1 | | | | | 1 | |  |  |  |
| Q8WXE9 | | Stonin-2 OS=Homo sapiens OX=9606 GN=STON2 PE=1 SV=1 | | 101,1 | 5,39 | | 162 | | | 60 | | | 2 | | | 1 | | | 1 | | | | | 1 | |  |  |  |
| Q8WUM4 | | Programmed cell death 6-interacting protein OS=Homo sapiens OX=9606 GN=PDCD6IP PE=1 SV=1 | | 96 | 6,52 | | 570 | | | 60 | | | 1 | | | 1 | | | 1 | | | | | 1 | |  |  |  |
| Q93034 | | Cullin-5 OS=Homo sapiens OX=9606 GN=CUL5 PE=1 SV=4 | | 90,9 | 7,94 | | 140 | | | 60 | | | 3 | | | 3 | | | 3 | | | | | 4 | |  |  |  |
| P26583 | | High mobility group protein B2 OS=Homo sapiens OX=9606 GN=HMGB2 PE=1 SV=2 | | 24 | 7,81 | | 91 | | | 60 | | | 2 | | | 2 | | | 2 | | | | | 11 | |  |  |  |
| Q12874 | | Splicing factor 3A subunit 3 OS=Homo sapiens OX=9606 GN=SF3A3 PE=1 SV=1 | | 58,8 | 5,38 | | 421 | | | 59 | | | 2 | | | 2 | | | 2 | | | | | 4 | |  |  |  |
| O95292 | | Vesicle-associated membrane protein-associated protein B/C OS=Homo sapiens OX=9606 GN=VAPB PE=1 SV=3 | | 27,2 | 7,3 | | 69 | | | 59 | | | 1 | | | 1 | | | 1 | | | | | 5 | |  |  |  |
| O95373 | | Importin-7 OS=Homo sapiens OX=9606 GN=IPO7 PE=1 SV=1 | | 119,4 | 4,82 | | 631 | | | 59 | | | 1 | | | 1 | | | 1 | | | | | 1 | |  |  |  |
| O43790 | | Keratin, type II cuticular Hb6 OS=Homo sapiens OX=9606 GN=KRT86 PE=1 SV=1 | | 53,5 | 5,66 | | 461 | | | 59 | | | 4 | | | 4 | | | 3 | | | | | 9 | |  |  |  |
| O00567 | | Nucleolar protein 56 OS=Homo sapiens OX=9606 GN=NOP56 PE=1 SV=4 | | 66 | 9,19 | | 637 | | | 58 | | | 3 | | | 3 | | | 3 | | | | | 5 | |  |  |  |
| Q9BPW8 | | Protein NipSnap homolog 1 OS=Homo sapiens OX=9606 GN=NIPSNAP1 PE=1 SV=1 | | 33,3 | 9,31 | | 707 | | | 58 | | | 1 | | | 1 | | | 1 | | | | | 3 | |  |  |  |
| P36969 | | Phospholipid hydroperoxide glutathione peroxidase GPX4 OS=Homo sapiens OX=9606 GN=GPX4 PE=1 SV=3 | | 22,2 | 8,37 | | 786 | | | 58 | | | 2 | | | 2 | | | 2 | | | | | 13 | |  |  |  |
| Q5RKV6 | | Exosome complex component MTR3 OS=Homo sapiens OX=9606 GN=EXOSC6 PE=1 SV=1 | | 28,2 | 6,28 | | 290 | | | 56 | | | 2 | | | 2 | | | 2 | | | | | 13 | |  |  |  |
| O43684 | | Mitotic checkpoint protein BUB3 OS=Homo sapiens OX=9606 GN=BUB3 PE=1 SV=1 | | 37,1 | 6,84 | | 77 | | | 56 | | | 2 | | | 2 | | | 2 | | | | | 6 | |  |  |  |
| P31930 | | Cytochrome b-c1 complex subunit 1, mitochondrial OS=Homo sapiens OX=9606 GN=UQCRC1 PE=1 SV=3 | | 52,6 | 6,37 | | 621 | | | 56 | | | 1 | | | 1 | | | 1 | | | | | 2 | |  |  |  |
| P51148 | | Ras-related protein Rab-5C OS=Homo sapiens OX=9606 GN=RAB5C PE=1 SV=2 | | 23,5 | 8,41 | | 221 | | | 55 | | | 1 | | | 1 | | | 1 | | | | | 5 | |  |  |  |
| P62314 | | Small nuclear ribonucleoprotein Sm D1 OS=Homo sapiens OX=9606 GN=SNRPD1 PE=1 SV=1 | | 13,3 | 11,56 | | 197 | | | 55 | | | 2 | | | 1 | | | 1 | | | | | 11 | |  |  |  |
| P51116 | | RNA-binding protein FXR2 OS=Homo sapiens OX=9606 GN=FXR2 PE=1 SV=2 | | 74,2 | 6,23 | | 806 | | | 55 | | | 2 | | | 2 | | | 2 | | | | | 3 | |  |  |  |
| Q14C86 | | GTPase-activating protein and VPS9 domain-containing protein 1 OS=Homo sapiens OX=9606 GN=GAPVD1 PE=1 SV=2 | | 164,9 | 5,22 | | 8 | | | 54 | | | 1 | | | 1 | | | 1 | | | | | 1 | |  |  |  |
| P55060 | | Exportin-2 OS=Homo sapiens OX=9606 GN=CSE1L PE=1 SV=3 | | 110,3 | 5,77 | | 311 | | | 54 | | | 2 | | | 2 | | | 2 | | | | | 2 | |  |  |  |
| Q9NRF8 | | CTP synthase 2 OS=Homo sapiens OX=9606 GN=CTPS2 PE=1 SV=1 | | 65,6 | 6,9 | | 256 | | | 53 | | | 1 | | | 1 | | | 1 | | | | | 2 | |  |  |  |
| P35613 | | Basigin OS=Homo sapiens OX=9606 GN=BSG PE=1 SV=2 | | 42,2 | 5,66 | | 699 | | | 52 | | | 2 | | | 2 | | | 2 | | | | | 8 | |  |  |  |
| P62318 | | Small nuclear ribonucleoprotein Sm D3 OS=Homo sapiens OX=9606 GN=SNRPD3 PE=1 SV=1 | | 13,9 | 10,32 | | 120 | | | 51 | | | 1 | | | 1 | | | 1 | | | | | 8 | |  |  |  |
| Q15424 | | Scaffold attachment factor B1 OS=Homo sapiens OX=9606 GN=SAFB PE=1 SV=4 | | 102,6 | 5,47 | | 48 | | | 51 | | | 2 | | | 2 | | | 2 | | | | | 2 | |  |  |  |
| P42766 | | Large ribosomal subunit protein uL29 OS=Homo sapiens OX=9606 GN=RPL35 PE=1 SV=2 | | 14,5 | 11,05 | | 175 | | | 51 | | | 2 | | | 2 | | | 2 | | | | | 14 | |  |  |  |
| P55209 | | Nucleosome assembly protein 1-like 1 OS=Homo sapiens OX=9606 GN=NAP1L1 PE=1 SV=1 | | 45,3 | 4,46 | | 227 | | | 50 | | | 2 | | | 1 | | | 1 | | | | | 3 | |  |  |  |
| P54577 | | Tyrosine--tRNA ligase, cytoplasmic OS=Homo sapiens OX=9606 GN=YARS1 PE=1 SV=4 | | 59,1 | 7,05 | | 615 | | | 50 | | | 1 | | | 1 | | | 1 | | | | | 2 | |  |  |  |
| Q07020 | | Large ribosomal subunit protein eL18 OS=Homo sapiens OX=9606 GN=RPL18 PE=1 SV=2 | | 21,6 | 11,72 | | 384 | | | 50 | | | 1 | | | 1 | | | 1 | | | | | 7 | |  |  |  |
| P04080 | | Cystatin-B OS=Homo sapiens OX=9606 GN=CSTB PE=1 SV=2 | | 11,1 | 7,56 | | 533 | | | 50 | | | 1 | | | 1 | | | 1 | | | | | 12 | |  |  |  |
| O60264 | | SWI/SNF-related matrix-associated actin-dependent regulator of chromatin subfamily A member 5 OS=Homo sapiens OX=9606 GN=SMARCA5 PE=1 SV=1 | | 121,8 | 8,09 | | 75 | | | 50 | | | 2 | | | 2 | | | 2 | | | | | 2 | |  |  |  |
| O14818 | | Proteasome subunit alpha type-7 OS=Homo sapiens OX=9606 GN=PSMA7 PE=1 SV=1 | | 27,9 | 8,46 | | 768 | | | 50 | | | 2 | | | 2 | | | 2 | | | | | 12 | |  |  |  |
| Q7L0Y3 | | tRNA methyltransferase 10 homolog C OS=Homo sapiens OX=9606 GN=TRMT10C PE=1 SV=2 | | 47,3 | 9,36 | | 191 | | | 50 | | | 2 | | | 2 | | | 2 | | | | | 4 | |  |  |  |
| P31942 | | Heterogeneous nuclear ribonucleoprotein H3 OS=Homo sapiens OX=9606 GN=HNRNPH3 PE=1 SV=2 | | 36,9 | 6,87 | | 476 | | | 49 | | | 2 | | | 2 | | | 2 | | | | | 5 | |  |  |  |
| P35998 | | 26S proteasome regulatory subunit 7 OS=Homo sapiens OX=9606 GN=PSMC2 PE=1 SV=3 | | 48,6 | 5,95 | | 95 | | | 49 | | | 2 | | | 2 | | | 2 | | | | | 6 | |  |  |  |
| P55072 | | Transitional endoplasmic reticulum ATPase OS=Homo sapiens OX=9606 GN=VCP PE=1 SV=4 | | 89,3 | 5,26 | | 111 | | | 48 | | | 2 | | | 2 | | | 2 | | | | | 3 | |  |  |  |
| O60762 | | Dolichol-phosphate mannosyltransferase subunit 1 OS=Homo sapiens OX=9606 GN=DPM1 PE=1 SV=1 | | 29,6 | 9,57 | | 491 | | | 46 | | | 2 | | | 2 | | | 2 | | | | | 7 | |  |  |  |
| P21912 | | Succinate dehydrogenase [ubiquinone] iron-sulfur subunit, mitochondrial OS=Homo sapiens OX=9606 GN=SDHB PE=1 SV=3 | | 31,6 | 8,76 | | 34 | | | 46 | | | 1 | | | 1 | | | 1 | | | | | 3 | |  |  |  |
| P27694 | | Replication protein A 70 kDa DNA-binding subunit OS=Homo sapiens OX=9606 GN=RPA1 PE=1 SV=2 | | 68,1 | 7,21 | | 467 | | | 46 | | | 2 | | | 2 | | | 2 | | | | | 4 | |  |  |  |
| Q9Y5B9 | | FACT complex subunit SPT16 OS=Homo sapiens OX=9606 GN=SUPT16H PE=1 SV=1 | | 119,8 | 5,66 | | 757 | | | 46 | | | 1 | | | 1 | | | 1 | | | | | 1 | |  |  |  |
| P21108 | | Ribose-phosphate pyrophosphokinase 3 OS=Homo sapiens OX=9606 GN=PRPS1L1 PE=1 SV=2 | | 34,8 | 6,35 | | 118 | | | 46 | | | 1 | | | 1 | | | 1 | | | | | 4 | |  |  |  |
| P17980 | | 26S proteasome regulatory subunit 6A OS=Homo sapiens OX=9606 GN=PSMC3 PE=1 SV=3 | | 49,2 | 5,24 | | 68 | | | 45 | | | 2 | | | 2 | | | 2 | | | | | 7 | |  |  |  |
| O43920 | | NADH dehydrogenase [ubiquinone] iron-sulfur protein 5 OS=Homo sapiens OX=9606 GN=NDUFS5 PE=1 SV=3 | | 12,5 | 9,14 | | 524 | | | 45 | | | 1 | | | 1 | | | 1 | | | | | 11 | |  |  |  |
| O75369 | | Filamin-B OS=Homo sapiens OX=9606 GN=FLNB PE=1 SV=2 | | 278 | 5,73 | | 578 | | | 45 | | | 2 | | | 2 | | | 1 | | | | | 1 | |  |  |  |
| P49915 | | GMP synthase [glutamine-hydrolyzing] OS=Homo sapiens OX=9606 GN=GMPS PE=1 SV=1 | | 76,7 | 6,87 | | 201 | | | 45 | | | 3 | | | 3 | | | 3 | | | | | 5 | |  |  |  |
| O00154 | | Cytosolic acyl coenzyme A thioester hydrolase OS=Homo sapiens OX=9606 GN=ACOT7 PE=1 SV=3 | | 41,8 | 8,54 | | 719 | | | 45 | | | 1 | | | 1 | | | 1 | | | | | 3 | |  |  |  |
| P61160 | | Actin-related protein 2 OS=Homo sapiens OX=9606 GN=ACTR2 PE=1 SV=1 | | 44,7 | 6,74 | | 314 | | | 45 | | | 1 | | | 1 | | | 1 | | | | | 3 | |  |  |  |
| Q9Y3B9 | | RRP15-like protein OS=Homo sapiens OX=9606 GN=RRP15 PE=1 SV=2 | | 31,5 | 5,52 | | 711 | | | 44 | | | 1 | | | 1 | | | 1 | | | | | 5 | |  |  |  |
| P62899 | | Large ribosomal subunit protein eL31 OS=Homo sapiens OX=9606 GN=RPL31 PE=1 SV=1 | | 14,5 | 10,54 | | 359 | | | 44 | | | 2 | | | 2 | | | 2 | | | | | 18 | |  |  |  |
| P27635 | | Large ribosomal subunit protein uL16 OS=Homo sapiens OX=9606 GN=RPL10 PE=1 SV=5 | | 24,6 | 10,08 | | 742 | | | 44 | | | 1 | | | 1 | | | 1 | | | | | 6 | |  |  |  |
| O00178 | | GTP-binding protein 1 OS=Homo sapiens OX=9606 GN=GTPBP1 PE=1 SV=3 | | 72,4 | 8,34 | | 174 | | | 43 | | | 2 | | | 2 | | | 2 | | | | | 4 | |  |  |  |
| P43307 | | Translocon-associated protein subunit alpha OS=Homo sapiens OX=9606 GN=SSR1 PE=1 SV=3 | | 32,2 | 4,49 | | 413 | | | 43 | | | 1 | | | 1 | | | 1 | | | | | 3 | |  |  |  |
| O15371 | | Eukaryotic translation initiation factor 3 subunit D OS=Homo sapiens OX=9606 GN=EIF3D PE=1 SV=1 | | 63,9 | 6,05 | | 237 | | | 43 | | | 1 | | | 1 | | | 1 | | | | | 3 | |  |  |  |
| Q01105 | | Protein SET OS=Homo sapiens OX=9606 GN=SET PE=1 SV=3 | | 33,5 | 4,32 | | 110 | | | 42 | | | 2 | | | 2 | | | 2 | | | | | 10 | |  |  |  |
| P35606 | | Coatomer subunit beta' OS=Homo sapiens OX=9606 GN=COPB2 PE=1 SV=2 | | 102,4 | 5,27 | | 698 | | | 42 | | | 2 | | | 2 | | | 2 | | | | | 3 | |  |  |  |
| Q9UQ35 | | Serine/arginine repetitive matrix protein 2 OS=Homo sapiens OX=9606 GN=SRRM2 PE=1 SV=2 | | 299,4 | 12,06 | | 350 | | | 42 | | | 2 | | | 2 | | | 2 | | | | | 1 | |  |  |  |
| Q9Y520 | | Protein PRRC2C OS=Homo sapiens OX=9606 GN=PRRC2C PE=1 SV=4 | | 316,7 | 9,13 | | 307 | | | 42 | | | 2 | | | 2 | | | 2 | | | | | 1 | |  |  |  |
| P68400 | | Casein kinase II subunit alpha OS=Homo sapiens OX=9606 GN=CSNK2A1 PE=1 SV=1 | | 45,1 | 7,74 | | 602 | | | 41 | | | 1 | | | 1 | | | 1 | | | | | 3 | |  |  |  |
| Q53H12 | | Acylglycerol kinase, mitochondrial OS=Homo sapiens OX=9606 GN=AGK PE=1 SV=2 | | 47,1 | 8,09 | | 701 | | | 41 | | | 1 | | | 1 | | | 1 | | | | | 3 | |  |  |  |
| Q13247 | | Serine/arginine-rich splicing factor 6 OS=Homo sapiens OX=9606 GN=SRSF6 PE=1 SV=2 | | 39,6 | 11,43 | | 499 | | | 41 | | | 1 | | | 1 | | | 1 | | | | | 3 | |  |  |  |
| P12814 | | Alpha-actinin-1 OS=Homo sapiens OX=9606 GN=ACTN1 PE=1 SV=2 | | 103 | 5,41 | | 334 | | | 40 | | | 3 | | | 3 | | | 1 | | | | | 4 | |  |  |  |
| Q9BSD7 | | Cancer-related nucleoside-triphosphatase OS=Homo sapiens OX=9606 GN=NTPCR PE=1 SV=1 | | 20,7 | 9,54 | | 794 | | | 40 | | | 1 | | | 1 | | | 1 | | | | | 8 | |  |  |  |
| O43795 | | Unconventional myosin-Ib OS=Homo sapiens OX=9606 GN=MYO1B PE=1 SV=3 | | 131,9 | 9,38 | | 652 | | | 40 | | | 2 | | | 2 | | | 2 | | | | | 2 | |  |  |  |
| Q13242 | | Serine/arginine-rich splicing factor 9 OS=Homo sapiens OX=9606 GN=SRSF9 PE=1 SV=1 | | 25,5 | 8,65 | | 418 | | | 40 | | | 1 | | | 1 | | | 1 | | | | | 5 | |  |  |  |
| Q96EY4 | | Translation machinery-associated protein 16 OS=Homo sapiens OX=9606 GN=TMA16 PE=1 SV=2 | | 23,8 | 9,26 | | 82 | | | 40 | | | 2 | | | 2 | | | 2 | | | | | 11 | |  |  |  |
| O00487 | | 26S proteasome non-ATPase regulatory subunit 14 OS=Homo sapiens OX=9606 GN=PSMD14 PE=1 SV=1 | | 34,6 | 6,52 | | 589 | | | 40 | | | 1 | | | 1 | | | 1 | | | | | 4 | |  |  |  |
| P08574 | | Cytochrome c1, heme protein, mitochondrial OS=Homo sapiens OX=9606 GN=CYC1 PE=1 SV=3 | | 35,4 | 9 | | 679 | | | 40 | | | 2 | | | 2 | | | 2 | | | | | 5 | |  |  |  |
| Q5H9R7 | | Serine/threonine-protein phosphatase 6 regulatory subunit 3 OS=Homo sapiens OX=9606 GN=PPP6R3 PE=1 SV=2 | | 97,6 | 4,6 | | 628 | | | 40 | | | 1 | | | 1 | | | 1 | | | | | 1 | |  |  |  |
| P30048 | | Thioredoxin-dependent peroxide reductase, mitochondrial OS=Homo sapiens OX=9606 GN=PRDX3 PE=1 SV=3 | | 27,7 | 7,78 | | 357 | | | 39 | | | 2 | | | 2 | | | 2 | | | | | 7 | |  |  |  |
| Q15287 | | RNA-binding protein with serine-rich domain 1 OS=Homo sapiens OX=9606 GN=RNPS1 PE=1 SV=1 | | 34,2 | 11,84 | | 715 | | | 39 | | | 2 | | | 2 | | | 2 | | | | | 6 | |  |  |  |
| Q96DA0 | | Pancreatic adenocarcinoma up-regulated factor OS=Homo sapiens OX=9606 GN=ZG16B PE=1 SV=4 | | 18,9 | 5,5 | | 493 | | | 39 | | | 1 | | | 1 | | | 1 | | | | | 9 | |  |  |  |
| P31025 | | Lipocalin-1 OS=Homo sapiens OX=9606 GN=LCN1 PE=1 SV=1 | | 19,2 | 5,58 | | 78 | | | 39 | | | 1 | | | 1 | | | 1 | | | | | 6 | |  |  |  |
| P40939 | | Trifunctional enzyme subunit alpha, mitochondrial OS=Homo sapiens OX=9606 GN=HADHA PE=1 SV=2 | | 82,9 | 9,04 | | 422 | | | 38 | | | 1 | | | 1 | | | 1 | | | | | 2 | |  |  |  |
| P32322 | | Pyrroline-5-carboxylate reductase 1, mitochondrial OS=Homo sapiens OX=9606 GN=PYCR1 PE=1 SV=2 | | 33,3 | 7,61 | | 228 | | | 38 | | | 1 | | | 1 | | | 1 | | | | | 3 | |  |  |  |
| Q8NI36 | | WD repeat-containing protein 36 OS=Homo sapiens OX=9606 GN=WDR36 PE=1 SV=2 | | 99,3 | 7,36 | | 509 | | | 38 | | | 1 | | | 1 | | | 1 | | | | | 2 | |  |  |  |
| P61626 | | Lysozyme C OS=Homo sapiens OX=9606 GN=LYZ PE=1 SV=1 | | 16,5 | 9,16 | | 504 | | | 38 | | | 1 | | | 1 | | | 1 | | | | | 8 | |  |  |  |
| Q9NZ01 | | Very-long-chain enoyl-CoA reductase OS=Homo sapiens OX=9606 GN=TECR PE=1 SV=1 | | 36 | 9,45 | | 751 | | | 38 | | | 1 | | | 1 | | | 1 | | | | | 4 | |  |  |  |
| Q92504 | | Zinc transporter SLC39A7 OS=Homo sapiens OX=9606 GN=SLC39A7 PE=1 SV=2 | | 50,1 | 6,87 | | 446 | | | 38 | | | 2 | | | 1 | | | 1 | | | | | 3 | |  |  |  |
| Q9NVS9 | | Pyridoxine-5'-phosphate oxidase OS=Homo sapiens OX=9606 GN=PNPO PE=1 SV=1 | | 30 | 7,06 | | 767 | | | 37 | | | 2 | | | 2 | | | 2 | | | | | 9 | |  |  |  |
| O95433 | | Activator of 90 kDa heat shock protein ATPase homolog 1 OS=Homo sapiens OX=9606 GN=AHSA1 PE=1 SV=1 | | 38,3 | 5,53 | | 511 | | | 37 | | | 2 | | | 2 | | | 2 | | | | | 7 | |  |  |  |
| P62191 | | 26S proteasome regulatory subunit 4 OS=Homo sapiens OX=9606 GN=PSMC1 PE=1 SV=1 | | 49,2 | 6,21 | | 774 | | | 37 | | | 3 | | | 3 | | | 2 | | | | | 9 | |  |  |  |
| P00390 | | Glutathione reductase, mitochondrial OS=Homo sapiens OX=9606 GN=GSR PE=1 SV=2 | | 56,2 | 8,5 | | 89 | | | 37 | | | 1 | | | 1 | | | 1 | | | | | 2 | |  |  |  |
| Q7Z2W4 | | Zinc finger CCCH-type antiviral protein 1 OS=Homo sapiens OX=9606 GN=ZC3HAV1 PE=1 SV=3 | | 101,4 | 8,4 | | 51 | | | 37 | | | 1 | | | 1 | | | 1 | | | | | 2 | |  |  |  |
| P15735 | | Phosphorylase b kinase gamma catalytic chain, liver/testis isoform OS=Homo sapiens OX=9606 GN=PHKG2 PE=1 SV=1 | | 46,4 | 6,38 | | 665 | | | 36 | | | 1 | | | 1 | | | 1 | | | | | 4 | |  |  |  |
| P46087 | | 28S rRNA (cytosine(4447)-C(5))-methyltransferase OS=Homo sapiens OX=9606 GN=NOP2 PE=1 SV=2 | | 89,2 | 9,23 | | 583 | | | 36 | | | 1 | | | 1 | | | 1 | | | | | 1 | |  |  |  |
| Q9UBX3 | | Mitochondrial dicarboxylate carrier OS=Homo sapiens OX=9606 GN=SLC25A10 PE=1 SV=2 | | 31,3 | 9,54 | | 502 | | | 36 | | | 1 | | | 1 | | | 1 | | | | | 3 | |  |  |  |
| P42704 | | Leucine-rich PPR motif-containing protein, mitochondrial OS=Homo sapiens OX=9606 GN=LRPPRC PE=1 SV=3 | | 157,8 | 6,13 | | 351 | | | 36 | | | 1 | | | 1 | | | 1 | | | | | 1 | |  |  |  |
| P57088 | | Transmembrane protein 33 OS=Homo sapiens OX=9606 GN=TMEM33 PE=1 SV=2 | | 28 | 9,7 | | 194 | | | 36 | | | 1 | | | 1 | | | 1 | | | | | 3 | |  |  |  |
| O60884 | | DnaJ homolog subfamily A member 2 OS=Homo sapiens OX=9606 GN=DNAJA2 PE=1 SV=1 | | 45,7 | 6,48 | | 664 | | | 36 | | | 2 | | | 2 | | | 2 | | | | | 5 | |  |  |  |
| Q8IZL8 | | Proline-, glutamic acid- and leucine-rich protein 1 OS=Homo sapiens OX=9606 GN=PELP1 PE=1 SV=2 | | 119,6 | 4,34 | | 223 | | | 36 | | | 1 | | | 1 | | | 1 | | | | | 3 | |  |  |  |
| P61619 | | Protein transport protein Sec61 subunit alpha isoform 1 OS=Homo sapiens OX=9606 GN=SEC61A1 PE=1 SV=2 | | 52,2 | 8,06 | | 137 | | | 35 | | | 1 | | | 1 | | | 1 | | | | | 2 | |  |  |  |
| Q9GZP4 | | PITH domain-containing protein 1 OS=Homo sapiens OX=9606 GN=PITHD1 PE=1 SV=1 | | 24,2 | 5,74 | | 225 | | | 35 | | | 2 | | | 2 | | | 2 | | | | | 13 | |  |  |  |
| O95456 | | Proteasome assembly chaperone 1 OS=Homo sapiens OX=9606 GN=PSMG1 PE=1 SV=1 | | 32,8 | 7,17 | | 168 | | | 34 | | | 1 | | | 1 | | | 1 | | | | | 5 | |  |  |  |
| Q14157 | | Ubiquitin-associated protein 2-like OS=Homo sapiens OX=9606 GN=UBAP2L PE=1 SV=2 | | 114,5 | 7,11 | | 718 | | | 34 | | | 2 | | | 2 | | | 2 | | | | | 3 | |  |  |  |
| P19525 | | Interferon-induced, double-stranded RNA-activated protein kinase OS=Homo sapiens OX=9606 GN=EIF2AK2 PE=1 SV=2 | | 62,1 | 8,4 | | 753 | | | 34 | | | 1 | | | 1 | | | 1 | | | | | 1 | |  |  |  |
| P31040 | | Succinate dehydrogenase [ubiquinone] flavoprotein subunit, mitochondrial OS=Homo sapiens OX=9606 GN=SDHA PE=1 SV=2 | | 72,6 | 7,39 | | 411 | | | 34 | | | 1 | | | 1 | | | 1 | | | | | 3 | |  |  |  |
| O00139 | | Kinesin-like protein KIF2A OS=Homo sapiens OX=9606 GN=KIF2A PE=1 SV=3 | | 79,9 | 6,68 | | 378 | | | 34 | | | 1 | | | 1 | | | 1 | | | | | 2 | |  |  |  |
| P61077 | | Ubiquitin-conjugating enzyme E2 D3 OS=Homo sapiens OX=9606 GN=UBE2D3 PE=1 SV=1 | | 16,7 | 7,8 | | 155 | | | 33 | | | 1 | | | 1 | | | 1 | | | | | 7 | |  |  |  |
| Q96PK6 | | RNA-binding protein 14 OS=Homo sapiens OX=9606 GN=RBM14 PE=1 SV=2 | | 69,4 | 9,67 | | 181 | | | 33 | | | 2 | | | 2 | | | 2 | | | | | 4 | |  |  |  |
| Q9UG63 | | ATP-binding cassette sub-family F member 2 OS=Homo sapiens OX=9606 GN=ABCF2 PE=1 SV=2 | | 71,2 | 7,37 | | 530 | | | 32 | | | 1 | | | 1 | | | 1 | | | | | 1 | |  |  |  |
| Q9Y5A9 | | YTH domain-containing family protein 2 OS=Homo sapiens OX=9606 GN=YTHDF2 PE=1 SV=2 | | 62,3 | 8,79 | | 685 | | | 32 | | | 2 | | | 2 | | | 1 | | | | | 4 | |  |  |  |
| Q9H583 | | HEAT repeat-containing protein 1 OS=Homo sapiens OX=9606 GN=HEATR1 PE=1 SV=3 | | 242,2 | 6,54 | | 676 | | | 32 | | | 1 | | | 1 | | | 1 | | | | | 1 | |  |  |  |
| Q9UHV9 | | Prefoldin subunit 2 OS=Homo sapiens OX=9606 GN=PFDN2 PE=1 SV=1 | | 16,6 | 6,58 | | 282 | | | 31 | | | 1 | | | 1 | | | 1 | | | | | 8 | |  |  |  |
| Q9HC36 | | rRNA methyltransferase 3, mitochondrial OS=Homo sapiens OX=9606 GN=MRM3 PE=1 SV=2 | | 47 | 8,73 | | 565 | | | 31 | | | 1 | | | 1 | | | 1 | | | | | 3 | |  |  |  |
| Q96EY1 | | DnaJ homolog subfamily A member 3, mitochondrial OS=Homo sapiens OX=9606 GN=DNAJA3 PE=1 SV=2 | | 52,5 | 9,26 | | 103 | | | 31 | | | 1 | | | 1 | | | 1 | | | | | 3 | |  |  |  |
| Q9H0D6 | | 5'-3' exoribonuclease 2 OS=Homo sapiens OX=9606 GN=XRN2 PE=1 SV=1 | | 108,5 | 7,47 | | 765 | | | 30 | | | 1 | | | 1 | | | 1 | | | | | 1 | |  |  |  |
| Q9BS26 | | Endoplasmic reticulum resident protein 44 OS=Homo sapiens OX=9606 GN=ERP44 PE=1 SV=1 | | 46,9 | 5,26 | | 617 | | | 30 | | | 1 | | | 1 | | | 1 | | | | | 2 | |  |  |  |
| Q9UHD1 | | Cysteine and histidine-rich domain-containing protein 1 OS=Homo sapiens OX=9606 GN=CHORDC1 PE=1 SV=2 | | 37,5 | 7,87 | | 451 | | | 30 | | | 1 | | | 1 | | | 1 | | | | | 2 | |  |  |  |
| P46109 | | Crk-like protein OS=Homo sapiens OX=9606 GN=CRKL PE=1 SV=1 | | 33,8 | 6,74 | | 157 | | | 29 | | | 1 | | | 1 | | | 1 | | | | | 4 | |  |  |  |
| O15269 | | Serine palmitoyltransferase 1 OS=Homo sapiens OX=9606 GN=SPTLC1 PE=1 SV=1 | | 52,7 | 6,01 | | 616 | | | 29 | | | 1 | | | 1 | | | 1 | | | | | 4 | |  |  |  |
| P61026 | | Ras-related protein Rab-10 OS=Homo sapiens OX=9606 GN=RAB10 PE=1 SV=1 | | 22,5 | 8,38 | | 17 | | | 29 | | | 1 | | | 1 | | | 1 | | | | | 6 | |  |  |  |
| Q00577 | | Transcriptional activator protein Pur-alpha OS=Homo sapiens OX=9606 GN=PURA PE=1 SV=2 | | 34,9 | 6,44 | | 1 | | | 29 | | | 1 | | | 1 | | | 1 | | | | | 3 | |  |  |  |
| O43395 | | U4/U6 small nuclear ribonucleoprotein Prp3 OS=Homo sapiens OX=9606 GN=PRPF3 PE=1 SV=2 | | 77,5 | 9,5 | | 582 | | | 28 | | | 1 | | | 1 | | | 1 | | | | | 1 | |  |  |  |
| P30153 | | Serine/threonine-protein phosphatase 2A 65 kDa regulatory subunit A alpha isoform OS=Homo sapiens OX=9606 GN=PPP2R1A PE=1 SV=4 | | 65,3 | 5,11 | | 383 | | | 28 | | | 1 | | | 1 | | | 1 | | | | | 2 | |  |  |  |
| P59998 | | Actin-related protein 2/3 complex subunit 4 OS=Homo sapiens OX=9606 GN=ARPC4 PE=1 SV=3 | | 19,7 | 8,43 | | 165 | | | 28 | | | 1 | | | 1 | | | 1 | | | | | 5 | |  |  |  |
| P43490 | | Nicotinamide phosphoribosyltransferase OS=Homo sapiens OX=9606 GN=NAMPT PE=1 SV=1 | | 55,5 | 7,15 | | 613 | | | 28 | | | 1 | | | 1 | | | 1 | | | | | 3 | |  |  |  |
| P02765 | | Alpha-2-HS-glycoprotein OS=Homo sapiens OX=9606 GN=AHSG PE=1 SV=2 | | 39,3 | 5,72 | | 71 | | | 28 | | | 1 | | | 1 | | | 1 | | | | | 3 | |  |  |  |
| Q9Y266 | | Nuclear migration protein nudC OS=Homo sapiens OX=9606 GN=NUDC PE=1 SV=1 | | 38,2 | 5,38 | | 253 | | | 27 | | | 1 | | | 1 | | | 1 | | | | | 5 | |  |  |  |
| P08758 | | Annexin A5 OS=Homo sapiens OX=9606 GN=ANXA5 PE=1 SV=2 | | 35,9 | 5,05 | | 738 | | | 27 | | | 2 | | | 2 | | | 2 | | | | | 6 | |  |  |  |
| P53618 | | Coatomer subunit beta OS=Homo sapiens OX=9606 GN=COPB1 PE=1 SV=3 | | 107,1 | 6,05 | | 387 | | | 27 | | | 3 | | | 3 | | | 3 | | | | | 5 | |  |  |  |
| Q92979 | | Ribosomal RNA small subunit methyltransferase NEP1 OS=Homo sapiens OX=9606 GN=EMG1 PE=1 SV=4 | | 26,7 | 9,17 | | 198 | | | 27 | | | 1 | | | 1 | | | 1 | | | | | 5 | |  |  |  |
| P02788 | | Lactotransferrin OS=Homo sapiens OX=9606 GN=LTF PE=1 SV=6 | | 78,1 | 8,12 | | 648 | | | 26 | | | 1 | | | 1 | | | 1 | | | | | 1 | |  |  |  |
| O15067 | | Phosphoribosylformylglycinamidine synthase OS=Homo sapiens OX=9606 GN=PFAS PE=1 SV=4 | | 144,6 | 5,76 | | 286 | | | 26 | | | 1 | | | 1 | | | 1 | | | | | 1 | |  |  |  |
| Q99873 | | Protein arginine N-methyltransferase 1 OS=Homo sapiens OX=9606 GN=PRMT1 PE=1 SV=3 | | 42,4 | 5,35 | | 398 | | | 26 | | | 1 | | | 1 | | | 1 | | | | | 3 | |  |  |  |
| Q92599 | | Septin-8 OS=Homo sapiens OX=9606 GN=SEPTIN8 PE=1 SV=4 | | 55,7 | 6,28 | | 344 | | | 26 | | | 2 | | | 2 | | | 2 | | | | | 4 | |  |  |  |
| Q8NFW8 | | N-acylneuraminate cytidylyltransferase OS=Homo sapiens OX=9606 GN=CMAS PE=1 SV=2 | | 48,3 | 7,93 | | 180 | | | 26 | | | 1 | | | 1 | | | 1 | | | | | 2 | |  |  |  |
| Q9H2U2 | | Inorganic pyrophosphatase 2, mitochondrial OS=Homo sapiens OX=9606 GN=PPA2 PE=1 SV=2 | | 37,9 | 7,39 | | 340 | | | 26 | | | 1 | | | 1 | | | 1 | | | | | 2 | |  |  |  |
| Q14676 | | Mediator of DNA damage checkpoint protein 1 OS=Homo sapiens OX=9606 GN=MDC1 PE=1 SV=3 | | 226,5 | 5,47 | | 119 | | | 25 | | | 2 | | | 2 | | | 2 | | | | | 3 | |  |  |  |
| Q9P016 | | Thymocyte nuclear protein 1 OS=Homo sapiens OX=9606 GN=THYN1 PE=1 SV=1 | | 25,7 | 9,25 | | 694 | | | 25 | | | 1 | | | 1 | | | 1 | | | | | 3 | |  |  |  |
| O75964 | | ATP synthase F(0) complex subunit g, mitochondrial OS=Homo sapiens OX=9606 GN=ATP5MG PE=1 SV=3 | | 11,4 | 9,64 | | 53 | | | 25 | | | 1 | | | 1 | | | 1 | | | | | 13 | |  |  |  |
| Q9ULV0 | | Unconventional myosin-Vb OS=Homo sapiens OX=9606 GN=MYO5B PE=1 SV=3 | | 213,5 | 7,2 | | 369 | | | 24 | | | 1 | | | 1 | | | 1 | | | | | 0 | |  |  |  |
| Q14974 | | Importin subunit beta-1 OS=Homo sapiens OX=9606 GN=KPNB1 PE=1 SV=2 | | 97,1 | 4,78 | | 92 | | | 24 | | | 2 | | | 2 | | | 2 | | | | | 3 | |  |  |  |
| Q7Z7B0 | | Filamin-A-interacting protein 1 OS=Homo sapiens OX=9606 GN=FILIP1 PE=1 SV=1 | | 138 | 8,32 | | 298 | | | 24 | | | 3 | | | 1 | | | 1 | | | | | 1 | |  |  |  |
| Q9H0S4 | | Probable ATP-dependent RNA helicase DDX47 OS=Homo sapiens OX=9606 GN=DDX47 PE=1 SV=1 | | 50,6 | 9,1 | | 177 | | | 24 | | | 1 | | | 1 | | | 1 | | | | | 2 | |  |  |  |
| Q10567 | | AP-1 complex subunit beta-1 OS=Homo sapiens OX=9606 GN=AP1B1 PE=1 SV=3 | | 104,5 | 5,06 | | 526 | | | 24 | | | 1 | | | 1 | | | 1 | | | | | 1 | |  |  |  |
| O43592 | | Exportin-T OS=Homo sapiens OX=9606 GN=XPOT PE=1 SV=2 | | 109,9 | 5,39 | | 733 | | | 24 | | | 1 | | | 1 | | | 1 | | | | | 1 | |  |  |  |
| Q13428 | | Treacle protein OS=Homo sapiens OX=9606 GN=TCOF1 PE=1 SV=3 | | 152 | 9,04 | | 812 | | | 23 | | | 1 | | | 1 | | | 1 | | | | | 1 | |  |  |  |
| Q9UKD2 | | mRNA turnover protein 4 homolog OS=Homo sapiens OX=9606 GN=MRTO4 PE=1 SV=2 | | 27,5 | 8,29 | | 265 | | | 23 | | | 1 | | | 1 | | | 1 | | | | | 5 | |  |  |  |
| Q9BXJ9 | | N-alpha-acetyltransferase 15, NatA auxiliary subunit OS=Homo sapiens OX=9606 GN=NAA15 PE=1 SV=1 | | 101,2 | 7,42 | | 43 | | | 23 | | | 1 | | | 1 | | | 1 | | | | | 2 | |  |  |  |
| Q13868 | | Exosome complex component RRP4 OS=Homo sapiens OX=9606 GN=EXOSC2 PE=1 SV=2 | | 32,8 | 7,5 | | 769 | | | 23 | | | 1 | | | 1 | | | 1 | | | | | 5 | |  |  |  |
| P60981 | | Destrin OS=Homo sapiens OX=9606 GN=DSTN PE=1 SV=3 | | 18,5 | 7,85 | | 784 | | | 23 | | | 1 | | | 1 | | | 1 | | | | | 7 | |  |  |  |
| Q9NZB2 | | Constitutive coactivator of PPAR-gamma-like protein 1 OS=Homo sapiens OX=9606 GN=FAM120A PE=1 SV=2 | | 121,8 | 8,88 | | 638 | | | 23 | | | 1 | | | 1 | | | 1 | | | | | 1 | |  |  |  |
| P34932 | | Heat shock 70 kDa protein 4 OS=Homo sapiens OX=9606 GN=HSPA4 PE=1 SV=4 | | 94,3 | 5,19 | | 380 | | | 23 | | | 2 | | | 2 | | | 2 | | | | | 3 | |  |  |  |
| P30040 | | Endoplasmic reticulum resident protein 29 OS=Homo sapiens OX=9606 GN=ERP29 PE=1 SV=4 | | 29 | 7,31 | | 179 | | | 22 | | | 1 | | | 1 | | | 1 | | | | | 6 | |  |  |  |
| P01024 | | Complement C3 OS=Homo sapiens OX=9606 GN=C3 PE=1 SV=2 | | 187 | 6,4 | | 247 | | | 22 | | | 1 | | | 1 | | | 1 | | | | | 1 | |  |  |  |
| O95487 | | Protein transport protein Sec24B OS=Homo sapiens OX=9606 GN=SEC24B PE=1 SV=2 | | 137,3 | 6,67 | | 505 | | | 22 | | | 1 | | | 1 | | | 1 | | | | | 1 | |  |  |  |
| Q8WU90 | | Zinc finger CCCH domain-containing protein 15 OS=Homo sapiens OX=9606 GN=ZC3H15 PE=1 SV=1 | | 48,6 | 5,31 | | 327 | | | 22 | | | 1 | | | 1 | | | 1 | | | | | 3 | |  |  |  |
| Q9ULA0 | | Aspartyl aminopeptidase OS=Homo sapiens OX=9606 GN=DNPEP PE=1 SV=2 | | 53,4 | 7,58 | | 320 | | | 22 | | | 1 | | | 1 | | | 1 | | | | | 2 | |  |  |  |
| P61313 | | Large ribosomal subunit protein eL15 OS=Homo sapiens OX=9606 GN=RPL15 PE=1 SV=2 | | 24,1 | 11,62 | | 647 | | | 21 | | | 2 | | | 2 | | | 2 | | | | | 11 | |  |  |  |
| P78347 | | General transcription factor II-I OS=Homo sapiens OX=9606 GN=GTF2I PE=1 SV=2 | | 112,3 | 6,39 | | 518 | | | 21 | | | 1 | | | 1 | | | 1 | | | | | 1 | |  |  |  |
| Q93009 | | Ubiquitin carboxyl-terminal hydrolase 7 OS=Homo sapiens OX=9606 GN=USP7 PE=1 SV=2 | | 128,2 | 5,55 | | 309 | | | 21 | | | 2 | | | 2 | | | 2 | | | | | 2 | |  |  |  |
| O43583 | | Density-regulated protein OS=Homo sapiens OX=9606 GN=DENR PE=1 SV=2 | | 22,1 | 5,3 | | 205 | | | 21 | | | 1 | | | 1 | | | 1 | | | | | 8 | |  |  |  |
| Q8IZP2 | | Putative protein FAM10A4 OS=Homo sapiens OX=9606 GN=ST13P4 PE=5 SV=1 | | 27,4 | 5,08 | | 131 | | | 20 | | | 1 | | | 1 | | | 1 | | | | | 6 | |  |  |  |
| P04085 | | Platelet-derived growth factor subunit A OS=Homo sapiens OX=9606 GN=PDGFA PE=1 SV=1 | | 24 | 9,39 | | 170 | | | 20 | | | 1 | | | 1 | | | 1 | | | | | 4 | |  |  |  |
| P05386 | | Large ribosomal subunit protein P1 OS=Homo sapiens OX=9606 GN=RPLP1 PE=1 SV=1 | | 11,5 | 4,32 | | 241 | | | 20 | | | 1 | | | 1 | | | 1 | | | | | 14 | |  |  |  |
| Q9BVG4 | | Protein PBDC1 OS=Homo sapiens OX=9606 GN=PBDC1 PE=1 SV=1 | | 26 | 4,79 | | 645 | | | 20 | | | 1 | | | 1 | | | 1 | | | | | 6 | |  |  |  |
| **Influenza A virus (IAV)** | | | | | | | | | | | | | | | | | | | | | | | | | | |  |  |
| **ACCESSION** | | | **DESCRIPTION** | | **MW [kDa]** | **calc. pI** | | **Protein Group IDs** | | | **Score Mascot: Mascot** | | | **#PSMs (by Search Engine): Mascot** | | | **#Peptides (by Search Engine): Mascot** | | | **#PSMs** | | | | | **#Peptides** | |  |  |
| Q7Z406 | | | Myosin-14 OS=Homo sapiens OX=9606 GN=MYH14 PE=1 SV=2 | | 227,7 | | | 5,6 | | 566 | | | | 4469 | | 91 | | | 69 | | | | 60 | 40 | | | | |
| P32455 | | | **Guanylate-binding protein 1** OS=Homo sapiens OX=9606 GN=GBP1 PE=1 SV=2 | | 67,9 | | | 6,32 | | | 637 | | | 4275 | | | 475 | | | 69 | | | 52 | | 87 | | | |
| P35749 | | | Myosin-11 OS=Homo sapiens OX=9606 GN=MYH11 PE=1 SV=3 | | 227,2 | | | 5,5 | | | 260 | | | 1434 | | | 37 | | | 23 | | | 1 | | 11 | | | |
| P63261 | | | Actin, cytoplasmic 2 OS=Homo sapiens OX=9606 GN=ACTG1 PE=1 SV=1 | | 41,8 | | | 5,48 | | | 607 | | | 1389 | | | 88 | | | 21 | | | 1 | | 62 | | | |
| Q16643 | | | Drebrin OS=Homo sapiens OX=9606 GN=DBN1 PE=1 SV=4 | | 71,4 | | | 4,45 | | | 374 | | | 1384 | | | 33 | | | 25 | | | 25 | | 41 | | | |
| O00159 | | | Unconventional myosin-Ic OS=Homo sapiens OX=9606 GN=MYO1C PE=1 SV=4 | | 121,6 | | | 9,41 | | | 36 | | | 1139 | | | 25 | | | 23 | | | 23 | | 25 | | | |
| P06753 | | | Tropomyosin alpha-3 chain OS=Homo sapiens OX=9606 GN=TPM3 PE=1 SV=2 | | 32,9 | | | 4,72 | | | 636 | | | 1064 | | | 24 | | | 20 | | | 6 | | 54 | | | |
| P67936 | | | Tropomyosin alpha-4 chain OS=Homo sapiens OX=9606 GN=TPM4 PE=1 SV=3 | | 28,5 | | | 4,69 | | | 350 | | | 1055 | | | 24 | | | 18 | | | 10 | | 49 | | | |
| P09493 | | | Tropomyosin alpha-1 chain OS=Homo sapiens OX=9606 GN=TPM1 PE=1 SV=2 | | 32,7 | | | 4,74 | | | 436 | | | 1034 | | | 25 | | | 18 | | | 6 | | 50 | | | |
| Q92614 | | | Unconventional myosin-XVIIIa OS=Homo sapiens OX=9606 GN=MYO18A PE=1 SV=3 | | 233 | | | 6,3 | | | 52 | | | 998 | | | 23 | | | 23 | | | 23 | | 15 | | | |
| P14649 | | | Myosin light chain 6B OS=Homo sapiens OX=9606 GN=MYL6B PE=1 SV=1 | | 22,8 | | | 5,73 | | | 635 | | | 981 | | | 19 | | | 16 | | | 15 | | 61 | | | |
| Q6WCQ1 | | | Myosin phosphatase Rho-interacting protein OS=Homo sapiens OX=9606 GN=MPRIP PE=1 SV=3 | | 116,5 | | | 6,21 | | | 138 | | | 841 | | | 16 | | | 16 | | | 16 | | 21 | | | |
| Q9H0R5 | | | Guanylate-binding protein 3 OS=Homo sapiens OX=9606 GN=GBP3 PE=1 SV=3 | | 68,1 | | | 6,51 | | | 412 | | | 820 | | | 96 | | | 15 | | | 1 | | 22 | | | |
| Q9Y608 | | | Leucine-rich repeat flightless-interacting protein 2 OS=Homo sapiens OX=9606 GN=LRRFIP2 PE=1 SV=1 | | 82,1 | | | 6,95 | | | 291 | | | 801 | | | 17 | | | 14 | | | 14 | | 21 | | | |
| P68032 | | | Actin, alpha cardiac muscle 1 OS=Homo sapiens OX=9606 GN=ACTC1 PE=1 SV=1 | | 42 | | | 5,39 | | | 600 | | | 780 | | | 58 | | | 15 | | | 3 | | 41 | | | |
| Q9P0K7 | | | Ankycorbin OS=Homo sapiens OX=9606 GN=RAI14 PE=1 SV=2 | | 110 | | | 6,21 | | | 433 | | | 707 | | | 21 | | | 20 | | | 20 | | 26 | | | |
| P52907 | | | F-actin-capping protein subunit alpha-1 OS=Homo sapiens OX=9606 GN=CAPZA1 PE=1 SV=3 | | 32,9 | | | 5,69 | | | 615 | | | 703 | | | 13 | | | 9 | | | 7 | | 55 | | | |
| P61158 | | | Actin-related protein 3 OS=Homo sapiens OX=9606 GN=ACTR3 PE=1 SV=3 | | 47,3 | | | 5,88 | | | 576 | | | 656 | | | 15 | | | 12 | | | 12 | | 36 | | | |
| O94832 | | | Unconventional myosin-Id OS=Homo sapiens OX=9606 GN=MYO1D PE=1 SV=2 | | 116,1 | | | 9,39 | | | 579 | | | 511 | | | 15 | | | 15 | | | 15 | | 18 | | | |
| P61160 | | | Actin-related protein 2 OS=Homo sapiens OX=9606 GN=ACTR2 PE=1 SV=1 | | 44,7 | | | 6,74 | | | 277 | | | 489 | | | 11 | | | 8 | | | 8 | | 22 | | | |
| Q13045 | | | Protein flightless-1 homolog OS=Homo sapiens OX=9606 GN=FLII PE=1 SV=2 | | 144,7 | | | 6,05 | | | 263 | | | 407 | | | 11 | | | 11 | | | 11 | | 10 | | | |
| P0DTE7 | | | Alpha-amylase 1B OS=Homo sapiens OX=9606 GN=AMY1B PE=1 SV=1 | | 57,7 | | | 6,93 | | | 84 | | | 401 | | | 12 | | | 10 | | | 10 | | 26 | | | |
| O60784 | | | Target of Myb1 membrane trafficking protein OS=Homo sapiens OX=9606 GN=TOM1 PE=1 SV=2 | | 53,8 | | | 4,7 | | | 75 | | | 392 | | | 9 | | | 8 | | | 8 | | 24 | | | |
| P32456 | | | Guanylate-binding protein 2 OS=Homo sapiens OX=9606 GN=GBP2 PE=1 SV=3 | | 67,2 | | | 5,71 | | | 618 | | | 388 | | | 43 | | | 8 | | | 2 | | 17 | | | |
| O15144 | | | Actin-related protein 2/3 complex subunit 2 OS=Homo sapiens OX=9606 GN=ARPC2 PE=1 SV=1 | | 34,3 | | | 7,36 | | | 657 | | | 385 | | | 12 | | | 10 | | | 10 | | 33 | | | |
| Q92747 | | | Actin-related protein 2/3 complex subunit 1A OS=Homo sapiens OX=9606 GN=ARPC1A PE=1 SV=2 | | 41,5 | | | 8,18 | | | 448 | | | 372 | | | 11 | | | 9 | | | 9 | | 31 | | | |
| O43795 | | | Unconventional myosin-Ib OS=Homo sapiens OX=9606 GN=MYO1B PE=1 SV=3 | | 131,9 | | | 9,38 | | | 538 | | | 355 | | | 10 | | | 10 | | | 10 | | 8 | | | |
| O15143 | | | Actin-related protein 2/3 complex subunit 1B OS=Homo sapiens OX=9606 GN=ARPC1B PE=1 SV=3 | | 40,9 | | | 8,35 | | | 547 | | | 340 | | | 7 | | | 6 | | | 6 | | 17 | | | |
| Q9NZR1 | | | Tropomodulin-2 OS=Homo sapiens OX=9606 GN=TMOD2 PE=1 SV=1 | | 39,6 | | | 5,27 | | | 257 | | | 301 | | | 8 | | | 7 | | | 7 | | 24 | | | |
| Q69YQ0 | | | Cytospin-A OS=Homo sapiens OX=9606 GN=SPECC1L PE=1 SV=3 | | 124,5 | | | 5,76 | | | 676 | | | 270 | | | 7 | | | 7 | | | 7 | | 8 | | | |
| O14974 | | | Protein phosphatase 1 regulatory subunit 12A OS=Homo sapiens OX=9606 GN=PPP1R12A PE=1 SV=1 | | 115,2 | | | 5,4 | | | 56 | | | 267 | | | 4 | | | 4 | | | 4 | | 5 | | | |
| P61981 | | | 14-3-3 protein gamma OS=Homo sapiens OX=9606 GN=YWHAG PE=1 SV=2 | | 28,3 | | | 4,89 | | | 370 | | | 247 | | | 5 | | | 5 | | | 1 | | 19 | | | |
| P62140 | | | Serine/threonine-protein phosphatase PP1-beta catalytic subunit OS=Homo sapiens OX=9606 GN=PPP1CB PE=1 SV=3 | | 37,2 | | | 6,19 | | | 521 | | | 245 | | | 6 | | | 5 | | | 1 | | 17 | | | |
| Q8WWI1 | | | LIM domain only protein 7 OS=Homo sapiens OX=9606 GN=LMO7 PE=1 SV=3 | | 192,6 | | | 8,09 | | | 178 | | | 224 | | | 5 | | | 5 | | | 5 | | 5 | | | |
| Q9H0E2 | | | Toll-interacting protein OS=Homo sapiens OX=9606 GN=TOLLIP PE=1 SV=1 | | 30,3 | | | 5,97 | | | 401 | | | 219 | | | 4 | | | 4 | | | 4 | | 16 | | | |
| O15511 | | | Actin-related protein 2/3 complex subunit 5 OS=Homo sapiens OX=9606 GN=ARPC5 PE=1 SV=3 | | 16,3 | | | 5,67 | | | 282 | | | 211 | | | 3 | | | 3 | | | 2 | | 26 | | | |
| Q9BPX5 | | | Actin-related protein 2/3 complex subunit 5-like protein OS=Homo sapiens OX=9606 GN=ARPC5L PE=1 SV=1 | | 16,9 | | | 6,6 | | | 146 | | | 179 | | | 5 | | | 4 | | | 3 | | 30 | | | |
| Q16576 | | | Histone-binding protein RBBP7 OS=Homo sapiens OX=9606 GN=RBBP7 PE=1 SV=1 | | 47,8 | | | 5,05 | | | 50 | | | 165 | | | 3 | | | 3 | | | 3 | | 7 | | | |
| Q96N67 | | | Dedicator of cytokinesis protein 7 OS=Homo sapiens OX=9606 GN=DOCK7 PE=1 SV=4 | | 242,4 | | | 6,8 | | | 242 | | | 160 | | | 6 | | | 5 | | | 5 | | 3 | | | |
| P02810 | | | Salivary acidic proline-rich phosphoprotein 1/2 OS=Homo sapiens OX=9606 GN=PRH1 PE=1 SV=3 | | 17 | | | 4,96 | | | 546 | | | 154 | | | 5 | | | 3 | | | 3 | | 26 | | | |
| Q96SB3 | | | Neurabin-2 OS=Homo sapiens OX=9606 GN=PPP1R9B PE=1 SV=3 | | 89,3 | | | 4,97 | | | 103 | | | 153 | | | 4 | | | 4 | | | 3 | | 6 | | | |
| Q0ZGT2 | | | Nexilin OS=Homo sapiens OX=9606 GN=NEXN PE=1 SV=1 | | 80,6 | | | 5,33 | | | 476 | | | 142 | | | 4 | | | 4 | | | 4 | | 5 | | | |
| Q02880 | | | DNA topoisomerase 2-beta OS=Homo sapiens OX=9606 GN=TOP2B PE=1 SV=3 | | 183,2 | | | 8 | | | 97 | | | 139 | | | 7 | | | 7 | | | 2 | | 5 | | | |
| Q04118 | | | Basic salivary proline-rich protein 3 OS=Homo sapiens OX=9606 GN=PRB3 PE=1 SV=3 | | 35,1 | | | 10,84 | | | 678 | | | 134 | | | 4 | | | 3 | | | 3 | | 9 | | | |
| Q9NQX4 | | | Unconventional myosin-Vc OS=Homo sapiens OX=9606 GN=MYO5C PE=1 SV=2 | | 202,7 | | | 7,71 | | | 338 | | | 134 | | | 2 | | | 2 | | | 1 | | 1 | | | |
| Q9ULV0 | | | Unconventional myosin-Vb OS=Homo sapiens OX=9606 GN=MYO5B PE=1 SV=3 | | 213,5 | | | 7,2 | | | 318 | | | 134 | | | 2 | | | 2 | | | 1 | | 1 | | | |
| O14818 | | | Proteasome subunit alpha type-7 OS=Homo sapiens OX=9606 GN=PSMA7 PE=1 SV=1 | | 27,9 | | | 8,46 | | | 632 | | | 130 | | | 4 | | | 4 | | | 4 | | 19 | | | |
| Q96DA0 | | | Pancreatic adenocarcinoma up-regulated factor OS=Homo sapiens OX=9606 GN=ZG16B PE=1 SV=4 | | 18,9 | | | 5,5 | | | 423 | | | 120 | | | 5 | | | 4 | | | 4 | | 30 | | | |
| Q12792 | | | Twinfilin-1 OS=Homo sapiens OX=9606 GN=TWF1 PE=1 SV=3 | | 40,3 | | | 6,96 | | | 23 | | | 117 | | | 4 | | | 4 | | | 4 | | 11 | | | |
| Q9Y4I1 | | | Unconventional myosin-Va OS=Homo sapiens OX=9606 GN=MYO5A PE=1 SV=2 | | 215,3 | | | 8,48 | | | 267 | | | 111 | | | 2 | | | 2 | | | 1 | | 2 | | | |
| P04083 | | | Annexin A1 OS=Homo sapiens OX=9606 GN=ANXA1 PE=1 SV=2 | | 38,7 | | | 7,02 | | | 514 | | | 107 | | | 3 | | | 3 | | | 3 | | 8 | | | |
| P01833 | | | Polymeric immunoglobulin receptor OS=Homo sapiens OX=9606 GN=PIGR PE=1 SV=4 | | 83,2 | | | 5,74 | | | 390 | | | 104 | | | 1 | | | 1 | | | 1 | | 2 | | | |
| Q7Z2W4 | | | Zinc finger CCCH-type antiviral protein 1 OS=Homo sapiens OX=9606 GN=ZC3HAV1 PE=1 SV=3 | | 101,4 | | | 8,4 | | | 42 | | | 100 | | | 1 | | | 1 | | | 1 | | 2 | | | |
| Q6DD87 | | | Zinc finger protein 787 OS=Homo sapiens OX=9606 GN=ZNF787 PE=1 SV=4 | | 40,4 | | | 7,96 | | | 312 | | | 91 | | | 1 | | | 1 | | | 1 | | 4 | | | |
| Q9BUL8 | | | Programmed cell death protein 10 OS=Homo sapiens OX=9606 GN=PDCD10 PE=1 SV=1 | | 24,7 | | | 8,19 | | | 402 | | | 90 | | | 2 | | | 2 | | | 2 | | 11 | | | |
| P30419 | | | Glycylpeptide N-tetradecanoyltransferase 1 OS=Homo sapiens OX=9606 GN=NMT1 PE=1 SV=2 | | 56,8 | | | 7,8 | | | 587 | | | 80 | | | 2 | | | 2 | | | 2 | | 5 | | | |
| Q6IS14 | | | Eukaryotic translation initiation factor 5A-1-like OS=Homo sapiens OX=9606 GN=EIF5AL1 PE=1 SV=2 | | 16,8 | | | 5 | | | 121 | | | 78 | | | 2 | | | 2 | | | 2 | | 13 | | | |
| Q14247 | | | Src substrate cortactin OS=Homo sapiens OX=9606 GN=CTTN PE=1 SV=2 | | 61,5 | | | 5,4 | | | 132 | | | 77 | | | 2 | | | 2 | | | 2 | | 4 | | | |
| P01037 | | | Cystatin-SN OS=Homo sapiens OX=9606 GN=CST1 PE=1 SV=3 | | 16,4 | | | 7,21 | | | 195 | | | 75 | | | 2 | | | 2 | | | 1 | | 21 | | | |
| O15145 | | | Actin-related protein 2/3 complex subunit 3 OS=Homo sapiens OX=9606 GN=ARPC3 PE=1 SV=3 | | 20,5 | | | 8,59 | | | 110 | | | 75 | | | 4 | | | 4 | | | 4 | | 24 | | | |
| Q96B26 | | | Exosome complex component RRP43 OS=Homo sapiens OX=9606 GN=EXOSC8 PE=1 SV=1 | | 30 | | | 5,3 | | | 315 | | | 75 | | | 1 | | | 1 | | | 1 | | 7 | | | |
| O14639 | | | Actin-binding LIM protein 1 OS=Homo sapiens OX=9606 GN=ABLIM1 PE=1 SV=3 | | 87,6 | | | 8,59 | | | 663 | | | 71 | | | 1 | | | 1 | | | 1 | | 2 | | | |
| Q9UBG3 | | | Cornulin OS=Homo sapiens OX=9606 GN=CRNN PE=1 SV=1 | | 53,5 | | | 6,1 | | | 595 | | | 71 | | | 3 | | | 3 | | | 3 | | 8 | | | |
| Q9H4A3 | | | Serine/threonine-protein kinase WNK1 OS=Homo sapiens OX=9606 GN=WNK1 PE=1 SV=2 | | 250,6 | | | 6,34 | | | 512 | | | 71 | | | 1 | | | 1 | | | 1 | | 1 | | | |
| P06493 | | | Cyclin-dependent kinase 1 OS=Homo sapiens OX=9606 GN=CDK1 PE=1 SV=3 | | 34,1 | | | 8,4 | | | 181 | | | 68 | | | 2 | | | 2 | | | 2 | | 6 | | | |
| P28482 | | | Mitogen-activated protein kinase 1 OS=Homo sapiens OX=9606 GN=MAPK1 PE=1 SV=3 | | 41,4 | | | 6,98 | | | 302 | | | 67 | | | 1 | | | 1 | | | 1 | | 4 | | | |
| Q9H444 | | | Charged multivesicular body protein 4b OS=Homo sapiens OX=9606 GN=CHMP4B PE=1 SV=1 | | 24,9 | | | 4,82 | | | 31 | | | 66 | | | 1 | | | 1 | | | 1 | | 6 | | | |
| Q9ULJ8 | | | Neurabin-1 OS=Homo sapiens OX=9606 GN=PPP1R9A PE=1 SV=2 | | 123,3 | | | 5,1 | | | 517 | | | 61 | | | 4 | | | 4 | | | 3 | | 4 | | | |
| P62333 | | | 26S proteasome regulatory subunit 10B OS=Homo sapiens OX=9606 GN=PSMC6 PE=1 SV=1 | | 44,1 | | | 7,49 | | | 274 | | | 57 | | | 2 | | | 2 | | | 2 | | 5 | | | |
| Q96IZ0 | | | PRKC apoptosis WT1 regulator protein OS=Homo sapiens OX=9606 GN=PAWR PE=1 SV=1 | | 36,5 | | | 5,41 | | | 623 | | | 53 | | | 1 | | | 1 | | | 1 | | 5 | | | |
| Q16543 | | | Hsp90 co-chaperone Cdc37 OS=Homo sapiens OX=9606 GN=CDC37 PE=1 SV=1 | | 44,4 | | | 5,25 | | | 565 | | | 49 | | | 2 | | | 2 | | | 2 | | 7 | | | |
| Q9BQI0 | | | Allograft inflammatory factor 1-like OS=Homo sapiens OX=9606 GN=AIF1L PE=1 SV=1 | | 17,1 | | | 7,2 | | | 551 | | | 49 | | | 1 | | | 1 | | | 1 | | 7 | | | |
| O15182 | | | Centrin-3 OS=Homo sapiens OX=9606 GN=CETN3 PE=1 SV=2 | | 19,5 | | | 4,74 | | | 60 | | | 49 | | | 1 | | | 1 | | | 1 | | 7 | | | |
| P20618 | | | Proteasome subunit beta type-1 OS=Homo sapiens OX=9606 GN=PSMB1 PE=1 SV=2 | | 26,5 | | | 8,13 | | | 180 | | | 44 | | | 2 | | | 2 | | | 2 | | 10 | | | |
| Q92922 | | | SWI/SNF complex subunit SMARCC1 OS=Homo sapiens OX=9606 GN=SMARCC1 PE=1 SV=3 | | 122,8 | | | 5,76 | | | 197 | | | 44 | | | 1 | | | 1 | | | 1 | | 1 | | | |
| P01859 | | | Immunoglobulin heavy constant gamma 2 OS=Homo sapiens OX=9606 GN=IGHG2 PE=1 SV=3 | | 43,8 | | | 6,52 | | | 12 | | | 43 | | | 1 | | | 1 | | | 1 | | 2 | | | |
| Q5T750 | | | Protein KPLCE OS=Homo sapiens OX=9606 GN=KPLCE PE=1 SV=1 | | 26,2 | | | 7,97 | | | 569 | | | 40 | | | 1 | | | 1 | | | 1 | | 3 | | | |
| Q05682 | | | Caldesmon OS=Homo sapiens OX=9606 GN=CALD1 PE=1 SV=3 | | 93,2 | | | 5,66 | | | 316 | | | 38 | | | 1 | | | 1 | | | 1 | | 2 | | | |
| Q16186 | | | Proteasomal ubiquitin receptor ADRM1 OS=Homo sapiens OX=9606 GN=ADRM1 PE=1 SV=2 | | 42,1 | | | 5,07 | | | 516 | | | 38 | | | 1 | | | 1 | | | 1 | | 4 | | | |
| P49720 | | | Proteasome subunit beta type-3 OS=Homo sapiens OX=9606 GN=PSMB3 PE=1 SV=2 | | 22,9 | | | 6,55 | | | 102 | | | 37 | | | 1 | | | 1 | | | 1 | | 3 | | | |
| P43686 | | | 26S proteasome regulatory subunit 6B OS=Homo sapiens OX=9606 GN=PSMC4 PE=1 SV=2 | | 47,3 | | | 5,21 | | | 535 | | | 36 | | | 2 | | | 2 | | | 2 | | 5 | | | |
| P33993 | | | DNA replication licensing factor MCM7 OS=Homo sapiens OX=9606 GN=MCM7 PE=1 SV=4 | | 81,3 | | | 6,46 | | | 340 | | | 36 | | | 1 | | | 1 | | | 1 | | 2 | | | |
| Q99436 | | | Proteasome subunit beta type-7 OS=Homo sapiens OX=9606 GN=PSMB7 PE=1 SV=1 | | 29,9 | | | 7,68 | | | 136 | | | 34 | | | 1 | | | 1 | | | 1 | | 4 | | | |
| Q9Y5K5 | | | Ubiquitin carboxyl-terminal hydrolase isozyme L5 OS=Homo sapiens OX=9606 GN=UCHL5 PE=1 SV=3 | | 37,6 | | | 5,33 | | | 230 | | | 32 | | | 1 | | | 1 | | | 1 | | 2 | | | |
| Q9UNM6 | | | 26S proteasome non-ATPase regulatory subunit 13 OS=Homo sapiens OX=9606 GN=PSMD13 PE=1 SV=2 | | 42,9 | | | 5,81 | | | 480 | | | 30 | | | 2 | | | 2 | | | 2 | | 4 | | | |
| O00232 | | | 26S proteasome non-ATPase regulatory subunit 12 OS=Homo sapiens OX=9606 GN=PSMD12 PE=1 SV=3 | | 52,9 | | | 7,65 | | | 251 | | | 30 | | | 1 | | | 1 | | | 1 | | 2 | | | |
| Q86UK7 | | | E3 ubiquitin-protein ligase ZNF598 OS=Homo sapiens OX=9606 GN=ZNF598 PE=1 SV=1 | | 98,6 | | | 8,4 | | | 113 | | | 29 | | | 1 | | | 1 | | | 1 | | 1 | | | |
| Q15517 | | | Corneodesmosin OS=Homo sapiens OX=9606 GN=CDSN PE=1 SV=4 | | 51,6 | | | 8,44 | | | 283 | | | 27 | | | 1 | | | 1 | | | 1 | | 2 | | | |
| P55735 | | | Protein SEC13 homolog OS=Homo sapiens OX=9606 GN=SEC13 PE=1 SV=3 | | 35,5 | | | 5,48 | | | 422 | | | 25 | | | 1 | | | 1 | | | 1 | | 8 | | | |
| Q53GQ0 | | | Very-long-chain 3-oxoacyl-CoA reductase OS=Homo sapiens OX=9606 GN=HSD17B12 PE=1 SV=2 | | 34,3 | | | 9,32 | | | 240 | | | 25 | | | 1 | | | 1 | | | 1 | | 4 | | | |
| P01036 | | | Cystatin-S OS=Homo sapiens OX=9606 GN=CST4 PE=1 SV=3 | | 16,2 | | | 5,02 | | | 135 | | | 24 | | | 2 | | | 2 | | | 1 | | 21 | | | |
| Q6ZVM7 | | | TOM1-like protein 2 OS=Homo sapiens OX=9606 GN=TOM1L2 PE=1 SV=1 | | 55,5 | | | 4,79 | | | 525 | | | 24 | | | 1 | | | 1 | | | 1 | | 1 | | | |
| P09496 | | | Clathrin light chain A OS=Homo sapiens OX=9606 GN=CLTA PE=1 SV=1 | | 27,1 | | | 4,51 | | | 668 | | | 23 | | | 1 | | | 1 | | | 1 | | 3 | | | |
| Q9UQE7 | | | Structural maintenance of chromosomes protein 3 OS=Homo sapiens OX=9606 GN=SMC3 PE=1 SV=2 | | 141,5 | | | 7,18 | | | 592 | | | 23 | | | 1 | | | 1 | | | 1 | | 2 | | | |
| Q7Z7B0 | | | Filamin-A-interacting protein 1 OS=Homo sapiens OX=9606 GN=FILIP1 PE=1 SV=1 | | 138 | | | 8,32 | | | 268 | | | 23 | | | 2 | | | 1 | | | 1 | | 1 | | | |
| Q9H2G2 | | | STE20-like serine/threonine-protein kinase OS=Homo sapiens OX=9606 GN=SLK PE=1 SV=1 | | 142,6 | | | 5,15 | | | 301 | | | 21 | | | 1 | | | 1 | | | 1 | | 1 | | | |
| Q9H0A0 | | | RNA cytidine acetyltransferase OS=Homo sapiens OX=9606 GN=NAT10 PE=1 SV=2 | | 115,7 | | | 8,27 | | | 82 | | | 21 | | | 1 | | | 1 | | | 1 | | 1 | | | |
| P41250 | | | Glycine--tRNA ligase OS=Homo sapiens OX=9606 GN=GARS1 PE=1 SV=3 | | 83,1 | | | 7,03 | | | 633 | | | 20 | | | 1 | | | 1 | | | 1 | | 1 | | | |
|  | | |  | |  | | |  | |  | | |  | | |  | | |  | | |  | |  | | | | |

**Table E. Qualitative proteomic analysis of proteins detected exclusively in GBP1-FLAG–overexpressing cells.** Proteins were identified by mass spectrometry following FLAG pull-down, and only those uniquely present in GBP1-FLAG–overexpressing cells compared with control cells (transfected with the empty plasmid) are shown. Protein accession, description, molecular weight, isoelectric point, group ID, Mascot score, PSMs, peptides, unique peptides, and sequence coverage are shown, ordered by Mascot score.

**Table F.**

| **Strand-specific primers used for reverse transcription (RT)** | |
| --- | --- |
| **Target** | **Sequences (5’-3’)** |
| **PB2-mRNA** | CCAGATCGTTCGAGTCGTTTTTTTTTTTTTTTTTAAACTATTCGA |
| **HA-mRNA** | CCAGATCGTTCGAGTCGTTTTTTTTTTTTTTTTTCCTCATATTTCT |
| **NA-mRNA** | CCAGATCGTTCGAGTCGTTTTTTTTTTTTTTTTT GAACAAACTAC |
| **NP-mRNA** | CCAGATCGTTCGAGTCGTTTTTTTTTTTTTTTTTCTTTAATTGTC |
| **NP-vRNA** | GGCCGTCATGGTGGCGAATGAATGGACGAAAAACAAGAATTGC |

**Table G.**

| **Gene-specific primers used for qPCR analysis** | | |
| --- | --- | --- |
| **Target** | **Forward (5’-3')** | **Reverse (5’-3')** |
| **PB2-mRNA** | CCAGATCGTTCGAGTCGT | GGAGATATGGGCCAGCATTA |
| **HA-mRNA** | CCAGATCGTTCGAGTCGT | GGGCAATCAGTTTCTGGATGTGTTCT |
| **NA-mRNA** | CCAGATCGTTCGAGTCGT | TGAATAGTGATACTGTAGATTGGTCT |
| **NP-mRNA** | CCAGATCGTTCGAGTCGT | CGATCGTGCCTTCCTTTG |
| **NP-vRNA** | GGCCGTCATGGTGGCGAAT | CTCAATATGAGTGCAGACCGTGCT |
